# Machine learning algorithm predicts fibrosis-related blood diagnosis markers of intervertebral disc degeneration

**DOI:** 10.1186/s12920-023-01705-6

**Published:** 2023-11-01

**Authors:** Wei Zhao, Jinzheng Wei, Xinghua Ji, Erlong Jia, Jinhu Li, Jianzhong Huo

**Affiliations:** 1 First Hospital of Shanxi Medical University, Taiyuan, Shanxi Province PR China; 2 Shanxi Medical University, Taiyuan, Shanxi Province PR China; 3 Tongji Shanxi Hospital, Shanxi Bethune Hospital, Shanxi Academy of Medical Sciences, Third Hospital of Shanxi Medical University, Taiyuan, Shanxi Province PR China; 4 Taiyuan Central Hospital of Shanxi Medical University, Taiyuan, Shanxi Province PR China

**Keywords:** Intervertebral disc degeneration, Fibrosis, Diagnostic genes, GSEA, Immune Infiltration, Regulatory network

## Abstract

**Background:**

Intervertebral disc cell fibrosis has been established as a contributing factor to intervertebral disc degeneration (IDD). This study aimed to identify fibrosis-related diagnostic genes for patients with IDD.

**Methods:**

RNA-sequencing data was downloaded from Gene Expression Omnibus (GEO) database. The diagnostic genes was identified using Random forest based on the differentially expressed fibrosis-related genes (DE-FIGs) between IDD and control samples. The immune infiltration states in IDD and the regulatory network as well as potential drugs targeted diagnostic genes were investigated. Quantitative Real-Time PCR was conducted for gene expression valifation.

**Results:**

*CEP120* and *SPDL1* merged as diagnostic genes. Substantial variations were observed in the proportions of natural killer cells, neutrophils, and myeloid-derived suppressor cells between IDD and control samples. Further experiments indicated that *AC144548.1* could regulate the expressions of *SPDL1* and *CEP120* by combining*hsa-miR-5195-3p* and *hsa-miR-455-3p*, respectively. Additionally, transcription factors FOXM1, PPARG, and ATF3 were identified as regulators of *SPDL1* and *CEP120* transcription. Notably, 56 drugs were predicted to target these genes. The down-regulation of *SPDL1* and *CEP120* was also validated.

**Conclusion:**

This study identified two diagnostic genes associated with fibrosis in patients with IDD. Additionally, we elucidated their potential regulatory networks and identified target drugs, which offer a theoretical basis and reference for further study into fibrosis-related genes involved in IDD.

**Supplementary Information:**

The online version contains supplementary material available at 10.1186/s12920-023-01705-6.

## Introduction

Intervertebral disc degeneration (IDD) is one of the leading causes of low back pain (LBP) affecting ~ 40% of adults worldwide [1]. This condition is frequently linked to cervical spondylosis, lumbar disc herniation, lumbar spinal stenosis, and lumbar spondylolisthesis [2]. Patients with IDD often experience high disability rates, which significantly affects their quality of life and imposes substantial economic burdens [3]. The intervertebral discs consist of the annulus fibrosus (AF), the nucleus pulposus (NP), and the hyaline cartilage plates of the upper and lower vertebral bodies. IDD mainly involves structural damage to the intervertebral disc and is characterized by an imbalance between catabolic and anabolic processes [4]. This encompasses myeloid nucleus senescence and apoptosis, progressive degradation of the extracellular matrix (ECM), fibro-annular fibrosis culminating in disc bulging, loss of NP and water content, and diminished disc height [5, 6]. Therefore, assessment frameworks like the Pfirrmann grading criteria are employed, relying on disc height, structure, and magnetic resonance imaging signal intensity to clinically classify degeneration levels as stages I–V [7, 8]. Besides, the etiology and pathogenesis of IDD are complex. On the one hand, factors such as age, genetics (e.g., polymorphisms in proteoglycan and collagen-encoding genes), and lifestyle (e.g., occupation, smoking, lack of physical activity, and night work) play contributory roles [9]. On the other hand, cellular processes such as apoptosis, autophagy, and the release of pro-inflammatory factors are involved. Moreover, the pathogenesis of IDD is also heritable, although the exact genetic and molecular mechanisms are still under investigation.

Fibrosis, a pathological process that occurs in various organs, is usually caused by dysregulation of tissue repair in response to chronic inflammation. This can lead to ECM remodeling and excessive accumulation of ECM components [10], consequently altering tissue biomechanical properties. It has been found that alterations in the concentration and morphology of types I and II collagen, and fibronectin during disc fibrosis are closely associated with the progression of disc degeneration [11, 12]. Moreover, myofibroblasts (MF) and macrophages are the main triggers for the progression of fibrosis [10, 13]. Within fibrotic tissues, the regulation of MFs allows for collagen deposition in the ECM, which in turn alters the structure and function of the tissue [14–16]. Also, it was found that MFs were involved in AF repair during IDD [17]. Macrophages can regulate cellular fibrosis through transglutaminases and matrix metalloproteinases (MMPs), which are present in herniated discs and have been widely reported; however, only MMP12 has been identified as a fibrosis marker in IDD [14, 18–22].

In this study, we downloaded two datasets (GSE150408 and GSE124272) from the Gene Expression Omnibus (GEO) database and analyzed them separately to obtain 336 differentially expressed genes (DEGs). Concurrently, we obtained a total of 2,539 fibrosis-associated genes (FIGs) from the GeneCards database. By intersecting these with the 336 DEGs, we obtained 29 differentially expressed FIGs (DE-FIGs). Next, using the random forest (RF) model with a fold number of 3, we successfully screened out two diagnostic genes—*CEP120* and *SPDL1*. The expressions of these genes were validated within both the GSE150408 dataset (training set) and the GSE124272 dataset (validation set), and their predictive potential was confirmed through receiver operating characteristic (ROC) curve analysis. Further exploration included single-sample gene set enrichment analysis (ssGSEA), as well as the construction of miRNA-mRNA-transcription factor (TF) networks and competing endogenous RNA (ceRNA) networks. These networks shed light on the underlying mechanisms relevant to the diagnostic genes. Additionally, diagnostic genes-associated drug prediction was also performed. Finally, we verified the significance of the DEGs in blood samples of healthy subjects and patients with IDD using quantitative real-time polymerase chain reaction (qRT-PCR). These efforts culminated in the identification of two diagnostic genes, which provide a theoretical basis and reference value for the study of fibrosis-related genes in IDD.

## Materials and methods

### Data sources

The GSE150408 and GSE124272 data sets were downloaded from the GEO database (http://www.ncbi.nlm.nih.gov/geo/). GSE150408 contains whole blood samples from 17 patients with IDD and 17 controls, whereas GSE124272 includes 8 pairs of whole blood samples from patients with lumbar disc prolapse and healthy controls. Additionally, 8,621 FIGs were obtained from the GeneCards database (http://www.genecards.org/) (Additional file 1). After a screening process based on a relevance score greater than 1, the selection was narrowed down to 2,539 FIGs for subsequent analyses (Additional file 2).

### Identification of DE-FIGs

Limma package 3.44.3 [23] was employed to screen DEGs between IDD samples and controls in GSE124272 and GSE150408 respectively, which were defined as DEGs1 and DEGs2. Then, the common DEGs between DEGs1 and DEGs2 were obtained by overlap analysis and functional enrichment analyses by clusterProfiler v3.16.0 [24] was subsequently conducted with a threshold of *p <* 0.05 [25–27]. Furthermore, the DEGs that intersected with the 2,539 FIGs were classified as DE-FIGs.

### Selection of diagnostic genes and nomogram construction

To obtain diagnostic genes for IDD, eight machine learning algorithms were used, including Logistic Regression (LR), RF by randomForest v4.6-14, Gradient Decision Tree (GBDT), eXtreme Gradient Boosting (XGB) by xgboost v1.4.1.1, Support Vector Machine (SVM) by e1071 v1.7-3 [28], Artificial Neural Network (ANN) by neuralnet v1.44.2 [29], Decision Tree (DT), Adaboost v4.2 [29], and MultinomialNB (MNB) by kalR. For detecting the most suitable model for DE-FIGs, all the samples in the GSE150408 dataset were randomly divided into training sets and validation sets according to an *n*-fold from 1 to 7. The regression analysis of the eight algorithms was conducted in the training set, and the verification was performed in the validation set. The most suitable algorithm was selected according to the comprehensive effects of the *n-*fold and each algorithm. Moreover, the diagnostic genes for IDD were identified based on the area under the ROC curve (AUC) values of the model under different numbers of variables. Finally, a nomogram consisting of the diagnostic genes was constructed, which was validated by the C-index, slope of the calibration curve, and decision curve analysis (DCA) curve.

### Diagnostic effectiveness of the diagnostic genes

To investigate whether the diagnostic genes as a whole could successfully distinguish IDD samples from healthy controls, principal component analysis (PCA) was performed on both the GSE150408 and GSE124272 datasets. Also, ROC curves were drawn for each diagnostic gene as well as collectively for all the diagnostic genes to differentiate between their diagnostic potential in IDD samples in the GSE 150,408 dataset. Simultaneously, similar ROC curves were plotted for the GSE124272 dataset to validate the results.

### ssGSEA

ssGSEA analysis was performed for the diagnostic genes by clusterProfiler v3.16.1, using GSE150408 as the input dataset. The correlation coefficient between a single diagnosis gene expression and each gene in the GSE150408 dataset was calculated and ranked. Then, according to the correlation coefficient sorting, GSEA was applied with the filter conditions: | NES | > 1; adjusted *p* < 0.05; *q* < 0.25 for Gene Ontology (GO) and Kyoto Encyclopedia of Genes and Genomes (KEGG) enrichment analyses.

### Immune infiltration analysis

The GSE150408 dataset was used as input in the MCP-counter algorithm (http://github.com/ebecht/MCPcounter) to calculate the content of eight immune cells (CD8^+^ T cells, T cells, cytotoxic lymphocytes, B lineage, natural killer cells, monocytic lineage, myeloid dendritic cells, and neutrophils), one fibroblast, and one epithelial cell in IDD samples and controls. In addition, single-sample gene set enrichment analysis (ssGSEA) analysis was performed to compute the proportions of 28 types of immune cells in IDD samples and controls in the GSE150408 dataset.

### Construction of miRNA-mRNA-TF and ceRNA regulatory networks

The level of mRNA is regulated by both miRNAs and TFs. Therefore, both miRNAs and TFs for diagnostic genes were predicted. First, the miRWalk website (http://mirwalk.umm.uni-heidelberg.de/) was used to predict miRNAs of diagnostic genes, and the miRNAs with energy < − 25 were selected. ChEA3 (http://amp.pharm.mssm.edu/chea3/) was subsequently utilized to predict TFs of the diagnostic genes, and the TFs with chip-seq were selected. The gene-TF relationship pairs and gene-miRNA relationship pairs were integrated into a miRNA-gene-TF network.

Meanwhile, the selected miRNAs of diagnostic genes acquired above were used to predict their target long non-coding RNAs (lncRNAs) from the Star base website (http://starbase.sysu.edu.cn/) with the selection criteria of clipExpNum > 5. After combining gene-miRNA and miRNA-lncRNA regulation pairs, the ceRNA network was established.

Afterwards, target drugs of diagnostic genes were predicted through the Comparative Toxicogenomics Database (CTD) (http://ctdbase.org/), and a drug-mRNA network was constructed. All the networks were visualized by Cytoscape v3.7.2.

### Validation by qRT-PCR

The qRT-PCR analysis was performed peripheral blood mononuclear cells (PBMCs) isolated from 24 whole blood samples (12 IDD samples vs. 12 Controls) to further investigate the gene expression pattern of signature genes. TRIzol reagent was first used to extract total RNA following the manufacturer’s procedure (Thermo Fisher Scientific, Waltham, MA, USA). After detection of concentration and purity, 1 µg total RNAs were reverse transcribed to synthesize cDNAs using the SureScript-First-strand-cDNA-synthesis-kit (Servicebio, Wuhan, China). A 20 µL reaction system, including 3 µL diluted cDNAs, 5 µL of 2×Universal Blue SYBR Green qPCR Master Mix, and 1 µL each of forward and reverse primer were combined for qRT-PCR reaction with a BIO-RAD CFX96 Touch TM PCR detection system (Bio-Rad, Hercules, CA, USA). The primer sequences of signature genes are shown in Table 1. The amplification conditions included the following parts: 95 °C for 60 s, 40 cycles at 95 °C for 20 s, 55 °C for 20 s, and 72 °C for 30 s. For subsequent melting curve analysis, a single peak represented the reliability of the primer, and gene expression was computed with the 2^−ΔΔCt^ method.

**Table 1:** Primers for qRT-PCR used in the current study

Primer	Sequence
*CEP120* F	AGTTGGCTACTGATCCTGTGG
*CEP120* R	GGAGTTTGATAGGAGTACGCTGT
*SPDL1* F	TGATGACCATGACTGAGCAGA
*SPDL1* R	ATAACTCTTACTGCTTCTGTGCC
internal reference-*GAPDH* F	CCCATCACCATCTTCCAGG
internal reference-*GAPDH* R	CATCACGCCACAGTTTCCC

F: forward; R: reverse

## Results

### A total of 336 common DEGs were identified

**Fig. 1:**
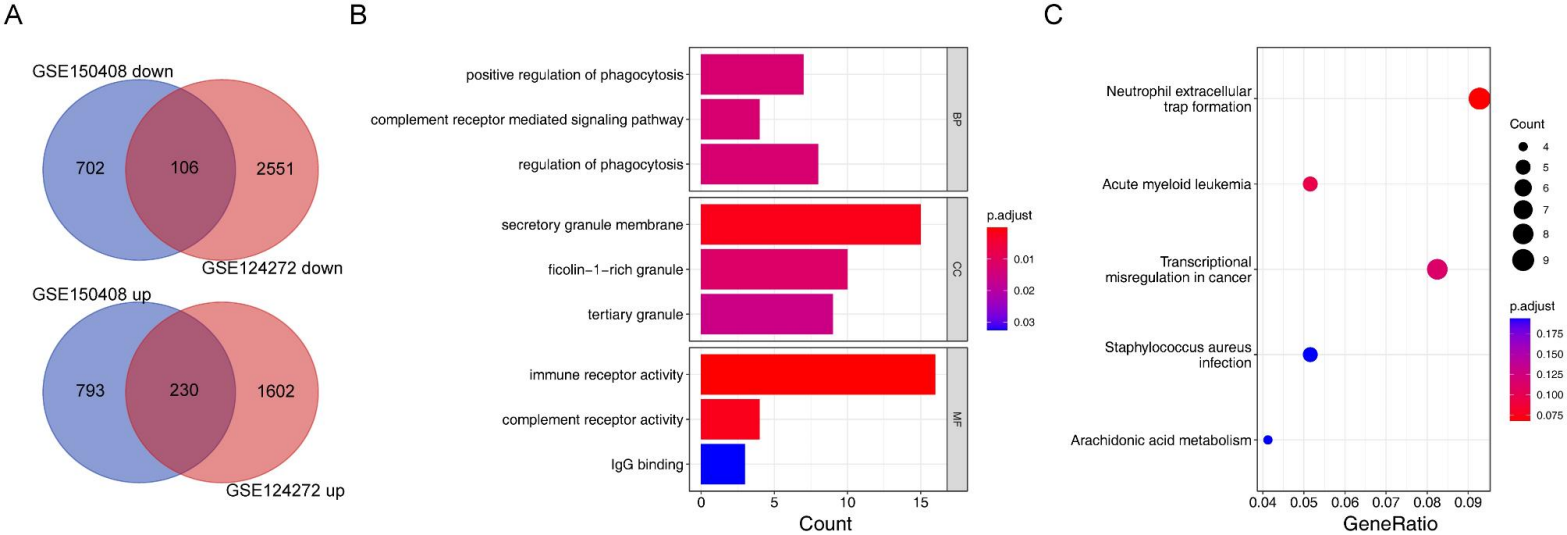
Identification and evaluation of common DEGs between the GSE124272 and GSE150408 datasets. (**A**) Venn diagram of common DEGs. (**B**) The enriched GO terms for 336 common DEGs. The horizontal coordinates represent the gene ratio, and the vertical coordinates represent the GO term. Bubble plots are arranged from smallest to largest *p*-value. (**C**) KEGG bubble chart for 336 common DEGs. The horizontal coordinates represent the gene ratio, and the vertical coordinates represent the terms enriched by KEGG. Bubble plots are sorted by *p*-value from smallest to largest. DEGs, differentially expressed genes; GO, Gene Ontology; KEGG, Kyoto Encyclopedia of Genes and Genomes

According to the differential analysis between IDD and healthy samples, 4,489 DEGs1 and 1,832 DEGs2 were screened out from the GSE124272 and GSE150408 datasets, respectively (Additional files 3–5). After comparing these datasets, 336 common DEGs emerged, which included 230 up-regulated and 106 down-regulated genes (Fig. 1A). The functional enrichment analyses indicated that these 336 common DEGS were associated with 9 KEGG pathways and 9 GO terms (Fig. 1B C). Involvement in the KEGG pathways included, among others, neutrophil extracellular trap formation, acute myeloid leukemia, and transcriptional misregulation in cancer; while for the GO terms it included, amongst others, positive regulation of phagocytosis, complement receptor-mediated signaling pathway, regulation of phagocytosis, secretory granule membrane, and immune receptor activity.

### *CEP120* and *SPDL1* were selected as diagnostic genes for IDD

**Fig. 2:**
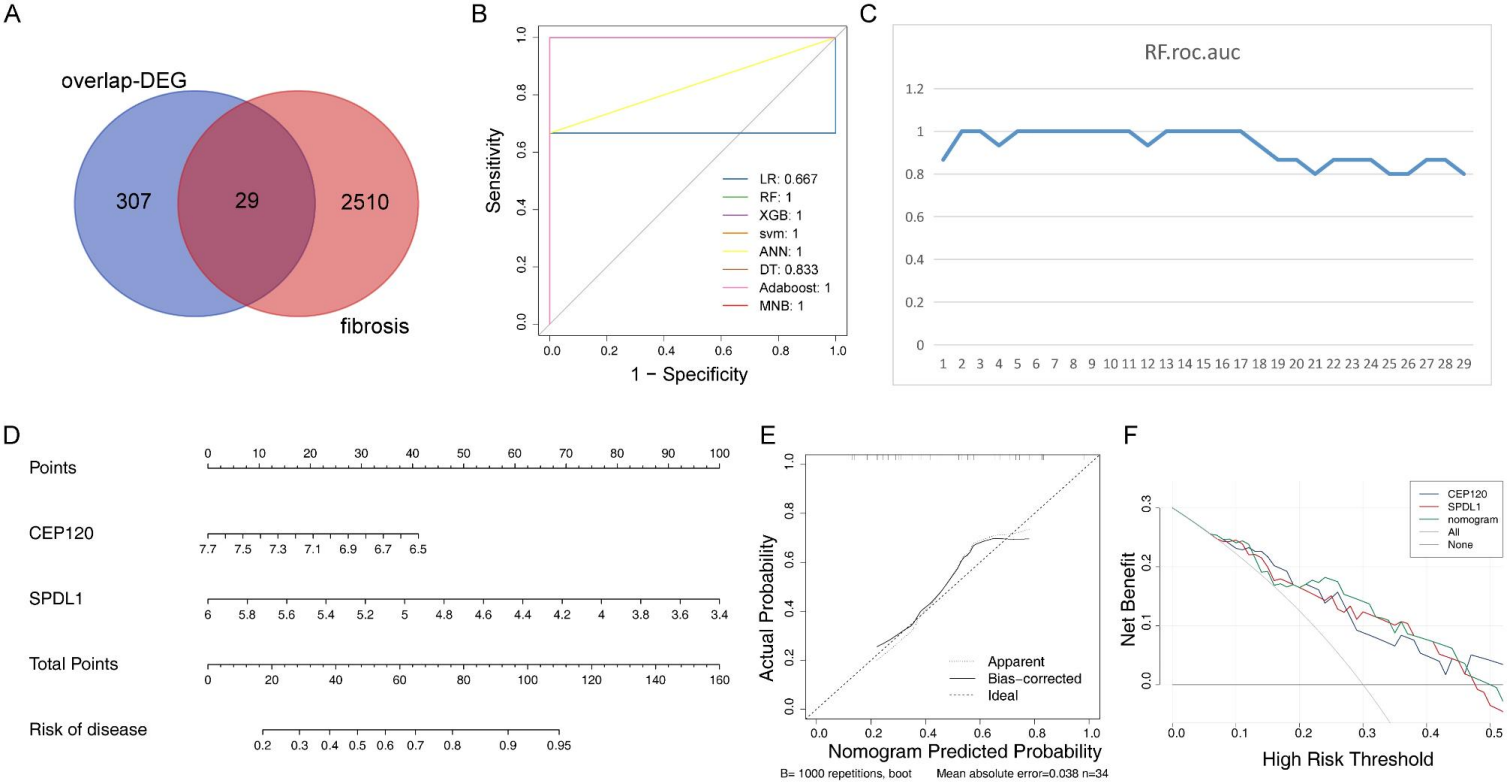
Identification and evaluation of diagnostic genes for IDD. (**A**) Venn diagram of differentially expressed fibrosis genes. (**B**) ROC curves for the eight models at *n-*fold = 3. (**C**) Predicted area under the curve values of RF models with different numbers of variables. (**D**) Nomogram to predict the survival rate of patients with IDD. (**E**) Calibration curve of the nomogram. (**F**) RF modeling for decision. IDD, intervertebral disc degeneration; RF, random forest; ROC, receiver operating characteristic

A total of 29 DE-FIGs were obtained from the overlap analysis between 336 common DEGs and 2,539 FIGs (Additional file 6, Fig. 2A). Moreover, the results of eight machine learning algorithms showed that the optimal fold number was 3 and RF was the most suitable model. As a result, an *n-*fold of 3 for the RF model was selected to construct a diagnostic model (Table 2; Fig. 2B). Subsequently, the RF model generated importance values for each variable, denoted as %IncMSE. Based on these value, the 29 DE-FIGs were ranked from highest to lowest. Using this ranking, the predicted AUC values of the RF model under different variables were displayed in a line chart (Fig. 2C). Notably, it can be observed that when the number of variables reached 2, the AUC value reached 1 and remained at 1 as the number of variables increased. Therefore, these two genes, namely *CEP120* and *SPDL1*, were considered diagnostic genes.

**Table 2:** The *n-*fold of random forest model

*n-*fold	LR	RF	XBG	SVM	ANN	DT		ADABOOST	MNB	Average
1	0.75	0.50	0.75	0.50	1.00		0.75	0.75	0.75	0.72
2	0.75	1.00	0.75	1.00	0.75		0.75	0.75	1.00	0.84
3	0.67	1.00	1.00	1.00	1.00		0.83	1.00	1.00	0.94
4	1.00	1.00	1.00	0.75	0.75		0.50	1.00	0.75	0.84
5	1.00	1.00	1.00	1.00	0.50		0.50	0.75	0.50	0.78
6	0.50	1.00	0.50	1.00	0.50		0.50	0.50	0.50	0.63
7	0.50	1.00	0.50	1.00	0.50		0.50	0.50	0.50	0.63
Average	0.74	0.93	0.79	0.89	0.71		0.62	0.75	0.71	0.77

Further, a nomogram comprising *CEP120* and *SPDL1* was constructed (Fig. 2D) with a C-index of 0.7681661 and a corrected C-index of 0.7326992, indicating that the prediction of the nomogram was accurate (Fig. 2E). Moreover, within the high-risk threshold range from 0 to 1, the nomogram for *CEP120* and *SPDL1* could provide benefit, with the net benefit higher than the curve of each single-gene. This indicated that the overall effectiveness of the nomogram exceeded that of a single gene, affirming the selection of these diagnostic genes as reasonable and effective (Fig. 2F).

### The diagnostic effectiveness of the diagnostic genes

**Fig. 3:**
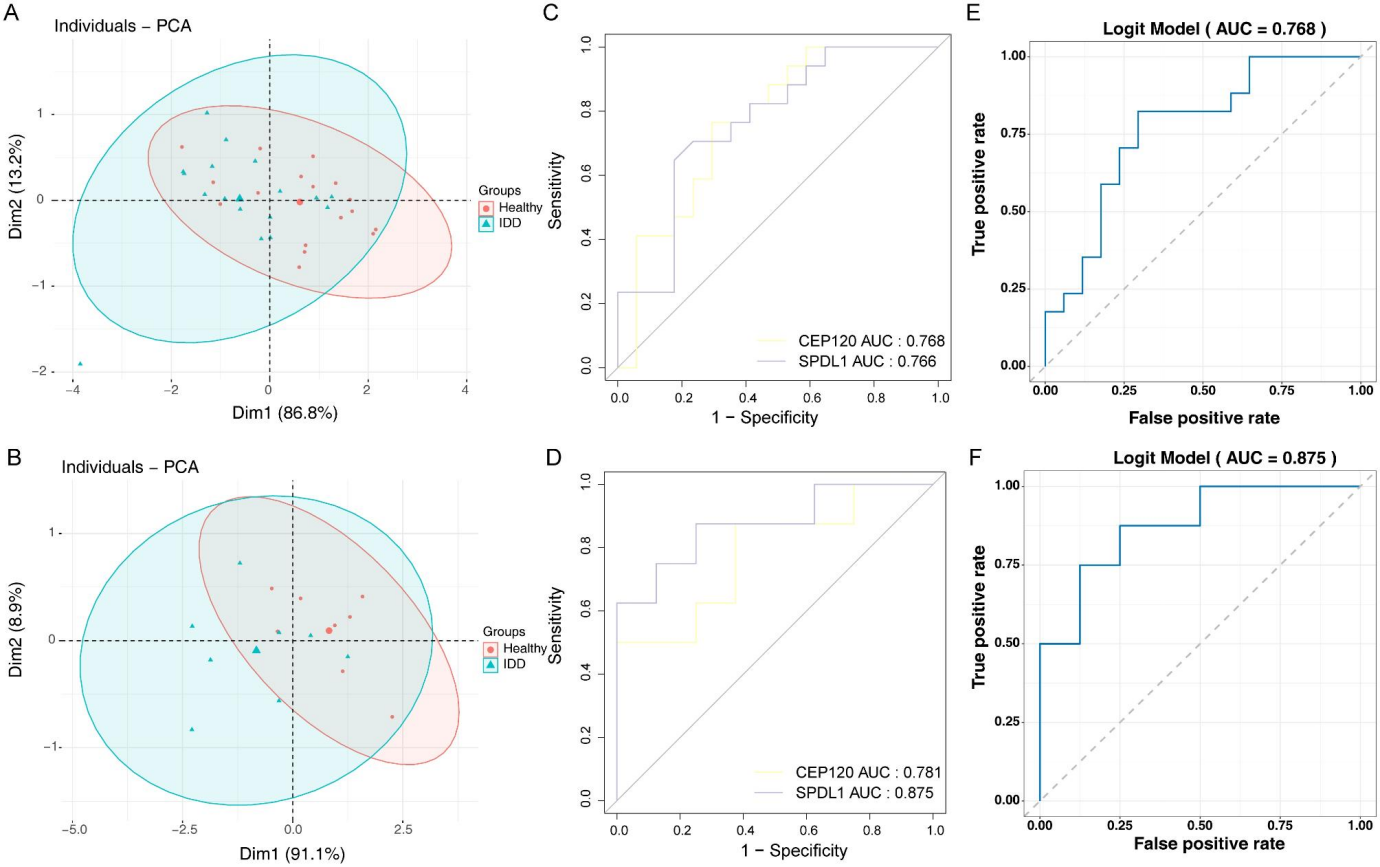
The diagnostic accuracy of the diagnostic genes. (**A**) PCA results of GSE150408. (**B**) PCA results of GSE124272. (**C**) ROC curves for diagnostic genes of GSE150408. (**D**) ROC curves for diagnostic genes of GSE124272. (**E**) Logistic regression ROC curve of GSE150408. (**F**) Logistic regression ROC curve of GSE124272. PCA, principal component analysis; ROC, receiver operating characteristic

The PCA results for both the GSE150408 and GSE124272 datasets showed that these two diagnostic genes, when considered together, could effectively distinguish IDD samples from healthy controls (Fig. 3A B).

Furthermore, the ROC curve for each diagnostic gene in both datasets showed AUC values greater than 0.75, which illustrated the diagnostic efficacy of each gene (Fig. 3C D). Simultaneously, the ROC curves for the combination of *CEP120* and *SPDL1* also had AUC values greater than 0.75 in both datasets, which also suggests the overall diagnostic effectiveness of the diagnostic genes was similar to their individual effects, confirming their substantial diagnostic power (Fig. 3E F).

### ssGSEA

**Fig. 4:**
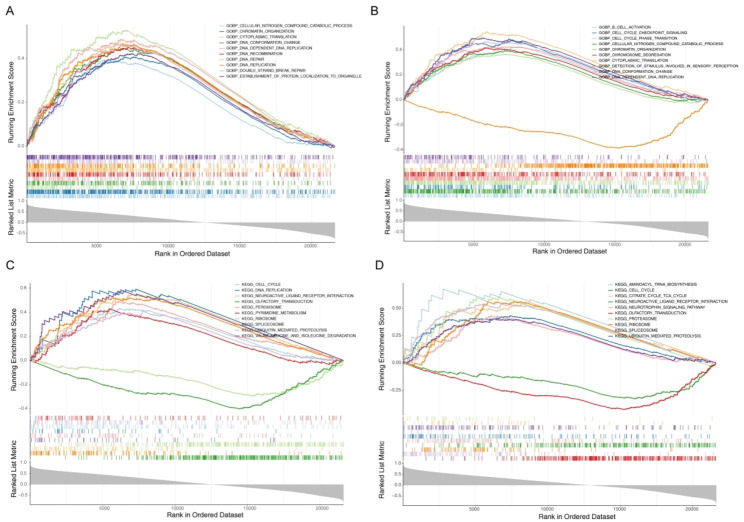
Single-sample GSEA analysis. (**A**) The top 10 results for GO enrichment of *CEP120*. The vertical coordinates represent the ES: positive ES indicates that the functional gene set is enriched in the front of the sequence; negative ES indicates that the functional gene set is enriched at the back of the sequence. The horizontal coordinates represent genes, and each small vertical line represents a gene. Overall, all genes in the top 10 are up-regulated. (**B**) The top 10 results for GO enrichment of *SPDL1*. Overall, 9 genes are up-regulated, and 1 gene is down-regulated. (**C**) The top 10 results for KEGG enrichment of *CEP120*. Overall, 8 genes are up-regulated and 2 are down-regulated. (**D**) The top 10 results for KEGG enrichment of *SPDL1*. Overall, 8 genes are up-regulated and 2 are down-regulated. ES, enrichment score; GO, Gene Ontology; GSEA, gene set enrichment analysis; KEGG, Kyoto Encyclopedia of Genes and Genomes

The top 10 enriched GO terms and KEGG pathways of *CEP120* and *SPDL1* are displayed in Fig. 4A D. Both diagnostic genes were found to participate in significant biological processes, including cellular nitrogen compound catabolic processes, corporation organization, cytoplasmic translation, DNA conformation change, and DNA-dependent DNA replication. Additionally, common KEGG pathways of both diagnostic genes included cell cycle, neuroactive ligand-receptor interaction, olfactory transduction, peroxisome, ribosome, spliceosome, and ubiquitin-mediated proteolysis.

### Immune infiltration between IDD and control samples

**Fig. 5:**
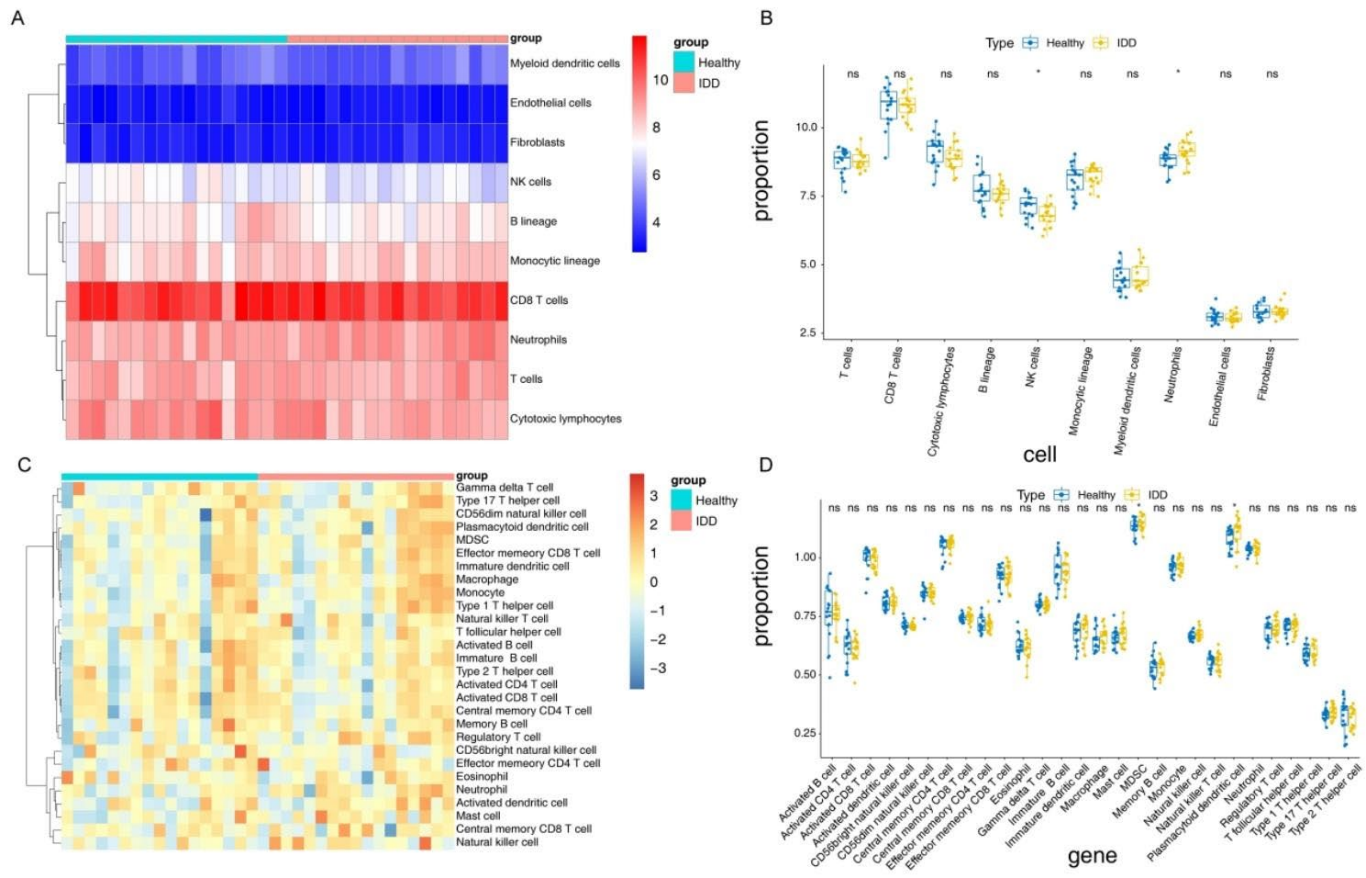
Immune-related analysis of diagnostic genes. (**A**) Heat map of different cell contents derived by MCP-counter analysis. The first row indicates the sample grouping, with blue for healthy samples and red for disease samples. Each row indicates the quantity of each cell in different samples, while each column indicates the total cell counts in each sample. The tree on the left side represents the results of cluster analysis, grouping different cell types from various samples. (**B**) Box plot of cell content between groups as derived by MCP-counter analysis. (**C**) Heat map of the different cell contents derived from ssGSEA analysis. The first row indicates the sample grouping, with blue for healthy samples and red for disease samples. Each row indicates the quantity of each of the 28 cell types in different samples, while each column indicates the cell counts in each sample. The tree on the left side represents the results of the cluster analysis of different cell types from different samples. (**D**) Box plot of cell content between groups as derived by ssGSEA analysis

The MCP-counter algorithm results of the GSE150408 dataset for both IDD and control samples showed that CD8 T cells were most abundant, while fibroblasts and epithelial cells were the least prevalent (Fig. 5A). Among the eight cell types analyzed, the proportion of natural killer cell was significantly lower in IDD samples, while neutrophils represented a larger percentage in IDD samples (Fig. 5B).

In addition, it can be seen from the ssGSEA results that the proportions of myeloid-derived suppressor cells (MDSCs) and neutrophils varied between IDD and healthy samples, with both cell types being more abundant in IDD samples (Fig. 5C-D).

### The miRNA-mRNA-TF and ceRNA regulatory networks and drug prediction

**Fig. 6:**
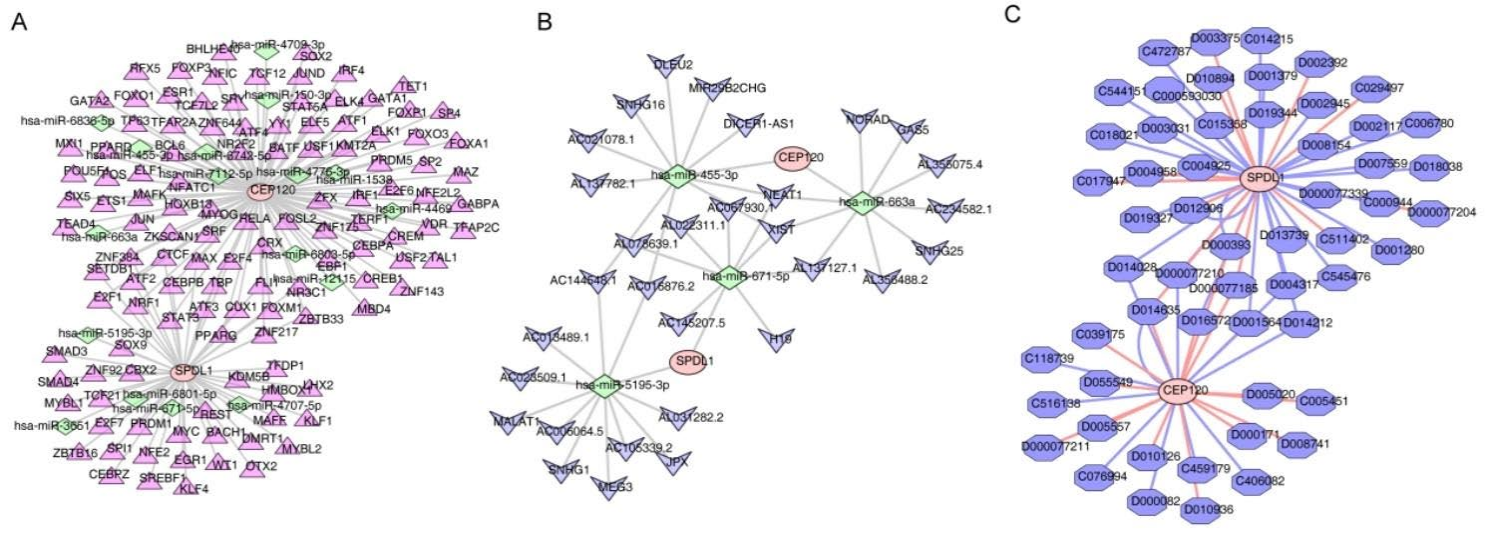
Construction of diagnostic genes-related regulatory networks. (**A**) The miRNA-mRNA-TF network diagram. The red circles indicate mRNAs; the pink triangles indicate TFs; the green diamonds indicate miRNAs. (**B**) The mRNA-miRNA-lncRNA network diagram. The red circles indicate mRNAs; the blue arrow shapes indicate lncRNAs; the green diamond indicates miRNAs. (**C**) Drug-diagnostic gene network diagram. The red circles indicate mRNAs; the blue hexagons indicate drugs

The miRNA prediction result from the miRwalk website indicated that *CEP120* and *SPDL1* are predicted to target 17 miRNAs. Further, 120 TFs were predicted to interact with these two diagnostic genes. Consequently, a complex miRNA-mRNA-TF regulatory network was constructed, consisting of 139 nodes (comprising the two diagnostic genes, 17 miRNAs, and 120 TFs) and 156 edges (Fig. 6A). For instance, miRNAs such as *hsa-miR-5195-3p*, *hsa-miR-6801-5p*, *hsa-miR-671-5p*, *hsa-miR-4707-5p*, *hsa-miR-3651*, along with TFs including FOXM1, PPARG, ATF3, ZNF217, and ZBTB33, were identified as potential regulators of *SPDL1*. On the other hands, the transcription of *CEP120* was regulated by *hsa-miR-150-3p*, *hsa-miR-4709-3p*, *hsa-miR-455-3p*, *hsa-miR-6836-5p*, *hsa-miR-6742-5p*, *hsa-miR-4776-3p*, *hsa-miR-1538*, *hsa-miR-7112-5p*, *hsa-miR-12,115*, *hsa-miR-663a*, *hsa-miR-4469*, *hsa-miR-6803-5p*, along with TFs including GATA2, ELF1, PPARD, GATA1, ELK4, SP4, and TET1.

Besides, based on the 17 target miRNAs of the two diagnostic genes, 31 lncRNAs were predicted to interact. However, these lncRNAs were only predicted by four miRNAs (*hsa-miR-455-3p*, *hsa-miR-663a*, *hsa-miR-671-5p*, and *hsa-miR-5195-3p*). Therefore, a ceRNA network was constructed consisting of 37 nodes (31 lncRNAs, 4 miRNAs, and the 2 diagnostic genes) and 42 edges. For example, *AC144548.1* was identified as a regulator of *SPDL1* and *CEP120* through *hsa-miR-5195-3p* and *hsa-miR-455-3p*, respectively (Fig. 6B).

Finally, a total of 56 drugs were predicted to be associated with the two diagnostic genes. D014028, D000077210, D000077185, D004317, D001564, and D014212 were identified as common drugs for both diagnostic genes (Fig. 6C). These findings provide insights into potential therapeutic interventions and the regulatory mechanisms of these diagnostic genes in the context of the disease.

### Validation of two diagnostic genes

To confirm the reliability of the two diagnostic genes obtained, the expressions of *CEP120* and *SPDL1* were verified by qRT-PCR. It can be seen from the results that *CEP120* expressions were lower in IDD samples compared to controls (*p <* 0.0058); similarly, *SPDL1* expression was down-regulated in IDD samples (*p <* 0.0073) (Additional file 7, Fig. 7). These findings provide experimental validation of the gene expression differences observed between healthy and IDD samples, supporting the diagnostic significance of *CEP120* and *SPDL1*.

**Fig. 7:**
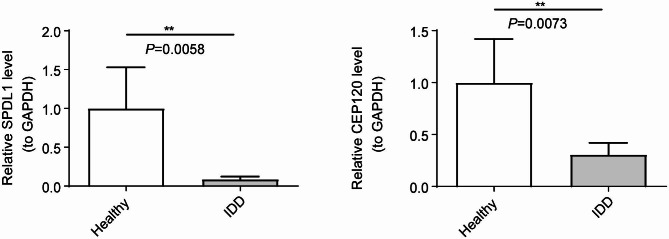
Comparison of differences in expression of diagnostic genes between healthy (n = 12) and IDD (n = 12) samples by qRT-PCR (*p <* 0.01)

## Discussion

The intervertebral disc, a complex cartilaginous tissue, connects adjacent vertebral bodies and maintains the mechanical load that allows the spine to move. In healthy intervertebral discs, homeostasis between assimilation and catabolic processes maintains the state of the ECM. Unfortunately, aging and constant mechanical stress can disrupt the metabolic environment of disc cells. This leads to the dysregulation of various factors in the surrounding environment, potentially resulting in the breakdown of macromolecules and the subsequent development of IDD [30].

Current treatments for IDD are either surgical interventions or the relief and management of individual symptoms. Nevertheless, various innovative approaches have been developed, including therapeutic protein or stem cell injections, gene therapy, molecular therapy, and tissue engineering. These approaches have shown promising results in animal models and hold promise for clinical applications [31, 32]. To expedite their clinical application, it is necessary to conduct in-depth research into the underlying mechanisms of IDD, examining key biomarkers, signaling pathways, and immune infiltration. In the study, through various bioinformatics analyses, we preliminarily identified two diagnostic genes and the associated regulatory networks, along with potential drugs that could be relevant to the progression of IDD.

The integration of bioinformatics has significantly enriched our study. We performed differential analysis on two datasets, GSE150408 and GSE124272, sourced from the GEO database, to obtain 336 DEGs, 230 up-regulated and 106 down-regulated. In particular, we focused on two promising diagnostic candidate genes, *CEP120* and *SPDL1*, which were selected through the RF model with a fold of 3. These genes were evaluated in the GSE150408 dataset and validated in the GSE124272 dataset.

CEP120 is a sub-centromere enrichment protein involved in centromere elongation, including centromere replication, assembly, elongation, and maturation [33–38]. During the cell cycle, CEP120 levels gradually increase from the early S phase to the M phase and decrease significantly at the end of mitosis. Its overexpression not only leads to centriole overgrowth but also generates atypical redundant centrioles [36]. On the other hand, the lack of CEP120 results in delayed or stalled cell cycles in vivo. It is worth noting that disc fibrosis has been associated with telomere shortening and DNA damage response (DDR) [39–41]. In patients with IDD, the ends of DNA strands are incompletely replicated during continuous cellular replication, causing gradual shortening, decreased telomerase activity, and activation of aging-related pathways [42]. In this context, intracellular DDR is activated in response to changes in telomere length, suggesting a potential correlation between the DDR process and down-regulation of CEP120 [43]. In addition, the activation of cellular senescence in NP cells can arrest the cell cycle and elevate reactive oxygen species (ROS) concentrations. DDR triggered by oxidative activation further induces senescence, degeneration, and fibrosis of intervertebral disc cells [44]. Additionally, various inflammatory factors such as TNF-α, IL-1α, IL-1β, IL-6, IL-17, and various chemokines can enhance the catabolism of disc ECM and promote the inflammatory response, exacerbating the disease [45–48].

SPDL1 is a functional glycosylated protein that has an inhibitory effect on activated T cells [49, 50].It is generated through selective splicing and translation of PD-L1 precursor mRNA [51–53]. SPDL1 is also an active circulating protein that induces apoptosis of CD8^+^ T cells and impairs the ability of these effector cells to kill tumor cells [54, 55]. Patients with high levels of SPDL1 tend to benefit more from anti-PD-L1 treatment. However, it is noteworthy that *SPDL1* expression does not always correlate with immunosensitivity, especially in lung cancer [56, 57]. Whether PD-L1 positively or negatively regulates *SPDL1* remains controversial. Besides, the SPDL1 level is elevated in many inflammatory diseases and is thus considered to be an inflammatory marker reflecting the widespread expression of mPD-L1 in an inflammatory environment. Based on the findings of our current research, we believe that *CEP120* can slow down mitosis and bring tissue development to a near-standstill, resulting in qualitative changes such as cell apoptosis. Meanwhile, the high *SPDL1* expression could alter the immune microenvironment, inhibiting immune responses and making the disc tissue less resistant to external stimuli or tolerant thereof. This cascade of events eventually leads to fibrosis, which may serve to stabilize the disc. However, the association between IDD and *CEP120* as well as *SPDL1* is relatively unexplored in existing literature. Therefore, our findings may indeed provide a new avenue for the diagnosis and treatment of IDD.

For the immune microenvironment of patients with IDD, the MCP-counter algorithm was used to quantify immune cells, fibroblasts, and epithelial cells in the GSE150408 dataset. This analysis revealed that the content of natural killer cells and neutrophils differed significantly between groups. The observed increase in neutrophils aligns with previous literature, where stimulation of IL-1β led to activated disc cells and the secretion of neutrophil-initiated cytokines, which are associated with inflammatory response in IDD [58]. This is consistent with the involvement of neutrophils in the cellular NLRP3/caspase-1 pathway and IL-1β secretion, as reported in earlier studies [59]. Simultaneously, NP cell damage caused by disc degeneration leads to a massive proliferation of natural killer cells, which induces hypersensitivity reactions [60, 61]. Furthermore, the ssGSEA results show higher infiltration of MDSCs and neutrophils in the IDD samples. Persistent inflammation during tissue aging is associated with a compensatory anti-inflammatory response that prevents excessive tissue damage. This response includes the activation of an associated immunosuppressive network that involves an increase in the number of MDSCs, regulatory T cells, and macrophages [62]. MDSCs, in particular, have been implicated in the development of cellular fibrosis by producing immunomodulatory mediators that stimulate the disposition of fibroblasts and stromal proteins a process aimed at limiting harmful inflammation [63].

The ssGSEA enrichment results for the two diagnostic genes repeatedly highlighted specific biological processes and signaling pathways. These include DNA conformation change, DNA-dependent DNA replication, as well as the KEGG singling pathways like the spliceosome, ubiquitin-mediated proteolysis, and cell cycle. These repeated enrichments indicate a strong relationship between these diagnostic genes and IDD progression, particularly in processes related to cell cycle, DNA damage, repair, replication, and degradation. Furthermore, upon literature review, we found that several molecular biomarkers are widely used to detect cellular senescence and degeneration. These include oncogene p53, cell cycle kinase-dependent (CDK) inhibitors p16 and p21, cell cycle regulators (retinoblastoma protein Rb), p38, and telomere length [64]. There is evidence that the central signaling pathways mediating disc cell fibrosis are the p53-p21-retinoblastoma protein (Rb) pathway and the p16-Rb pathway, but the exact role of each signaling molecule has not been fully elucidated [65].

In the p53-p21-Rb pathway, p53 plays an irreplaceable role in inducing cell senescence by responding to telomerase shortening and DDR (i.e., it initiates the irreversible process that brings the cell cycle to a standstill) [64]. At the site of DNA damage, following the direct phosphorylation of p53, p21 (a crucial downstream regulator of p53 signaling) inhibits CDK2 activity. This, in turn, prevents Rb phosphorylation, thus blocking the activation of E2F factors and delaying the cell cycle transition from the G1 to S phase ultimately leading to cell cycle arrest [66, 67]. This mechanism may be one of the potential reasons why *CEP120* expression is not elevated in this context. Notably, in NP and AF specimens of IDD, the expression of *p53*, *p21*, and *Rb* genes were significantly up-regulated, while telomere shortening and decreased telomerase activity could be detected [68, 69], confirming the oxidative activation of this pathway in NP cells.

In the p16-Rb signaling pathways, oxidative activation of p16 can inhibit the expression of *CDK4* and *CDK6*, thereby inhibiting the phosphorylation of Rb and delaying or halting cell cycle progression [69, 70]. The number of p16-positive cells in intervertebral disc tissue was positively correlated with Pfirrmann grading as well [68]. This correlation is attributed to that the p16-Rb pathway mediates mitochondrial damage with high glucose concentration, exacerbating the production of ROS in the intervertebral disc [71].

Given that the level of mRNA is regulated by both miRNAs and TFs, we predicted 17 target miRNAs and 120 TFs relevant to the two diagnostic genes. This prediction allowed us to construct a miRNA-mRNA-TF regulatory network. According to the existing studies, we found that p53 can regulate the expression of *hsa-miR-663a*, which in turn has a positive effect on cell proliferation, colony formation, and targeted invasion during the cell cycle, with E2F2 as the key TF target [72]. By acting on CCND1 and CDC34, *miR-671-5p* not only inhibits cell proliferation, cell cycle progression, as well as cloning in vitro and in vivo, but also promotes cell apoptosis [73]. Overexpression of *hsa-miR-5195-3p*, a potential regulator of the TGFβ signaling pathway, can significantly down-regulate c-MYC and cyclin D1, concomitantly affecting p21 levels by increasing its expression [74]. Besides, the deletion of p16 suppresses the down-regulation of TF E2F1/2 levels and regulates oxidative stress, thereby slowing down disc degeneration [75]. According to the results of the miRNA-mRNA-TF regulatory network, *CEP120* was considered to be involved in this process as well.

It was also found that NRF1 induces autophagy and inhibits apoptotic responses by promoting *Atg7* expression in compressed NP cells, delaying disc degeneration [76]. Phosphorylation of STAT3 accelerates NP apoptosis and IDD by degrading ECM [77]. Our forecast results for TF also confirm this view.

The prediction of potential drugs for *CEP120* and *SPDL1*, as target genes, revealed 56 potential drugs or molecular compounds. After review, we found that D014028 (tobacco smoke pollution) and D001564 (benzo(a)pyrene), both components of cigarette smoking, accelerated the degradation of collagen and proteoglycan in rat intervertebral discs thereby causing apoptosis of NP cells and eventually IDD. The therapeutic effect of D000077185 (resveratrol) on IDD is related to its antioxidant and anti-inflammatory activity, while resveratrol also affects NP cell apoptosis, autophagy, and ECM biosynthesis through various signaling pathways [78–80]. However, the specific mechanism of action of the two genes in IDD requires further experimental studies. These findings suggest that once the specific mechanisms of *CEP120* and *SPDL1* in IDD are elucidated, these drugs and molecular compounds could offer new therapeutic avenues for the treatment of IDD.

In summary, this study has elucidated the diagnostic genes-related biological processes and pathways associated with the development of IDD through comprehensive bioinformatics analysis. These findings contribute to our understanding of the etiology of IDD and shed light on potential diagnostic genes (i.e., *CEP120* and *SPDL1*) and therapeutic agents. Notably, the study predicted various therapeutic agents, including tobacco smoke pollution, benzo(a)pyrene, and resveratrol, which may play roles in the development and progression of IDD. However, research on these drugs is currently in the animal stage, and further clinical studies are needed to investigate their effects in patients with IDD.

Our study is not without limitations. First, all results were derived by bioinformatics algorithms without raw data from clinical samples to assess the quality of sequencing samples, such as Pfirrmann grading. Second, fibrosis-related genes in IDD still need to be explored in depth by various in vivo or in vitro experiments. We subsequently need more studies to validate and reveal further mechanisms, which could lead to the discovery of new potential therapeutic targets for IDD.

## Conclusion

In summary, *CEP120* and *SPDL1* were identified as key fibrosis-related diagnostic genes for patients with IDD. Additionally, a potential regulatory network and therapeutic agents targeting these two key genes were preliminarily predicted. These findings provide a reference for further study of IDD in fibrosis-related genes.

### Electronic supplementary material

Below is the link to the electronic supplementary material.

Supplementary Material 1

Supplementary Material 2

Supplementary Material 3

## Data Availability

The data presented in this study are openly available in the GEO database (https://www.ncbi.nlm.nih.gov/geo/), reference number [GSE150408 and GSE124272].

## Abbreviations

IDD: Intervertebral disc degeneration
LBP: Low back pain
AF: Annulus fibrosus
NP: Nucleus pulposus
ECM: Extracellular matrix
FN: Fibronectin
MF: Myofibroblasts
MMPs: Matrix metalloproteinases
DEGs: Differentially expressed genes
FIGs: Fibrosis-associated genes
DE-FIGs: Differentially expressed FIGs
RF: Random forest
ROC: Receiver operating characteristic
GSEA: Gene set enrichment analysis
TFs: Transcription factors
qRT-PCR: Quantitative real-time polymerase chain reaction
LR: Logistic Regression
GBDT: Gradient boosting Decision Tree
SVM: Support Vector Machine
ANN: Artificial Neural Network
DT: Decision Tree
MNB: MultinomialNB
AUC: The area under the ROC curve
DCA: Decision curve analysis
ssGSEA: Single-sample gene set enrichment analysis
ceRNA: Competing endogenous RNA
CTD: Comparative Toxicogenomics Database
