# Supplementary material for: Machine learning algorithm predicts fibrosis-related blood diagnosis markers of intervertebral disc degeneration
Source: BMC Med Genomics. 2023 Nov 1;16:274. doi: 10.1186/s12920-023-01705-6 (PMC10619283; doi:10.1186/s12920-023-01705-6)
Supplement: Supplementary file 3 — Supplementary Material 3 [file 12920_2023_1705_MOESM3_ESM.docx]

**Additional file**

**Additional file 1. Table S1.** Lists of 8621 fibrosis-related genes (FIGs) in GeneCards database.

See Additional file 6. Table S5.elsx

**Additional file 2. Table S2.** Lists of 2539 fibrosis-related genes (FIGs) screened by relevance score > 1.

See Additional file 7. Table S6.elsx

**Additional file 3. Table S3.** Lists of 4489 differentially expressed genes between healthy and IDD samples in GSE124272 dataset.

| Symble | logFC | AveExpr | t | P.Value | adj.P.Val | B |
| --- | --- | --- | --- | --- | --- | --- |
| KCNH4 | 1.752769705 | 1.920216742 | 6.304587585 | 1.26E-05 | 0.196110915 | 2.794909592 |
| VTRNA1-3 | -1.01451853 | 1.144161442 | -6.210867399 | 1.49E-05 | 0.196110915 | 2.669605609 |
| ZNF542P | -0.711068784 | 5.245451388 | -6.13910284 | 1.69E-05 | 0.196110915 | 2.572576612 |
| LOC101927136 | 0.807095519 | 2.267253431 | 5.794724993 | 3.20E-05 | 0.22403432 | 2.093926404 |
| LGALSL | 1.363692236 | 8.819182379 | 5.789862143 | 3.23E-05 | 0.22403432 | 2.087013359 |
| CBLB | -0.550769984 | 8.585886952 | -5.615225118 | 4.48E-05 | 0.25029242 | 1.835920679 |
| lnc-KRT80-3 | -1.264737777 | 1.876104484 | -5.552375342 | 5.04E-05 | 0.25029242 | 1.744215352 |
| lnc-DYNC1I1-2 | 1.710341357 | 2.362773119 | 5.359024726 | 7.30E-05 | 0.253687302 | 1.457697686 |
| lnc-SLC48A1-1 | 0.751800026 | 4.931301647 | 5.230189517 | 9.37E-05 | 0.253687302 | 1.263151042 |
| XLOC_l2_014720 | -0.489553858 | 10.52570898 | -5.198698114 | 9.96E-05 | 0.253687302 | 1.21516304 |
| lnc-C11orf46-2 | 0.769268467 | 1.469857182 | 5.145789403 | 0.00011037 | 0.253687302 | 1.134158436 |
| PMS2P1 | -0.465762324 | 8.657154638 | -4.977094794 | 0.000153655 | 0.253687302 | 0.872753224 |
| USP11 | -0.466756592 | 8.919235165 | -4.963707171 | 0.000157767 | 0.253687302 | 0.851807448 |
| KHSRP | -0.45049683 | 11.0462558 | -4.949752934 | 0.000162172 | 0.253687302 | 0.829944254 |
| SMYD3 | -0.645557429 | 6.533470537 | -4.934180737 | 0.000167239 | 0.253687302 | 0.805508959 |
| SPATA22 | 1.406628329 | 1.581696555 | 4.925474273 | 0.000170142 | 0.253687302 | 0.791830102 |
| lnc-ALDH1A3-1 | -1.109434165 | 1.975416083 | -4.919477083 | 0.000172172 | 0.253687302 | 0.782400752 |
| TMIGD3 | 1.858415865 | 2.984030111 | 4.911857541 | 0.000174788 | 0.253687302 | 0.77041228 |
| lnc-LY96-2 | 1.404621206 | 1.640381649 | 4.902628759 | 0.00017801 | 0.253687302 | 0.755879427 |
| INCENP | -0.574104349 | 5.431738594 | -4.868219037 | 0.000190574 | 0.253687302 | 0.701573961 |
| GNAS-AS1 | -1.214634107 | 2.246238693 | -4.86486192 | 0.000191848 | 0.253687302 | 0.696265718 |
| LEPROT | 0.384577388 | 7.877098356 | 4.832386266 | 0.000204631 | 0.253687302 | 0.644824037 |
| CEP250 | -0.523210806 | 7.799201206 | -4.79668125 | 0.000219699 | 0.253687302 | 0.588077075 |
| ACSBG1 | 1.080899847 | 2.861807899 | 4.789781151 | 0.00022274 | 0.253687302 | 0.577087804 |
| SYDE1 | 1.429892892 | 1.831314959 | 4.778900996 | 0.000227624 | 0.253687302 | 0.559744908 |
| HSPA6 | 1.121714694 | 9.874257071 | 4.776855807 | 0.000228554 | 0.253687302 | 0.556482861 |
| MTMR8 | -0.565257702 | 3.825031903 | -4.775496709 | 0.000229174 | 0.253687302 | 0.554314765 |
| LINC00528 | 0.570251007 | 3.541833441 | 4.773814565 | 0.000229944 | 0.253687302 | 0.551630937 |
| ADAM21 | 2.215294028 | 2.604959666 | 4.7615244 | 0.000235652 | 0.253687302 | 0.532009098 |
| RNF225 | 1.014459351 | 3.447130288 | 4.756412241 | 0.000238069 | 0.253687302 | 0.523840502 |
| SHMT2 | -0.579083825 | 9.393648084 | -4.755355754 | 0.000238572 | 0.253687302 | 0.522151871 |
| MSRB2 | 0.812523169 | 9.330536006 | 4.722744814 | 0.000254636 | 0.253687302 | 0.469945106 |
| FCGBP | -0.910131066 | 7.565877048 | -4.714263009 | 0.000258993 | 0.253687302 | 0.456340328 |
| lnc-RP11-511B23.3.1-3 | 1.360023418 | 1.755131586 | 4.68409402 | 0.000275122 | 0.253687302 | 0.407862258 |
| WTIP | -0.646948729 | 2.837658312 | -4.64254539 | 0.000299038 | 0.253687302 | 0.340878049 |
| SLC22A15 | 0.988134503 | 8.091082334 | 4.638230784 | 0.00030164 | 0.253687302 | 0.333907606 |
| CAPN5 | -0.66341783 | 6.489310058 | -4.637653447 | 0.00030199 | 0.253687302 | 0.332974687 |
| DHODH | -0.429325322 | 6.653269424 | -4.637357002 | 0.00030217 | 0.253687302 | 0.332495642 |
| C18orf54 | -0.958774029 | 3.254387061 | -4.585690234 | 0.000335264 | 0.253687302 | 0.248810308 |
| ANXA6 | -0.58726792 | 9.030429138 | -4.582516481 | 0.000337414 | 0.253687302 | 0.243657286 |
| lnc-TRPV6-2 | -0.689158513 | 6.633569804 | -4.575998915 | 0.000341874 | 0.253687302 | 0.233070648 |
| SMARCD1 | -0.300144562 | 8.533484455 | -4.573775491 | 0.000343409 | 0.253687302 | 0.229457714 |
| LRRC8A | -0.412178942 | 9.990386995 | -4.566662541 | 0.000348368 | 0.253687302 | 0.217894894 |
| LOC101930611 | -1.13186848 | 4.839339835 | -4.56150719 | 0.000352008 | 0.253687302 | 0.209509895 |
| FYN | -0.548238409 | 11.41569145 | -4.559508812 | 0.00035343 | 0.253687302 | 0.206258597 |
| lnc-MYBBP1A-2 | -0.536105224 | 7.624629352 | -4.559446308 | 0.000353474 | 0.253687302 | 0.206156897 |
| NTRK2 | 1.055455273 | 1.402918651 | 4.559296036 | 0.000353581 | 0.253687302 | 0.205912386 |
| VEGFB | -0.435279602 | 10.43305839 | -4.54699115 | 0.000362468 | 0.253687302 | 0.185880039 |
| lnc-GPR182-1 | 1.378701302 | 1.809850512 | 4.52614005 | 0.000378053 | 0.253687302 | 0.151886315 |
| CD300LF | 0.689344586 | 8.190456068 | 4.521800523 | 0.000381381 | 0.253687302 | 0.144803982 |
| RAB31 | 0.599429253 | 10.10359558 | 4.515350804 | 0.000386384 | 0.253687302 | 0.134272916 |
| HTR1B | 1.630681707 | 3.400809255 | 4.507278139 | 0.000392741 | 0.253687302 | 0.121083867 |
| DUSP26 | 0.938139822 | 1.192852639 | 4.478278835 | 0.000416472 | 0.253687302 | 0.07363184 |
| lnc-FOXD4L3-4 | 1.208481652 | 4.055632612 | 4.473428932 | 0.000420581 | 0.253687302 | 0.065684768 |
| UCHL1 | -2.331049551 | 1.97975603 | -4.458161077 | 0.000433792 | 0.253687302 | 0.040646212 |
| RAP2C | 0.419498695 | 6.386457833 | 4.447182071 | 0.000443553 | 0.253687302 | 0.022621968 |
| ITSN1 | 0.619678062 | 3.933801371 | 4.445736986 | 0.000444855 | 0.253687302 | 0.020248381 |
| BACH1 | 0.498567377 | 10.40378329 | 4.433602734 | 0.000455938 | 0.253687302 | 0.000306706 |
| PCTP | 0.508471506 | 8.934448908 | 4.433207075 | 0.000456304 | 0.253687302 | -0.000343854 |
| FZD3 | -1.030792906 | 3.464860567 | -4.422243785 | 0.000466568 | 0.253687302 | -0.018378338 |
| GPR125 | -1.19611318 | 4.583395194 | -4.409037889 | 0.000479246 | 0.253687302 | -0.040122683 |
| CXXC5 | -0.626593575 | 9.767009285 | -4.405361416 | 0.000482838 | 0.253687302 | -0.046180253 |
| SAE1 | -0.501323005 | 9.476611563 | -4.404192685 | 0.000483985 | 0.253687302 | -0.048106287 |
| lnc-JMJD7-PLA2G4B-1 | -1.389476045 | 7.798974966 | -4.397482524 | 0.000490628 | 0.253687302 | -0.059167839 |
| lnc-HS1BP3-1 | 0.542565608 | 4.192833797 | 4.384433007 | 0.000503814 | 0.253687302 | -0.080696222 |
| FAM159A | -0.553323448 | 8.50854585 | -4.382478351 | 0.00050582 | 0.253687302 | -0.083922779 |
| HEBP2 | 0.544679955 | 10.69354133 | 4.378581618 | 0.000509844 | 0.253687302 | -0.090356581 |
| ALG1L | -0.425585271 | 5.264133774 | -4.376713962 | 0.000511784 | 0.253687302 | -0.093440905 |
| HCG9 | 1.23621958 | 2.903809619 | 4.373567668 | 0.000515069 | 0.253687302 | -0.098637828 |
| NUF2 | -1.247247698 | 3.016607493 | -4.349095601 | 0.000541369 | 0.253687302 | -0.139102361 |
| NCF1B | 1.000633012 | 9.846608623 | 4.345309319 | 0.000545558 | 0.253687302 | -0.145369665 |
| ZNF850 | -0.506316961 | 4.608769447 | -4.344739226 | 0.000546192 | 0.253687302 | -0.146313476 |
| URB1 | -0.786087438 | 4.482663942 | -4.34033078 | 0.000551118 | 0.253687302 | -0.153613182 |
| ZC3H7B | -0.606035771 | 7.086606121 | -4.33421297 | 0.000558029 | 0.253687302 | -0.163747316 |
| LOC285181 | 1.612298282 | 2.691560408 | 4.32819155 | 0.000564917 | 0.253687302 | -0.173726284 |
| S100Z | 0.603283931 | 7.924009316 | 4.30865178 | 0.000587874 | 0.253687302 | -0.206139033 |
| IFNGR2 | 0.645963045 | 8.00061168 | 4.297137242 | 0.000601845 | 0.253687302 | -0.225261141 |
| P2RY13 | 0.915980883 | 8.557550179 | 4.294429591 | 0.000605179 | 0.253687302 | -0.229760033 |
| OSCAR | 0.632229706 | 10.31380919 | 4.28971596 | 0.000611028 | 0.253687302 | -0.237594053 |
| B3GALT6 | -0.490410017 | 8.630777309 | -4.287443196 | 0.000613869 | 0.253687302 | -0.241372319 |
| AUNIP | -1.107257357 | 2.120985983 | -4.281156494 | 0.000621798 | 0.253687302 | -0.251826599 |
| B3GNT5 | 1.008206396 | 6.225617007 | 4.275450839 | 0.000629084 | 0.253687302 | -0.261318706 |
| APBA2 | -0.575401763 | 6.809241413 | -4.258804744 | 0.000650843 | 0.253687302 | -0.289033588 |
| HMOX2 | -0.415701971 | 9.906449487 | -4.257262741 | 0.000652897 | 0.253687302 | -0.291602585 |
| TP53I3 | 0.481775185 | 7.042922633 | 4.237836091 | 0.000679347 | 0.253687302 | -0.323991251 |
| ZFP90 | -0.635370451 | 8.724126536 | -4.236640749 | 0.000681009 | 0.253687302 | -0.325985581 |
| LTBR | 0.864539138 | 6.766349581 | 4.23247058 | 0.000686842 | 0.253687302 | -0.332944443 |
| LINC00618 | -0.444857408 | 6.46150716 | -4.224769549 | 0.000697748 | 0.253687302 | -0.345800557 |
| FOXK2 | -0.355432104 | 7.143439868 | -4.22215477 | 0.000701491 | 0.253687302 | -0.350167205 |
| FBXO32 | -0.529426859 | 7.467239675 | -4.222132622 | 0.000701523 | 0.253687302 | -0.350204196 |
| LOC101930010 | 0.797676498 | 2.814439475 | 4.21006041 | 0.000719072 | 0.253687302 | -0.370374686 |
| FXYD1 | 1.221469739 | 2.03966916 | 4.205042427 | 0.000726497 | 0.253687302 | -0.378763647 |
| TMIGD2 | -0.827852734 | 9.137100559 | -4.204360309 | 0.000727512 | 0.253687302 | -0.379904214 |
| CYP27A1 | 1.072974192 | 9.995380089 | 4.202171831 | 0.00073078 | 0.253687302 | -0.383563915 |
| DENND2D | -0.714464388 | 11.88287562 | -4.193579479 | 0.000743753 | 0.253687302 | -0.397937691 |
| CASC18 | 0.904452455 | 1.47863928 | 4.18682299 | 0.000754119 | 0.253687302 | -0.409246065 |
| HK3 | 0.768308177 | 6.367116238 | 4.185185671 | 0.000756653 | 0.253687302 | -0.411987212 |
| GANAB | -0.401817219 | 9.015676972 | -4.180730799 | 0.000763592 | 0.253687302 | -0.4194469 |
| NINJ1 | 0.731022312 | 13.549437 | 4.16945492 | 0.000781445 | 0.253687302 | -0.438338044 |
| DACT3 | -0.803911629 | 2.250694967 | -4.167810517 | 0.000784084 | 0.253687302 | -0.441094162 |
| CCDC88C | -0.458863151 | 9.282063977 | -4.163507635 | 0.000791032 | 0.253687302 | -0.44830743 |
| lnc-BZRAP1-1 | 0.619586317 | 8.94276094 | 4.157549195 | 0.000800756 | 0.253687302 | -0.458299327 |
| HIST2H2BE | 1.090054616 | 3.707615759 | 4.152165019 | 0.000809648 | 0.253687302 | -0.46733149 |
| lnc-BRI3BP-2 | 0.870534364 | 1.24641363 | 4.144987445 | 0.000821659 | 0.253687302 | -0.479376944 |
| CAPZA2 | 0.518231727 | 7.634930789 | 4.142639533 | 0.000825627 | 0.253687302 | -0.483318409 |
| CCR9 | -2.360747562 | 5.509837881 | -4.134032925 | 0.000840339 | 0.253687302 | -0.497771377 |
| LOC100128607 | 1.701530327 | 3.478424745 | 4.132893848 | 0.000842306 | 0.253687302 | -0.499684796 |
| UBE2W | 0.380614661 | 8.657946211 | 4.131917907 | 0.000843995 | 0.253687302 | -0.501324288 |
| LINC01163 | -0.767834552 | 4.411707576 | -4.131210029 | 0.000845222 | 0.253687302 | -0.502513522 |
| REEP6 | -0.556286008 | 5.644936407 | -4.122815461 | 0.000859915 | 0.253687302 | -0.516620342 |
| ARHGEF9 | -0.791550106 | 4.912055571 | -4.122284225 | 0.000860853 | 0.253687302 | -0.517513313 |
| LOC100506191 | -1.101264462 | 1.299384039 | -4.119743224 | 0.000865357 | 0.253687302 | -0.521784968 |
| ALYREF | -0.425591623 | 9.134689353 | -4.112618427 | 0.000878112 | 0.253687302 | -0.533765948 |
| LOC100134368 | -0.995924155 | 1.485777391 | -4.11196757 | 0.000879287 | 0.253687302 | -0.534860682 |
| ACOT1 | -0.569422016 | 8.077051389 | -4.111836961 | 0.000879523 | 0.253687302 | -0.535080371 |
| LPAR5 | -0.662588084 | 5.271046276 | -4.104782468 | 0.000892361 | 0.253687302 | -0.546948811 |
| SH3D19 | -0.775298313 | 1.373258376 | -4.100635287 | 0.000899998 | 0.253687302 | -0.553928366 |
| XLOC_l2_014645 | -0.746767165 | 7.551449144 | -4.099628749 | 0.000901861 | 0.253687302 | -0.555622595 |
| SYP-AS1 | 1.152784528 | 3.728781372 | 4.098406188 | 0.000904129 | 0.253687302 | -0.557680577 |
| CLEC1A | 0.758429734 | 5.4254387 | 4.097477907 | 0.000905856 | 0.253687302 | -0.559243289 |
| BST1 | 0.637759295 | 8.239067876 | 4.097003209 | 0.00090674 | 0.253687302 | -0.56004245 |
| NADK | 0.753081987 | 9.41349461 | 4.092482056 | 0.000915204 | 0.253687302 | -0.567655021 |
| GBP6 | 1.300932876 | 1.932264639 | 4.089651878 | 0.000920543 | 0.253687302 | -0.572421426 |
| DBP | -0.567463742 | 6.708940872 | -4.084175058 | 0.000930966 | 0.253687302 | -0.581647409 |
| lnc-RABEP2-4 | -0.621836592 | 5.637509456 | -4.07373049 | 0.000951175 | 0.253687302 | -0.599250063 |
| SATB1-AS1 | -0.8007114 | 8.500259787 | -4.069852332 | 0.000958792 | 0.253687302 | -0.605788814 |
| NLRP7 | -2.531252895 | 3.445398182 | -4.066843688 | 0.000964744 | 0.253687302 | -0.610862541 |
| ZNF48 | -0.570359604 | 6.044934473 | -4.058783666 | 0.000980873 | 0.253687302 | -0.624459162 |
| NCAPD2 | -0.413495868 | 9.768492626 | -4.052494058 | 0.00099365 | 0.253687302 | -0.635073599 |
| LOC649294 | 1.410941993 | 2.080986422 | 4.043718933 | 0.001011758 | 0.253687302 | -0.649888963 |
| lnc-NRSN1-3 | 1.300212282 | 1.615558168 | 4.04089352 | 0.00101766 | 0.253687302 | -0.65466077 |
| SLC22A4 | 1.210801849 | 7.610203769 | 4.039527429 | 0.001020525 | 0.253687302 | -0.656968217 |
| C3AR1 | 0.749975149 | 8.125784443 | 4.038274687 | 0.001023161 | 0.253687302 | -0.659084362 |
| LOC100507437 | -1.0390914 | 1.878308669 | -4.037466408 | 0.001024865 | 0.253687302 | -0.660449795 |
| XBP1 | -0.789213986 | 11.89198498 | -4.036746917 | 0.001026384 | 0.253687302 | -0.661665288 |
| GPR160 | 0.713673975 | 7.711256529 | 4.035426852 | 0.001029177 | 0.253687302 | -0.663895506 |
| RFWD2 | 0.388253492 | 8.078084303 | 4.035404712 | 0.001029224 | 0.253687302 | -0.663932913 |
| SBF2 | 0.557929988 | 5.985031512 | 4.027498332 | 0.001046117 | 0.253687302 | -0.67729399 |
| KDELC2 | -0.834287922 | 6.025350613 | -4.026699138 | 0.00104784 | 0.253687302 | -0.678644881 |
| lnc-ATP6AP2-6 | 1.413608452 | 1.509324402 | 4.023587453 | 0.001054577 | 0.253687302 | -0.683905181 |
| IFFO2 | -0.562932854 | 9.435394716 | -4.022824555 | 0.001056235 | 0.253687302 | -0.685194996 |
| PITPNM2 | -0.511906173 | 4.789369276 | -4.020675106 | 0.001060921 | 0.253687302 | -0.688829313 |
| LOC101929696 | 1.782427606 | 2.548877217 | 4.016247403 | 0.001070642 | 0.253687302 | -0.696317077 |
| SETBP1 | -0.60051388 | 8.186281201 | -4.01501946 | 0.001073353 | 0.253687302 | -0.698393991 |
| MRM1 | -0.291231492 | 6.225467673 | -4.010707385 | 0.001082931 | 0.253687302 | -0.705688431 |
| BIVM | -0.58871014 | 5.343772265 | -4.009809083 | 0.001084937 | 0.253687302 | -0.70720824 |
| RNF135 | 0.35977641 | 9.728081498 | 4.006003586 | 0.001093478 | 0.253687302 | -0.713647457 |
| CALU | -0.405573506 | 6.833367278 | -4.000740603 | 0.001105401 | 0.253687302 | -0.722555022 |
| XPO5 | -0.362704094 | 5.35686375 | -3.999001515 | 0.00110937 | 0.253687302 | -0.725498967 |
| TMEM97 | -0.858606679 | 4.873916905 | -3.997961625 | 0.001111751 | 0.253687302 | -0.727259434 |
| CLEC5A | 1.232244106 | 2.326211458 | 3.987632046 | 0.001135676 | 0.253687302 | -0.744751986 |
| lnc-SHB-1 | 1.327135383 | 2.218199885 | 3.985103896 | 0.001141611 | 0.253687302 | -0.749034709 |
| FAS | 0.674564975 | 8.676484828 | 3.983830787 | 0.001144612 | 0.253687302 | -0.751191586 |
| ADA | -0.571010503 | 10.89762085 | -3.977588821 | 0.001159439 | 0.253687302 | -0.761768675 |
| lnc-NPAS2-1 | 1.054359981 | 4.507321156 | 3.975625137 | 0.001164144 | 0.253687302 | -0.765096868 |
| HPDL | -1.250401897 | 3.716095719 | -3.972634559 | 0.001171346 | 0.253687302 | -0.770166158 |
| HARS | -0.351271683 | 8.536271125 | -3.971830023 | 0.001173291 | 0.253687302 | -0.77153005 |
| NLRC4 | 0.609726693 | 8.96622628 | 3.965732845 | 0.00118814 | 0.253687302 | -0.781868125 |
| GZMM | -0.764253985 | 9.513112153 | -3.965349789 | 0.001189079 | 0.253687302 | -0.782517723 |
| MTBP | -1.28136109 | 2.487376479 | -3.958552039 | 0.001205872 | 0.253687302 | -0.794047643 |
| PARVG | 0.378385373 | 8.890371348 | 3.955852575 | 0.001212607 | 0.253687302 | -0.7986274 |
| SLC41A1 | -0.508677249 | 7.048893562 | -3.955437067 | 0.001213647 | 0.253687302 | -0.799332382 |
| TMEM154 | 0.517774627 | 9.439774665 | 3.955252985 | 0.001214108 | 0.253687302 | -0.799644713 |
| IL10RB | 0.62774509 | 8.438980276 | 3.948818403 | 0.001230336 | 0.253687302 | -0.810564071 |
| TMEM117 | -0.710232364 | 4.235420695 | -3.945920362 | 0.001237717 | 0.253687302 | -0.815483126 |
| BCL9 | -0.658898848 | 4.324714401 | -3.944512622 | 0.001241318 | 0.253687302 | -0.817872838 |
| EIF1AD | -0.245354483 | 9.081673084 | -3.940604104 | 0.001251372 | 0.253687302 | -0.824508619 |
| DLST | -0.425115599 | 8.578770682 | -3.936749299 | 0.001261369 | 0.253687302 | -0.831054448 |
| LOH12CR1 | -0.365268502 | 6.797750238 | -3.932368809 | 0.001272828 | 0.253687302 | -0.838494429 |
| STX3 | 0.886505674 | 6.21619841 | 3.926061258 | 0.001289512 | 0.253687302 | -0.84921016 |
| ELOVL6 | -0.913931485 | 4.225031912 | -3.925454185 | 0.001291129 | 0.253687302 | -0.85024167 |
| STMN1 | -0.766532298 | 8.963821414 | -3.924738452 | 0.001293039 | 0.253687302 | -0.851457849 |
| FARSB | -0.68719191 | 4.907801463 | -3.923194838 | 0.001297167 | 0.253687302 | -0.854080914 |
| TBC1D31 | -0.719527979 | 7.236192551 | -3.921561772 | 0.001301549 | 0.253687302 | -0.856856192 |
| TXLNA | -0.395523521 | 9.290594071 | -3.912451447 | 0.00132627 | 0.253687302 | -0.872342467 |
| CCDC127 | -0.333379975 | 7.163514395 | -3.912313692 | 0.001326647 | 0.253687302 | -0.872576683 |
| lnc-KRAS-1 | 0.996422991 | 2.316680791 | 3.911462689 | 0.001328981 | 0.253687302 | -0.874023616 |
| ZNF575 | -0.499858091 | 6.856371715 | -3.909578032 | 0.001334165 | 0.253687302 | -0.877228244 |
| SLC25A5-AS1 | -0.819006609 | 3.255572645 | -3.900231601 | 0.001360176 | 0.253687302 | -0.893124836 |
| lnc-AC091801.1.1-5 | -1.318147225 | 7.563676789 | -3.895506467 | 0.00137352 | 0.253687302 | -0.901164037 |
| RAB24 | 0.774417214 | 10.78771991 | 3.894146073 | 0.001377386 | 0.253687302 | -0.90347889 |
| LOC102724344 | 0.979090112 | 1.885809778 | 3.890077919 | 0.001389014 | 0.253687302 | -0.910402134 |
| ANXA3 | 1.606162164 | 8.011661959 | 3.889511249 | 0.001390642 | 0.253687302 | -0.911366603 |
| TTC26 | 0.79359984 | 1.100731547 | 3.885823656 | 0.00140128 | 0.253687302 | -0.917643459 |
| TNIK | -0.544322244 | 7.440739828 | -3.885667213 | 0.001401734 | 0.253687302 | -0.917909773 |
| RBBP4 | -0.453001786 | 10.36316945 | -3.884430559 | 0.001405321 | 0.253687302 | -0.920014998 |
| TMEM102 | -0.444547524 | 5.20471985 | -3.884133908 | 0.001406183 | 0.253687302 | -0.920520019 |
| TPP2 | -0.463714336 | 7.925951688 | -3.883871006 | 0.001406947 | 0.253687302 | -0.920967593 |
| C9orf47 | 0.777048601 | 6.52416806 | 3.883548507 | 0.001407885 | 0.253687302 | -0.921516635 |
| lnc-TMEM56-1 | 0.994095585 | 1.128632511 | 3.883266044 | 0.001408707 | 0.253687302 | -0.921997522 |
| ZNF773 | -0.523800569 | 6.511047297 | -3.87990829 | 0.001418518 | 0.253687302 | -0.927714493 |
| lnc-SBF2-1 | 1.429397831 | 3.617254556 | 3.87930138 | 0.001420299 | 0.253687302 | -0.928747919 |
| CCDC144NL-AS1 | 1.777807911 | 2.771596693 | 3.878330539 | 0.001423152 | 0.253687302 | -0.930401094 |
| FOXD2-AS1 | -1.24255664 | 1.969953684 | -3.876145545 | 0.001429594 | 0.253687302 | -0.934122019 |
| DLEU2L | 0.789283742 | 9.076944788 | 3.875524167 | 0.001431432 | 0.253687302 | -0.935180258 |
| GINS4 | -0.83913635 | 5.892713901 | -3.875435683 | 0.001431693 | 0.253687302 | -0.935330952 |
| DRAM1 | 0.51474242 | 7.9702305 | 3.859055824 | 0.00148101 | 0.255885338 | -0.963237113 |
| CD81 | -0.622247938 | 10.67116237 | -3.85519779 | 0.001492873 | 0.255885338 | -0.969812856 |
| C19orf48 | -0.483598765 | 7.665077607 | -3.855122309 | 0.001493106 | 0.255885338 | -0.969941519 |
| PLEKHA1 | -0.648009113 | 9.705902826 | -3.855093871 | 0.001493194 | 0.255885338 | -0.969989994 |
| NBPF12 | 0.553051975 | 7.103056335 | 3.849470655 | 0.001510661 | 0.255885338 | -0.979576329 |
| GK3P | 1.126887283 | 6.529035164 | 3.847565923 | 0.001516624 | 0.255885338 | -0.982823988 |
| GLG1 | -0.34695516 | 10.52905344 | -3.846276052 | 0.001520676 | 0.255885338 | -0.985023426 |
| LINC01539 | 0.604380358 | 4.491310483 | 3.845894486 | 0.001521877 | 0.255885338 | -0.98567408 |
| SH2D1A | -0.656379037 | 8.275078554 | -3.829433629 | 0.001574595 | 0.255885338 | -1.013753219 |
| CCDC147-AS1 | 1.137116005 | 4.674287409 | 3.828975639 | 0.001576088 | 0.255885338 | -1.014534733 |
| LCK | -0.563524118 | 10.34888445 | -3.826955313 | 0.001582691 | 0.255885338 | -1.01798239 |
| TRIM54 | 0.72871247 | 4.149520887 | 3.823765208 | 0.001593173 | 0.255885338 | -1.023426828 |
| BCL2L12 | -0.291339224 | 9.939844933 | -3.818353174 | 0.001611116 | 0.255885338 | -1.032664933 |
| HP | 1.416503449 | 8.682775363 | 3.810193585 | 0.001638556 | 0.255885338 | -1.046596716 |
| HERC2P7 | -0.951253994 | 5.478396942 | -3.804345054 | 0.001658513 | 0.255885338 | -1.056585287 |
| LRRC41 | -0.346869426 | 6.036200403 | -3.802094928 | 0.001666257 | 0.255885338 | -1.060428822 |
| MPZL2 | 0.682713827 | 7.237545615 | 3.800262538 | 0.001672589 | 0.255885338 | -1.063559049 |
| lnc-TRAPPC8-1 | -0.945036373 | 3.203322555 | -3.799355464 | 0.001675733 | 0.255885338 | -1.065108662 |
| KIAA1614 | 0.6368569 | 1.156303181 | 3.7992891 | 0.001675963 | 0.255885338 | -1.065222038 |
| XCL1 | -0.870413823 | 8.628267568 | -3.798644776 | 0.001678201 | 0.255885338 | -1.066322812 |
| MAZ | -0.591639168 | 5.288547901 | -3.796921606 | 0.001684198 | 0.255885338 | -1.069266841 |
| GPX7 | -0.722789137 | 4.611908375 | -3.794282703 | 0.001693425 | 0.255885338 | -1.073775764 |
| CUTA | -0.503301975 | 12.32676437 | -3.794176124 | 0.001693799 | 0.255885338 | -1.073957879 |
| JARID2-AS1 | 0.727414166 | 4.991708585 | 3.793913541 | 0.00169472 | 0.255885338 | -1.074406563 |
| lnc-MAGEB1-1 | 1.181059974 | 6.851934764 | 3.793324327 | 0.001696789 | 0.255885338 | -1.075413389 |
| METTL13 | -0.320796708 | 9.355926868 | -3.792501775 | 0.001699681 | 0.255885338 | -1.076818973 |
| SCARNA3 | 0.686810295 | 6.719174385 | 3.791237103 | 0.001704137 | 0.255885338 | -1.07898014 |
| ZNF557 | -0.530572075 | 7.081875846 | -3.788409827 | 0.001714143 | 0.255885338 | -1.083811966 |
| lnc-RNF144A-3 | -0.72773895 | 6.418243416 | -3.78686421 | 0.001719638 | 0.255885338 | -1.086453647 |
| CENPL | -1.084916529 | 2.657014194 | -3.786331524 | 0.001721536 | 0.255885338 | -1.087364119 |
| GNG10 | 0.697946271 | 11.21764289 | 3.786148801 | 0.001722187 | 0.255885338 | -1.087676434 |
| CLINT1 | -0.361062858 | 9.644530007 | -3.785451455 | 0.001724676 | 0.255885338 | -1.088868378 |
| lnc-TUSC5-2 | 0.800119733 | 6.801265341 | 3.782698349 | 0.001734536 | 0.255885338 | -1.093574448 |
| RHOT1 | 0.567029734 | 5.770854481 | 3.782603374 | 0.001734877 | 0.255885338 | -1.093736802 |
| ZNF543 | -0.702531332 | 4.678931946 | -3.781861651 | 0.001737544 | 0.255885338 | -1.095004765 |
| CYB5R4 | 0.563677854 | 8.392039647 | 3.78151228 | 0.001738802 | 0.255885338 | -1.09560202 |
| AAAS | -0.339423248 | 6.936414286 | -3.780474437 | 0.001742543 | 0.255885338 | -1.097376274 |
| MCM6 | -0.59783094 | 7.847665489 | -3.776800126 | 0.001755853 | 0.255885338 | -1.103658263 |
| CNN3 | -1.098391206 | 1.752023131 | -3.77514717 | 0.001761875 | 0.255885338 | -1.106484604 |
| LOC102724552 | 1.055201751 | 4.793029338 | 3.773216429 | 0.001768934 | 0.255885338 | -1.109786131 |
| RHOB | 0.623776438 | 7.498857955 | 3.765535466 | 0.001797302 | 0.255885338 | -1.122922669 |
| AUTS2 | -0.747573974 | 8.523279945 | -3.765400495 | 0.001797804 | 0.255885338 | -1.12315354 |
| FAM171A1 | -0.575087374 | 5.297795563 | -3.760426703 | 0.001816423 | 0.255885338 | -1.131662011 |
| DDIT3 | 0.490233439 | 5.339280118 | 3.756813066 | 0.001830072 | 0.255885338 | -1.137844643 |
| LINC01503 | 0.69767557 | 5.305507109 | 3.756625237 | 0.001830785 | 0.255885338 | -1.138166024 |
| lnc-INPP5F-1 | 0.938404346 | 1.678523807 | 3.755379765 | 0.001835515 | 0.255885338 | -1.140297113 |
| lnc-JMJD7-PLA2G4B-2 | -0.67751351 | 4.112300646 | -3.754903131 | 0.001837328 | 0.255885338 | -1.141112691 |
| GATC | -0.391268396 | 3.785995666 | -3.754785971 | 0.001837774 | 0.255885338 | -1.141313169 |
| lnc-MAGEB1-2 | 1.038083853 | 1.899780573 | 3.754522945 | 0.001838776 | 0.255885338 | -1.141763246 |
| CPEB4 | 0.543272491 | 8.328839394 | 3.754032765 | 0.001840644 | 0.255885338 | -1.142602027 |
| APOBEC3D | -0.736347018 | 4.701106207 | -3.753587939 | 0.001842341 | 0.255885338 | -1.143363214 |
| CEP63 | 0.446772546 | 8.284727014 | 3.75314161 | 0.001844045 | 0.255885338 | -1.144126983 |
| LOC286272 | -0.830566289 | 5.270252833 | -3.752551469 | 0.001846301 | 0.255885338 | -1.145136864 |
| ANKRD33B | 0.423183601 | 6.673477861 | 3.75249729 | 0.001846509 | 0.255885338 | -1.145229579 |
| MBLAC1 | -0.670617788 | 4.971114101 | -3.746350751 | 0.001870175 | 0.255885338 | -1.155749095 |
| MPI | -0.440314961 | 8.104669091 | -3.742851448 | 0.001883784 | 0.255885338 | -1.161738959 |
| HCG27 | 0.900456123 | 8.047475248 | 3.742526146 | 0.001885054 | 0.255885338 | -1.162295823 |
| COL20A1 | 0.839728516 | 3.0214362 | 3.741996251 | 0.001887125 | 0.255885338 | -1.163202931 |
| TERF2 | -0.388156734 | 5.852877169 | -3.737568457 | 0.00190452 | 0.255885338 | -1.170783332 |
| SMARCD3 | 0.645221965 | 9.385109749 | 3.737049383 | 0.00190657 | 0.255885338 | -1.171672061 |
| SMARCAL1 | -0.368531123 | 9.076963732 | -3.736240447 | 0.001909768 | 0.255885338 | -1.173057106 |
| POLR1A | -0.758724714 | 5.356129293 | -3.730419772 | 0.001932945 | 0.255885338 | -1.183024224 |
| PINK1 | 0.580798133 | 8.819978925 | 3.729881381 | 0.001935103 | 0.255885338 | -1.18394624 |
| PABPN1L | 1.301848259 | 1.831982501 | 3.726854301 | 0.001947281 | 0.255885338 | -1.189130535 |
| ZMYND11 | -0.519694399 | 6.178067558 | -3.726425218 | 0.001949014 | 0.255885338 | -1.189865439 |
| lnc-STARD10-1 | 1.099286872 | 5.857215476 | 3.725860428 | 0.001951296 | 0.255885338 | -1.190832789 |
| PTTG1IP | 0.38680977 | 10.68930783 | 3.72485254 | 0.001955377 | 0.255885338 | -1.192559102 |
| ID2 | -0.708610765 | 11.18708655 | -3.719698816 | 0.001976376 | 0.255885338 | -1.201387271 |
| TREML5P | 1.231555763 | 2.280810558 | 3.718351708 | 0.001981903 | 0.255885338 | -1.203695061 |
| SIDT1 | -0.486878567 | 7.919229205 | -3.718206603 | 0.001982499 | 0.255885338 | -1.203943652 |
| B4GAT1 | -0.594492817 | 4.440370789 | -3.717234895 | 0.001986496 | 0.255885338 | -1.205608396 |
| INTS9 | -0.343425788 | 9.159859223 | -3.715172835 | 0.001995005 | 0.255885338 | -1.20914131 |
| ZNF267 | 0.480299973 | 8.07364203 | 3.714193602 | 0.001999059 | 0.255885338 | -1.210819103 |
| CTSF | -0.578816095 | 8.558248739 | -3.714016369 | 0.001999793 | 0.255885338 | -1.211122775 |
| MIS18A | -0.538510624 | 6.850389 | -3.713231052 | 0.002003052 | 0.255885338 | -1.21246836 |
| MCM3 | -0.549219415 | 9.873752875 | -3.712729222 | 0.002005136 | 0.255885338 | -1.213328228 |
| KLF3-AS1 | -0.821789567 | 4.952621409 | -3.711219311 | 0.002011422 | 0.255885338 | -1.215915486 |
| FITM1 | 0.736761671 | 3.462502023 | 3.706567178 | 0.002030915 | 0.256559386 | -1.223887739 |
| HPR | 1.266508762 | 7.262144182 | 3.706429266 | 0.002031495 | 0.256559386 | -1.224124092 |
| CRAMP1L | -0.584500481 | 2.867792216 | -3.698313475 | 0.002065966 | 0.259852767 | -1.238034657 |
| PCNT | -0.433750676 | 7.790703029 | -3.694921147 | 0.002080548 | 0.259852767 | -1.243850137 |
| TSHZ1 | -0.435030871 | 8.78817774 | -3.694149554 | 0.002083879 | 0.259852767 | -1.245172961 |
| LOC101927780 | 1.108205031 | 1.957094806 | 3.692937691 | 0.002089122 | 0.259852767 | -1.247250648 |
| CHAF1B | -0.493619188 | 5.664234673 | -3.691386576 | 0.002095852 | 0.259852767 | -1.249910074 |
| UBTF | -0.453332235 | 6.495708815 | -3.68986714 | 0.002102466 | 0.259852767 | -1.2525153 |
| DZIP3 | -0.615496865 | 6.303733907 | -3.68738501 | 0.002113314 | 0.260221487 | -1.256771406 |
| HYMAI | 0.735071448 | 7.174703061 | 3.682189606 | 0.002136205 | 0.260221487 | -1.265680928 |
| UCK2 | -0.675351083 | 6.429965668 | -3.677576043 | 0.002156741 | 0.260221487 | -1.273593745 |
| KCNK1 | -1.021986431 | 1.820977507 | -3.677251038 | 0.002158195 | 0.260221487 | -1.274151208 |
| lnc-ARHGAP25-1 | 0.880231658 | 4.797385521 | 3.675674212 | 0.002165264 | 0.260221487 | -1.276855909 |
| WBP11 | -0.286301342 | 9.077543129 | -3.675151075 | 0.002167614 | 0.260221487 | -1.277753262 |
| MTMR2 | -0.418700988 | 5.770315501 | -3.672516296 | 0.002179491 | 0.260221487 | -1.282272979 |
| CIAPIN1 | -0.352447485 | 7.833309103 | -3.66440329 | 0.002216474 | 0.260221487 | -1.296192093 |
| lnc-RELT-1 | 0.872738751 | 8.490933455 | 3.664231947 | 0.002217262 | 0.260221487 | -1.296486091 |
| SATB1 | -0.549385266 | 10.66905514 | -3.663910109 | 0.002218743 | 0.260221487 | -1.297038318 |
| lnc-NADSYN1-1 | -0.494487335 | 5.02928425 | -3.662279779 | 0.002226258 | 0.260221487 | -1.299835799 |
| CDK5RAP3 | 0.361049169 | 8.396502897 | 3.660508164 | 0.002234454 | 0.260221487 | -1.302875846 |
| PMEPA1 | -1.04471226 | 5.389955498 | -3.660340088 | 0.002235233 | 0.260221487 | -1.303164268 |
| RNMTL1 | -0.455556367 | 6.634021809 | -3.654544526 | 0.002262265 | 0.260221487 | -1.313110325 |
| lnc-ADAMTS14-2 | 0.788307737 | 1.52282002 | 3.654176207 | 0.002263994 | 0.260221487 | -1.313742467 |
| ZNF215 | -1.207082345 | 1.841408086 | -3.653237497 | 0.002268407 | 0.260221487 | -1.315353589 |
| lnc-ERGIC2-4 | 1.29659294 | 4.484410435 | 3.653041432 | 0.00226933 | 0.260221487 | -1.315690103 |
| LOC101928346 | -0.634542582 | 6.033413617 | -3.651063691 | 0.002278659 | 0.260221487 | -1.31908467 |
| NMB | -0.355082874 | 7.754359507 | -3.650666284 | 0.002280538 | 0.260221487 | -1.319766796 |
| FPGS | -0.438181423 | 6.902938386 | -3.650001368 | 0.002283685 | 0.260221487 | -1.320908098 |
| lnc-FAM160A1-1 | -1.502530605 | 4.315169139 | -3.649977899 | 0.002283797 | 0.260221487 | -1.320948383 |
| EIF2B2 | -0.271672776 | 8.009710563 | -3.645825466 | 0.002303554 | 0.260221487 | -1.328076305 |
| RPRD2 | -0.377526195 | 8.179475508 | -3.645343552 | 0.002305858 | 0.260221487 | -1.328903589 |
| NONO | -0.356163813 | 10.3731484 | -3.641751001 | 0.002323107 | 0.260221487 | -1.335071103 |
| lnc-LGALSL-1 | 1.282654335 | 7.930737724 | 3.641682971 | 0.002323435 | 0.260221487 | -1.335187898 |
| NUCKS1 | -0.501759679 | 6.558339259 | -3.641398225 | 0.002324808 | 0.260221487 | -1.33567676 |
| LSM2 | -0.504712081 | 10.89193924 | -3.640719959 | 0.002328081 | 0.260221487 | -1.336841244 |
| GPAA1 | -0.305699334 | 11.28584854 | -3.64030767 | 0.002330073 | 0.260221487 | -1.337549093 |
| PRKCQ | -0.543388231 | 7.155611146 | -3.635842659 | 0.002351757 | 0.260221487 | -1.345215417 |
| OTUD7B | -0.633961169 | 3.914219474 | -3.635395037 | 0.002353942 | 0.260221487 | -1.345984019 |
| ITCH | -0.429067013 | 6.202181478 | -3.632758299 | 0.002366854 | 0.260221487 | -1.350511672 |
| RNASEL | 0.379612693 | 8.348600095 | 3.631420234 | 0.002373433 | 0.260221487 | -1.352809427 |
| HUS1B | -1.11978092 | 1.416659859 | -3.630281808 | 0.002379046 | 0.260221487 | -1.354764413 |
| LOC729040 | 1.082055305 | 7.034339073 | 3.630047686 | 0.002380202 | 0.260221487 | -1.35516647 |
| RNF157-AS1 | -1.206265373 | 5.482527972 | -3.628811117 | 0.002386316 | 0.260221487 | -1.357290065 |
| CEACAM1 | 1.321128938 | 8.827222509 | 3.628760437 | 0.002386567 | 0.260221487 | -1.357377101 |
| PRKACB | -0.471437263 | 9.014729842 | -3.627624639 | 0.002392197 | 0.260221487 | -1.359327695 |
| ANAPC1 | -0.45274465 | 8.169160842 | -3.627556417 | 0.002392536 | 0.260221487 | -1.359444859 |
| CD3G | -0.582963631 | 11.44498502 | -3.625115182 | 0.002404685 | 0.260221487 | -1.363637561 |
| TTC5 | -0.429432345 | 9.785184072 | -3.624525119 | 0.002407631 | 0.260221487 | -1.364651001 |
| DHRS3 | -0.57594453 | 8.605132298 | -3.617861743 | 0.002441149 | 0.260221487 | -1.376096356 |
| PLIN4 | 1.612442661 | 7.750540043 | 3.617539491 | 0.002442781 | 0.260221487 | -1.376649915 |
| FCGR2C | 0.813891868 | 11.64989818 | 3.616385471 | 0.002448638 | 0.260221487 | -1.378632306 |
| ADCY4 | 0.970268178 | 7.080882183 | 3.612914811 | 0.002466335 | 0.260221487 | -1.384594553 |
| PCNXL2 | -0.409096997 | 8.610719652 | -3.612755916 | 0.002467148 | 0.260221487 | -1.38486753 |
| TPM3P9 | -0.341804706 | 7.040811583 | -3.612645092 | 0.002467716 | 0.260221487 | -1.385057923 |
| MRPL37 | -0.407897626 | 7.952746776 | -3.609747359 | 0.002482598 | 0.260221487 | -1.390036312 |
| TMEM214 | -0.394991611 | 10.6739652 | -3.609685349 | 0.002482917 | 0.260221487 | -1.390142851 |
| BAZ1B | -0.452431565 | 8.421607318 | -3.606010521 | 0.002501923 | 0.260221487 | -1.396456764 |
| TMEM109 | -0.598872142 | 9.406008185 | -3.604740716 | 0.002508524 | 0.260221487 | -1.398638595 |
| CAMLG | -0.570657129 | 3.453071689 | -3.603899887 | 0.002512905 | 0.260221487 | -1.400083374 |
| H2AFX | -0.508214936 | 8.360522926 | -3.602069437 | 0.002522468 | 0.260221487 | -1.403228687 |
| MNDA | 0.554976401 | 12.82515531 | 3.601343475 | 0.002526271 | 0.260221487 | -1.404476161 |
| lnc-H3F3C-3 | 1.088724003 | 7.96455873 | 3.601014106 | 0.002527998 | 0.260221487 | -1.405042146 |
| HIATL1 | 0.976409254 | 8.166036744 | 3.597747838 | 0.002545191 | 0.260221487 | -1.410655079 |
| AAR2 | -0.394922752 | 7.954304462 | -3.597250932 | 0.002547817 | 0.260221487 | -1.411509023 |
| AGK | -0.663663321 | 5.341733042 | -3.596251312 | 0.002553107 | 0.260221487 | -1.413226916 |
| HSPBP1 | -0.420497721 | 8.503890016 | -3.595665395 | 0.002556214 | 0.260221487 | -1.414233858 |
| PPP2R5D | -0.263878628 | 8.027693481 | -3.595511622 | 0.002557029 | 0.260221487 | -1.414498129 |
| ISM2 | 0.896213851 | 1.566840415 | 3.594579893 | 0.002561978 | 0.260221487 | -1.416099405 |
| CTBP1 | -0.332305444 | 8.142693418 | -3.59448109 | 0.002562504 | 0.260221487 | -1.416269209 |
| BDH1 | -0.651422902 | 7.714465599 | -3.58998454 | 0.002586527 | 0.26136655 | -1.423997438 |
| H3F3C | 0.509683415 | 12.62942467 | 3.588458395 | 0.002594732 | 0.26136655 | -1.42662058 |
| PTPRS | -1.240269629 | 8.25024014 | -3.584813458 | 0.002614434 | 0.26136655 | -1.432885824 |
| PTPRCAP | -0.47923312 | 9.50460093 | -3.581203537 | 0.002634094 | 0.26136655 | -1.439091306 |
| POLR3A | -0.385398359 | 7.796603465 | -3.58004459 | 0.002640437 | 0.26136655 | -1.441083635 |
| lnc-PRICKLE4-1 | 0.719227388 | 4.405823834 | 3.579605997 | 0.002642842 | 0.26136655 | -1.441837625 |
| STT3A | -0.587028173 | 6.95756664 | -3.579284991 | 0.002644603 | 0.26136655 | -1.442389473 |
| lnc-CTD-2134A5.2.1-2 | -0.760424614 | 4.385226476 | -3.578829944 | 0.002647102 | 0.26136655 | -1.443171761 |
| OPN4 | 0.809790926 | 3.294896227 | 3.577786 | 0.002652843 | 0.26136655 | -1.444966467 |
| SDF2L1 | -0.644335952 | 10.82499883 | -3.57324851 | 0.002677943 | 0.26136655 | -1.45276753 |
| DCAKD | -0.573425629 | 3.286338863 | -3.573190187 | 0.002678267 | 0.26136655 | -1.452867806 |
| FLJ31713 | 1.016544588 | 3.242861452 | 3.573116737 | 0.002678676 | 0.26136655 | -1.452994091 |
| lnc-LRRC17-1 | 1.213358995 | 8.561999626 | 3.573102764 | 0.002678753 | 0.26136655 | -1.453018115 |
| GK | 1.072254953 | 6.770516234 | 3.573033412 | 0.002679139 | 0.26136655 | -1.453137353 |
| SNX10 | 0.560131141 | 9.356668603 | 3.5691109 | 0.002701039 | 0.262764897 | -1.459881658 |
| lnc-TARDBP-1 | -1.002779884 | 1.410407922 | -3.567243017 | 0.00271153 | 0.263048708 | -1.463093429 |
| TGIF1 | -0.45666366 | 6.335828343 | -3.565149763 | 0.002723336 | 0.263458092 | -1.466692845 |
| PSAT1 | -0.905557059 | 5.71499684 | -3.562524114 | 0.002738217 | 0.264161905 | -1.471207912 |
| lnc-OSBPL10-1 | -0.802368574 | 1.340703629 | -3.560453886 | 0.002750008 | 0.264564492 | -1.474768019 |
| PXMP2 | -0.466913652 | 6.14308191 | -3.55793663 | 0.002764414 | 0.265215694 | -1.479097032 |
| NUDT16P1 | 0.748545207 | 4.329905413 | 3.556088056 | 0.00277504 | 0.265501796 | -1.482276204 |
| RELT | 0.490324144 | 10.67383992 | 3.552201247 | 0.002797518 | 0.265794613 | -1.488961037 |
| TMEM140 | 0.928644996 | 7.23607428 | 3.550731462 | 0.002806065 | 0.265794613 | -1.491488996 |
| PYDC1 | 0.521158658 | 6.403385212 | 3.549925161 | 0.002810765 | 0.265794613 | -1.492875818 |
| DHX30 | -0.403376354 | 10.289559 | -3.548900709 | 0.002816748 | 0.265794613 | -1.494637883 |
| LAMP2 | 0.615625774 | 10.23286062 | 3.547543403 | 0.002824695 | 0.265794613 | -1.496972501 |
| CFAP58 | 0.914292513 | 4.533764336 | 3.547122965 | 0.002827161 | 0.265794613 | -1.497695679 |
| SMA4 | 1.312002663 | 9.330737537 | 3.546354659 | 0.002831673 | 0.265794613 | -1.499017226 |
| RORA | -0.516819834 | 9.542963322 | -3.54451481 | 0.002842507 | 0.266092406 | -1.502181974 |
| LOC100131792 | 1.496632307 | 1.627068678 | 3.5426261 | 0.002853673 | 0.266419489 | -1.50543086 |
| XLOC_l2_001569 | 0.895507885 | 1.39246572 | 3.540071848 | 0.002868842 | 0.266727127 | -1.50982473 |
| WDFY3 | 1.163466597 | 8.07427797 | 3.537970843 | 0.00288138 | 0.266727127 | -1.513439042 |
| S100A9 | 0.483633795 | 13.21925446 | 3.537225685 | 0.00288584 | 0.266727127 | -1.514720948 |
| UTP20 | -0.551877054 | 5.931030059 | -3.536917338 | 0.002887688 | 0.266727127 | -1.515251405 |
| CTNNA1 | 0.364380544 | 8.271934903 | 3.535017057 | 0.0028991 | 0.266793993 | -1.518520561 |
| lnc-POLR2B-2 | -0.536283708 | 1.157163865 | -3.530393708 | 0.002927056 | 0.266793993 | -1.526474724 |
| GINS3 | -0.653847908 | 6.211623776 | -3.528621729 | 0.002937841 | 0.266793993 | -1.529523432 |
| AKTIP | -0.47262198 | 6.292190278 | -3.527589524 | 0.002944142 | 0.266793993 | -1.531299385 |
| KRT2 | -0.978826127 | 1.194744578 | -3.527539069 | 0.002944451 | 0.266793993 | -1.531386195 |
| SIRPB2 | 1.078892504 | 7.108924247 | 3.527390258 | 0.00294536 | 0.266793993 | -1.531642232 |
| FBXL13 | 1.196456937 | 6.444842515 | 3.527204263 | 0.002946498 | 0.266793993 | -1.531962249 |
| SFMBT1 | -0.595182252 | 6.343290258 | -3.526595556 | 0.002950223 | 0.266793993 | -1.533009573 |
| HIST1H3A | 0.519153455 | 12.10631355 | 3.524498669 | 0.002963092 | 0.266793993 | -1.536617481 |
| lnc-ARNTL-1 | 0.660544422 | 5.491889035 | 3.52415094 | 0.002965231 | 0.266793993 | -1.537215793 |
| lnc-S1PR1-1 | -0.884680125 | 3.034500674 | -3.521831396 | 0.002979542 | 0.267388897 | -1.54120694 |
| TMEM106C | -0.41269738 | 8.622817094 | -3.519540789 | 0.002993743 | 0.26745983 | -1.545148414 |
| ESYT2 | -0.641172626 | 8.629776166 | -3.51922028 | 0.002995735 | 0.26745983 | -1.545699927 |
| SRGAP3 | -0.429193023 | 5.350706341 | -3.51732547 | 0.003007541 | 0.267825341 | -1.548960449 |
| LOC145474 | 0.851561488 | 7.441522584 | 3.515255296 | 0.003020492 | 0.268290758 | -1.55252282 |
| LOC100996412 | 0.445741849 | 4.880063481 | 3.51073814 | 0.003048946 | 0.26878245 | -1.560296287 |
| SERPINF1 | -0.94282087 | 7.284722537 | -3.50850636 | 0.003063103 | 0.26878245 | -1.564137061 |
| GREB1L | 0.437471093 | 1.112671497 | 3.507554602 | 0.003069161 | 0.26878245 | -1.565775014 |
| NACAP1 | -0.374734758 | 8.64639434 | -3.506254487 | 0.003077455 | 0.26878245 | -1.568012513 |
| LOC100130370 | 0.754284704 | 1.315314564 | 3.505943999 | 0.003079439 | 0.26878245 | -1.568546868 |
| lnc-PAPLN-1 | 0.676082901 | 4.889304642 | 3.504945375 | 0.003085828 | 0.26878245 | -1.570265528 |
| IL21R | -0.57248397 | 7.913426866 | -3.503649586 | 0.003094139 | 0.26878245 | -1.572495647 |
| MRPS18B | -0.529322265 | 10.46857799 | -3.50348029 | 0.003095227 | 0.26878245 | -1.572787017 |
| lnc-HMGB2-1 | 1.148977058 | 1.925764903 | 3.503409723 | 0.00309568 | 0.26878245 | -1.572908468 |
| WFS1 | -0.775966107 | 3.962481639 | -3.497315234 | 0.003135091 | 0.269705494 | -1.58339785 |
| TMEM263 | -0.791891594 | 8.377972002 | -3.492393059 | 0.003167286 | 0.269705494 | -1.591870024 |
| KRT86 | -1.118444138 | 5.655366167 | -3.492242689 | 0.003168275 | 0.269705494 | -1.592128853 |
| IL2RB | -0.856024488 | 11.04249472 | -3.49132885 | 0.00317429 | 0.269705494 | -1.593701824 |
| lnc-AF127577.1-4 | 0.778709564 | 4.480864936 | 3.490216733 | 0.003181626 | 0.269705494 | -1.595616107 |
| LPAL2 | -0.91403517 | 2.754209667 | -3.48956169 | 0.003185955 | 0.269705494 | -1.596743641 |
| ANKS6 | -0.68983928 | 7.695328354 | -3.487709087 | 0.00319823 | 0.269705494 | -1.599932587 |
| RBFA | -0.47434782 | 6.729841777 | -3.487308538 | 0.00320089 | 0.269705494 | -1.600622074 |
| SSR3 | -0.531093507 | 4.775548179 | -3.486003746 | 0.003209571 | 0.269705494 | -1.602868096 |
| SET | -0.416610767 | 11.80957461 | -3.484094303 | 0.003222317 | 0.269705494 | -1.606154995 |
| LOC101928246 | 0.847920421 | 1.155550112 | 3.48387964 | 0.003223753 | 0.269705494 | -1.606524518 |
| PAIP2B | -0.907048639 | 3.782730302 | -3.482009006 | 0.003236295 | 0.269705494 | -1.609744672 |
| IL24 | -0.855110924 | 4.177724551 | -3.478784482 | 0.003258028 | 0.269705494 | -1.615295574 |
| ACSS2 | 0.405027038 | 7.20358258 | 3.47715786 | 0.003269047 | 0.269705494 | -1.618095802 |
| MAN1B1-AS1 | -0.791720203 | 4.462181041 | -3.475648312 | 0.003279306 | 0.269705494 | -1.620694524 |
| NDUFV1 | -0.394010783 | 11.16927156 | -3.474968477 | 0.003283936 | 0.269705494 | -1.621864886 |
| TDP1 | -0.519253943 | 8.177649127 | -3.472416187 | 0.00330138 | 0.269705494 | -1.626258811 |
| MSH2 | -0.822390006 | 7.507922051 | -3.472193565 | 0.003302905 | 0.269705494 | -1.626642073 |
| RASGRF2-AS1 | -1.348962052 | 2.060536518 | -3.470487886 | 0.00331462 | 0.269705494 | -1.629578559 |
| USP32 | 0.773861359 | 8.042741781 | 3.469653012 | 0.003320368 | 0.269705494 | -1.631015887 |
| lnc-AC007405.7.1-1 | -0.508896091 | 0.918128774 | -3.468947019 | 0.003325238 | 0.269705494 | -1.63223134 |
| lnc-GPR144-1 | -0.850278447 | 3.76203693 | -3.468842302 | 0.00332596 | 0.269705494 | -1.632411623 |
| METRN | -0.518777232 | 8.511215242 | -3.465131273 | 0.003351678 | 0.269705494 | -1.638800721 |
| ABCF3 | -0.22478246 | 9.008691525 | -3.462571922 | 0.00336953 | 0.269705494 | -1.643207132 |
| PPP5C | -0.780285 | 2.876403562 | -3.462075976 | 0.003373 | 0.269705494 | -1.644061007 |
| SKAP1 | -0.723923424 | 11.12320231 | -3.460288713 | 0.003385536 | 0.269705494 | -1.647138179 |
| NRG2 | -1.499048019 | 2.33351159 | -3.4582984 | 0.00339955 | 0.269705494 | -1.65056499 |
| VPS11 | -0.34672115 | 10.57181535 | -3.455641025 | 0.003418352 | 0.269705494 | -1.65514038 |
| lnc-BLID-3 | 0.831006445 | 5.661748053 | 3.455571551 | 0.003418845 | 0.269705494 | -1.655259999 |
| PEAK1 | -0.317615042 | 7.234113523 | -3.454187609 | 0.00342868 | 0.269705494 | -1.657642861 |
| P4HB | -0.419110208 | 12.4861573 | -3.454083912 | 0.003429418 | 0.269705494 | -1.657821407 |
| lnc-FBXL22-1 | -0.901266022 | 2.581794291 | -3.452409753 | 0.003441355 | 0.269705494 | -1.660703994 |
| TAS2R19 | -0.953989322 | 3.358394819 | -3.451465627 | 0.003448105 | 0.269705494 | -1.662329613 |
| USP28 | -1.017154995 | 2.467213792 | -3.44735537 | 0.003477646 | 0.269705494 | -1.669406855 |
| GLCCI1 | -0.487031742 | 8.139619876 | -3.447182249 | 0.003478895 | 0.269705494 | -1.669704946 |
| C8orf60 | 1.068721411 | 10.11056057 | 3.447165737 | 0.003479015 | 0.269705494 | -1.669733378 |
| SMARCB1 | -0.461014991 | 8.949275458 | -3.443821366 | 0.003503247 | 0.269705494 | -1.675492003 |
| NBEA | -1.102353993 | 4.7344695 | -3.442309954 | 0.003514253 | 0.269705494 | -1.678094514 |
| PPIH | -0.500262299 | 7.993036046 | -3.441340552 | 0.003521331 | 0.269705494 | -1.679763744 |
| ELP6 | -0.326817688 | 8.440828762 | -3.438307638 | 0.003543566 | 0.269705494 | -1.684986221 |
| HIST1H3C | 0.51534063 | 11.11480041 | 3.436954803 | 0.003553529 | 0.269705494 | -1.687315738 |
| TNFRSF21 | -1.147275102 | 5.230242134 | -3.434242554 | 0.003573587 | 0.269705494 | -1.691986138 |
| lnc-AC018816.3.1-4 | -1.282410054 | 3.342907441 | -3.431681099 | 0.003592635 | 0.269705494 | -1.696396924 |
| LOC100130539 | 0.821720603 | 5.812759096 | 3.431522486 | 0.003593817 | 0.269705494 | -1.696670054 |
| PAAF1 | -0.521857079 | 8.232239037 | -3.431319287 | 0.003595333 | 0.269705494 | -1.697019961 |
| SIX5 | -0.383288445 | 5.192809335 | -3.430364832 | 0.003602462 | 0.269705494 | -1.698663531 |
| ZDHHC14 | -0.403239995 | 8.357450114 | -3.42821217 | 0.003618592 | 0.269705494 | -1.702370432 |
| KLRB1 | -0.979064846 | 10.79451195 | -3.428010025 | 0.00362011 | 0.269705494 | -1.702718529 |
| RASD1 | -0.776468116 | 5.819901014 | -3.426301407 | 0.003632969 | 0.269705494 | -1.705660804 |
| FAM69A | -0.633917059 | 7.782965433 | -3.423539825 | 0.003653849 | 0.269705494 | -1.710416339 |
| CLN3 | 0.425833308 | 6.309369139 | 3.423249854 | 0.003656049 | 0.269705494 | -1.71091568 |
| LIMCH1 | -1.022172637 | 1.718756238 | -3.42256023 | 0.003661285 | 0.269705494 | -1.712103242 |
| C9orf72 | 0.563889246 | 6.144146413 | 3.42002588 | 0.003680591 | 0.269705494 | -1.716467521 |
| lnc-PERP-3 | -0.840516725 | 2.081517635 | -3.419319156 | 0.003685993 | 0.269705494 | -1.717684542 |
| MCM7 | -0.405673288 | 10.84131551 | -3.416022668 | 0.003711294 | 0.269705494 | -1.723361311 |
| ATP7B | 0.427507689 | 4.783074087 | 3.415164307 | 0.003717911 | 0.269705494 | -1.724839473 |
| SARS2 | -0.46330096 | 5.667731837 | -3.413769498 | 0.003728687 | 0.269705494 | -1.727241441 |
| SCAMP5 | -1.351231851 | 4.323578046 | -3.411453354 | 0.003746651 | 0.269705494 | -1.731230036 |
| DRG2 | -0.312699999 | 7.899353968 | -3.410653958 | 0.003752871 | 0.269705494 | -1.732606667 |
| PTGS2 | 0.682318848 | 6.244927984 | 3.41047954 | 0.00375423 | 0.269705494 | -1.73290703 |
| LINC01568 | 0.286286228 | 1.094405602 | 3.40828797 | 0.003771342 | 0.269705494 | -1.736681115 |
| KATNBL1 | 0.658022321 | 4.601607933 | 3.407984094 | 0.00377372 | 0.269705494 | -1.737204419 |
| HGH1 | -0.34691129 | 7.118799345 | -3.407492313 | 0.003777573 | 0.269705494 | -1.738051314 |
| PTPLAD2 | 0.400417566 | 8.002126879 | 3.406995549 | 0.003781469 | 0.269705494 | -1.73890679 |
| RPAP1 | -0.335899885 | 8.04867132 | -3.406743618 | 0.003783446 | 0.269705494 | -1.73934064 |
| SNORA11 | -0.32244684 | 3.510315543 | -3.406197684 | 0.003787735 | 0.269705494 | -1.740280792 |
| LINC01359 | 1.076799907 | 3.434779852 | 3.404486382 | 0.003801209 | 0.269705494 | -1.743227826 |
| HIRIP3 | -0.441572832 | 8.635246297 | -3.404398622 | 0.003801901 | 0.269705494 | -1.743378957 |
| lnc-LONRF1-2 | 0.909097114 | 7.898287672 | 3.404394289 | 0.003801935 | 0.269705494 | -1.74338642 |
| POP7 | -0.358911054 | 10.57918248 | -3.4010756 | 0.003828206 | 0.269705494 | -1.749101549 |
| ZNF616 | -0.423907117 | 6.057605411 | -3.400814491 | 0.003830281 | 0.269705494 | -1.749551207 |
| CRK | 0.505949285 | 5.600203115 | 3.398933929 | 0.003845256 | 0.269705494 | -1.752789735 |
| lnc-C17orf75-2 | 0.84036742 | 2.894350789 | 3.398035179 | 0.003852433 | 0.269705494 | -1.754337481 |
| ZNF614 | -0.575965731 | 6.459816041 | -3.397778808 | 0.003854483 | 0.269705494 | -1.754778979 |
| STK3 | 0.701576799 | 6.71929457 | 3.397600813 | 0.003855906 | 0.269705494 | -1.755085505 |
| F2RL1 | 0.722994097 | 7.818096467 | 3.397124546 | 0.003859719 | 0.269705494 | -1.755905689 |
| FTO | -0.35838294 | 7.75962938 | -3.396894967 | 0.003861558 | 0.269705494 | -1.756301049 |
| ETV6 | 0.380292103 | 7.466825152 | 3.394666906 | 0.003879451 | 0.269705494 | -1.760138012 |
| TREM1 | 0.941336728 | 9.273264609 | 3.393972906 | 0.003885041 | 0.269705494 | -1.761333155 |
| CD46 | 0.544213685 | 6.614697994 | 3.391653853 | 0.003903779 | 0.269705494 | -1.765326812 |
| TRAP1 | -0.492282026 | 5.208425641 | -3.390624393 | 0.003912126 | 0.269705494 | -1.767099651 |
| RNF126 | -0.437465229 | 10.73978545 | -3.390528632 | 0.003912903 | 0.269705494 | -1.767264562 |
| XYLT2 | -0.392134828 | 9.756726654 | -3.388643494 | 0.003928237 | 0.269705494 | -1.770510966 |
| MAD1L1 | -0.536738266 | 6.754368072 | -3.385772107 | 0.003951709 | 0.269705494 | -1.775455785 |
| PGAP3 | -0.440382866 | 7.977684306 | -3.384451898 | 0.003962547 | 0.269705494 | -1.777729313 |
| CCAR2 | -0.423627563 | 6.17833931 | -3.383063358 | 0.003973978 | 0.269705494 | -1.780120508 |
| lnc-GRB7-1 | -0.768636076 | 2.529576468 | -3.382360377 | 0.003979778 | 0.269705494 | -1.781331105 |
| FNBP1L | -0.675244487 | 3.799817984 | -3.382082144 | 0.003982076 | 0.269705494 | -1.781810248 |
| ALOX12-AS1 | 0.331434006 | 6.712914074 | 3.381799544 | 0.003984412 | 0.269705494 | -1.78229691 |
| lnc-GPR123-4 | -0.632021532 | 1.046777688 | -3.379610271 | 0.004002549 | 0.269705494 | -1.786067026 |
| GHDC | -0.361278772 | 6.754685946 | -3.379363053 | 0.004004603 | 0.269705494 | -1.786492755 |
| KU-MEL-3 | 0.447245483 | 6.503279961 | 3.377499928 | 0.004020111 | 0.269705494 | -1.789701203 |
| EXOSC10 | -0.343244454 | 8.732456371 | -3.37674217 | 0.004026436 | 0.269705494 | -1.79100612 |
| HIST1H2BC | 0.660309069 | 6.524017787 | 3.37639699 | 0.00402932 | 0.269705494 | -1.791600544 |
| DNAAF5 | -0.442793844 | 6.338544199 | -3.374966096 | 0.004041298 | 0.269705494 | -1.794064643 |
| ZNF185 | 0.625297216 | 6.50747893 | 3.374802539 | 0.00404267 | 0.269705494 | -1.794346298 |
| BRINP1 | 0.687566651 | 1.254083268 | 3.369654057 | 0.004086077 | 0.269705494 | -1.803212263 |
| GLA | 0.340059524 | 9.662708255 | 3.367776174 | 0.004102025 | 0.269705494 | -1.806446047 |
| WBSCR22 | -0.401652433 | 9.054363134 | -3.367640783 | 0.004103177 | 0.269705494 | -1.806679196 |
| GRIK1-AS1 | 1.582156316 | 3.740658718 | 3.367557778 | 0.004103884 | 0.269705494 | -1.806822132 |
| TARS2 | -0.271001458 | 7.264854724 | -3.367373498 | 0.004105453 | 0.269705494 | -1.807139469 |
| SPTAN1 | -0.392739194 | 8.991666999 | -3.365642491 | 0.004120221 | 0.269705494 | -1.810120307 |
| FANCI | -0.390218004 | 6.062181716 | -3.364297192 | 0.004131734 | 0.269705494 | -1.812436934 |
| AKR1C1 | -1.10564257 | 2.38088442 | -3.363403815 | 0.004139398 | 0.269705494 | -1.813975337 |
| ZFAT | -0.401702489 | 7.997439793 | -3.361967903 | 0.004151745 | 0.269705494 | -1.816447981 |
| LOC101928143 | 1.031997512 | 6.986099237 | 3.3615108 | 0.004155684 | 0.269705494 | -1.817235112 |
| ZNF317 | -0.215840185 | 8.210574693 | -3.360330405 | 0.004165871 | 0.269705494 | -1.819267741 |
| lnc-RASA1-5 | 0.827521717 | 6.139413489 | 3.359963309 | 0.004169044 | 0.269705494 | -1.819899876 |
| SCAMP3 | -0.384082223 | 11.19043147 | -3.359883123 | 0.004169737 | 0.269705494 | -1.820037954 |
| HTATIP2 | 0.338948274 | 8.606003933 | 3.359803882 | 0.004170423 | 0.269705494 | -1.820174407 |
| LOC102723564 | -0.555051719 | 6.016536497 | -3.359629264 | 0.004171933 | 0.269705494 | -1.820475096 |
| KIF11 | -1.064368632 | 4.238631259 | -3.359220081 | 0.004175476 | 0.269705494 | -1.821179702 |
| HNRNPA3 | -0.370348808 | 11.16522161 | -3.357003977 | 0.004194713 | 0.269705494 | -1.82499577 |
| PRKCH | -0.614829055 | 10.52279012 | -3.356312204 | 0.004200735 | 0.269705494 | -1.826186975 |
| XLOC_l2_001496 | 0.994345196 | 5.980693475 | 3.356192187 | 0.004201781 | 0.269705494 | -1.82639364 |
| FRG2C | 0.743739205 | 1.469971283 | 3.355633374 | 0.004206654 | 0.269705494 | -1.827355891 |
| ABI2 | -0.67884736 | 6.333275829 | -3.355197359 | 0.00421046 | 0.269705494 | -1.828106687 |
| KCNJ2-AS1 | 1.419842136 | 2.371683356 | 3.354753318 | 0.00421434 | 0.269705494 | -1.828871304 |
| GAR1 | -0.454922037 | 9.094965069 | -3.354535287 | 0.004216246 | 0.269705494 | -1.829246742 |
| lnc-SLC16A3-5 | 0.653984458 | 3.589524225 | 3.35453348 | 0.004216262 | 0.269705494 | -1.829249852 |
| SSFA2 | 0.302923608 | 8.515434092 | 3.354260165 | 0.004218652 | 0.269705494 | -1.829720486 |
| DHRS7B | 0.314842979 | 6.416709438 | 3.352930499 | 0.004230302 | 0.269705494 | -1.832010088 |
| LOC283440 | -0.893258768 | 2.648858837 | -3.351508566 | 0.004242796 | 0.269705494 | -1.834458551 |
| IGSF9 | 0.909875281 | 4.780780336 | 3.351109996 | 0.004246305 | 0.269705494 | -1.835144855 |
| SPECC1 | 0.419996216 | 8.295841918 | 3.351099622 | 0.004246397 | 0.269705494 | -1.835162719 |
| MTA2 | -0.402002564 | 7.615416732 | -3.350671719 | 0.004250167 | 0.269705494 | -1.835899531 |
| SHPK | -0.277759013 | 6.67301354 | -3.349303085 | 0.004262248 | 0.269705494 | -1.838256192 |
| XLOC_l2_015212 | 1.027297047 | 4.578292502 | 3.348277423 | 0.004271324 | 0.269705494 | -1.840022276 |
| LOC102723729 | -0.814264696 | 1.383414468 | -3.34721144 | 0.004280777 | 0.269705494 | -1.841857778 |
| lnc-PANK4-1 | 1.111983729 | 2.19845912 | 3.345776079 | 0.004293539 | 0.269705494 | -1.844329292 |
| lnc-ARHGEF5-1 | 0.929576647 | 1.337546439 | 3.345766548 | 0.004293624 | 0.269705494 | -1.844345703 |
| SBK1 | -0.554416538 | 8.852502319 | -3.344599426 | 0.004304029 | 0.269705494 | -1.846355327 |
| KLHL31 | -1.088978804 | 1.74456545 | -3.344147671 | 0.004308063 | 0.269705494 | -1.847133182 |
| PELP1 | -0.397922593 | 9.732684551 | -3.343999091 | 0.004309391 | 0.269705494 | -1.847389015 |
| CASC22 | 0.850331604 | 2.001995891 | 3.343164651 | 0.004316855 | 0.269705494 | -1.848825793 |
| LOC100128882 | 0.950501377 | 3.447044617 | 3.342803131 | 0.004320092 | 0.269705494 | -1.849448273 |
| NOL3 | 0.355853044 | 6.758118148 | 3.342648732 | 0.004321476 | 0.269705494 | -1.849714124 |
| NMT2 | -0.656414049 | 5.53659846 | -3.342400316 | 0.004323703 | 0.269705494 | -1.850141855 |
| LOC102723385 | -1.482426553 | 2.257275062 | -3.342268091 | 0.004324888 | 0.269705494 | -1.850369525 |
| STARD7-AS1 | 1.02798182 | 5.395773359 | 3.340291211 | 0.004342655 | 0.269705494 | -1.853773364 |
| PMPCA | -0.257685751 | 8.127423835 | -3.339635267 | 0.004348566 | 0.269705494 | -1.854902774 |
| FAM71F2 | 1.049509066 | 3.375231543 | 3.338811008 | 0.004356005 | 0.269705494 | -1.856321985 |
| RRAS2 | -0.69207245 | 8.158985813 | -3.337239717 | 0.004370222 | 0.269705494 | -1.859027416 |
| TLR6 | 0.773249598 | 6.071863662 | 3.336713273 | 0.004374995 | 0.269705494 | -1.859933835 |
| XLOC_l2_007835 | 0.726065199 | 7.830102128 | 3.335175206 | 0.004388971 | 0.269705494 | -1.862582025 |
| CCDC102A | -0.612790702 | 6.515764203 | -3.335102386 | 0.004389634 | 0.269705494 | -1.862707404 |
| MESDC1 | -0.302331893 | 9.549181665 | -3.334829924 | 0.004392114 | 0.269705494 | -1.863176517 |
| C1orf109 | -0.366864032 | 6.333202758 | -3.334825023 | 0.004392159 | 0.269705494 | -1.863184954 |
| LOC101927979 | -0.592331192 | 4.071840329 | -3.333893841 | 0.004400648 | 0.269705494 | -1.864788213 |
| ADH1C | 0.779758541 | 1.111544224 | 3.332812203 | 0.004410529 | 0.269705494 | -1.866650508 |
| TAF6 | -0.27831868 | 9.608196815 | -3.329417166 | 0.004441687 | 0.269705494 | -1.872495768 |
| PDP2 | -0.58186085 | 7.009219586 | -3.327655024 | 0.004457945 | 0.269705494 | -1.875529604 |
| lnc-IRF2BP2-2 | 0.794043025 | 3.984622989 | 3.327353057 | 0.004460737 | 0.269705494 | -1.876049489 |
| LOC100132495 | 0.589637895 | 5.950410307 | 3.327029978 | 0.004463726 | 0.269705494 | -1.876605721 |
| TTYH3 | 0.444980358 | 6.695727574 | 3.326465178 | 0.004468957 | 0.269705494 | -1.87757811 |
| MIR1247 | 0.770425793 | 1.273093565 | 3.324649511 | 0.004485812 | 0.269705494 | -1.880704027 |
| S1PR1 | -0.604131285 | 8.896952556 | -3.324402469 | 0.00448811 | 0.269705494 | -1.88112934 |
| PRR7-AS1 | 0.506423747 | 2.574351422 | 3.322555101 | 0.004505333 | 0.269705494 | -1.884309781 |
| lnc-RLBP1-1 | 0.863715806 | 3.710752898 | 3.322553686 | 0.004505347 | 0.269705494 | -1.884312218 |
| lnc-RNF186-1 | -0.213878235 | 6.273739076 | -3.321276349 | 0.004517294 | 0.269705494 | -1.886511262 |
| CLEC18B | 1.144973817 | 6.668948873 | 3.317582013 | 0.004552025 | 0.269705494 | -1.892871244 |
| TATDN2 | -0.311008552 | 8.431268424 | -3.316733004 | 0.004560044 | 0.269705494 | -1.894332826 |
| KCNE3 | 0.651309829 | 8.600526216 | 3.315585071 | 0.004570909 | 0.269705494 | -1.896308993 |
| SLC16A5 | 0.475761472 | 10.55403902 | 3.313855635 | 0.004587326 | 0.269705494 | -1.89928618 |
| LYRM7 | -0.710306449 | 3.354560506 | -3.313800989 | 0.004587846 | 0.269705494 | -1.899380251 |
| CCNDBP1 | 0.325023698 | 9.629048916 | 3.313713814 | 0.004588675 | 0.269705494 | -1.899530319 |
| TSPAN31 | -0.357877338 | 5.588626469 | -3.313569786 | 0.004590045 | 0.269705494 | -1.899778259 |
| SF3B4 | 0.851752834 | 2.381410136 | 3.313422873 | 0.004591443 | 0.269705494 | -1.900031163 |
| CRISP2 | 1.532075233 | 2.804298556 | 3.313279748 | 0.004592806 | 0.269705494 | -1.900277546 |
| XLOC_l2_007834 | -0.728216148 | 2.236794271 | -3.311812503 | 0.004606796 | 0.269705494 | -1.902803325 |
| PDRG1 | -0.273557473 | 7.964371604 | -3.310141164 | 0.004622785 | 0.269705494 | -1.9056804 |
| HIST2H2AB | 0.656813949 | 9.407202867 | 3.30986732 | 0.00462541 | 0.269705494 | -1.906151796 |
| ZNF250 | -0.457427381 | 5.781900965 | -3.309852813 | 0.004625549 | 0.269705494 | -1.906176768 |
| CDC25A | -0.942515447 | 1.181480764 | -3.307509175 | 0.004648075 | 0.269705494 | -1.910211061 |
| ICK | -0.652866468 | 6.598329197 | -3.306987678 | 0.004653102 | 0.269705494 | -1.911108743 |
| lnc-RPS24-2 | 0.403489309 | 4.755639292 | 3.306609511 | 0.004656751 | 0.269705494 | -1.911759699 |
| HIST1H2AC | 0.763067209 | 9.347231718 | 3.30651301 | 0.004657683 | 0.269705494 | -1.911925811 |
| MYBPC3 | 1.069783235 | 7.763856511 | 3.305815278 | 0.004664424 | 0.269705494 | -1.913126843 |
| PEBP1 | -0.7855383 | 10.89767482 | -3.305676939 | 0.004665762 | 0.269705494 | -1.913364971 |
| GOT2 | -0.456581621 | 10.46342563 | -3.305446767 | 0.004667988 | 0.269705494 | -1.913761171 |
| FHL2 | 0.467292886 | 6.477948683 | 3.305180761 | 0.004670563 | 0.269705494 | -1.914219053 |
| EXO1 | -0.853682886 | 2.331409388 | -3.305027793 | 0.004672044 | 0.269705494 | -1.91448236 |
| PCBP4 | -0.43153985 | 8.089225556 | -3.304881175 | 0.004673464 | 0.269705494 | -1.914734736 |
| lnc-FAM92A1-1 | 0.907120601 | 4.290130577 | 3.303545617 | 0.004686419 | 0.269705494 | -1.917033636 |
| LOC102723428 | -0.541114446 | 5.410725081 | -3.302654856 | 0.00469508 | 0.269705494 | -1.918566889 |
| PFDN6 | -0.348812949 | 7.173701809 | -3.302285232 | 0.004698678 | 0.269705494 | -1.919203111 |
| FAHD2A | -0.350246871 | 6.894480618 | -3.301326971 | 0.00470802 | 0.269705494 | -1.920852529 |
| MLLT11 | -0.439611243 | 6.899356311 | -3.300620686 | 0.004714917 | 0.269705494 | -1.922068218 |
| SARS | -0.337006074 | 10.56091058 | -3.29978237 | 0.004723116 | 0.269705494 | -1.923511154 |
| ICOS | -0.70457216 | 7.347041056 | -3.298620732 | 0.004734501 | 0.269705494 | -1.925510579 |
| ZNF624 | -0.844564456 | 4.201264246 | -3.298377441 | 0.004736889 | 0.269705494 | -1.925929332 |
| LINC01301 | 0.587926193 | 5.454949372 | 3.297877123 | 0.004741804 | 0.269705494 | -1.926790474 |
| PCID2 | -0.572769467 | 9.345988407 | -3.297384395 | 0.004746649 | 0.269705494 | -1.927638549 |
| CARD17 | 0.737675601 | 8.084308622 | 3.296590966 | 0.004754461 | 0.269705494 | -1.929004177 |
| CRYGD | 0.58860355 | 1.042415241 | 3.295448579 | 0.004765731 | 0.269705494 | -1.930970398 |
| SLAMF6 | -0.438175875 | 5.809643404 | -3.294954936 | 0.004770609 | 0.269705494 | -1.931820024 |
| C9orf139 | 0.865714337 | 7.18253398 | 3.294392564 | 0.004776172 | 0.269705494 | -1.932787937 |
| MRPS17 | -0.428020622 | 7.633972464 | -3.294371883 | 0.004776377 | 0.269705494 | -1.932823531 |
| POLR2G | -0.38822605 | 11.08063102 | -3.293531286 | 0.004784705 | 0.269705494 | -1.934270292 |
| CD2 | -0.574218487 | 12.57438563 | -3.293447266 | 0.004785538 | 0.269705494 | -1.934414899 |
| TACC1 | 0.299217339 | 8.776438666 | 3.293216893 | 0.004787824 | 0.269705494 | -1.934811395 |
| TAX1BP3 | 0.304332548 | 4.708243529 | 3.29273634 | 0.004792594 | 0.269705494 | -1.93563847 |
| FBXL5 | 0.423486776 | 11.40771689 | 3.291997906 | 0.004799934 | 0.269705494 | -1.936909374 |
| LOC90246 | 0.790148789 | 1.443691056 | 3.290245459 | 0.004817398 | 0.269705494 | -1.939925431 |
| UQCC1 | -0.535828583 | 6.958559675 | -3.287008076 | 0.004849825 | 0.269705494 | -1.945496978 |
| C14orf159 | 0.438971801 | 9.522412161 | 3.286684829 | 0.004853075 | 0.269705494 | -1.946053276 |
| GCA | 0.820731875 | 13.34385591 | 3.286382455 | 0.004856116 | 0.269705494 | -1.946573649 |
| LOC100128310 | 0.874121585 | 1.925586148 | 3.284793061 | 0.004872136 | 0.269705494 | -1.949308901 |
| PLEKHG2 | -0.336959905 | 6.985118548 | -3.283884177 | 0.004881321 | 0.269705494 | -1.950873011 |
| NDUFA7 | -0.426070323 | 10.60583645 | -3.281993028 | 0.004900486 | 0.269705494 | -1.954127454 |
| TMUB1 | -0.326605163 | 11.16196421 | -3.280787445 | 0.004912743 | 0.269705494 | -1.95620208 |
| TGFBRAP1 | -0.333017185 | 7.434668541 | -3.280221612 | 0.004918506 | 0.269705494 | -1.95717578 |
| LOC101927841 | -1.066718617 | 2.19427636 | -3.280058672 | 0.004920167 | 0.269705494 | -1.957456172 |
| lnc-MRPS9-1 | -0.748549407 | 1.197449329 | -3.278515036 | 0.004935929 | 0.269705494 | -1.960112461 |
| LYAR | -0.579172127 | 8.305794204 | -3.277976687 | 0.004941438 | 0.269705494 | -1.96103884 |
| ANKRD39 | -0.438724966 | 7.720167174 | -3.277872682 | 0.004942503 | 0.269705494 | -1.961217808 |
| PISD | 0.857407078 | 7.208970975 | 3.277690206 | 0.004944371 | 0.269705494 | -1.961531806 |
| CLEC4E | 1.242407434 | 6.057362287 | 3.277633003 | 0.004944958 | 0.269705494 | -1.96163024 |
| lnc-TGS1-1 | 0.940967538 | 8.447278506 | 3.274630588 | 0.004975814 | 0.269705494 | -1.96679658 |
| ERC1 | -0.410758222 | 6.020571776 | -3.274554657 | 0.004976597 | 0.269705494 | -1.966927233 |
| CKAP5 | -0.50416017 | 6.92510624 | -3.273907177 | 0.004983277 | 0.269705494 | -1.968041342 |
| LY96 | 0.757078765 | 10.427177 | 3.273411902 | 0.004988393 | 0.269705494 | -1.968893547 |
| lnc-LAMA5-1 | -0.995333913 | 1.716281906 | -3.272840557 | 0.004994301 | 0.269705494 | -1.969876636 |
| UBXN10-AS1 | -0.979441699 | 3.941423257 | -3.271591094 | 0.005007246 | 0.269705494 | -1.972026505 |
| OR6K2 | 1.150220497 | 1.975827494 | 3.27100213 | 0.005013359 | 0.269705494 | -1.973039884 |
| CDAN1 | -0.431334048 | 4.210924281 | -3.270553444 | 0.005018021 | 0.269705494 | -1.973811894 |
| CARD11 | -0.506163614 | 6.736218518 | -3.270204864 | 0.005021646 | 0.269705494 | -1.974411657 |
| MXD1 | 0.683831135 | 12.26832873 | 3.269154401 | 0.005032586 | 0.269705494 | -1.976219057 |
| OLFM2 | -0.78773092 | 2.585638234 | -3.268806088 | 0.005036219 | 0.269705494 | -1.976818349 |
| IKZF3 | -0.527873266 | 12.06023853 | -3.267671739 | 0.005048068 | 0.269705494 | -1.97877004 |
| CUL7 | -0.35018865 | 3.874239078 | -3.26726792 | 0.005052292 | 0.269705494 | -1.979464818 |
| COASY | 0.293845094 | 7.4857067 | 3.267138332 | 0.005053649 | 0.269705494 | -1.979687774 |
| lnc-FRMD1-3 | 0.783263564 | 6.108184255 | 3.267064797 | 0.005054419 | 0.269705494 | -1.979814292 |
| CTRL | 0.45305095 | 5.31292225 | 3.267046965 | 0.005054605 | 0.269705494 | -1.979844973 |
| DDX11-AS1 | -0.888860556 | 1.454848691 | -3.266386142 | 0.005061529 | 0.269705494 | -1.980981918 |
| LOC100287042 | -0.975889148 | 2.038455449 | -3.26413394 | 0.005085198 | 0.269705494 | -1.984856749 |
| LOC645354 | -0.746084768 | 1.243233275 | -3.263891038 | 0.005087758 | 0.269705494 | -1.985274645 |
| HIST1H3G | 0.468925226 | 8.641978821 | 3.263560236 | 0.005091245 | 0.269705494 | -1.985843764 |
| NPHS2 | 1.296974778 | 1.565859982 | 3.263527399 | 0.005091592 | 0.269705494 | -1.985900258 |
| LOC101930246 | 0.939620021 | 5.518109241 | 3.263519419 | 0.005091676 | 0.269705494 | -1.985913986 |
| PTPN4 | -0.547766895 | 7.621500925 | -3.262536912 | 0.005102049 | 0.269705494 | -1.987604296 |
| lnc-NDUFA4-1 | 0.988675307 | 4.119382277 | 3.261913186 | 0.005108645 | 0.269705494 | -1.988677346 |
| TNFSF13 | 0.420698474 | 6.361627304 | 3.26141013 | 0.005113971 | 0.269705494 | -1.989542788 |
| ZSWIM6 | 0.42771149 | 8.281353885 | 3.261301549 | 0.005115121 | 0.269705494 | -1.989729586 |
| SLC17A3 | 0.650290514 | 1.140440638 | 3.25975391 | 0.005131544 | 0.269705494 | -1.992392052 |
| ZBTB7B | 0.427816643 | 5.947707118 | 3.259565721 | 0.005133545 | 0.269705494 | -1.992715796 |
| FKBP11 | -0.648230335 | 10.26966757 | -3.259231529 | 0.0051371 | 0.269705494 | -1.99329071 |
| FRAT1 | 0.615404822 | 8.771473202 | 3.257971937 | 0.005150519 | 0.269705494 | -1.995457572 |
| SERP2 | -0.844746473 | 3.946105776 | -3.25786897 | 0.005151618 | 0.269705494 | -1.995634703 |
| lnc-CA7-3 | 0.681888954 | 3.02037194 | 3.257284111 | 0.005157862 | 0.269705494 | -1.996640812 |
| MSI1 | 0.74726962 | 4.532494294 | 3.256820613 | 0.005162816 | 0.269705494 | -1.997438144 |
| TCP1 | -0.322883745 | 7.351305992 | -3.256637353 | 0.005164776 | 0.269705494 | -1.997753395 |
| LINC00310 | -0.45951135 | 1.700619296 | -3.256625296 | 0.005164905 | 0.269705494 | -1.997774135 |
| lnc-BCL2L11-2 | 0.856097861 | 4.768640883 | 3.256436931 | 0.00516692 | 0.269705494 | -1.998098168 |
| CYP4F8 | 0.675223166 | 5.812421722 | 3.25627707 | 0.005168631 | 0.269705494 | -1.998373164 |
| NOD2 | 0.811935444 | 9.233582826 | 3.256198813 | 0.005169469 | 0.269705494 | -1.998507784 |
| LRG1 | 1.304635914 | 9.237473392 | 3.256086913 | 0.005170667 | 0.269705494 | -1.998700277 |
| PRR12 | -0.294639037 | 7.953195981 | -3.255482959 | 0.005177139 | 0.269705494 | -1.999739206 |
| SPDL1 | -0.538154632 | 5.263803519 | -3.255319402 | 0.005178893 | 0.269705494 | -2.000020557 |
| GABPB1 | -0.432958271 | 5.924260778 | -3.255142247 | 0.005180794 | 0.269705494 | -2.000325298 |
| PTPRG-AS1 | 0.699809865 | 4.09786568 | 3.25426117 | 0.005190256 | 0.269705494 | -2.001840911 |
| RRP12 | 0.551632536 | 6.759648807 | 3.254152361 | 0.005191426 | 0.269705494 | -2.002028081 |
| lnc-TRPM1-1 | -0.814179097 | 1.9556155 | -3.253923477 | 0.005193888 | 0.269705494 | -2.002421799 |
| LOC649305 | 1.0523512 | 4.120877655 | 3.252733813 | 0.0052067 | 0.269705494 | -2.004468193 |
| SLC26A6 | 0.330233399 | 5.924320192 | 3.251661392 | 0.005218277 | 0.269705494 | -2.006312877 |
| KIF20A | -1.137644436 | 1.262087818 | -3.251083136 | 0.00522453 | 0.269705494 | -2.007307529 |
| LOC101929718 | -0.844517196 | 1.373086574 | -3.250643075 | 0.005229293 | 0.269705494 | -2.008064466 |
| HOXA4 | -0.804814004 | 2.099591967 | -3.250599504 | 0.005229765 | 0.269705494 | -2.008139411 |
| ELAC2 | -0.61323803 | 10.72758818 | -3.249302003 | 0.005243837 | 0.269705494 | -2.010371176 |
| lnc-RER1-1 | 0.898681728 | 7.775704332 | 3.249027976 | 0.005246813 | 0.269705494 | -2.010842509 |
| TEX10 | -0.443574033 | 8.260771679 | -3.249006279 | 0.005247049 | 0.269705494 | -2.01087983 |
| ARIH2 | -0.3217963 | 10.58049356 | -3.248576511 | 0.005251721 | 0.269705494 | -2.011619037 |
| lnc-C17orf63-3 | 0.674392561 | 4.682895249 | 3.248423292 | 0.005253387 | 0.269705494 | -2.011882574 |
| DTYMK | -0.342479532 | 9.092657767 | -3.247173295 | 0.005267004 | 0.269705494 | -2.014032552 |
| SUMF2 | -0.440645913 | 6.357096371 | -3.247025283 | 0.005268618 | 0.269705494 | -2.014287128 |
| lnc-NDFIP1-1 | -0.710977658 | 4.366938706 | -3.246847168 | 0.005270562 | 0.269705494 | -2.014593479 |
| EMD | -0.470786493 | 6.562283538 | -3.246462717 | 0.005274759 | 0.269705494 | -2.015254715 |
| LOC101928235 | 0.575018805 | 5.392837678 | 3.246438704 | 0.005275022 | 0.269705494 | -2.015296017 |
| SLPI | 1.34831381 | 7.782718265 | 3.246321142 | 0.005276306 | 0.269705494 | -2.015498218 |
| AADACL2-AS1 | 1.529591952 | 1.968388211 | 3.245916459 | 0.00528073 | 0.269705494 | -2.016194247 |
| SPATS2 | -0.553814295 | 6.027675954 | -3.242293473 | 0.005320495 | 0.270727201 | -2.022425338 |
| SLC25A10 | -1.155862244 | 1.654046427 | -3.241958489 | 0.005324186 | 0.270727201 | -2.02300145 |
| DET1 | -0.469038232 | 7.3354413 | -3.241467523 | 0.005329602 | 0.270727201 | -2.023845816 |
| P2RX1 | 0.951157094 | 7.096643631 | 3.241257944 | 0.005331915 | 0.270727201 | -2.024206249 |
| CUX2 | -1.544936337 | 3.202196969 | -3.239741358 | 0.005348684 | 0.27108401 | -2.026814427 |
| lnc-ZNF438-1 | 0.983380777 | 3.641724518 | 3.236713892 | 0.005382316 | 0.27108401 | -2.032020755 |
| LOC641746 | -0.405285353 | 6.352421826 | -3.236228853 | 0.005387724 | 0.27108401 | -2.032854848 |
| SASS6 | -0.845758672 | 3.090142055 | -3.236001736 | 0.005390258 | 0.27108401 | -2.033245407 |
| SCARA5 | -1.17814211 | 4.03763232 | -3.235336228 | 0.00539769 | 0.27108401 | -2.034389827 |
| LPAR2 | 0.970890339 | 7.27357745 | 3.235326894 | 0.005397794 | 0.27108401 | -2.034405878 |
| TLCD1 | 0.568131844 | 3.27814572 | 3.234090976 | 0.005411623 | 0.27108401 | -2.036531148 |
| DTL | -0.947920343 | 3.837662464 | -3.233771821 | 0.0054152 | 0.27108401 | -2.037079955 |
| KLHDC4 | -0.541799037 | 9.909106166 | -3.233753718 | 0.005415403 | 0.27108401 | -2.037111084 |
| lnc-AL391421.1-3 | 0.835081952 | 3.743304632 | 3.232548494 | 0.005428932 | 0.27108401 | -2.039183512 |
| CD96 | -0.555857625 | 8.928110735 | -3.232355536 | 0.005431101 | 0.27108401 | -2.039515307 |
| PRPF8 | -0.296476743 | 8.848874781 | -3.231698844 | 0.005438489 | 0.27108401 | -2.04064449 |
| XLOC_l2_005490 | 0.871058411 | 8.031650763 | 3.231096253 | 0.005445278 | 0.27108401 | -2.041680635 |
| TUBG1 | -0.42864347 | 8.066632945 | -3.230835441 | 0.005448219 | 0.27108401 | -2.042129092 |
| AIFM1 | -0.40296386 | 9.092928163 | -3.228811609 | 0.005471092 | 0.271452018 | -2.045608931 |
| TBC1D24 | -0.438041092 | 4.897146375 | -3.228147354 | 0.00547862 | 0.271452018 | -2.04675104 |
| NDUFS8 | -0.367207669 | 9.763291112 | -3.226253491 | 0.00550014 | 0.271452018 | -2.050007242 |
| XLOC_l2_000941 | 1.026215822 | 4.876179214 | 3.226194078 | 0.005500817 | 0.271452018 | -2.050109391 |
| MTHFSD | -0.541354062 | 5.356920885 | -3.226115353 | 0.005501713 | 0.271452018 | -2.050244743 |
| PACSIN1 | -0.963366408 | 7.666119462 | -3.225371739 | 0.005510188 | 0.271452018 | -2.051523235 |
| lnc-SLC25A19-1 | 0.846696332 | 3.485216715 | 3.223702231 | 0.005529262 | 0.271452018 | -2.054393542 |
| IARS | -0.534991794 | 8.302524924 | -3.223463372 | 0.005531997 | 0.271452018 | -2.054804194 |
| N4BP2 | -0.46258147 | 7.592507545 | -3.223174444 | 0.005535306 | 0.271452018 | -2.055300924 |
| RANBP3 | -0.245628253 | 10.08094232 | -3.221857388 | 0.005550416 | 0.271452018 | -2.057565187 |
| ZAP70 | -0.5562728 | 8.654283735 | -3.221484326 | 0.005554703 | 0.271452018 | -2.058206537 |
| EVI2B | 0.508292351 | 11.57064875 | 3.221022813 | 0.005560012 | 0.271452018 | -2.058999944 |
| FIP1L1 | -0.2368386 | 9.33387713 | -3.220898776 | 0.005561439 | 0.271452018 | -2.05921318 |
| LINC00649 | -0.704734154 | 8.038147404 | -3.220586072 | 0.00556504 | 0.271452018 | -2.059750757 |
| LOC101059954 | -0.966738799 | 5.422022796 | -3.218575597 | 0.005588244 | 0.272019912 | -2.063206931 |
| RUFY4 | 0.638063322 | 4.245291978 | 3.218213747 | 0.005592431 | 0.272019912 | -2.063828966 |
| lnc-C1QTNF8-4 | -0.450477541 | 4.368021984 | -3.21600404 | 0.005618064 | 0.272019912 | -2.067627443 |
| lnc-TCIRG1-1 | 0.694990095 | 8.364897411 | 3.215966742 | 0.005618498 | 0.272019912 | -2.067691558 |
| TRIM6 | 1.008280231 | 3.679423297 | 3.215267215 | 0.005626637 | 0.272019912 | -2.068894004 |
| TC2N | -0.66426778 | 7.452811337 | -3.214907775 | 0.005630824 | 0.272019912 | -2.069511855 |
| ZNF488 | 1.310174663 | 3.208828297 | 3.214498553 | 0.005635595 | 0.272019912 | -2.070215271 |
| XLOC_l2_015565 | 0.58838387 | 1.081390543 | 3.213777537 | 0.00564401 | 0.272019912 | -2.071454617 |
| SEMA4C | -0.642796774 | 9.227624868 | -3.213056987 | 0.005652432 | 0.272019912 | -2.072693146 |
| C12orf42 | -0.618152451 | 5.071025176 | -3.212430768 | 0.005659762 | 0.272019912 | -2.073769515 |
| ZBTB25 | -0.623660328 | 5.767601249 | -3.212168095 | 0.005662839 | 0.272019912 | -2.074221004 |
| NFIL3 | 0.76896156 | 10.47939114 | 3.2096247 | 0.005692721 | 0.272782051 | -2.078592519 |
| SGK223 | -0.654778019 | 10.29632276 | -3.209481025 | 0.005694414 | 0.272782051 | -2.078839457 |
| HIST2H3D | 0.443408719 | 10.92765271 | 3.207716648 | 0.005715241 | 0.273145009 | -2.081871868 |
| P3H4 | -0.508930702 | 6.593200983 | -3.207507036 | 0.00571772 | 0.273145009 | -2.082232118 |
| USP32P2 | 0.724437244 | 6.320687272 | 3.199777763 | 0.00580989 | 0.276274536 | -2.095514866 |
| PPCDC | 0.61508751 | 4.538373069 | 3.198662043 | 0.005823315 | 0.276274536 | -2.097432039 |
| CA5B | -0.585143978 | 7.649884839 | -3.198114483 | 0.005829915 | 0.276274536 | -2.098372909 |
| SAMD3 | -0.719258139 | 8.815551833 | -3.196965475 | 0.005843789 | 0.276274536 | -2.100347206 |
| lnc-KANK4-1 | 1.119109736 | 5.521758787 | 3.196725615 | 0.005846689 | 0.276274536 | -2.100759341 |
| lnc-C2-2 | 0.600652327 | 3.419604678 | 3.195367835 | 0.005863134 | 0.276274536 | -2.103092281 |
| EZR | -0.407265286 | 9.239546566 | -3.195030816 | 0.005867223 | 0.276274536 | -2.103671337 |
| DIAPH3 | -1.241996071 | 2.039141126 | -3.194675102 | 0.005871542 | 0.276274536 | -2.104282508 |
| TAF3 | -0.329457341 | 8.600486783 | -3.194281552 | 0.005876323 | 0.276274536 | -2.104958682 |
| lnc-IFNG-3 | -0.752741029 | 1.774650986 | -3.194251427 | 0.00587669 | 0.276274536 | -2.105010442 |
| NUMB | 0.513160152 | 8.774830013 | 3.193935353 | 0.005880533 | 0.276274536 | -2.105553496 |
| JMJD1C-AS1 | 1.203987822 | 2.679850257 | 3.19356899 | 0.005884991 | 0.276274536 | -2.106182948 |
| CASD1 | -0.634727209 | 5.113833221 | -3.193200255 | 0.005889481 | 0.276274536 | -2.10681647 |
| KCNJ14 | -0.432288581 | 5.412202529 | -3.192780325 | 0.005894599 | 0.276274536 | -2.107537942 |
| SH2D4B | 0.97174981 | 1.484060855 | 3.191927673 | 0.005905004 | 0.27638922 | -2.109002844 |
| ATOH8 | 1.090515711 | 3.580753507 | 3.190799574 | 0.005918799 | 0.27666202 | -2.110940934 |
| ADM | 1.287533306 | 10.46524327 | 3.188715696 | 0.005944364 | 0.277234295 | -2.114520926 |
| IL10RB-AS1 | 0.662027938 | 5.820806507 | 3.187830976 | 0.005955251 | 0.277234295 | -2.116040772 |
| LINC00282 | 1.205367304 | 5.80959724 | 3.186296469 | 0.005974181 | 0.277234295 | -2.118676803 |
| LINC00881 | 0.598999609 | 3.899047467 | 3.184656774 | 0.005994474 | 0.277234295 | -2.121493419 |
| MTFR1L | 0.251108056 | 6.261473448 | 3.184190363 | 0.006000259 | 0.277234295 | -2.122294585 |
| YPEL1 | -0.595823049 | 6.735408997 | -3.183016164 | 0.006014846 | 0.277234295 | -2.124311495 |
| lnc-C12orf42-3 | 1.109094177 | 2.823691005 | 3.182752084 | 0.006018132 | 0.277234295 | -2.124765094 |
| lnc-AC040934.1-3 | 0.836194979 | 5.255959916 | 3.18270904 | 0.006018668 | 0.277234295 | -2.124839028 |
| SNORD86 | -0.421764441 | 5.850088406 | -3.180717914 | 0.0060435 | 0.277234295 | -2.128259002 |
| DVL2 | -0.368681974 | 7.139197639 | -3.179225027 | 0.006062185 | 0.277234295 | -2.130823084 |
| GPI | -0.609494635 | 11.54910818 | -3.178873195 | 0.006066597 | 0.277234295 | -2.131427354 |
| DENND5A | 0.360449677 | 10.0744144 | 3.178062553 | 0.006076775 | 0.277234295 | -2.132819604 |
| SPRYD7 | -0.860880653 | 2.862804036 | -3.177887188 | 0.006078978 | 0.277234295 | -2.133120785 |
| AKR7L | -0.361792217 | 7.499664671 | -3.177855712 | 0.006079374 | 0.277234295 | -2.133174843 |
| lnc-CD53-1 | -1.351384632 | 3.828043551 | -3.176551516 | 0.00609579 | 0.277234295 | -2.135414685 |
| SOD2 | 0.729203389 | 12.52112148 | 3.17625282 | 0.006099556 | 0.277234295 | -2.135927659 |
| LOC100506014 | -0.478990841 | 3.294417026 | -3.175683023 | 0.006106746 | 0.277234295 | -2.136906205 |
| SLC35D1 | -0.673569763 | 3.51457602 | -3.174993594 | 0.006115457 | 0.277234295 | -2.138090181 |
| CELSR3-AS1 | 0.866174865 | 1.932196227 | 3.174788243 | 0.006118054 | 0.277234295 | -2.138442833 |
| RAB39B | -0.325541563 | 6.563219344 | -3.173766112 | 0.006130997 | 0.277234295 | -2.14019812 |
| LOC101929151 | 0.792300724 | 4.946559227 | 3.173349796 | 0.006136276 | 0.277234295 | -2.140913039 |
| DFFA | -0.26897038 | 7.007620432 | -3.172881611 | 0.006142219 | 0.277234295 | -2.14171702 |
| lnc-C21orf58-1 | -0.52046736 | 7.663910133 | -3.172804539 | 0.006143198 | 0.277234295 | -2.14184937 |
| PLG | -1.189663791 | 1.824765591 | -3.172285486 | 0.006149794 | 0.277234295 | -2.142740691 |
| LOC730338 | 0.438842782 | 6.580872835 | 3.172063219 | 0.00615262 | 0.277234295 | -2.143122365 |
| KIF22 | -0.510846126 | 6.818297928 | -3.171568007 | 0.006158923 | 0.277234295 | -2.143972728 |
| GPM6B | -0.800326286 | 3.276168013 | -3.169330084 | 0.006187483 | 0.277234295 | -2.14781549 |
| COL4A2-AS1 | 1.245683747 | 2.281938526 | 3.16776607 | 0.00620752 | 0.277234295 | -2.150500937 |
| ZFP2 | -1.160551638 | 2.221437325 | -3.165179323 | 0.006240802 | 0.277234295 | -2.154942197 |
| WDR24 | -0.262463273 | 8.081275628 | -3.16380001 | 0.00625862 | 0.277234295 | -2.15731025 |
| MRPL4 | -0.384561736 | 9.819753885 | -3.162397655 | 0.006276787 | 0.277234295 | -2.159717771 |
| LUCAT1 | 1.149705389 | 8.565678828 | 3.161956041 | 0.006282519 | 0.277234295 | -2.160475901 |
| VARS | -0.373718864 | 8.349206537 | -3.161816766 | 0.006284328 | 0.277234295 | -2.160714997 |
| lnc-TMEM189-UBE2V1-2 | -0.342116163 | 1.04593364 | -3.161544057 | 0.006287871 | 0.277234295 | -2.161183157 |
| LL22NC03-75H12.2 | -1.173202984 | 2.646590744 | -3.160258891 | 0.006304595 | 0.277234295 | -2.16338936 |
| SRMS | 0.712008869 | 7.822733291 | 3.15916316 | 0.006318889 | 0.277234295 | -2.165270301 |
| ZNF521 | -0.867651502 | 1.407984315 | -3.159001517 | 0.006321 | 0.277234295 | -2.165547776 |
| SOWAHD | 0.348900683 | 9.382805429 | 3.158843979 | 0.006323059 | 0.277234295 | -2.1658182 |
| IGF2BP3 | 0.715494428 | 7.462713568 | 3.158838693 | 0.006323128 | 0.277234295 | -2.165827274 |
| ATG16L2 | 0.68188564 | 13.226046 | 3.158705005 | 0.006324875 | 0.277234295 | -2.166056758 |
| ZNF45 | -0.528461536 | 4.843963479 | -3.158342271 | 0.006329618 | 0.277234295 | -2.16667941 |
| CD160 | -1.05891351 | 5.898007188 | -3.157317437 | 0.006343038 | 0.277234295 | -2.168438558 |
| SLC40A1 | 0.645358246 | 8.968252025 | 3.155854253 | 0.006362248 | 0.277234295 | -2.170950054 |
| SEPHS1 | -0.468118055 | 7.675425154 | -3.155631069 | 0.006365183 | 0.277234295 | -2.171333131 |
| ITGB7 | -0.644771611 | 11.88712971 | -3.155150133 | 0.006371512 | 0.277234295 | -2.172158612 |
| PPAN-P2RY11 | -0.528948366 | 4.189171219 | -3.154841116 | 0.006375582 | 0.277234295 | -2.172689003 |
| CECR6 | 0.952548117 | 8.279520928 | 3.154686841 | 0.006377615 | 0.277234295 | -2.172953796 |
| CTSS | 0.491193624 | 8.270070837 | 3.154659847 | 0.006377971 | 0.277234295 | -2.173000127 |
| ZNF416 | -0.44370012 | 5.884462914 | -3.154443182 | 0.006380827 | 0.277234295 | -2.173372003 |
| SETD1A | -0.339311222 | 9.205986318 | -3.15247631 | 0.006406814 | 0.277234295 | -2.176747759 |
| CEACAM3 | 0.871096422 | 6.569563129 | 3.151788491 | 0.006415926 | 0.277234295 | -2.177928223 |
| CD247 | -0.664191482 | 10.72908627 | -3.151481317 | 0.00642 | 0.277234295 | -2.178455399 |
| GCM1 | 0.864594715 | 4.327798631 | 3.151242935 | 0.006423163 | 0.277234295 | -2.178864512 |
| LY9 | -0.419915411 | 8.859135879 | -3.151166205 | 0.006424181 | 0.277234295 | -2.178996196 |
| IL1R1 | 1.459957756 | 4.219883015 | 3.150705121 | 0.006430305 | 0.277234295 | -2.1797875 |
| NANOG | -1.166136758 | 2.801570165 | -3.150036868 | 0.00643919 | 0.277234295 | -2.180934324 |
| ZNF664-FAM101A | 1.009142379 | 2.57622584 | 3.149906998 | 0.006440918 | 0.277234295 | -2.181157199 |
| HCST | -0.490295414 | 13.64944255 | -3.149278543 | 0.006449287 | 0.277234295 | -2.1822357 |
| AES | -0.582857414 | 12.35654556 | -3.148801436 | 0.006455648 | 0.277234295 | -2.183054458 |
| DHRS4-AS1 | -0.296480839 | 7.056268325 | -3.148740936 | 0.006456455 | 0.277234295 | -2.183158281 |
| AGTRAP | 0.616064391 | 7.793862909 | 3.148549272 | 0.006459012 | 0.277234295 | -2.18348719 |
| DCAF17 | -0.442652324 | 7.545750833 | -3.148007891 | 0.006466241 | 0.277234295 | -2.184416226 |
| ZNF256 | -0.635028273 | 6.191016084 | -3.146503577 | 0.006486369 | 0.277234295 | -2.186997626 |
| NFATC3 | -0.345222386 | 9.662787248 | -3.146440491 | 0.006487215 | 0.277234295 | -2.187105878 |
| SON | -0.266678059 | 7.505632869 | -3.146377772 | 0.006488056 | 0.277234295 | -2.187213501 |
| LOC102723471 | 0.958135394 | 2.647624583 | 3.145883553 | 0.006494684 | 0.277234295 | -2.188061552 |
| SIRT7 | 0.278206741 | 8.788478016 | 3.145733175 | 0.006496702 | 0.277234295 | -2.188319589 |
| lnc-COPS4-1 | -1.11465994 | 4.228823851 | -3.145526632 | 0.006499475 | 0.277234295 | -2.188674 |
| GRIPAP1 | 0.334460544 | 7.82498103 | 3.145512436 | 0.006499665 | 0.277234295 | -2.188698359 |
| LOC101929207 | 1.035716602 | 3.031892502 | 3.145266616 | 0.006502967 | 0.277234295 | -2.189120163 |
| FCGR2B | 0.980927923 | 9.498060674 | 3.145234756 | 0.006503395 | 0.277234295 | -2.189174831 |
| C1orf145 | -0.921324562 | 1.699513002 | -3.144978531 | 0.006506838 | 0.277234295 | -2.189614484 |
| SBK3 | 1.155419147 | 1.914154651 | 3.144463268 | 0.006513769 | 0.277234295 | -2.190498608 |
| LOC145945 | 0.769517483 | 1.348020512 | 3.142577864 | 0.006539189 | 0.277922842 | -2.1937336 |
| PRPS1 | -0.409188442 | 9.31347494 | -3.142077557 | 0.006545951 | 0.277922842 | -2.194592 |
| CSF3R | 0.646568524 | 14.8120038 | 3.139584844 | 0.006579745 | 0.277999776 | -2.198868671 |
| SIPA1L2 | 1.221078292 | 7.180997841 | 3.139392137 | 0.006582364 | 0.277999776 | -2.199199278 |
| lnc-RP11-293M10.1.1-1 | 0.625364449 | 4.128694801 | 3.138828195 | 0.006590037 | 0.277999776 | -2.200166765 |
| RPTOR | -0.439223888 | 8.018178277 | -3.137917579 | 0.006602444 | 0.277999776 | -2.201728965 |
| SNORA34 | 0.578984011 | 7.036006172 | 3.137056162 | 0.006614202 | 0.277999776 | -2.203206721 |
| NF2 | -0.399280152 | 10.25909536 | -3.13688558 | 0.006616533 | 0.277999776 | -2.203499349 |
| ZAK | 0.409415049 | 7.915415328 | 3.136839177 | 0.006617167 | 0.277999776 | -2.203578952 |
| STAT4 | -0.541706754 | 9.185383313 | -3.134911491 | 0.006643566 | 0.277999776 | -2.206885721 |
| SH3TC2 | 1.406673192 | 2.436070371 | 3.134300024 | 0.006651961 | 0.277999776 | -2.207934596 |
| DHCR7 | -0.345360687 | 7.769822245 | -3.133341238 | 0.006665146 | 0.277999776 | -2.209579202 |
| TRIM35 | -0.352170814 | 7.75852879 | -3.132873451 | 0.006671589 | 0.277999776 | -2.210381579 |
| EIF5B | -0.568839864 | 6.291192147 | -3.132851911 | 0.006671886 | 0.277999776 | -2.210418525 |
| F11-AS1 | 0.449522775 | 5.384803841 | 3.132632211 | 0.006674913 | 0.277999776 | -2.210795363 |
| TMC8 | -0.485263883 | 11.02899806 | -3.132102018 | 0.006682226 | 0.277999776 | -2.211704762 |
| DIXDC1 | -0.895830032 | 3.39892012 | -3.132081863 | 0.006682504 | 0.277999776 | -2.211739333 |
| SLC12A2 | -0.598835152 | 4.701628421 | -3.131565098 | 0.00668964 | 0.277999776 | -2.212625684 |
| POLRMT | -0.332229473 | 8.118448362 | -3.131540311 | 0.006689982 | 0.277999776 | -2.212668198 |
| XLOC_l2_008755 | 1.372051853 | 3.978170158 | 3.131175294 | 0.006695027 | 0.277999776 | -2.213294263 |
| lnc-ACOT1-3 | 1.150666572 | 4.693363818 | 3.130826571 | 0.006699851 | 0.277999776 | -2.213892376 |
| RITA1 | -0.301545695 | 7.078766665 | -3.129687097 | 0.006715635 | 0.278322193 | -2.215846696 |
| RRM2B | 0.469216167 | 5.725122291 | 3.12822861 | 0.006735892 | 0.278581602 | -2.218348056 |
| RPUSD2 | -0.452895554 | 8.722600853 | -3.127593774 | 0.006744728 | 0.278581602 | -2.219436786 |
| CHST12 | -0.594498785 | 9.577381609 | -3.12724206 | 0.006749628 | 0.278581602 | -2.22003996 |
| lnc-PLXND1-1 | 0.897817595 | 6.246622812 | 3.12692993 | 0.00675398 | 0.278581602 | -2.220575243 |
| FFAR2 | 0.986897753 | 8.773157365 | 3.125280422 | 0.006777023 | 0.278751683 | -2.223403951 |
| lnc-CMBL-1 | -0.295964074 | 4.308854356 | -3.124934458 | 0.006781866 | 0.278751683 | -2.22399722 |
| PDCD2L | -0.667177965 | 6.927046773 | -3.124529762 | 0.006787535 | 0.278751683 | -2.224691194 |
| lnc-INPPL1-1 | 0.516130245 | 3.609205848 | 3.124168184 | 0.006792604 | 0.278751683 | -2.225311222 |
| APCS | 1.143460681 | 1.842269321 | 3.123766926 | 0.006798234 | 0.278751683 | -2.225999285 |
| SEZ6L2 | 1.158051879 | 2.599952839 | 3.123149214 | 0.00680691 | 0.278778291 | -2.227058497 |
| LINC01270 | 1.273759916 | 6.09695113 | 3.12217246 | 0.006820651 | 0.278904878 | -2.228733325 |
| RGL3 | 1.181146433 | 2.396647866 | 3.121788353 | 0.006826062 | 0.278904878 | -2.229391935 |
| NABP2 | -0.562247754 | 4.123457681 | -3.120700079 | 0.006841417 | 0.279203756 | -2.231257901 |
| FAM209A | 0.537091574 | 8.127191824 | 3.11983362 | 0.006853666 | 0.279228182 | -2.232743492 |
| CPEB2 | 0.650866448 | 3.704489228 | 3.119520666 | 0.006858095 | 0.279228182 | -2.233280059 |
| GINS2 | -1.473508583 | 3.94633894 | -3.117756449 | 0.006883118 | 0.279264443 | -2.236304743 |
| UBIAD1 | -0.401500893 | 7.098060582 | -3.116993369 | 0.006893969 | 0.279264443 | -2.237612959 |
| LOC100996405 | 0.506778309 | 6.631799944 | 3.116723242 | 0.006897815 | 0.279264443 | -2.238076055 |
| LIMK2 | 0.872084746 | 10.9956107 | 3.116679776 | 0.006898434 | 0.279264443 | -2.238150571 |
| NAB2 | -0.457939798 | 8.156219236 | -3.116626618 | 0.006899191 | 0.279264443 | -2.238241702 |
| ANGPT1 | 0.913845712 | 4.234849929 | 3.116037134 | 0.006907591 | 0.279278974 | -2.239252273 |
| lnc-MGAT5B-1 | 0.76660608 | 3.335276387 | 3.114532872 | 0.006929073 | 0.27982176 | -2.241830984 |
| lnc-EMP3-1 | -0.516727043 | 3.727870093 | -3.113852994 | 0.006938804 | 0.279889274 | -2.242996437 |
| EIF4ENIF1 | -0.259252183 | 7.454954231 | -3.112803752 | 0.006953848 | 0.280170693 | -2.244795002 |
| LMO1 | 0.772617666 | 2.533702468 | 3.111407092 | 0.006973923 | 0.28040373 | -2.247189 |
| LDOC1 | -0.595471042 | 7.168327497 | -3.111248939 | 0.0069762 | 0.28040373 | -2.24746008 |
| TSSK4 | 1.004498884 | 2.408705682 | 3.109822766 | 0.006996764 | 0.28040373 | -2.249904532 |
| CCDC57 | -0.260722771 | 7.414440446 | -3.109721981 | 0.00699822 | 0.28040373 | -2.250077274 |
| AMOT | -0.941833865 | 3.31775338 | -3.109598678 | 0.007000001 | 0.28040373 | -2.250288607 |
| MON1A | -0.29138211 | 7.466884701 | -3.106863912 | 0.00703962 | 0.280670255 | -2.254975611 |
| TSPYL1 | -0.314787214 | 9.165182819 | -3.106503852 | 0.007044853 | 0.280670255 | -2.255592669 |
| MANF | -0.562546774 | 11.03874557 | -3.106351726 | 0.007047065 | 0.280670255 | -2.255853374 |
| PDE4D | -0.433839126 | 5.969455529 | -3.104767038 | 0.007070148 | 0.280670255 | -2.258569055 |
| CRYM | -1.096936529 | 4.398159486 | -3.104481657 | 0.007074312 | 0.280670255 | -2.259058096 |
| LAS1L | -0.413053485 | 9.309483721 | -3.104461941 | 0.0070746 | 0.280670255 | -2.259091881 |
| UBAC2-AS1 | -0.530934517 | 4.565779463 | -3.103454713 | 0.00708932 | 0.280670255 | -2.260817872 |
| UTP14C | -0.285307401 | 7.693244597 | -3.103237376 | 0.0070925 | 0.280670255 | -2.261190293 |
| OR10A2 | 0.633467567 | 1.184054395 | 3.103119077 | 0.007094231 | 0.280670255 | -2.261393006 |
| TCTN3 | -0.527193901 | 7.690070497 | -3.102198051 | 0.007107726 | 0.280670255 | -2.262971207 |
| GLUL | 0.498545314 | 9.460851604 | 3.102090728 | 0.0071093 | 0.280670255 | -2.263155104 |
| RUNX3 | -0.550028898 | 13.16529705 | -3.100542327 | 0.00713205 | 0.280670255 | -2.265808202 |
| B3GAT2 | -0.659911359 | 2.982864509 | -3.100470715 | 0.007133104 | 0.280670255 | -2.265930902 |
| ZNF347 | -0.653411567 | 4.663737726 | -3.100162037 | 0.007137648 | 0.280670255 | -2.266459787 |
| C14orf169 | -0.418708463 | 8.378392251 | -3.099841723 | 0.007142367 | 0.280670255 | -2.2670086 |
| ATIC | -0.497519601 | 7.564163788 | -3.099759359 | 0.007143581 | 0.280670255 | -2.26714972 |
| lnc-FAM27D1.1-3 | 0.72944532 | 1.902469591 | 3.099728213 | 0.00714404 | 0.280670255 | -2.267203083 |
| TCF4 | -0.611702038 | 9.343143249 | -3.098607823 | 0.007160573 | 0.281001923 | -2.269122662 |
| SPOPL | 0.418001924 | 7.906864029 | 3.097554829 | 0.007176146 | 0.281107714 | -2.2709267 |
| GATS | 0.505228376 | 9.04143985 | 3.096976661 | 0.007184711 | 0.281107714 | -2.271917215 |
| CXorf56 | -0.675515446 | 4.297162606 | -3.095958464 | 0.007199819 | 0.281107714 | -2.273661537 |
| TMEM235 | 1.007631474 | 3.229870109 | 3.094806828 | 0.007216944 | 0.281107714 | -2.275634381 |
| RTTN | -0.538534471 | 4.629361361 | -3.094350984 | 0.007223734 | 0.281107714 | -2.276415254 |
| NT5M | 0.970853399 | 9.208038022 | 3.093978741 | 0.007229283 | 0.281107714 | -2.277052909 |
| PTGDR | -0.906193977 | 7.450351373 | -3.093608662 | 0.007234804 | 0.281107714 | -2.277686847 |
| C4orf3 | 0.321260625 | 11.61917954 | 3.093568897 | 0.007235397 | 0.281107714 | -2.277754963 |
| SURF2 | -0.32488746 | 7.740933746 | -3.093190325 | 0.00724105 | 0.281107714 | -2.278403439 |
| lnc-WDR7-8 | 0.834275454 | 2.088306852 | 3.09297883 | 0.00724421 | 0.281107714 | -2.278765717 |
| CYFIP2 | -0.500562093 | 8.981018561 | -3.090454924 | 0.007282022 | 0.282083344 | -2.283088791 |
| CCDC71L | 0.570627276 | 8.913114861 | 3.089895949 | 0.007290423 | 0.282083344 | -2.284046178 |
| LOC100129617 | 0.751931549 | 3.913364041 | 3.089534232 | 0.007295864 | 0.282083344 | -2.284665699 |
| RFTN1 | -0.535560017 | 9.202585935 | -3.089137219 | 0.007301841 | 0.282083344 | -2.285345663 |
| DNAJC7 | -0.230522596 | 7.619696596 | -3.08651993 | 0.007341363 | 0.282251792 | -2.289828044 |
| NAGLU | -0.312120685 | 8.094830704 | -3.085870882 | 0.007351196 | 0.282251792 | -2.290939538 |
| PYHIN1 | -0.592415127 | 6.891721768 | -3.084838521 | 0.007366864 | 0.282251792 | -2.2927074 |
| GRM7-AS1 | 0.707965073 | 1.190611374 | 3.084805145 | 0.007367371 | 0.282251792 | -2.292764553 |
| CENPM | -0.823081279 | 7.629840345 | -3.084344813 | 0.007374368 | 0.282251792 | -2.293552824 |
| TLR4 | 0.864917233 | 9.824337533 | 3.08412006 | 0.007377787 | 0.282251792 | -2.293937685 |
| PDIA4 | -0.791086907 | 7.64662056 | -3.082796107 | 0.007397957 | 0.282251792 | -2.29620472 |
| USP29 | -0.466030136 | 1.173862267 | -3.08279566 | 0.007397964 | 0.282251792 | -2.296205485 |
| CCNL1 | 0.440551386 | 9.13495633 | 3.082232529 | 0.00740656 | 0.282251792 | -2.297169713 |
| XLOC_l2_009439 | 0.996577905 | 3.30714807 | 3.082052269 | 0.007409314 | 0.282251792 | -2.297478361 |
| ABHD14B | -0.389651053 | 7.537533624 | -3.082008853 | 0.007409977 | 0.282251792 | -2.297552699 |
| REXO4 | -0.491241839 | 9.63225346 | -3.081982337 | 0.007410382 | 0.282251792 | -2.297598101 |
| MDN1 | -0.437080305 | 4.28961594 | -3.081456211 | 0.007418426 | 0.282251792 | -2.298498939 |
| ZDHHC24 | -0.341675431 | 7.263967484 | -3.081354667 | 0.007419979 | 0.282251792 | -2.298672801 |
| lnc-BTBD19-1 | 1.86069558 | 2.429635265 | 3.080571598 | 0.00743197 | 0.2823986 | -2.300013541 |
| OSER1 | 0.294080647 | 8.778832459 | 3.079047185 | 0.007455367 | 0.282763128 | -2.302623465 |
| PPP1CC | -0.269283006 | 9.0385592 | -3.078786834 | 0.00745937 | 0.282763128 | -2.303069194 |
| GNG12-AS1 | 1.208449928 | 2.285792015 | 3.078356694 | 0.007465989 | 0.282763128 | -2.303805594 |
| TRIP13 | -1.344381511 | 3.421729733 | -3.077143184 | 0.007484692 | 0.283056035 | -2.305883061 |
| lnc-TMED3-1 | -0.761572312 | 1.455575939 | -3.076797843 | 0.007490023 | 0.283056035 | -2.306474251 |
| LOC400655 | 1.638751727 | 3.144243184 | 3.075258032 | 0.007513838 | 0.283421019 | -2.309110153 |
| IMP3 | -0.458822608 | 9.693749447 | -3.075118356 | 0.007516002 | 0.283421019 | -2.309349247 |
| SCARNA17 | -0.501532898 | 12.85814273 | -3.07445919 | 0.007526223 | 0.283498612 | -2.310477577 |
| OTUD3 | -0.603702504 | 5.587177595 | -3.073738414 | 0.007537414 | 0.28361257 | -2.311711334 |
| POLR3E | -0.404276378 | 7.535425321 | -3.072654592 | 0.007554274 | 0.283718731 | -2.313566453 |
| lnc-CCDC83-1 | 0.985696836 | 4.341757977 | 3.072506884 | 0.007556574 | 0.283718731 | -2.31381927 |
| ZNF780B | -0.583290678 | 3.981149306 | -3.071979703 | 0.007564791 | 0.2837205 | -2.314721585 |
| ZBTB34 | 0.406866901 | 8.165247464 | 3.069673568 | 0.007600837 | 0.284764895 | -2.318668503 |
| PTDSS1 | -0.412883195 | 11.02597227 | -3.069031701 | 0.007610899 | 0.284834628 | -2.319766984 |
| SLC16A3 | 0.701459526 | 13.11836742 | 3.0680953 | 0.007625603 | 0.285077711 | -2.321369477 |
| PDCD4 | -0.271651291 | 8.775053243 | -3.067358922 | 0.007637186 | 0.285203713 | -2.322629623 |
| ANKRD34A | -0.998294235 | 1.97957646 | -3.065992008 | 0.007658732 | 0.285701124 | -2.32496869 |
| IPO4 | -0.475943452 | 5.652403934 | -3.06490378 | 0.007675928 | 0.286035367 | -2.326830778 |
| lnc-TTC21A-2 | 0.894460096 | 1.702574117 | 3.063805613 | 0.007693319 | 0.286072523 | -2.328709788 |
| SF3B3 | -0.509194653 | 7.886830599 | -3.06364399 | 0.007695882 | 0.286072523 | -2.328986325 |
| THAP4 | -0.276021417 | 7.958394437 | -3.061505489 | 0.007729871 | 0.286072523 | -2.332645128 |
| lnc-C14orf57-1 | 1.018256285 | 2.088253309 | 3.06122739 | 0.007734302 | 0.286072523 | -2.33312091 |
| COL7A1 | 0.530834054 | 3.995692982 | 3.061169876 | 0.007735219 | 0.286072523 | -2.333219304 |
| GABARAPL2 | 0.275969109 | 8.940802969 | 3.059667968 | 0.007759195 | 0.286072523 | -2.335788723 |
| lnc-DARS2-1 | -0.633956607 | 1.910507136 | -3.059479727 | 0.007762205 | 0.286072523 | -2.336110748 |
| SLC33A1 | -0.347893362 | 4.585081936 | -3.059433597 | 0.007762943 | 0.286072523 | -2.336189662 |
| lnc-PRPS1-1 | 0.783443654 | 4.043453762 | 3.059379973 | 0.007763801 | 0.286072523 | -2.336281396 |
| GPR173 | 0.900042917 | 1.63982939 | 3.058731203 | 0.007774186 | 0.286072523 | -2.33739123 |
| lnc-AC090186.1-3 | 0.62024804 | 3.543141879 | 3.058292334 | 0.007781219 | 0.286072523 | -2.338141975 |
| UFSP1 | -0.31052268 | 4.358620132 | -3.057783742 | 0.007789378 | 0.286072523 | -2.339011973 |
| ZNF584 | -0.563150914 | 4.340534128 | -3.057103752 | 0.007800298 | 0.286072523 | -2.340175136 |
| C5AR2 | 0.787990881 | 4.902994608 | 3.057102642 | 0.007800316 | 0.286072523 | -2.340177034 |
| LOC100129216 | -0.578255851 | 2.188507782 | -3.057092428 | 0.00780048 | 0.286072523 | -2.340194506 |
| EFTUD2 | -0.339146741 | 10.51007557 | -3.055442734 | 0.007827038 | 0.286545342 | -2.343016267 |
| TRUB2 | -0.43102324 | 6.4751865 | -3.055266872 | 0.007829874 | 0.286545342 | -2.343317064 |
| PFN1P2 | -0.302986613 | 10.77686039 | -3.053337016 | 0.007861066 | 0.287075337 | -2.346617764 |
| lnc-VMO1-1 | 0.564948634 | 4.210169322 | 3.052942062 | 0.007867464 | 0.287075337 | -2.347293235 |
| PEX14 | -0.422613422 | 9.523534679 | -3.052837817 | 0.007869154 | 0.287075337 | -2.347471518 |
| TSFM | -0.416140612 | 8.65925338 | -3.051908228 | 0.007884238 | 0.287323787 | -2.349061299 |
| XLOC_l2_002433 | -0.858486329 | 1.868108333 | -3.051216124 | 0.007895486 | 0.287432106 | -2.350244892 |
| CARD6 | 0.514611134 | 9.091745634 | 3.049490016 | 0.007923609 | 0.288153847 | -2.353196625 |
| lnc-ICOSLG-6 | -0.576049744 | 5.045268896 | -3.048425678 | 0.007940998 | 0.288484171 | -2.35501659 |
| HEATR1 | -0.50386741 | 7.866107006 | -3.047643154 | 0.007953807 | 0.288647576 | -2.356354613 |
| CCNC | -0.567879829 | 6.495949794 | -3.047093479 | 0.007962817 | 0.288672903 | -2.357294467 |
| CD7 | -0.582472629 | 7.978886644 | -3.046416256 | 0.007973931 | 0.28877439 | -2.358452375 |
| RP2 | 0.477462773 | 9.657706131 | 3.04362148 | 0.008019959 | 0.289299721 | -2.363230493 |
| BZRAP1-AS1 | -0.498232144 | 9.015347496 | -3.043391509 | 0.008023758 | 0.289299721 | -2.36362364 |
| LBH | -0.590535413 | 9.651681439 | -3.042819877 | 0.008033209 | 0.289299721 | -2.364600857 |
| ARHGAP27 | 0.357695439 | 13.00362869 | 3.042818847 | 0.008033226 | 0.289299721 | -2.364602617 |
| SLC25A42 | -0.526258147 | 6.222216453 | -3.042757557 | 0.00803424 | 0.289299721 | -2.364707393 |
| LINC00029 | 1.165817974 | 3.849341734 | 3.041257955 | 0.008059089 | 0.289299721 | -2.367270868 |
| lnc-NLRP12-1 | -0.734473083 | 1.274782075 | -3.040979502 | 0.008063711 | 0.289299721 | -2.367746849 |
| HSPA13 | -0.676318785 | 6.987707429 | -3.039150417 | 0.008094138 | 0.289299721 | -2.370873291 |
| GRB2 | 0.379510962 | 9.11002248 | 3.038626226 | 0.008102879 | 0.289299721 | -2.371769241 |
| BANF1 | -0.310289958 | 7.601534874 | -3.0378932 | 0.008115117 | 0.289299721 | -2.373022098 |
| XLOC_l2_003856 | 1.16822944 | 2.907141562 | 3.037797789 | 0.008116712 | 0.289299721 | -2.373185167 |
| ANAPC5 | -0.297418024 | 11.45206106 | -3.03734355 | 0.008124306 | 0.289299721 | -2.373961508 |
| PARP16 | -0.427322365 | 6.136158079 | -3.037063508 | 0.008128992 | 0.289299721 | -2.374440123 |
| CCDC117 | -0.454193863 | 7.555106499 | -3.036717577 | 0.008134784 | 0.289299721 | -2.375031338 |
| KCTD1 | -0.46934198 | 5.525251603 | -3.036298037 | 0.008141813 | 0.289299721 | -2.375748343 |
| C6orf226 | -0.377907896 | 8.585708588 | -3.035980573 | 0.008147136 | 0.289299721 | -2.376290887 |
| CNPY4 | -0.488455522 | 4.892915782 | -3.035576913 | 0.00815391 | 0.289299721 | -2.376980729 |
| C21orf62-AS1 | -0.672508371 | 3.576052415 | -3.03470252 | 0.008168601 | 0.289299721 | -2.378474998 |
| LOC102724050 | 1.14234885 | 2.343902768 | 3.034674172 | 0.008169078 | 0.289299721 | -2.378523442 |
| PKDREJ | -1.023406194 | 1.364713645 | -3.03445031 | 0.008172843 | 0.289299721 | -2.378905995 |
| C22orf29 | -0.37450929 | 8.59285196 | -3.034255469 | 0.008176122 | 0.289299721 | -2.379238951 |
| TMEM55A | 0.501959381 | 8.899204491 | 3.033768262 | 0.008184327 | 0.289299721 | -2.380071507 |
| lnc-RAB1A-2 | -0.820329466 | 1.613805646 | -3.033532369 | 0.008188302 | 0.289299721 | -2.380474603 |
| ZNF425 | -0.41356536 | 4.554150363 | -3.032809165 | 0.008200502 | 0.289299721 | -2.381710394 |
| LOC100507391 | -1.168833933 | 1.832970705 | -3.032465962 | 0.008206297 | 0.289299721 | -2.382296835 |
| BCL7B | -0.273771346 | 7.129525029 | -3.032045983 | 0.008213395 | 0.289299721 | -2.383014453 |
| LOC100996425 | 0.546198065 | 4.812701836 | 3.032003146 | 0.008214119 | 0.289299721 | -2.383087647 |
| lnc-SDSL-4 | 0.396751703 | 3.617862415 | 3.031153921 | 0.008228491 | 0.289299721 | -2.384538676 |
| LMAN1 | -0.771788495 | 2.138007976 | -3.030827093 | 0.008234028 | 0.289299721 | -2.385097095 |
| ST8SIA4 | 0.37300226 | 9.638901419 | 3.02973266 | 0.008252598 | 0.289299721 | -2.386966987 |
| ATP9B | -0.405727478 | 7.025497309 | -3.029082984 | 0.008263641 | 0.289299721 | -2.388076946 |
| LOC101928448 | 1.090892278 | 2.941087202 | 3.028798368 | 0.008268483 | 0.289299721 | -2.388563197 |
| TNRC6A | -0.368917546 | 8.1585845 | -3.028669992 | 0.008270668 | 0.289299721 | -2.388782519 |
| C11orf49 | -0.329449694 | 6.271599284 | -3.028611951 | 0.008271656 | 0.289299721 | -2.388881678 |
| TTC23 | -0.690610384 | 1.152016827 | -3.026683241 | 0.008304556 | 0.290158191 | -2.392176579 |
| ACSL4 | 0.61252416 | 6.136716635 | 3.025271368 | 0.008328722 | 0.290236983 | -2.394588361 |
| lnc-SMCR5-2 | -0.894650772 | 1.597636945 | -3.025086219 | 0.008331896 | 0.290236983 | -2.394904623 |
| NT5C2 | 0.711404832 | 8.293690135 | 3.02499095 | 0.008333529 | 0.290236983 | -2.395067356 |
| FASN | -0.370093237 | 7.082090357 | -3.024446499 | 0.008342872 | 0.290236983 | -2.395997341 |
| LOC101927522 | 1.240757489 | 5.198832886 | 3.024112872 | 0.008348602 | 0.290236983 | -2.396567204 |
| GPLD1 | -0.874611865 | 1.738844739 | -3.023174421 | 0.00836474 | 0.290236983 | -2.398170107 |
| NCAPG2 | -1.121399095 | 2.687763861 | -3.022847653 | 0.008370366 | 0.290236983 | -2.398728221 |
| GPA33 | -0.806252206 | 7.447153035 | -3.022362414 | 0.008378728 | 0.290236983 | -2.399556985 |
| APOBR | 0.535999028 | 9.868413874 | 3.022171293 | 0.008382024 | 0.290236983 | -2.399883405 |
| ALOX5 | 0.724350258 | 12.95001371 | 3.02141767 | 0.008395032 | 0.290252719 | -2.401170506 |
| TRAF4 | -0.314767131 | 5.62809207 | -3.020862041 | 0.008404636 | 0.290252719 | -2.402119428 |
| USF1 | 0.860866843 | 3.936924279 | 3.020693504 | 0.008407551 | 0.290252719 | -2.402407257 |
| lnc-ARRDC3-1 | 0.992615266 | 10.53305036 | 3.020036176 | 0.00841893 | 0.290356926 | -2.403529822 |
| BAHD1 | -0.382184659 | 9.101724555 | -3.019109716 | 0.008434993 | 0.290378381 | -2.405111946 |
| CENPF | -0.834433084 | 6.288499184 | -3.019035928 | 0.008436274 | 0.290378381 | -2.405237951 |
| THEM6 | -0.457943815 | 7.134101362 | -3.016166279 | 0.008486228 | 0.290752266 | -2.410138024 |
| LAX1 | -0.519903378 | 6.567397808 | -3.01539407 | 0.00849972 | 0.290752266 | -2.411456496 |
| ODF2 | -0.309979114 | 7.145784256 | -3.015319093 | 0.008501031 | 0.290752266 | -2.41158451 |
| MRPL49 | -0.369038435 | 8.29526173 | -3.014684274 | 0.00851214 | 0.290752266 | -2.412668363 |
| FNDC3B | 0.570485802 | 8.846551507 | 3.01430356 | 0.008518809 | 0.290752266 | -2.413318356 |
| SMIM3 | 0.503874083 | 8.574984771 | 3.013845445 | 0.008526841 | 0.290752266 | -2.414100481 |
| PHB2 | -0.442187018 | 10.36440148 | -3.013018473 | 0.008541358 | 0.290752266 | -2.4155123 |
| C15orf59 | 0.475044126 | 5.305074756 | 3.012623213 | 0.008548305 | 0.290752266 | -2.416187074 |
| ATP10A | -0.592334995 | 8.472948478 | -3.01200652 | 0.008559156 | 0.290752266 | -2.417239844 |
| XLOC_l2_014423 | 1.045367613 | 2.655407138 | 3.011920547 | 0.008560669 | 0.290752266 | -2.417386609 |
| VMP1 | 0.599501546 | 11.41048587 | 3.011895647 | 0.008561108 | 0.290752266 | -2.417429115 |
| UFSP2 | -0.566294052 | 6.902377741 | -3.011857096 | 0.008561787 | 0.290752266 | -2.417494926 |
| KDM1B | 0.341474945 | 4.02418198 | 3.011424032 | 0.008569416 | 0.290752266 | -2.418234195 |
| URGCP | -0.562418693 | 5.664008111 | -3.011405903 | 0.008569736 | 0.290752266 | -2.418265141 |
| KCTD16 | -0.578418836 | 1.204434497 | -3.010536739 | 0.00858507 | 0.290752266 | -2.419748818 |
| FBXO16 | -1.122380534 | 1.396013207 | -3.010460978 | 0.008586408 | 0.290752266 | -2.419878139 |
| SNAR-B1 | 0.458828725 | 1.170422739 | 3.010008753 | 0.008594398 | 0.290752266 | -2.420650067 |
| LINC00906 | 1.017996455 | 1.325080738 | 3.00981473 | 0.008597828 | 0.290752266 | -2.42098125 |
| ZNF579 | -0.231235356 | 8.635930696 | -3.008601326 | 0.008619312 | 0.290761275 | -2.423052372 |
| CDCA4 | -0.779832727 | 2.72415139 | -3.008473735 | 0.008621574 | 0.290761275 | -2.423270145 |
| MS4A6A | 0.370449788 | 9.635635301 | 3.007867049 | 0.008632338 | 0.290761275 | -2.424305628 |
| MYD88 | 0.449504518 | 10.05688789 | 3.007310722 | 0.00864222 | 0.290761275 | -2.425255131 |
| ZNF528 | -1.143472881 | 2.586466199 | -3.006793872 | 0.008651411 | 0.290761275 | -2.426137234 |
| lnc-SLC7A11-1 | 1.142263348 | 2.63627223 | 3.006626462 | 0.00865439 | 0.290761275 | -2.426422947 |
| HMP19 | 0.998120305 | 1.671181481 | 3.006496714 | 0.008656699 | 0.290761275 | -2.426644382 |
| LOC401188 | 1.028662736 | 3.195458045 | 3.004905488 | 0.008685072 | 0.291104345 | -2.429359936 |
| EXOC3L4 | 0.943296201 | 1.408475232 | 3.004678796 | 0.008689121 | 0.291104345 | -2.429746787 |
| lnc-FRG1-5 | 0.95451336 | 5.521387405 | 3.004514417 | 0.008692059 | 0.291104345 | -2.430027296 |
| VPS51 | -0.313850306 | 10.0146944 | -3.003991537 | 0.00870141 | 0.291136757 | -2.43091957 |
| HCK | 0.712112719 | 9.122975402 | 3.002324731 | 0.008731283 | 0.291460505 | -2.433763755 |
| SLC25A23 | -0.562273222 | 6.512429617 | -3.002211518 | 0.008733316 | 0.291460505 | -2.433956929 |
| HMMR | -1.501854694 | 3.991308457 | -3.001844247 | 0.008739913 | 0.291460505 | -2.434583593 |
| ATP6V1E1 | 0.276836226 | 6.441908776 | 3.001580465 | 0.008744654 | 0.291460505 | -2.435033671 |
| lnc-ARFIP1-2 | 0.754967287 | 1.07573774 | 2.999732628 | 0.00877794 | 0.291768791 | -2.43818637 |
| DDX39A | -0.413873911 | 10.48101246 | -2.999369653 | 0.008784493 | 0.291768791 | -2.43880563 |
| EVI2A | 0.526027197 | 9.280221871 | 2.999302069 | 0.008785713 | 0.291768791 | -2.438920931 |
| TMEM41A | -0.355309982 | 4.720255134 | -2.999202705 | 0.008787508 | 0.291768791 | -2.439090451 |
| FLJ41733 | 0.488219833 | 1.289651506 | 2.998232203 | 0.008805058 | 0.292072276 | -2.440746119 |
| OSBPL11 | 0.29504951 | 8.625503172 | 2.996557424 | 0.008835425 | 0.292745619 | -2.443603094 |
| PRKCD | 0.482490136 | 9.635884925 | 2.995602382 | 0.008852788 | 0.292745619 | -2.445232176 |
| ABHD15 | -0.712923355 | 5.342169776 | -2.994966679 | 0.008864363 | 0.292745619 | -2.446316494 |
| LOC101927187 | -0.54996429 | 2.768765259 | -2.994823423 | 0.008866974 | 0.292745619 | -2.446560841 |
| ZGLP1 | 0.313453798 | 7.770384678 | 2.994195381 | 0.008878428 | 0.292745619 | -2.447632052 |
| lnc-DHRS9-1 | 1.014325055 | 1.849721593 | 2.994165784 | 0.008878968 | 0.292745619 | -2.447682533 |
| CROCC | -0.290679759 | 8.089841594 | -2.993132931 | 0.008897838 | 0.292745619 | -2.449444127 |
| METTL16 | -0.437523504 | 9.157400708 | -2.992311996 | 0.008912864 | 0.292745619 | -2.450844216 |
| lnc-RGL4-1 | -0.778233581 | 3.209668623 | -2.992022508 | 0.008918168 | 0.292745619 | -2.451337919 |
| TPM2 | -0.692394904 | 7.849462409 | -2.991557675 | 0.008926692 | 0.292745619 | -2.452130645 |
| OR52K2 | 0.779707143 | 6.534355127 | 2.991248571 | 0.008932365 | 0.292745619 | -2.452657781 |
| RTN2 | 0.744314994 | 5.043072233 | 2.990874562 | 0.008939233 | 0.292745619 | -2.453295592 |
| GPR126 | -0.928061001 | 1.671950517 | -2.990545977 | 0.008945272 | 0.292745619 | -2.453855931 |
| FCGR2A | 0.779403589 | 11.40414427 | 2.990204453 | 0.008951553 | 0.292745619 | -2.454438324 |
| LRRK2 | 0.554023516 | 9.236690897 | 2.989948923 | 0.008956255 | 0.292745619 | -2.454874067 |
| CDKN2D | 0.396175854 | 12.9196515 | 2.988735912 | 0.008978609 | 0.292745619 | -2.456942479 |
| NFE4 | 1.271172161 | 3.606321517 | 2.988573567 | 0.008981605 | 0.292745619 | -2.457219299 |
| lnc-MORF4L2-1 | 0.456200461 | 1.004388829 | 2.987126924 | 0.009008344 | 0.292745619 | -2.45968591 |
| REM2 | 0.761590021 | 9.161203633 | 2.986337038 | 0.009022977 | 0.292745619 | -2.461032634 |
| ADAMTSL4 | 0.536634232 | 5.160958091 | 2.986133056 | 0.00902676 | 0.292745619 | -2.461380407 |
| C20orf24 | 0.325570294 | 11.5519165 | 2.985281793 | 0.009042563 | 0.292745619 | -2.4628317 |
| MCCC2 | -0.589643318 | 4.074910826 | -2.984718931 | 0.009053027 | 0.292745619 | -2.463791272 |
| HMGN2 | -0.32991879 | 13.76753193 | -2.984551533 | 0.009056141 | 0.292745619 | -2.464076649 |
| lnc-GAS2L2-1 | 0.335582059 | 4.584826356 | 2.984502282 | 0.009057057 | 0.292745619 | -2.464160609 |
| LOC101927686 | 0.841744394 | 8.060590869 | 2.984371123 | 0.009059498 | 0.292745619 | -2.464384205 |
| BUB1B | -1.075409027 | 3.145012358 | -2.984090247 | 0.009064728 | 0.292745619 | -2.464863024 |
| FBLN2 | -1.348497283 | 3.471926459 | -2.982746481 | 0.009089789 | 0.292745619 | -2.4671537 |
| RALB | 0.573915531 | 11.05572047 | 2.981738649 | 0.009108629 | 0.292745619 | -2.468871616 |
| NCF1 | 0.671712852 | 13.38520427 | 2.98077439 | 0.00912669 | 0.292745619 | -2.470515173 |
| LRRC25 | 0.589530331 | 9.993967402 | 2.979863961 | 0.009143776 | 0.292745619 | -2.472066901 |
| lnc-MDGA2-2 | 1.14623091 | 3.054383709 | 2.978713443 | 0.009165411 | 0.292745619 | -2.474027731 |
| SCAPER | -0.583684511 | 6.142674887 | -2.978456597 | 0.009170248 | 0.292745619 | -2.474465459 |
| ZNF502 | -0.581712405 | 6.476662655 | -2.978346667 | 0.009172319 | 0.292745619 | -2.474652803 |
| ZCCHC6 | 0.495769574 | 8.016564563 | 2.977189113 | 0.009194154 | 0.292745619 | -2.476625467 |
| FAM160B1 | 0.572879044 | 8.181596393 | 2.976819392 | 0.009201139 | 0.292745619 | -2.477255507 |
| lnc-PHOX2B-2 | 0.666573307 | 1.145995733 | 2.976619651 | 0.009204914 | 0.292745619 | -2.477595879 |
| SNN | 0.475315002 | 9.573668079 | 2.976573346 | 0.00920579 | 0.292745619 | -2.477674787 |
| ATP6V1B2 | 0.446963987 | 10.05677524 | 2.976471295 | 0.00920772 | 0.292745619 | -2.477848686 |
| EIF4A1 | -0.308209051 | 10.33115219 | -2.976408636 | 0.009208905 | 0.292745619 | -2.47795546 |
| ELL | 0.62595437 | 7.492281934 | 2.976136281 | 0.009214058 | 0.292745619 | -2.478419562 |
| RNF125 | -0.561525847 | 9.558847319 | -2.975136219 | 0.009233003 | 0.292745619 | -2.48012364 |
| C11orf63 | -0.775543659 | 3.837834828 | -2.974877253 | 0.009237915 | 0.292745619 | -2.480564897 |
| C10orf105 | 0.966902714 | 7.538695689 | 2.974521888 | 0.009244659 | 0.292745619 | -2.4811704 |
| MYADML2 | 1.219920368 | 2.104863446 | 2.973491733 | 0.009264239 | 0.292745619 | -2.482925604 |
| HLCS | -0.623358261 | 6.982082348 | -2.973284982 | 0.009268173 | 0.292745619 | -2.48327786 |
| F5 | 0.613980735 | 5.719430092 | 2.973162851 | 0.009270498 | 0.292745619 | -2.483485941 |
| lnc-C2orf54-2 | 0.656868966 | 1.130015754 | 2.972351546 | 0.009285956 | 0.292745619 | -2.484868171 |
| lnc-NIPSNAP3B-1 | 0.824664984 | 4.557463342 | 2.972228151 | 0.00928831 | 0.292745619 | -2.485078394 |
| lnc-TCTN3-1 | 0.523547942 | 1.083483712 | 2.971414894 | 0.009303835 | 0.292745619 | -2.486463881 |
| ZNF438 | 0.781182104 | 9.023278076 | 2.971150938 | 0.009308879 | 0.292745619 | -2.486913551 |
| LOC93444 | 1.305571163 | 5.513040056 | 2.97109674 | 0.009309915 | 0.292745619 | -2.487005881 |
| lnc-PIK3R1-1 | -0.897386259 | 1.995543353 | -2.970404232 | 0.009323164 | 0.292745619 | -2.488185588 |
| KIF4A | -0.890582332 | 1.684825275 | -2.970156916 | 0.009327899 | 0.292745619 | -2.488606887 |
| SLC35F2 | -0.552152171 | 5.890486843 | -2.970094007 | 0.009329104 | 0.292745619 | -2.488714051 |
| lnc-APOA1-1 | 0.558844112 | 6.164307628 | 2.969801279 | 0.009334714 | 0.292745619 | -2.489212699 |
| MBD6 | 0.692599192 | 9.129405185 | 2.969147417 | 0.009347255 | 0.292745619 | -2.490326496 |
| PANX2 | 1.335209487 | 3.512851665 | 2.968713467 | 0.009355587 | 0.292745619 | -2.491065669 |
| RACGAP1 | -0.490990183 | 5.302697617 | -2.968684058 | 0.009356152 | 0.292745619 | -2.491115763 |
| NPEPL1 | 0.47216888 | 10.78489019 | 2.967868708 | 0.009371828 | 0.292745619 | -2.49250455 |
| MYOM2 | -3.290562835 | 7.989512579 | -2.967820047 | 0.009372765 | 0.292745619 | -2.492587432 |
| PA2G4 | -0.320148594 | 10.91235366 | -2.967658772 | 0.009375869 | 0.292745619 | -2.492862125 |
| ZNF668 | -0.215986627 | 8.831569303 | -2.967313086 | 0.009382526 | 0.292745619 | -2.493450907 |
| RTN4R | -0.605101157 | 5.479597466 | -2.967003178 | 0.009388497 | 0.292745619 | -2.493978742 |
| CLEC4A | 0.313202263 | 10.03516743 | 2.966994933 | 0.009388656 | 0.292745619 | -2.493992784 |
| FAM186B | 0.777260332 | 4.336335224 | 2.966272803 | 0.009402586 | 0.292745619 | -2.49522268 |
| FAM60A | -0.34719155 | 9.920356558 | -2.965406428 | 0.009419325 | 0.292745619 | -2.496698182 |
| RIMS3 | -0.618404438 | 5.483109906 | -2.963986281 | 0.009446827 | 0.292745619 | -2.499116651 |
| CIR1 | 0.38238171 | 8.462364661 | 2.963982418 | 0.009446901 | 0.292745619 | -2.499123228 |
| lnc-USP28-1 | -0.45100839 | 4.705654624 | -2.963120683 | 0.009463628 | 0.292745619 | -2.500590646 |
| LOC100130542 | 0.962116945 | 2.074274654 | 2.96249328 | 0.009475823 | 0.292745619 | -2.501658984 |
| SPOCK2 | -0.530122669 | 12.4192351 | -2.96244139 | 0.009476833 | 0.292745619 | -2.50174734 |
| LOC101927571 | -0.69114351 | 1.57324796 | -2.962422005 | 0.00947721 | 0.292745619 | -2.501780348 |
| SNHG4 | -0.82110938 | 2.147199204 | -2.96232607 | 0.009479076 | 0.292745619 | -2.501943701 |
| SSRP1 | -0.487936249 | 10.76063418 | -2.962311597 | 0.009479358 | 0.292745619 | -2.501968344 |
| KCNJ2 | 0.752709169 | 10.3101544 | 2.961915611 | 0.009487066 | 0.292745619 | -2.502642602 |
| KIAA0825 | 0.803635883 | 4.647933595 | 2.961501755 | 0.009495129 | 0.292745619 | -2.50334727 |
| ADAMTS17 | -1.015164485 | 2.171872158 | -2.961453737 | 0.009496065 | 0.292745619 | -2.503429029 |
| AIM2 | 1.001271034 | 8.815704877 | 2.960525785 | 0.009514169 | 0.292745619 | -2.505008982 |
| ZNRF3 | -0.685944815 | 4.590586677 | -2.960129597 | 0.009521909 | 0.292745619 | -2.505683517 |
| TLDC1 | -0.259829572 | 7.490791654 | -2.959874176 | 0.009526902 | 0.292745619 | -2.506118379 |
| STAB1 | 0.567850294 | 7.020738067 | 2.958697776 | 0.009549932 | 0.292745619 | -2.508121156 |
| lnc-NPIPL2-2 | 0.914587791 | 1.46881318 | 2.958379604 | 0.00955617 | 0.292745619 | -2.508662809 |
| KRTCAP2 | -0.352519348 | 10.93742789 | -2.957693085 | 0.009569644 | 0.292745619 | -2.509831502 |
| ZNF549 | -1.159894376 | 3.92076661 | -2.957552202 | 0.009572411 | 0.292745619 | -2.510071329 |
| EPHX4 | -1.38236615 | 2.625580422 | -2.957006954 | 0.009583129 | 0.292745619 | -2.510999489 |
| EPS8L2 | -0.916064658 | 3.670757722 | -2.956463124 | 0.00959383 | 0.292745619 | -2.511925208 |
| GAB2 | 0.766694806 | 9.724625129 | 2.956292219 | 0.009597195 | 0.292745619 | -2.51221612 |
| ATP6V0E1 | 0.36142884 | 10.44533762 | 2.956056453 | 0.00960184 | 0.292745619 | -2.512617434 |
| lnc-AC012313.1-1 | -0.743003246 | 1.586813276 | -2.955945647 | 0.009604023 | 0.292745619 | -2.512806043 |
| FAM126A | -0.774799195 | 4.233256553 | -2.955017496 | 0.009622333 | 0.292745619 | -2.51438585 |
| XLOC_l2_013857 | -1.09886825 | 2.774517077 | -2.954711332 | 0.00962838 | 0.292745619 | -2.514906955 |
| CHEK2 | -0.647479795 | 4.339050503 | -2.954647325 | 0.009629645 | 0.292745619 | -2.515015897 |
| DOCK9-AS2 | -0.57172753 | 5.639399769 | -2.954545366 | 0.00963166 | 0.292745619 | -2.515189433 |
| PPRC1 | -0.332806741 | 8.372486934 | -2.954325912 | 0.009635998 | 0.292745619 | -2.515562944 |
| ACSL1 | 1.145122705 | 10.40291521 | 2.954215035 | 0.009638191 | 0.292745619 | -2.515751655 |
| KLRK1 | -0.739341057 | 5.884542646 | -2.954203441 | 0.00963842 | 0.292745619 | -2.515771387 |
| lnc-ZBTB44-1 | 0.621361901 | 1.267502813 | 2.95369392 | 0.009648502 | 0.292745619 | -2.516638569 |
| F11R | 0.435888699 | 11.02319703 | 2.953546677 | 0.009651418 | 0.292745619 | -2.516889167 |
| ZNF595 | -0.750131778 | 5.287046116 | -2.952674538 | 0.009668704 | 0.292946978 | -2.518373438 |
| SMIM1 | 1.060485309 | 8.318542025 | 2.951648189 | 0.009689086 | 0.292946978 | -2.520120063 |
| NCAPH2 | -0.252335478 | 10.1162343 | -2.951617013 | 0.009689706 | 0.292946978 | -2.520173116 |
| WISP2 | 0.749057134 | 1.219938215 | 2.951511904 | 0.009691796 | 0.292946978 | -2.520351982 |
| FAM78A | -0.331465661 | 10.35572566 | -2.950157363 | 0.009718767 | 0.293506778 | -2.522656946 |
| AKIRIN2 | 0.576848695 | 7.463353319 | 2.949581795 | 0.00973025 | 0.293598256 | -2.523636313 |
| LOC100287896 | -0.502186507 | 5.406444125 | -2.948521632 | 0.009751436 | 0.293653621 | -2.525440163 |
| HS3ST3B1 | -0.546178271 | 6.686338933 | -2.947529387 | 0.009771305 | 0.293653621 | -2.527128354 |
| SGCE | -1.061840225 | 3.913628677 | -2.947385299 | 0.009774193 | 0.293653621 | -2.527373495 |
| lnc-RNF13-2 | 0.901983919 | 3.541722206 | 2.94708201 | 0.009780276 | 0.293653621 | -2.527889482 |
| HOMER3 | 0.444259851 | 10.1732702 | 2.946874203 | 0.009784446 | 0.293653621 | -2.52824302 |
| NCF4 | 0.878468164 | 9.322042763 | 2.946685639 | 0.009788231 | 0.293653621 | -2.528563817 |
| HN1L | -0.432665393 | 7.111606545 | -2.946308195 | 0.009795813 | 0.293653621 | -2.529205938 |
| HLX | 0.853459849 | 4.975719672 | 2.945877531 | 0.00980447 | 0.293653621 | -2.529938584 |
| lnc-DMRT2-1 | 0.918782885 | 3.937495917 | 2.945619295 | 0.009809665 | 0.293653621 | -2.530377885 |
| COG1 | -0.255446707 | 9.451718409 | -2.945272831 | 0.009816638 | 0.293653621 | -2.530967265 |
| CALR | -0.568012724 | 13.07492766 | -2.944755944 | 0.009827052 | 0.293712134 | -2.531846537 |
| NANOS2 | 0.973601342 | 1.589496293 | 2.944020136 | 0.009841894 | 0.293887964 | -2.533098169 |
| NUTM2F | -0.593173991 | 6.49197097 | -2.943577427 | 0.009850834 | 0.293887964 | -2.533851205 |
| SNORD4B | -0.616163336 | 3.777860601 | -2.94320618 | 0.009858337 | 0.293887964 | -2.53448267 |
| lnc-TNFRSF14-1 | 1.096946882 | 1.974946589 | 2.942477762 | 0.009873076 | 0.293887964 | -2.53572162 |
| FAR1 | 0.284375328 | 7.406347401 | 2.94237066 | 0.009875245 | 0.293887964 | -2.535903784 |
| LINC01560 | -1.093311603 | 2.457180727 | -2.94006085 | 0.009922133 | 0.294033218 | -2.539832118 |
| CDNF | -1.023600083 | 1.480076211 | -2.93991486 | 0.009925104 | 0.294033218 | -2.540080388 |
| DARS2 | -0.541743086 | 4.976175081 | -2.939196743 | 0.009939731 | 0.294033218 | -2.541301584 |
| SLC30A4 | -0.662623886 | 3.572929456 | -2.939121119 | 0.009941272 | 0.294033218 | -2.541430183 |
| TLR1 | 0.595290869 | 10.79131886 | 2.938734713 | 0.009949152 | 0.294033218 | -2.542087262 |
| USP49 | 0.413048815 | 4.762539034 | 2.938510827 | 0.009953721 | 0.294033218 | -2.542467972 |
| PDE2A | 0.792878289 | 4.930276654 | 2.937915295 | 0.009965883 | 0.294033218 | -2.543480627 |
| IMP4 | -0.423593384 | 7.338104465 | -2.937870763 | 0.009966793 | 0.294033218 | -2.543556347 |
| P2RY8 | -0.42014403 | 10.392184 | -2.937845825 | 0.009967302 | 0.294033218 | -2.543598752 |
| NOA1 | -0.290723894 | 9.097252098 | -2.937483674 | 0.009974706 | 0.294033218 | -2.544214541 |
| FTCD | -0.426737854 | 1.057259508 | -2.937294253 | 0.009978581 | 0.294033218 | -2.544536619 |
| GPR68 | -0.680214121 | 8.564290928 | -2.937140828 | 0.009981721 | 0.294033218 | -2.544797491 |
| lnc-P2RY2-2 | 0.998859629 | 2.774248081 | 2.936018078 | 0.010004725 | 0.294326812 | -2.546706454 |
| NAMPT | 0.852644673 | 12.45218543 | 2.93568009 | 0.01001166 | 0.294326812 | -2.547281096 |
| DUSP2 | -0.750299477 | 4.986318556 | -2.935120383 | 0.010023156 | 0.294326812 | -2.548232675 |
| lnc-LDLRAP1-1 | -0.4394473 | 5.39939682 | -2.934921147 | 0.010027251 | 0.294326812 | -2.548571396 |
| lnc-GLS-2 | -0.779881245 | 4.343306779 | -2.93398785 | 0.010046455 | 0.294326812 | -2.550158042 |
| lnc-CPEB4-1 | 0.62371729 | 7.437985468 | 2.933341757 | 0.01005977 | 0.294326812 | -2.551256376 |
| lnc-TRAPPC8-2 | -0.460479283 | 5.705989809 | -2.932948928 | 0.010067875 | 0.294326812 | -2.551924151 |
| MBOAT2 | 0.912975146 | 6.424329155 | 2.931926824 | 0.010088991 | 0.294326812 | -2.553661563 |
| BMPR2 | -0.757504124 | 3.385605853 | -2.931561315 | 0.010096553 | 0.294326812 | -2.554282846 |
| KRT3 | 0.589105559 | 5.662957815 | 2.93112089 | 0.010105673 | 0.294326812 | -2.555031448 |
| MRPL9 | -0.492897031 | 9.943560571 | -2.930134756 | 0.01012612 | 0.294326812 | -2.556707537 |
| LINC00847 | -0.282142323 | 8.446196209 | -2.929947398 | 0.01013001 | 0.294326812 | -2.55702597 |
| PRIM2 | -0.482963623 | 6.532540093 | -2.929804905 | 0.010132969 | 0.294326812 | -2.557268148 |
| STX4 | 0.267758114 | 9.93920354 | 2.929679204 | 0.01013558 | 0.294326812 | -2.557481785 |
| DPRXP4 | 0.724846933 | 7.259755075 | 2.928480146 | 0.010160519 | 0.294326812 | -2.559519584 |
| TSSC1 | -0.298718205 | 8.526570491 | -2.928154613 | 0.010167301 | 0.294326812 | -2.560072802 |
| ORC5 | -0.549342444 | 7.495016085 | -2.92690354 | 0.010193403 | 0.294326812 | -2.562198802 |
| TMEM40 | 0.795791788 | 7.178865363 | 2.926294196 | 0.01020614 | 0.294326812 | -2.563234226 |
| DDHD1 | -0.331180646 | 6.150252533 | -2.925505331 | 0.010222653 | 0.294326812 | -2.564574645 |
| C11orf24 | -0.398012469 | 8.439635407 | -2.924983543 | 0.01023359 | 0.294326812 | -2.565461218 |
| lnc-C12orf48-1 | -0.824954031 | 2.394351011 | -2.922824662 | 0.010278961 | 0.294326812 | -2.569129088 |
| PDGFD | -0.90288169 | 3.404089855 | -2.922574538 | 0.010284231 | 0.294326812 | -2.569554011 |
| IPO5P1 | -0.498542689 | 6.995283251 | -2.922463732 | 0.010286566 | 0.294326812 | -2.56974225 |
| CFAP61 | -0.611640472 | 1.582647723 | -2.922419899 | 0.01028749 | 0.294326812 | -2.569816714 |
| lnc-C2orf76-1 | 0.718452445 | 4.74814216 | 2.922003186 | 0.010296277 | 0.294326812 | -2.570524623 |
| MAP3K10 | -0.446734544 | 7.21967019 | -2.921970645 | 0.010296964 | 0.294326812 | -2.570579903 |
| GTPBP8 | -0.488493407 | 9.776190386 | -2.921864157 | 0.01029921 | 0.294326812 | -2.570760801 |
| LOC101060179 | -0.427565536 | 5.453597049 | -2.921836898 | 0.010299786 | 0.294326812 | -2.570807107 |
| XYLB | -0.737610256 | 1.547170717 | -2.921814257 | 0.010300264 | 0.294326812 | -2.57084557 |
| lnc-CPT2-6 | 0.623232433 | 3.216507632 | 2.920749366 | 0.010322761 | 0.294326812 | -2.572654496 |
| lnc-CD99L2-2 | -0.826323552 | 1.582670727 | -2.919793858 | 0.010342989 | 0.294326812 | -2.574277515 |
| TCOF1 | -0.321009549 | 8.808191319 | -2.919537762 | 0.010348417 | 0.294326812 | -2.574712501 |
| HLTF | -0.644989853 | 5.98892886 | -2.918757523 | 0.010364971 | 0.294326812 | -2.576037717 |
| TMED2 | -0.33751166 | 10.6786944 | -2.918608431 | 0.010368137 | 0.294326812 | -2.576290939 |
| IL2RG | -0.250492795 | 11.80168003 | -2.918130274 | 0.010378298 | 0.294326812 | -2.577103038 |
| UBE2B | 0.438985698 | 6.692533214 | 2.917724187 | 0.010386935 | 0.294326812 | -2.577792714 |
| FAM131B | -1.347175741 | 2.703137613 | -2.917487219 | 0.010391979 | 0.294326812 | -2.57819516 |
| ETS1 | -0.587006667 | 6.731998652 | -2.917080416 | 0.010400642 | 0.294326812 | -2.578886025 |
| GPR97 | 0.907937189 | 7.887847241 | 2.916757662 | 0.010407521 | 0.294326812 | -2.579434139 |
| LOC93432 | 1.381788562 | 1.71502656 | 2.916632282 | 0.010410194 | 0.294326812 | -2.579647062 |
| lnc-C12orf49-2 | 0.604222605 | 7.230990002 | 2.915119341 | 0.010442505 | 0.294326812 | -2.582216235 |
| RARA-AS1 | 0.778432559 | 6.076112088 | 2.915086036 | 0.010443218 | 0.294326812 | -2.582272789 |
| lnc-HSPA4-2 | 0.611630716 | 1.220781445 | 2.913930541 | 0.010467962 | 0.294326812 | -2.584234805 |
| SNORA11C | 0.651207245 | 7.094730029 | 2.913550599 | 0.010476111 | 0.294326812 | -2.58487991 |
| ERV18-1 | -0.603596643 | 2.163176661 | -2.912552359 | 0.010497551 | 0.294326812 | -2.586574756 |
| lnc-NCOA5-1 | 1.030578419 | 3.831604894 | 2.911863809 | 0.010512364 | 0.294326812 | -2.587743739 |
| LINC00865 | 1.275054089 | 2.235808342 | 2.911552342 | 0.010519072 | 0.294326812 | -2.588272513 |
| SCARNA20 | 0.689952011 | 5.083405761 | 2.911307466 | 0.010524348 | 0.294326812 | -2.58868823 |
| FGD4 | 0.763604822 | 9.059389297 | 2.9107912 | 0.010535481 | 0.294326812 | -2.589564653 |
| lnc-TOR2A-1 | -0.327654207 | 1.043441081 | -2.910721505 | 0.010536984 | 0.294326812 | -2.589682967 |
| QARS | -0.34275464 | 10.99562936 | -2.910477624 | 0.010542248 | 0.294326812 | -2.590096972 |
| lnc-SPIRE2-1 | -0.667619433 | 6.765669028 | -2.910166726 | 0.010548962 | 0.294326812 | -2.590624734 |
| MLC1 | -0.81428046 | 7.828727563 | -2.908635272 | 0.010582095 | 0.294326812 | -2.593224294 |
| GRAMD3 | -0.840758223 | 4.605145804 | -2.908540231 | 0.010584154 | 0.294326812 | -2.593385613 |
| SP4 | -0.469992031 | 8.20635818 | -2.908475067 | 0.010585566 | 0.294326812 | -2.593496218 |
| APOM | -0.312469512 | 6.381736396 | -2.908247559 | 0.010590499 | 0.294326812 | -2.593882376 |
| RGL2 | 0.578215688 | 8.651197168 | 2.908231147 | 0.010590855 | 0.294326812 | -2.593910232 |
| MIR92A1 | -1.013715706 | 2.683964933 | -2.907919266 | 0.01059762 | 0.294326812 | -2.594439589 |
| SCGB2B2 | 0.830844978 | 4.817608869 | 2.907707769 | 0.01060221 | 0.294326812 | -2.594798558 |
| ZNF420 | -0.393196343 | 8.003944853 | -2.907442831 | 0.010607963 | 0.294326812 | -2.595248223 |
| KLF12 | -0.730739617 | 9.437562061 | -2.907295809 | 0.010611157 | 0.294326812 | -2.595497753 |
| PIP4K2B | -0.275584753 | 9.604295226 | -2.907229823 | 0.01061259 | 0.294326812 | -2.595609745 |
| ARID1B | -0.274810675 | 7.496241193 | -2.90714475 | 0.010614439 | 0.294326812 | -2.595754133 |
| LOC729732 | 1.043999667 | 7.556474485 | 2.906593672 | 0.010626422 | 0.294326812 | -2.596689408 |
| LYZL1 | 1.48836751 | 2.622883984 | 2.906310784 | 0.010632578 | 0.294326812 | -2.597169506 |
| UBR2 | 0.384515294 | 9.481091856 | 2.906034425 | 0.010638596 | 0.294326812 | -2.597638513 |
| APBB3 | 0.376178253 | 8.00256 | 2.905884492 | 0.010641862 | 0.294326812 | -2.597892961 |
| MLF1 | -1.013125734 | 3.030703103 | -2.905666442 | 0.010646614 | 0.294326812 | -2.598263004 |
| CRTAM | -0.714236232 | 7.097979455 | -2.905292664 | 0.010654764 | 0.294326812 | -2.598897315 |
| UBXN2B | 0.534888477 | 7.412905569 | 2.904702556 | 0.010667644 | 0.294326812 | -2.599898711 |
| SMKR1 | -0.846263519 | 5.134726761 | -2.904652757 | 0.010668732 | 0.294326812 | -2.599983216 |
| FBXW8 | -0.523550352 | 3.887207903 | -2.904286387 | 0.010676736 | 0.294326812 | -2.600604916 |
| ATF7IP2 | -0.579818189 | 7.894872184 | -2.90369425 | 0.010689686 | 0.294326812 | -2.601609691 |
| ALKBH2 | -0.489535532 | 7.540852352 | -2.902993982 | 0.010705021 | 0.294326812 | -2.602797901 |
| lnc-OR4M2-7 | 0.51836158 | 7.131915039 | 2.90291135 | 0.010706832 | 0.294326812 | -2.602938107 |
| MRPS9 | -0.430353298 | 9.124824704 | -2.902720057 | 0.010711025 | 0.294326812 | -2.60326268 |
| NRCAM | -1.55134864 | 3.537238954 | -2.902027321 | 0.010726224 | 0.294326812 | -2.604438038 |
| MEGF9 | 0.671300756 | 8.750515592 | 2.901919293 | 0.010728597 | 0.294326812 | -2.604621323 |
| GTPBP10 | -0.526008477 | 3.838087814 | -2.901892412 | 0.010729187 | 0.294326812 | -2.60466693 |
| FAM45A | 0.350802393 | 9.122147235 | 2.901517886 | 0.010737415 | 0.294326812 | -2.605302355 |
| ATP6V1C2 | -0.702559326 | 4.183146228 | -2.900762604 | 0.010754027 | 0.294326812 | -2.606583732 |
| MRPS2 | -0.280067588 | 9.442956825 | -2.900686188 | 0.01075571 | 0.294326812 | -2.606713372 |
| C12orf65 | -0.437859371 | 9.074451263 | -2.90032826 | 0.010763592 | 0.294326812 | -2.607320592 |
| RIPK3 | 0.329385473 | 6.446420532 | 2.900282235 | 0.010764606 | 0.294326812 | -2.607398672 |
| ECHDC3 | 1.356105365 | 3.32652983 | 2.900257211 | 0.010765157 | 0.294326812 | -2.607441124 |
| KCNE2 | -0.750648342 | 0.956308923 | -2.900052509 | 0.010769669 | 0.294326812 | -2.60778839 |
| TPX2 | -0.872444776 | 4.159934793 | -2.89999112 | 0.010771022 | 0.294326812 | -2.607892533 |
| MTL5 | 0.408849155 | 5.487017564 | 2.899673014 | 0.010778037 | 0.294326812 | -2.608432171 |
| ZNF599 | -0.700519692 | 2.315058632 | -2.898968528 | 0.010793588 | 0.294326812 | -2.609627229 |
| USP13 | -0.426009443 | 4.590909943 | -2.898510843 | 0.010803703 | 0.294326812 | -2.610403596 |
| lnc-GNG5P2-1 | -0.32604141 | 1.150361246 | -2.898455673 | 0.010804923 | 0.294326812 | -2.610497179 |
| MTF1 | 0.312610382 | 7.798743655 | 2.898280103 | 0.010808806 | 0.294326812 | -2.610794989 |
| SRSF11 | -0.291747766 | 10.82197018 | -2.897786288 | 0.010819735 | 0.294326812 | -2.611632605 |
| RAD54L | -0.643286898 | 3.768011035 | -2.896272386 | 0.010853308 | 0.294326812 | -2.61420034 |
| lnc-USP24-1 | -1.236860693 | 2.501670625 | -2.896032388 | 0.010858639 | 0.294326812 | -2.614607378 |
| HIST2H2AA4 | 0.829706063 | 7.778903774 | 2.895980919 | 0.010859783 | 0.294326812 | -2.614694669 |
| ZNF117 | 0.85357422 | 7.044178569 | 2.895606353 | 0.01086811 | 0.294326812 | -2.61532992 |
| lnc-B3GNT7-1 | -0.838866875 | 1.267786094 | -2.894560826 | 0.010891386 | 0.294326812 | -2.617103018 |
| XLOC_l2_015121 | 0.817977112 | 1.688696613 | 2.894314748 | 0.010896871 | 0.294326812 | -2.617520321 |
| XLOC_l2_012836 | 1.059819852 | 6.142896271 | 2.89363601 | 0.010912015 | 0.294326812 | -2.618671302 |
| IDE | -0.392607634 | 6.25261866 | -2.893557635 | 0.010913765 | 0.294326812 | -2.618804204 |
| MCHR2-AS1 | 1.153766979 | 3.117330029 | 2.893344413 | 0.010918527 | 0.294326812 | -2.619165766 |
| MAGED2 | -0.24752818 | 7.013747058 | -2.892922198 | 0.010927964 | 0.294326812 | -2.619881705 |
| LOC100134937 | -0.459883884 | 5.967989092 | -2.892678565 | 0.010933412 | 0.294326812 | -2.620294817 |
| TSGA10 | -0.960022825 | 3.550313241 | -2.892676726 | 0.010933453 | 0.294326812 | -2.620297936 |
| FEN1 | -0.66552845 | 3.960597769 | -2.892181724 | 0.010944532 | 0.294326812 | -2.62113726 |
| C12orf66 | -0.95506227 | 2.692577869 | -2.892073961 | 0.010946945 | 0.294326812 | -2.621319978 |
| SCARNA23 | -0.972705991 | 1.680218289 | -2.890584406 | 0.010980355 | 0.294326812 | -2.623845477 |
| GPRIN3 | -0.723955119 | 3.648915586 | -2.890427726 | 0.010983876 | 0.294326812 | -2.624111109 |
| CD99L2 | -0.305263747 | 10.04417969 | -2.890313566 | 0.010986441 | 0.294326812 | -2.624304651 |
| LILRB2 | 0.624261358 | 12.30673683 | 2.889727527 | 0.01099962 | 0.294326812 | -2.625298178 |
| ARAP3 | 0.670140207 | 10.3018984 | 2.889711915 | 0.010999971 | 0.294326812 | -2.625324645 |
| STYX | -0.384682037 | 8.674477063 | -2.889131137 | 0.011013048 | 0.294326812 | -2.626309214 |
| REPS1 | -0.405352451 | 9.34442311 | -2.889035416 | 0.011015205 | 0.294326812 | -2.626471483 |
| TMUB2 | 0.386110396 | 8.490761104 | 2.888794241 | 0.01102064 | 0.294326812 | -2.626880323 |
| PPP1R3B | 0.819898209 | 10.12438527 | 2.888751692 | 0.011021599 | 0.294326812 | -2.626952452 |
| lnc-PCDH7-6 | 1.010803875 | 2.128459994 | 2.888716459 | 0.011022394 | 0.294326812 | -2.627012178 |
| DDX19A | -0.440552263 | 7.620950038 | -2.888532246 | 0.011026548 | 0.294326812 | -2.62732445 |
| LOC101059976 | -0.804869076 | 1.23925406 | -2.887808616 | 0.011042881 | 0.294326812 | -2.62855109 |
| LOC102723466 | 1.163733167 | 2.3541776 | 2.887361887 | 0.011052977 | 0.294326812 | -2.629308318 |
| ANGPT4 | 0.814163906 | 1.494173498 | 2.886703692 | 0.011067867 | 0.294326812 | -2.630423953 |
| EVL | -0.516491911 | 11.93605846 | -2.886549512 | 0.011071358 | 0.294326812 | -2.630685279 |
| FCRL3 | -0.774889879 | 9.370644775 | -2.886315611 | 0.011076656 | 0.294326812 | -2.631081723 |
| APOBEC3B | 1.011688857 | 8.836212733 | 2.886232016 | 0.01107855 | 0.294326812 | -2.631223409 |
| LOC648570 | 0.745050692 | 1.172334738 | 2.886006412 | 0.011083663 | 0.294326812 | -2.631605782 |
| LINC00487 | 0.723295957 | 2.264935927 | 2.885766637 | 0.011089099 | 0.294326812 | -2.632012167 |
| LOC254896 | 1.066846278 | 9.533354788 | 2.885646578 | 0.011091823 | 0.294326812 | -2.63221565 |
| LOC101927805 | -1.010757835 | 1.824146335 | -2.885622825 | 0.011092362 | 0.294326812 | -2.632255907 |
| ZNF227 | -0.691429097 | 4.347443585 | -2.8855076 | 0.011094976 | 0.294326812 | -2.632451192 |
| MAGED1 | -0.526533986 | 6.323969734 | -2.88523748 | 0.011101107 | 0.294326812 | -2.632908992 |
| PLIN5 | 1.331684245 | 7.767262271 | 2.885203655 | 0.011101875 | 0.294326812 | -2.632966318 |
| RABL6 | -0.471199352 | 4.566262831 | -2.883252257 | 0.011146269 | 0.294826805 | -2.636273301 |
| lnc-RP11-195B21.3.1-2 | 0.721162064 | 11.54742928 | 2.882389103 | 0.011165961 | 0.294826805 | -2.637735928 |
| lnc-MYO1G-1 | -0.419938414 | 5.769324935 | -2.882370126 | 0.011166394 | 0.294826805 | -2.637768084 |
| CD93 | 0.532904671 | 7.941662519 | 2.882140878 | 0.01117163 | 0.294826805 | -2.638156534 |
| PRRG4 | 0.921100258 | 5.211575369 | 2.881999486 | 0.01117486 | 0.294826805 | -2.638396112 |
| LOC101927636 | 1.330662187 | 2.047918773 | 2.881973412 | 0.011175456 | 0.294826805 | -2.638440292 |
| USH2A | 0.508071755 | 1.339464663 | 2.881767689 | 0.011180158 | 0.294826805 | -2.638788871 |
| PILRA | 0.395070145 | 8.859600852 | 2.881072365 | 0.011196066 | 0.294851905 | -2.639966996 |
| AAK1 | -0.455191166 | 10.81721711 | -2.880645453 | 0.011205843 | 0.294851905 | -2.640690309 |
| LMNB2 | -0.525702067 | 4.64193637 | -2.879992243 | 0.01122082 | 0.294851905 | -2.641796996 |
| N6AMT2 | -0.503278414 | 5.740462492 | -2.879920096 | 0.011222475 | 0.294851905 | -2.641919227 |
| CSRP2BP | -0.443024127 | 8.560236483 | -2.879471772 | 0.011232767 | 0.294851905 | -2.642678759 |
| CSNK2A1 | -0.227646549 | 7.454646134 | -2.878692746 | 0.011250672 | 0.294851905 | -2.643998497 |
| EEF2KMT | -0.389503348 | 6.768412183 | -2.878587289 | 0.011253098 | 0.294851905 | -2.644177145 |
| LOC286087 | -2.723102554 | 5.06708572 | -2.877808397 | 0.011271032 | 0.294851905 | -2.645496576 |
| NUTM2D | 0.37574842 | 3.37076909 | 2.87761741 | 0.011275434 | 0.294851905 | -2.645820095 |
| AADACP1 | 1.262505548 | 4.426093568 | 2.876998467 | 0.01128971 | 0.294851905 | -2.646868512 |
| ARMC5 | -0.289808774 | 8.387220121 | -2.876996654 | 0.011289752 | 0.294851905 | -2.646871583 |
| SLC38A1 | -0.606549783 | 10.33827292 | -2.876878264 | 0.011292485 | 0.294851905 | -2.647072117 |
| ZKSCAN3 | -0.717319822 | 3.293170964 | -2.876854017 | 0.011293045 | 0.294851905 | -2.647113187 |
| SPCS1 | -0.353927502 | 10.00883775 | -2.876554222 | 0.011299968 | 0.294851905 | -2.647620985 |
| SAT1 | 0.571797759 | 12.60920468 | 2.87604552 | 0.011311725 | 0.294937096 | -2.648482608 |
| AGFG2 | -0.57551274 | 6.147578495 | -2.875295062 | 0.011329091 | 0.295168302 | -2.649753654 |
| SNX20 | 0.488947449 | 5.927044313 | 2.87427071 | 0.011352838 | 0.295565265 | -2.651488488 |
| WDR81 | -0.278081329 | 9.837523197 | -2.872369746 | 0.011397035 | 0.295622441 | -2.654707624 |
| C11orf65 | -0.583998139 | 2.753489981 | -2.872024779 | 0.011405073 | 0.295622441 | -2.655291754 |
| ZBTB39 | -0.530585098 | 3.576763293 | -2.871784212 | 0.011410682 | 0.295622441 | -2.655699096 |
| RUSC1-AS1 | -0.287100416 | 6.911481364 | -2.87151069 | 0.011417062 | 0.295622441 | -2.656162229 |
| LINC00836 | 1.228866344 | 2.487349856 | 2.87110325 | 0.011426573 | 0.295622441 | -2.656852103 |
| PERP | -0.777498143 | 6.219509536 | -2.870506359 | 0.01144052 | 0.295622441 | -2.657862717 |
| ZNF807 | -0.679568503 | 3.317911496 | -2.87039344 | 0.011443161 | 0.295622441 | -2.658053897 |
| EHBP1 | -0.29718917 | 6.060405646 | -2.870301467 | 0.011445312 | 0.295622441 | -2.658209615 |
| MANEA-AS1 | -0.817205433 | 1.782054669 | -2.869934434 | 0.0114539 | 0.295622441 | -2.658831021 |
| C3orf52 | -0.808675985 | 1.885495588 | -2.869910384 | 0.011454463 | 0.295622441 | -2.658871738 |
| AVIL | 0.914459771 | 5.925138904 | 2.869864236 | 0.011455543 | 0.295622441 | -2.658949868 |
| lnc-RP11-181C3.1.1-1 | -0.719367023 | 1.187442321 | -2.869721958 | 0.011458874 | 0.295622441 | -2.659190745 |
| FBXW12 | 1.301362442 | 2.280717912 | 2.869298602 | 0.011468792 | 0.295622441 | -2.659907478 |
| MTERF2 | -0.633976821 | 5.387134933 | -2.869067804 | 0.011474202 | 0.295622441 | -2.660298205 |
| ZNF428 | -0.468812809 | 11.75842104 | -2.868302284 | 0.011492166 | 0.295865758 | -2.661594139 |
| TIMM10B | -0.39448692 | 9.689218465 | -2.86645344 | 0.011535662 | 0.296074029 | -2.664723729 |
| ZNF260 | -0.545946338 | 7.293841592 | -2.86619012 | 0.01154187 | 0.296074029 | -2.665169425 |
| MEN1 | -0.361997409 | 6.331154159 | -2.865858905 | 0.011549683 | 0.296074029 | -2.665730028 |
| XLOC_l2_011627 | 0.94801733 | 1.762582115 | 2.865795041 | 0.01155119 | 0.296074029 | -2.665838122 |
| lnc-EGLN1-1 | 0.887323549 | 7.701383898 | 2.865561143 | 0.011556712 | 0.296074029 | -2.666234001 |
| ZNF783 | -0.428623919 | 6.669770725 | -2.865499983 | 0.011558156 | 0.296074029 | -2.666337514 |
| CGN | -0.810291961 | 2.385440543 | -2.86475051 | 0.011575868 | 0.296074029 | -2.667605969 |
| ITM2C | -0.914636864 | 8.976960853 | -2.864734912 | 0.011576236 | 0.296074029 | -2.667632368 |
| HOXB3 | -0.484391755 | 3.01090267 | -2.863691974 | 0.011600928 | 0.296074029 | -2.669397387 |
| lnc-AMZ2-1 | -0.691841902 | 5.519449736 | -2.863612479 | 0.011602812 | 0.296074029 | -2.669531915 |
| CSPG4 | 1.258865878 | 1.845609223 | 2.863549423 | 0.011604307 | 0.296074029 | -2.669638624 |
| KRTAP13-2 | 0.706617712 | 1.591719514 | 2.863081036 | 0.011615416 | 0.296074029 | -2.670431251 |
| ARL4C | -0.552681573 | 11.3932975 | -2.862972835 | 0.011617983 | 0.296074029 | -2.67061435 |
| lnc-RP3-368B9.1.1-1 | 0.999086155 | 1.534674236 | 2.862063749 | 0.011639579 | 0.296074029 | -2.672152662 |
| ALG1 | -0.24312248 | 7.602854324 | -2.861860958 | 0.011644401 | 0.296074029 | -2.672495803 |
| ZNF70 | -0.56261829 | 4.07839826 | -2.861562908 | 0.011651493 | 0.296074029 | -2.673000119 |
| FAF1 | -0.379081361 | 7.834478907 | -2.861547732 | 0.011651854 | 0.296074029 | -2.673025798 |
| GPR82 | 0.803541773 | 1.504383636 | 2.861469935 | 0.011653706 | 0.296074029 | -2.673157433 |
| CEACAM4 | 0.912544932 | 8.175651565 | 2.860499598 | 0.011676826 | 0.296271182 | -2.674799214 |
| SNHG16 | -0.317889124 | 9.942939725 | -2.860428291 | 0.011678527 | 0.296271182 | -2.674919858 |
| GPR15 | 0.714407242 | 3.874028365 | 2.859502107 | 0.011700641 | 0.296368531 | -2.676486821 |
| PSG11 | -0.622987133 | 1.413233169 | -2.859390424 | 0.01170331 | 0.296368531 | -2.676675765 |
| FEM1A | -0.349872351 | 5.322921934 | -2.85919571 | 0.011707965 | 0.296368531 | -2.677005176 |
| lnc-WDR67-1 | -0.646471095 | 1.592759878 | -2.858507219 | 0.01172444 | 0.296569405 | -2.678169907 |
| MAPRE2 | -0.293760787 | 9.157424319 | -2.85811571 | 0.011733818 | 0.296590614 | -2.678832202 |
| PIGO | -0.325496454 | 6.472140855 | -2.856715247 | 0.011767425 | 0.29713799 | -2.681201144 |
| MMRN1 | 0.968660744 | 4.46523073 | 2.856500584 | 0.011772585 | 0.29713799 | -2.681564236 |
| LZTFL1 | -0.699107361 | 4.449615612 | -2.85605726 | 0.011783247 | 0.297191129 | -2.682314076 |
| GPD1L | -0.415657099 | 7.201068522 | -2.855594519 | 0.011794387 | 0.297256215 | -2.683096733 |
| lnc-AC110373.1-6 | -0.914935113 | 1.681451978 | -2.854928981 | 0.011810427 | 0.297325806 | -2.684222346 |
| LOC101929505 | 0.581993145 | 1.412530107 | 2.85465093 | 0.011817134 | 0.297325806 | -2.684692593 |
| PLXDC2 | 0.663435308 | 8.540400518 | 2.854003987 | 0.011832754 | 0.297325806 | -2.685786683 |
| LOC101929023 | 0.637802598 | 3.741168641 | 2.853830308 | 0.011836951 | 0.297325806 | -2.686080396 |
| ZNF551 | -0.565868129 | 6.03837374 | -2.853356234 | 0.011848415 | 0.297325806 | -2.686882092 |
| BZW2 | -0.494411241 | 8.744911078 | -2.8527127 | 0.011863993 | 0.297325806 | -2.687970318 |
| NOL10 | -0.367029186 | 7.691297628 | -2.852693907 | 0.011864448 | 0.297325806 | -2.688002096 |
| URI1 | -0.368490979 | 6.817269383 | -2.852644824 | 0.011865637 | 0.297325806 | -2.688085094 |
| LINC00968 | 0.751842842 | 3.17011593 | 2.852019323 | 0.0118808 | 0.297375195 | -2.68914277 |
| TNS4 | -0.71833061 | 4.294330923 | -2.851857191 | 0.011884733 | 0.297375195 | -2.689416915 |
| ABHD14A | -0.456315384 | 9.922122967 | -2.850575877 | 0.011915862 | 0.297578947 | -2.69158335 |
| EIF3B | -0.346769349 | 10.15566428 | -2.850474125 | 0.011918337 | 0.297578947 | -2.691755384 |
| FAM212B | 0.74843243 | 8.428181724 | 2.849954191 | 0.011930994 | 0.297578947 | -2.692634421 |
| PPP2R5E | -0.274958686 | 9.216552837 | -2.849887823 | 0.011932611 | 0.297578947 | -2.692746624 |
| GRWD1 | -0.408575739 | 7.849402623 | -2.848907298 | 0.011956518 | 0.297578947 | -2.69440427 |
| ZDHHC18 | 0.833222673 | 7.424136771 | 2.848588811 | 0.011964294 | 0.297578947 | -2.694942669 |
| AOAH | 0.592957551 | 8.633995964 | 2.848484101 | 0.011966851 | 0.297578947 | -2.695119676 |
| YPEL3 | 0.444136267 | 9.275520157 | 2.848104786 | 0.01197612 | 0.297578947 | -2.695760884 |
| USP15 | 0.592585029 | 11.51832395 | 2.847982359 | 0.011979114 | 0.297578947 | -2.695967836 |
| TUBA1A | 0.521302873 | 8.698841066 | 2.847749162 | 0.011984817 | 0.297578947 | -2.696362027 |
| ASAP1-IT1 | 0.842751256 | 8.143715744 | 2.847654704 | 0.011987128 | 0.297578947 | -2.696521695 |
| GNPTAB | -0.500751673 | 9.024477363 | -2.846854171 | 0.01200673 | 0.29767114 | -2.697874842 |
| NLRP2 | -1.037264853 | 4.602970327 | -2.846062049 | 0.012026157 | 0.29767114 | -2.699213693 |
| COX7A1 | 0.655665077 | 1.306496755 | 2.845962772 | 0.012028594 | 0.29767114 | -2.699381486 |
| XLOC_l2_005276 | -0.656905871 | 1.221185313 | -2.845625927 | 0.012036866 | 0.29767114 | -2.699950796 |
| NTF4 | -0.344121674 | 0.804065187 | -2.84529649 | 0.012044962 | 0.29767114 | -2.700507574 |
| LRRC36 | -1.333404844 | 3.968605398 | -2.845016146 | 0.012051855 | 0.29767114 | -2.700981367 |
| ILF2 | -0.247193435 | 10.17794323 | -2.844636311 | 0.012061201 | 0.29767114 | -2.701623292 |
| ZNF485 | -0.739636696 | 4.837856852 | -2.844395011 | 0.012067142 | 0.29767114 | -2.702031081 |
| LOC101928948 | 1.068273846 | 8.413020401 | 2.843995784 | 0.012076977 | 0.29767114 | -2.702705747 |
| ODF3B | 0.695529661 | 9.972056984 | 2.843552523 | 0.012087906 | 0.29767114 | -2.703454802 |
| COL5A2 | -1.14588681 | 1.751828918 | -2.843550152 | 0.012087965 | 0.29767114 | -2.703458809 |
| ZNF469 | -0.802229397 | 6.041421597 | -2.843117091 | 0.012098652 | 0.29767114 | -2.704190606 |
| lnc-RP11-1396O13.13.1-1 | -1.037229271 | 2.492343138 | -2.842752674 | 0.012107652 | 0.29767114 | -2.704806387 |
| RPN2 | -0.385702466 | 10.37032437 | -2.841785162 | 0.012131579 | 0.29767114 | -2.706441183 |
| NSFL1C | 0.59806296 | 6.726509247 | 2.841752185 | 0.012132395 | 0.29767114 | -2.706496902 |
| USP37 | -0.433869275 | 6.520915396 | -2.841648804 | 0.012134955 | 0.29767114 | -2.706671578 |
| DNMT1 | -0.448439009 | 7.481051459 | -2.841296233 | 0.012143688 | 0.29767114 | -2.707267277 |
| CAMP | 1.391886178 | 9.5452917 | 2.840683226 | 0.012158886 | 0.29767114 | -2.708302972 |
| MFSD2B | 1.249405645 | 2.661987778 | 2.840651977 | 0.012159661 | 0.29767114 | -2.708355767 |
| LDLRAD4 | -0.434787717 | 6.702073847 | -2.839969969 | 0.012176593 | 0.29767114 | -2.709507982 |
| lnc-CMPK2-13 | 0.649283051 | 1.357684429 | 2.839912122 | 0.01217803 | 0.29767114 | -2.70960571 |
| lnc-HSPB7-1 | 0.231223838 | 1.093272195 | 2.83972083 | 0.012182784 | 0.29767114 | -2.709928876 |
| PKN2 | 0.399007639 | 8.99430255 | 2.839512041 | 0.012187975 | 0.29767114 | -2.710281595 |
| RBMX | -0.407158607 | 7.421237594 | -2.837516214 | 0.012237701 | 0.298279913 | -2.71365299 |
| TP53I13 | -0.331900179 | 10.67185712 | -2.837348611 | 0.012241886 | 0.298279913 | -2.713936085 |
| SPIN2B | -0.463081867 | 6.41901526 | -2.837221621 | 0.012245058 | 0.298279913 | -2.71415058 |
| SMC2 | -0.658207268 | 5.29478732 | -2.837133671 | 0.012247255 | 0.298279913 | -2.714299133 |
| SALL2 | -0.613004359 | 5.602058936 | -2.835491793 | 0.012288341 | 0.299070845 | -2.717072174 |
| FBXO31 | -0.404805691 | 5.409154852 | -2.834156372 | 0.012321858 | 0.299431779 | -2.719327377 |
| lnc-OBSL1-1 | -0.593160325 | 1.120235216 | -2.833931402 | 0.012327513 | 0.299431779 | -2.719707274 |
| C20orf196 | -0.352441478 | 7.291278054 | -2.83387081 | 0.012329037 | 0.299431779 | -2.719809593 |
| WNT5B | -1.036825527 | 2.02208345 | -2.832617654 | 0.012360587 | 0.299944574 | -2.721925619 |
| LOC100506406 | 0.592019982 | 1.322259801 | 2.832346525 | 0.012367424 | 0.299944574 | -2.722383409 |
| LINC01501 | 0.71515745 | 3.80984907 | 2.831685668 | 0.012384103 | 0.30013949 | -2.723499201 |
| lnc-MRPL16-1 | 0.870180583 | 4.165305865 | 2.830797027 | 0.012406565 | 0.300214511 | -2.724999498 |
| SLC20A2 | -0.448321496 | 6.855496797 | -2.830419664 | 0.012416116 | 0.300214511 | -2.725636571 |
| GCN1L1 | -0.506731349 | 10.64300496 | -2.830126599 | 0.012423538 | 0.300214511 | -2.726131317 |
| PRO0628 | 0.394041116 | 7.458956448 | 2.830036005 | 0.012425833 | 0.300214511 | -2.726284255 |
| ELANE | 1.860879822 | 4.417676178 | 2.829553006 | 0.012438077 | 0.300214511 | -2.727099614 |
| SHQ1 | -0.649546785 | 4.820864134 | -2.829514098 | 0.012439064 | 0.300214511 | -2.727165295 |
| NAIP | 0.863833208 | 10.19083691 | 2.829166173 | 0.012447891 | 0.300218937 | -2.727752613 |
| DECR1 | 0.221270294 | 8.217535367 | 2.828586325 | 0.012462617 | 0.300365506 | -2.728731397 |
| LYN | 0.55559674 | 12.78415056 | 2.828179075 | 0.01247297 | 0.300382204 | -2.729418811 |
| NDUFAF4P1 | -0.541297503 | 0.976236003 | -2.827613442 | 0.012487362 | 0.300382204 | -2.73037353 |
| GTF3C2 | -0.318052073 | 9.270062684 | -2.827539026 | 0.012489257 | 0.300382204 | -2.730499131 |
| lnc-IRF8-1 | -1.032362702 | 1.571523214 | -2.827052838 | 0.012501643 | 0.300472018 | -2.731319719 |
| TXNL4A | -0.307116975 | 10.85683241 | -2.826367689 | 0.012519118 | 0.300683933 | -2.732476063 |
| FAM174A | 0.420743108 | 6.939442463 | 2.825960896 | 0.012529505 | 0.300725426 | -2.733162589 |
| PDCL3 | -0.468070258 | 9.16799513 | -2.824323643 | 0.012571393 | 0.301330885 | -2.73592549 |
| DSCR8 | 1.438281798 | 2.733450328 | 2.823245595 | 0.012599048 | 0.301330885 | -2.737744532 |
| S100B | -2.20282593 | 6.043442438 | -2.823017949 | 0.012604896 | 0.301330885 | -2.738128632 |
| MAP1LC3B | 0.39568924 | 8.953254522 | 2.82264683 | 0.012614434 | 0.301330885 | -2.738754793 |
| HCP5 | 0.675588202 | 10.62966833 | 2.822458948 | 0.012619266 | 0.301330885 | -2.739071785 |
| LRCOL1 | 0.513628391 | 6.001697134 | 2.822071494 | 0.012629235 | 0.301330885 | -2.739725479 |
| LOC102724545 | 1.040747215 | 4.631537468 | 2.821875742 | 0.012634275 | 0.301330885 | -2.740055733 |
| NDUFB8 | -0.40374178 | 11.55580307 | -2.821833422 | 0.012635365 | 0.301330885 | -2.740127133 |
| lnc-TMCC1-4 | 0.998453572 | 1.53004786 | 2.821803216 | 0.012636143 | 0.301330885 | -2.740178092 |
| SNX17 | -0.288379536 | 10.62892206 | -2.821383091 | 0.012646967 | 0.301330885 | -2.74088687 |
| lnc-SMYD3-3 | -0.960161447 | 2.712321677 | -2.821258827 | 0.012650171 | 0.301330885 | -2.741096508 |
| SLC2A1 | -0.375566699 | 7.977874064 | -2.820882235 | 0.012659884 | 0.301355558 | -2.741731816 |
| LOC399900 | 0.716828789 | 5.200708891 | 2.819873879 | 0.012685927 | 0.301646931 | -2.743432818 |
| TCF19 | -0.581036092 | 2.781662451 | -2.819617206 | 0.012692564 | 0.301646931 | -2.74386578 |
| THEMIS2 | 0.529326851 | 9.259821936 | 2.819400108 | 0.012698181 | 0.301646931 | -2.744231981 |
| SCML4 | -0.408961476 | 11.06504687 | -2.818871474 | 0.012711867 | 0.301765653 | -2.745123651 |
| RPA1 | -0.342814002 | 9.995476175 | -2.818227342 | 0.012728564 | 0.301885577 | -2.746210087 |
| SNORA11D | 0.624623409 | 6.188873 | 2.817685379 | 0.012742628 | 0.301885577 | -2.747124157 |
| KRT76 | 0.882064987 | 2.09254898 | 2.81767121 | 0.012742996 | 0.301885577 | -2.747148054 |
| ZFYVE28 | -0.592905233 | 7.195245858 | -2.816681951 | 0.012768708 | 0.302261346 | -2.748816427 |
| ACVR2B | -1.006532256 | 4.654399488 | -2.816391606 | 0.012776264 | 0.302261346 | -2.749306066 |
| DAPP1 | 0.484891948 | 8.779843095 | 2.815834934 | 0.012790763 | 0.302398373 | -2.750244809 |
| ZNF571-AS1 | -0.680891405 | 1.509118116 | -2.815023306 | 0.012811932 | 0.302501524 | -2.751613425 |
| FPR1 | 0.673498463 | 13.69796553 | 2.814999748 | 0.012812547 | 0.302501524 | -2.751653148 |
| CYP4F3 | 0.838741459 | 7.107041659 | 2.81364027 | 0.012848081 | 0.302925484 | -2.753945383 |
| ZIK1 | -0.511720884 | 4.789379153 | -2.812688457 | 0.012873017 | 0.302925484 | -2.755550102 |
| ZKSCAN2 | -0.458196563 | 5.300300817 | -2.812356468 | 0.012881726 | 0.302925484 | -2.756109793 |
| lnc-CTSL1-1 | 0.757364043 | 6.924023006 | 2.812041692 | 0.012889989 | 0.302925484 | -2.756640454 |
| CLEC4D | 1.11465831 | 7.474157854 | 2.811908267 | 0.012893492 | 0.302925484 | -2.756865381 |
| PIGM | -0.518268501 | 3.707172863 | -2.811894587 | 0.012893852 | 0.302925484 | -2.756888444 |
| SEC14L1P1 | 0.397639948 | 7.194842621 | 2.811814199 | 0.012895963 | 0.302925484 | -2.757023961 |
| MSX2P1 | -0.523035048 | 6.134467737 | -2.811409108 | 0.012906608 | 0.302925484 | -2.757706846 |
| NCOA5 | -0.303962997 | 4.541464982 | -2.811317987 | 0.012909004 | 0.302925484 | -2.75786045 |
| RBM14 | -0.243476987 | 9.680470362 | -2.810780928 | 0.012923133 | 0.303012103 | -2.75876576 |
| CASP5 | 0.474212317 | 10.6076496 | 2.810514619 | 0.012930145 | 0.303012103 | -2.759214658 |
| CXorf21 | 0.503397507 | 8.188711527 | 2.809707788 | 0.012951411 | 0.303103239 | -2.760574617 |
| PLAGL1 | 0.38841458 | 3.962287694 | 2.809704829 | 0.012951489 | 0.303103239 | -2.760579605 |
| FAM20C | 0.521944227 | 7.351927073 | 2.808989508 | 0.012970371 | 0.303133386 | -2.761785246 |
| ZBTB4 | -0.368344482 | 8.330505408 | -2.808649 | 0.012979369 | 0.303133386 | -2.762359134 |
| NMUR1 | -0.804323288 | 8.551270888 | -2.808351736 | 0.012987229 | 0.303133386 | -2.762860124 |
| DOCK5 | 0.926080404 | 7.263417321 | 2.808151119 | 0.012992536 | 0.303133386 | -2.763198227 |
| RNF216 | -0.300218778 | 8.940882754 | -2.807817195 | 0.013001375 | 0.303133386 | -2.763760979 |
| FLJ13224 | 0.82776123 | 1.621403971 | 2.807674745 | 0.013005147 | 0.303133386 | -2.764001042 |
| LINC01207 | 1.212812753 | 1.854971516 | 2.80619046 | 0.013044515 | 0.303312562 | -2.766502259 |
| lnc-C2orf71-1 | -0.802285482 | 1.624105898 | -2.806039594 | 0.013048523 | 0.303312562 | -2.766756471 |
| ZNF433 | -0.841799782 | 2.649901054 | -2.805875114 | 0.013052894 | 0.303312562 | -2.767033621 |
| TRIM61 | -0.427548464 | 4.722736846 | -2.805818242 | 0.013054405 | 0.303312562 | -2.767129451 |
| DISC1-IT1 | 0.859807079 | 4.401583448 | 2.805633201 | 0.013059325 | 0.303312562 | -2.767441239 |
| RAP2B | -0.403821623 | 6.715632195 | -2.805411022 | 0.013065234 | 0.303312562 | -2.767815597 |
| SEC14L2 | -0.720372079 | 2.317544215 | -2.804928083 | 0.013078088 | 0.303408155 | -2.768629301 |
| KIFC1 | -1.075005376 | 1.566197076 | -2.804451003 | 0.013090798 | 0.303500285 | -2.769433101 |
| PDXP | -0.680956577 | 3.937021062 | -2.804118287 | 0.013099669 | 0.303503347 | -2.769993653 |
| ACTG1P4 | -0.327574998 | 8.989420084 | -2.803043447 | 0.013128367 | 0.303594317 | -2.771804417 |
| ZFP30 | -0.703927825 | 6.219348694 | -2.801184127 | 0.013178155 | 0.303594317 | -2.774936415 |
| ZNF134 | -0.379326602 | 5.895397654 | -2.801077724 | 0.013181009 | 0.303594317 | -2.775115635 |
| MSRB1 | 0.689563854 | 11.75633094 | 2.800777233 | 0.013189074 | 0.303594317 | -2.775621759 |
| TNFAIP6 | 1.261610064 | 8.318959384 | 2.800601311 | 0.013193798 | 0.303594317 | -2.775918065 |
| LILRA2 | 0.812592818 | 7.589415745 | 2.800331396 | 0.013201049 | 0.303594317 | -2.776372672 |
| USE1 | -0.307587358 | 9.183306196 | -2.799676003 | 0.013218672 | 0.303594317 | -2.777476485 |
| lnc-PSD2-1 | 1.766702631 | 3.820543957 | 2.799287684 | 0.013229125 | 0.303594317 | -2.778130465 |
| CCNE1 | -0.456899738 | 6.697448617 | -2.798144313 | 0.013259947 | 0.303594317 | -2.780055931 |
| IL1R2 | 1.470925379 | 7.801205994 | 2.797920198 | 0.013265997 | 0.303594317 | -2.780433324 |
| SLED1 | 1.425316497 | 5.016234417 | 2.797547386 | 0.013276066 | 0.303594317 | -2.781061099 |
| LOC441081 | 0.869890169 | 7.219265804 | 2.797469164 | 0.01327818 | 0.303594317 | -2.781192815 |
| XLOC_l2_015641 | 1.326779056 | 6.327607908 | 2.797264069 | 0.013283724 | 0.303594317 | -2.781538163 |
| NKG7 | -0.7334354 | 13.60673958 | -2.796566849 | 0.013302586 | 0.303594317 | -2.782712128 |
| LHFPL2 | 0.67597634 | 6.843272361 | 2.796182848 | 0.013312986 | 0.303594317 | -2.783358673 |
| LOC158435 | 0.459807353 | 11.00038612 | 2.795941083 | 0.013319537 | 0.303594317 | -2.783765723 |
| KLF4 | 0.478814397 | 5.204533504 | 2.795069005 | 0.013343196 | 0.303594317 | -2.785233943 |
| TSPAN16 | 0.991089919 | 7.675249947 | 2.794917172 | 0.013347319 | 0.303594317 | -2.785489557 |
| DNAJA2 | -0.346754336 | 6.139268434 | -2.794716988 | 0.013352757 | 0.303594317 | -2.785826564 |
| LOC101928631 | -0.686484849 | 1.063763863 | -2.7947073 | 0.01335302 | 0.303594317 | -2.785842874 |
| DIP2B | 0.343846299 | 7.144638971 | 2.794361015 | 0.013362433 | 0.303594317 | -2.786425829 |
| XLOC_l2_007449 | -0.784252048 | 2.778260026 | -2.794203187 | 0.013366725 | 0.303594317 | -2.78669152 |
| DUS1L | -0.316837544 | 10.50368785 | -2.79384371 | 0.013376506 | 0.303594317 | -2.787296659 |
| RHOH | -0.499209113 | 9.889650234 | -2.793752206 | 0.013378996 | 0.303594317 | -2.787450691 |
| LOC102723859 | 1.059541217 | 2.005660529 | 2.793598272 | 0.013383188 | 0.303594317 | -2.787709815 |
| SLC22A31 | 1.033080204 | 5.510703165 | 2.793023803 | 0.01339884 | 0.303594317 | -2.788676811 |
| EIF3D | -0.318845936 | 11.52567416 | -2.792833218 | 0.013404037 | 0.303594317 | -2.788997609 |
| PCED1B | -0.501153411 | 10.37022597 | -2.792800289 | 0.013404935 | 0.303594317 | -2.789053036 |
| GTF3C4 | -0.340850137 | 6.066451374 | -2.792778584 | 0.013405527 | 0.303594317 | -2.789089571 |
| AKR7A2P1 | -0.60336829 | 3.611248261 | -2.7927531 | 0.013406222 | 0.303594317 | -2.789132465 |
| RBBP7 | -0.499104897 | 9.945770889 | -2.791897704 | 0.013429574 | 0.303594317 | -2.790572226 |
| RASGRP1 | -0.573403809 | 9.824125694 | -2.791363714 | 0.013444172 | 0.303594317 | -2.791470961 |
| PER2 | -0.263260094 | 6.760288684 | -2.791260991 | 0.013446982 | 0.303594317 | -2.791643844 |
| INAFM1 | 0.576709777 | 7.797964171 | 2.79066184 | 0.013463383 | 0.303594317 | -2.792652195 |
| PCDH11Y | -0.358312293 | 1.239673519 | -2.79065527 | 0.013463563 | 0.303594317 | -2.792663251 |
| lnc-CCDC23-1 | -0.73036884 | 1.706536161 | -2.789763305 | 0.013488015 | 0.303594317 | -2.794164302 |
| AGPAT9 | 0.636151389 | 8.789715047 | 2.789424685 | 0.013497309 | 0.303594317 | -2.794734123 |
| TRIT1 | -0.670126211 | 6.913993749 | -2.789369878 | 0.013498814 | 0.303594317 | -2.79482635 |
| CALML3 | 0.386152312 | 5.919137295 | 2.789369723 | 0.013498818 | 0.303594317 | -2.794826612 |
| MIF | -0.474515609 | 12.92183361 | -2.789090736 | 0.013506481 | 0.303594317 | -2.795296069 |
| ACO2 | -0.295747371 | 9.826567282 | -2.78864097 | 0.013518844 | 0.303594317 | -2.796052878 |
| SIRPD | 0.664532994 | 7.377348608 | 2.788637993 | 0.013518926 | 0.303594317 | -2.796057887 |
| TET3 | 0.827293591 | 3.699788557 | 2.788514403 | 0.013522325 | 0.303594317 | -2.796265844 |
| LIM2 | -0.966618443 | 5.052201989 | -2.787727985 | 0.013543973 | 0.303594317 | -2.797589047 |
| NDUFC2 | -0.360612547 | 9.794811193 | -2.787659703 | 0.013545854 | 0.303594317 | -2.797703932 |
| LOC643733 | 0.463448758 | 9.135143305 | 2.787649807 | 0.013546126 | 0.303594317 | -2.797720583 |
| LOC102723526 | 1.203419923 | 2.812867654 | 2.787228214 | 0.013557747 | 0.303594317 | -2.798429902 |
| ATP6V0A1 | 0.319536975 | 10.74848234 | 2.787188646 | 0.013558839 | 0.303594317 | -2.798496473 |
| CFLAR | 0.655111798 | 9.279543313 | 2.786800723 | 0.013569541 | 0.303594317 | -2.79914912 |
| lnc-CTC-236F12.4.1-3 | 0.258910872 | 0.975567067 | 2.785177717 | 0.013614406 | 0.303594317 | -2.801879461 |
| DYTN | 0.652378932 | 9.815225241 | 2.785047595 | 0.013618009 | 0.303594317 | -2.802098345 |
| lnc-MTX1-1 | 0.84296592 | 5.050672726 | 2.785013349 | 0.013618957 | 0.303594317 | -2.802155952 |
| lnc-ASF1A-2 | 0.703025934 | 3.082644506 | 2.784852494 | 0.013623413 | 0.303594317 | -2.802426531 |
| GTPBP3 | -0.394509326 | 6.446234043 | -2.784772996 | 0.013625616 | 0.303594317 | -2.802560255 |
| P3H2 | -1.648223227 | 5.135822081 | -2.784712739 | 0.013627286 | 0.303594317 | -2.802661612 |
| XLOC_l2_012150 | -0.352791576 | 7.592859644 | -2.784098287 | 0.013644324 | 0.303594317 | -2.803695153 |
| GALNS | 0.438844571 | 5.05453384 | 2.783733878 | 0.013654439 | 0.303594317 | -2.804308084 |
| GADD45B | 0.382082864 | 10.77273012 | 2.783004166 | 0.013674715 | 0.303594317 | -2.805535391 |
| SDF2 | 0.324132057 | 7.144394566 | 2.782875072 | 0.013678305 | 0.303594317 | -2.805752508 |
| RBM47 | 0.704203832 | 9.748833062 | 2.782378591 | 0.013692121 | 0.303594317 | -2.806587492 |
| LOC101927710 | 0.983226296 | 2.004239225 | 2.782137711 | 0.013698829 | 0.303594317 | -2.806992593 |
| lnc-PLEK-1 | 0.74950587 | 1.585834772 | 2.78149255 | 0.01371681 | 0.303594317 | -2.808077553 |
| lnc-TLCD2-2 | 0.964813737 | 4.174401304 | 2.781415187 | 0.013718968 | 0.303594317 | -2.808207648 |
| HIST1H3J | 0.507456814 | 8.457259813 | 2.780999568 | 0.013730566 | 0.303594317 | -2.808906555 |
| GTF3C1 | -0.391717621 | 2.850898058 | -2.78096166 | 0.013731624 | 0.303594317 | -2.808970301 |
| LTBP3 | -0.582660016 | 5.957071638 | -2.780868896 | 0.013734214 | 0.303594317 | -2.80912629 |
| lnc-C11orf1-1 | -0.567800385 | 3.852973997 | -2.780820254 | 0.013735573 | 0.303594317 | -2.809208082 |
| IL1RAP | 0.809584779 | 8.301676944 | 2.780792569 | 0.013736346 | 0.303594317 | -2.809254636 |
| MKL2 | -0.611480869 | 6.186044388 | -2.780784094 | 0.013736582 | 0.303594317 | -2.809268888 |
| HNRNPM | -0.31921491 | 9.707235926 | -2.779978707 | 0.013759093 | 0.303594317 | -2.810623137 |
| ZNF573 | -0.492087993 | 9.392424022 | -2.779880562 | 0.013761839 | 0.303594317 | -2.81078816 |
| ZNF567 | -0.604666023 | 6.102960801 | -2.779624244 | 0.013769012 | 0.303594317 | -2.811219132 |
| RPS4X | -0.590203028 | 14.36261912 | -2.779420675 | 0.013774712 | 0.303594317 | -2.811561407 |
| AP4S1 | -0.503529566 | 4.437312262 | -2.779400851 | 0.013775267 | 0.303594317 | -2.811594737 |
| TMC4 | 0.858139876 | 5.472290898 | 2.778728503 | 0.013794108 | 0.303594317 | -2.812725158 |
| EFNB2 | 0.801050117 | 1.732278624 | 2.778164136 | 0.013809943 | 0.303594317 | -2.81367398 |
| LIPN | 0.785899325 | 4.822760584 | 2.777888211 | 0.013817691 | 0.303594317 | -2.814137852 |
| CSNK2A2 | -0.320000855 | 7.956686461 | -2.777624916 | 0.013825088 | 0.303594317 | -2.814580483 |
| MYO16 | -0.647239785 | 1.174601334 | -2.777355295 | 0.013832667 | 0.303594317 | -2.815033736 |
| RANGRF | -0.400600811 | 9.8635512 | -2.777233748 | 0.013836085 | 0.303594317 | -2.815238063 |
| PLEKHO2 | 0.526003935 | 10.20307737 | 2.777113248 | 0.013839475 | 0.303594317 | -2.815440627 |
| POLE3 | -0.387098673 | 8.825773644 | -2.77669393 | 0.013851276 | 0.303594317 | -2.8161455 |
| B4GALT3 | -0.273296156 | 9.590289577 | -2.776591908 | 0.013854148 | 0.303594317 | -2.816316996 |
| XLOC_014422 | 0.474674739 | 7.872896497 | 2.77550305 | 0.013884843 | 0.303594317 | -2.818147229 |
| lnc-ATF7IP-1 | 0.810055831 | 1.493716082 | 2.775346798 | 0.013889254 | 0.303594317 | -2.818409855 |
| DCPS | -0.472042489 | 7.870305529 | -2.775317357 | 0.013890085 | 0.303594317 | -2.818459338 |
| WDR5 | -0.351113613 | 9.368725621 | -2.775149088 | 0.013894836 | 0.303594317 | -2.818742157 |
| CLSTN1 | -0.37894944 | 8.532234756 | -2.774092879 | 0.013924694 | 0.303594317 | -2.820517298 |
| SPN | -0.435802118 | 5.514214939 | -2.773631372 | 0.01393776 | 0.303594317 | -2.821292889 |
| SLC19A3 | 0.566206123 | 6.233954142 | 2.773626681 | 0.013937893 | 0.303594317 | -2.821300773 |
| SLAIN1 | -0.580054821 | 5.609760382 | -2.773459912 | 0.013942618 | 0.303594317 | -2.821581032 |
| ZNF689 | -0.332900546 | 6.372311408 | -2.773455903 | 0.013942731 | 0.303594317 | -2.821587769 |
| ZSCAN31 | -0.642307669 | 1.194985224 | -2.773103523 | 0.013952719 | 0.303594317 | -2.822179937 |
| NCBP2 | -0.522710273 | 6.473309477 | -2.772928361 | 0.013957687 | 0.303594317 | -2.822474286 |
| ARHGEF11 | 0.441748311 | 5.373199762 | 2.772740967 | 0.013963003 | 0.303594317 | -2.822789187 |
| CASP9 | 0.285948604 | 7.47089454 | 2.772682941 | 0.01396465 | 0.303594317 | -2.822886693 |
| RPAP2 | -0.432623296 | 6.629449423 | -2.772528179 | 0.013969042 | 0.303594317 | -2.823146753 |
| SLC1A7 | -1.192435033 | 7.76731342 | -2.772153105 | 0.013979693 | 0.303594317 | -2.823777007 |
| NPEPPS | 0.27786991 | 8.408920207 | 2.772070459 | 0.013982041 | 0.303594317 | -2.823915878 |
| STX18-AS1 | -0.60853052 | 3.991332575 | -2.772042859 | 0.013982825 | 0.303594317 | -2.823962254 |
| MGA | -0.296927603 | 8.234067064 | -2.771913776 | 0.013986493 | 0.303594317 | -2.824179152 |
| BRI3 | 0.285618599 | 13.01481549 | 2.771593764 | 0.013995591 | 0.303602044 | -2.824716855 |
| TRIQK | 0.406430553 | 6.386900383 | 2.771044092 | 0.014011231 | 0.303627245 | -2.825640414 |
| SLC2A4RG | -0.476490988 | 10.89415508 | -2.770938503 | 0.014014238 | 0.303627245 | -2.825817821 |
| TRIM59 | -0.678200695 | 6.204413888 | -2.769775893 | 0.014047381 | 0.304060736 | -2.827771077 |
| HIF1A-AS1 | 0.619713936 | 4.975222358 | 2.769622635 | 0.014051756 | 0.304060736 | -2.828028544 |
| ZNF286B | -0.571955556 | 6.744898783 | -2.768506087 | 0.014083667 | 0.304561499 | -2.829904206 |
| AGER | 0.548428344 | 9.047647751 | 2.767707802 | 0.014106526 | 0.304587057 | -2.831245116 |
| PHB | -0.385533588 | 7.748417234 | -2.767511211 | 0.014112161 | 0.304587057 | -2.831575323 |
| PREP | -0.357998502 | 8.298883113 | -2.767398394 | 0.014115395 | 0.304587057 | -2.831764817 |
| MAP3K7 | -0.299599733 | 8.201184393 | -2.767240276 | 0.01411993 | 0.304587057 | -2.832030394 |
| LOC339862 | 0.822125993 | 1.66276016 | 2.766763232 | 0.014133619 | 0.304670209 | -2.832831626 |
| lnc-ADAR-1 | 0.602490912 | 4.407674976 | 2.766312569 | 0.014146564 | 0.304670209 | -2.833588521 |
| WIZ | -0.189107793 | 8.593756392 | -2.766124828 | 0.01415196 | 0.304670209 | -2.833903826 |
| lnc-TCF19-1 | 0.81294564 | 10.5164243 | 2.765884331 | 0.014158875 | 0.304670209 | -2.834307724 |
| ITPRIPL1 | -0.672571973 | 5.889294272 | -2.765433576 | 0.014171844 | 0.304760462 | -2.835064714 |
| CEP78 | -0.538438802 | 6.019745024 | -2.764767962 | 0.014191017 | 0.304968027 | -2.836182481 |
| lnc-DLX2-2 | -0.869493572 | 2.609094501 | -2.764344277 | 0.014203234 | 0.304968027 | -2.836893945 |
| ANKAR | -0.639091853 | 5.641480878 | -2.763749014 | 0.014220415 | 0.304968027 | -2.83789348 |
| LOC100132593 | 0.871186103 | 2.38177243 | 2.763203881 | 0.014236168 | 0.304968027 | -2.838808794 |
| LOC102724955 | 1.031203874 | 5.85237096 | 2.762600655 | 0.014253619 | 0.304968027 | -2.839821601 |
| SCT | -0.883578002 | 3.854913001 | -2.762307115 | 0.014262118 | 0.304968027 | -2.840314431 |
| lnc-HNMT-2 | 0.729064205 | 9.772409048 | 2.762070711 | 0.014268966 | 0.304968027 | -2.840711324 |
| KCNK10 | -1.573313457 | 2.916876114 | -2.761310794 | 0.014291003 | 0.304968027 | -2.841987077 |
| C15orf61 | -0.48656048 | 7.115819159 | -2.761086429 | 0.014297515 | 0.304968027 | -2.842363727 |
| ZNF304 | -0.686350091 | 4.713570626 | -2.760957789 | 0.01430125 | 0.304968027 | -2.842579677 |
| LAT2 | 0.445153099 | 12.93720534 | 2.759991627 | 0.014329334 | 0.304968027 | -2.844201504 |
| AK5 | -1.324210546 | 3.408883401 | -2.759691682 | 0.014338064 | 0.304968027 | -2.844704972 |
| BTBD9 | -0.246345387 | 8.774182872 | -2.759622346 | 0.014340082 | 0.304968027 | -2.844821352 |
| CHPT1 | 0.369464659 | 6.917483462 | 2.759547566 | 0.01434226 | 0.304968027 | -2.844946871 |
| LRPAP1 | 0.358809091 | 7.195886862 | 2.759511327 | 0.014343315 | 0.304968027 | -2.845007697 |
| GIPC1 | -0.390434564 | 9.886059599 | -2.758568364 | 0.014370802 | 0.304968027 | -2.846590383 |
| ZNF566 | -0.997195777 | 3.017706225 | -2.75849752 | 0.014372869 | 0.304968027 | -2.846709284 |
| ZNHIT6 | -0.452342162 | 6.980527378 | -2.758300603 | 0.014378617 | 0.304968027 | -2.847039776 |
| PIK3AP1 | 0.599914258 | 5.859728974 | 2.758067928 | 0.014385411 | 0.304968027 | -2.847430272 |
| RRP9 | -0.343392657 | 7.066288085 | -2.758057712 | 0.014385709 | 0.304968027 | -2.847447418 |
| SLC6A17 | 1.017149552 | 1.78679879 | 2.758011471 | 0.014387059 | 0.304968027 | -2.847525022 |
| lnc-TM4SF4-2 | 1.159245863 | 5.850905216 | 2.75799553 | 0.014387525 | 0.304968027 | -2.847551775 |
| MEX3C | -0.589050153 | 9.093267363 | -2.757374422 | 0.014405678 | 0.304968027 | -2.848594131 |
| ITGA9 | 0.578190851 | 3.301448251 | 2.757319276 | 0.014407291 | 0.304968027 | -2.848686675 |
| PIGS | -0.235518478 | 11.23550249 | -2.757251179 | 0.014409283 | 0.304968027 | -2.848800952 |
| CDK5RAP2 | -0.287640702 | 8.000995693 | -2.757056848 | 0.014414969 | 0.304968027 | -2.849127068 |
| TANK | 0.282944674 | 9.938058354 | 2.756865435 | 0.014420571 | 0.304968027 | -2.849448279 |
| KIAA1143 | -0.337138831 | 7.723574884 | -2.756633341 | 0.014427367 | 0.304968027 | -2.849837753 |
| FRAT2 | 0.552036909 | 12.06429609 | 2.755924978 | 0.014448128 | 0.305085788 | -2.851026395 |
| HID1 | -0.645737563 | 5.117723218 | -2.755843863 | 0.014450507 | 0.305085788 | -2.851162502 |
| POMZP3 | -0.466427443 | 4.771590711 | -2.755530626 | 0.014459698 | 0.305094367 | -2.851688089 |
| lnc-EIF4E3-1 | 0.533330585 | 8.903015216 | 2.754809584 | 0.014480877 | 0.305355714 | -2.852897888 |
| XLOC_l2_011407 | 0.722608515 | 1.201974052 | 2.753978959 | 0.014505312 | 0.305685359 | -2.854291457 |
| ZNF777 | -0.426746866 | 7.601408854 | -2.753086343 | 0.014531614 | 0.305922149 | -2.855788914 |
| CPQ | 0.445904552 | 9.233478667 | 2.751790903 | 0.014569869 | 0.305922149 | -2.857961941 |
| CKLF | 0.46358161 | 10.68165277 | 2.751712317 | 0.014572193 | 0.305922149 | -2.858093758 |
| TXNRD3 | -0.913741787 | 1.902281739 | -2.751130307 | 0.014589414 | 0.305922149 | -2.859069959 |
| LOC102724448 | -0.617468751 | 3.622336897 | -2.750959596 | 0.014594469 | 0.305922149 | -2.859356282 |
| GSTCD | -1.338547854 | 2.268800317 | -2.750790003 | 0.014599492 | 0.305922149 | -2.859640724 |
| LOC100996664 | -0.595373808 | 1.136973294 | -2.75032016 | 0.014613418 | 0.305922149 | -2.860428727 |
| SNORA11B | 0.671769879 | 7.987391455 | 2.749692464 | 0.014632043 | 0.305922149 | -2.861481424 |
| KPNA3 | -0.368101128 | 8.321253428 | -2.749672758 | 0.014632628 | 0.305922149 | -2.861514473 |
| TBXAS1 | 0.550185967 | 8.716260941 | 2.74900842 | 0.014652366 | 0.305922149 | -2.862628556 |
| MLLT6 | -0.36843017 | 9.91805737 | -2.748976943 | 0.014653302 | 0.305922149 | -2.86268134 |
| lnc-C14orf166-1 | 1.557072915 | 2.215412537 | 2.748577028 | 0.014665197 | 0.305922149 | -2.863351957 |
| PDCD4-AS1 | 0.329911075 | 9.449730293 | 2.748198443 | 0.014676466 | 0.305922149 | -2.863986783 |
| lnc-ASH1L-2 | -0.845045422 | 2.256150264 | -2.747864086 | 0.014686425 | 0.305922149 | -2.864547428 |
| MIEF1 | -0.356324318 | 10.14484558 | -2.747608981 | 0.014694028 | 0.305922149 | -2.864975173 |
| TIMD4 | -0.877271367 | 4.540023808 | -2.747244121 | 0.014704909 | 0.305922149 | -2.865586932 |
| C1orf159 | -0.284779905 | 5.920169581 | -2.746926882 | 0.014714376 | 0.305922149 | -2.866118829 |
| TUFT1 | 0.408660898 | 6.817920338 | 2.746903639 | 0.01471507 | 0.305922149 | -2.866157799 |
| lnc-ATG2B-2 | -0.620352879 | 4.164155113 | -2.746838444 | 0.014717016 | 0.305922149 | -2.866267105 |
| DDX59 | 0.309555495 | 6.712351533 | 2.7461011 | 0.014739047 | 0.305922149 | -2.867503301 |
| LMNB1 | 0.849077186 | 8.645824191 | 2.745914025 | 0.014744642 | 0.305922149 | -2.867816928 |
| XRCC6 | -0.368558425 | 13.06464898 | -2.745736416 | 0.014749955 | 0.305922149 | -2.868114682 |
| NOS1AP | 1.209913669 | 1.624292545 | 2.745683019 | 0.014751553 | 0.305922149 | -2.868204199 |
| MFSD6 | -0.476500889 | 7.582538013 | -2.745678595 | 0.014751685 | 0.305922149 | -2.868211615 |
| lnc-ASPSCR1-1 | 0.58674595 | 1.217552782 | 2.745327807 | 0.014762186 | 0.305922149 | -2.868799679 |
| XLOC_l2_014294 | 0.748096931 | 4.275964379 | 2.745168817 | 0.014766948 | 0.305922149 | -2.869066205 |
| C2orf61 | 1.013049242 | 2.648189741 | 2.744583079 | 0.014784504 | 0.305922149 | -2.870048084 |
| S1PR5 | -0.827381196 | 7.402749647 | -2.744174953 | 0.014796748 | 0.305922149 | -2.8707322 |
| TMEM44 | -0.857298119 | 2.887898534 | -2.74410028 | 0.014798989 | 0.305922149 | -2.870857367 |
| lnc-SBDS-10 | 1.143635924 | 3.786972054 | 2.743753021 | 0.014809417 | 0.305922149 | -2.871439431 |
| CXCR3 | -0.548640952 | 9.578709872 | -2.74367659 | 0.014811713 | 0.305922149 | -2.87156754 |
| BMS1P20 | -0.389179931 | 8.802894663 | -2.743638427 | 0.014812859 | 0.305922149 | -2.871631507 |
| AGAP1-IT1 | 1.133981559 | 1.968152764 | 2.743630926 | 0.014813085 | 0.305922149 | -2.871644079 |
| AEBP1 | -0.700094622 | 5.244761693 | -2.74353258 | 0.01481604 | 0.305922149 | -2.871808918 |
| KLHL20 | -0.354698267 | 6.016946278 | -2.743048704 | 0.014830588 | 0.306040585 | -2.872619931 |
| STX11 | 0.918532104 | 6.999413367 | 2.742630682 | 0.014843167 | 0.306118275 | -2.873320536 |
| LOC101928020 | -0.600949016 | 3.693677295 | -2.741780565 | 0.01486878 | 0.30646453 | -2.874745255 |
| ST6GAL1 | -0.601507079 | 10.98163859 | -2.741147065 | 0.014887895 | 0.30663067 | -2.875806872 |
| ZNF296 | -0.427137645 | 3.814518845 | -2.740928383 | 0.014894499 | 0.30663067 | -2.876173324 |
| PTCH2 | 0.414997393 | 4.489089552 | 2.73993312 | 0.01492459 | 0.307068134 | -2.877841026 |
| KLHL14 | -1.424215292 | 2.598864764 | -2.739361642 | 0.014941895 | 0.30724216 | -2.87879855 |
| SNORD67 | 0.92207976 | 2.747144163 | 2.738910657 | 0.014955565 | 0.307341281 | -2.879554148 |
| XLOC_l2_014549 | -0.599911466 | 4.948141392 | -2.738412743 | 0.014970671 | 0.307469789 | -2.880388339 |
| FITM2 | -0.72420793 | 2.301787427 | -2.73759296 | 0.014995575 | 0.307791783 | -2.881761695 |
| PDCD11 | -0.674343289 | 4.569845265 | -2.736586153 | 0.015026216 | 0.307791783 | -2.883448229 |
| SEC61B | -0.426461245 | 11.64807573 | -2.736442396 | 0.015030596 | 0.307791783 | -2.883689027 |
| lnc-RNF144A-1 | -0.75665527 | 1.70339382 | -2.73630569 | 0.015034762 | 0.307791783 | -2.883918014 |
| TMEM39B | -0.180172043 | 7.769969156 | -2.736132108 | 0.015040053 | 0.307791783 | -2.884208762 |
| EMC1 | -0.455257582 | 4.973349156 | -2.735929523 | 0.015046232 | 0.307791783 | -2.884548087 |
| lnc-SLC45A1-1 | 0.733920943 | 3.832713023 | 2.735630877 | 0.015055344 | 0.307791783 | -2.885048298 |
| DAD1 | -0.306011256 | 11.4985943 | -2.735193143 | 0.015068709 | 0.307791783 | -2.885781446 |
| LOC101928423 | 0.731347965 | 1.135026185 | 2.73505159 | 0.015073034 | 0.307791783 | -2.886018523 |
| CTB-7E3.1 | 0.870663523 | 1.800664644 | 2.734988117 | 0.015074973 | 0.307791783 | -2.886124829 |
| PLAC9 | 0.471732796 | 3.593641143 | 2.73414029 | 0.015100903 | 0.308140057 | -2.887544722 |
| TPTE2P5 | -1.025090662 | 3.087946751 | -2.733362821 | 0.01512472 | 0.30844481 | -2.888846685 |
| MBOAT7 | 0.665428093 | 11.92842217 | 2.732569259 | 0.015149066 | 0.308521261 | -2.890175502 |
| DDAH2 | 0.341381245 | 7.386160519 | 2.731798605 | 0.015172746 | 0.308521261 | -2.891465867 |
| CMTM2 | 0.700820013 | 10.56814527 | 2.731687605 | 0.01517616 | 0.308521261 | -2.891651716 |
| LOC100132111 | -0.887382 | 2.698350034 | -2.731366052 | 0.015186053 | 0.308521261 | -2.892190085 |
| PELI2 | 0.446085917 | 8.450605598 | 2.731316078 | 0.015187591 | 0.308521261 | -2.892273753 |
| CEP85L | -0.249282765 | 7.653506205 | -2.731132056 | 0.015193256 | 0.308521261 | -2.892581848 |
| SEC24B-AS1 | -0.54048184 | 4.362422651 | -2.730342761 | 0.015217578 | 0.308521261 | -2.893903248 |
| FUBP1 | -0.445155473 | 7.696731821 | -2.730321246 | 0.015218241 | 0.308521261 | -2.893939268 |
| MRPS11 | -0.230658988 | 8.812580878 | -2.729927346 | 0.015230394 | 0.308521261 | -2.894598679 |
| FLOT1 | 0.623259183 | 9.187164176 | 2.729845402 | 0.015232923 | 0.308521261 | -2.894735854 |
| CPSF3L | -0.246823811 | 11.19972951 | -2.729689194 | 0.015237746 | 0.308521261 | -2.894997348 |
| XLOC_l2_005403 | -0.794113835 | 1.972320755 | -2.729628099 | 0.015239632 | 0.308521261 | -2.89509962 |
| LOC101927533 | 1.27629546 | 1.853356892 | 2.729488208 | 0.015243953 | 0.308521261 | -2.895333794 |
| CXCR2 | 0.753830978 | 12.39889528 | 2.728963306 | 0.015260175 | 0.308596208 | -2.896212437 |
| lnc-OBFC2A-1 | 0.855192398 | 8.152837862 | 2.728604307 | 0.01527128 | 0.308596208 | -2.896813348 |
| CCDC136 | -0.345380111 | 6.953243102 | -2.728477871 | 0.015275193 | 0.308596208 | -2.897024978 |
| CDCA5 | -0.88283656 | 4.965706 | -2.72821929 | 0.015283198 | 0.308596208 | -2.897457786 |
| EVC | -0.610179596 | 3.91261492 | -2.727415302 | 0.015308115 | 0.30890753 | -2.898803423 |
| SMARCC2 | -0.251374289 | 11.77678467 | -2.727148061 | 0.015316406 | 0.30890753 | -2.89925068 |
| ABHD3 | 0.505009176 | 7.136554641 | 2.726473162 | 0.015337363 | 0.308911111 | -2.900380155 |
| HEPH | 1.001013566 | 2.479299967 | 2.72518819 | 0.015377341 | 0.308911111 | -2.902530416 |
| MAN1A2 | -0.318480992 | 7.546212406 | -2.725159057 | 0.015378248 | 0.308911111 | -2.902579163 |
| NOP56 | -0.459669546 | 10.40039318 | -2.725102762 | 0.015380002 | 0.308911111 | -2.902673362 |
| HIST1H2BJ | 0.52926853 | 6.095974104 | 2.724957104 | 0.015384541 | 0.308911111 | -2.902917085 |
| TPRN | -0.251888779 | 9.223815938 | -2.724609807 | 0.015395368 | 0.308911111 | -2.903498193 |
| MLKL | 0.415683466 | 9.985298479 | 2.724450734 | 0.01540033 | 0.308911111 | -2.903764351 |
| SEMA4B | 0.676173695 | 5.21975296 | 2.724385245 | 0.015402373 | 0.308911111 | -2.903873925 |
| ALDH18A1 | -0.589292477 | 6.254262468 | -2.724346639 | 0.015403578 | 0.308911111 | -2.90393852 |
| LEKR1 | -1.136757452 | 2.461611747 | -2.72409148 | 0.015411541 | 0.308911111 | -2.904365436 |
| HNRNPH2 | 0.321308825 | 8.765019368 | 2.723764014 | 0.015421767 | 0.308911111 | -2.904913317 |
| DCP1B | -0.329511237 | 8.129762685 | -2.723714341 | 0.015423319 | 0.308911111 | -2.904996424 |
| ZNF610 | -0.899595076 | 1.808776306 | -2.723092868 | 0.015442746 | 0.309051662 | -2.906036156 |
| ATP5G3 | -0.469703264 | 12.34271243 | -2.722920656 | 0.015448134 | 0.309051662 | -2.906324258 |
| lnc-DTHD1-2 | 0.664566637 | 5.338050862 | 2.720951337 | 0.015509872 | 0.309516593 | -2.909618499 |
| SEMA4A | 0.640969935 | 6.323859133 | 2.720218537 | 0.015532906 | 0.309516593 | -2.910844157 |
| CACNA1H | -0.67897025 | 3.745956593 | -2.720120399 | 0.015535993 | 0.309516593 | -2.911008293 |
| CRY2 | -0.306685392 | 8.434164866 | -2.719959844 | 0.015541045 | 0.309516593 | -2.911276819 |
| KIAA1731NL | 0.930140029 | 3.293272006 | 2.719934331 | 0.015541848 | 0.309516593 | -2.911319488 |
| PTGFRN | -0.864271789 | 2.606824793 | -2.719926822 | 0.015542085 | 0.309516593 | -2.911332047 |
| COMMD4 | -0.263103559 | 10.15007629 | -2.718991781 | 0.01557154 | 0.309516593 | -2.912895802 |
| ITM2A | -0.521654801 | 10.34073575 | -2.718792652 | 0.01557782 | 0.309516593 | -2.913228806 |
| ZNF362 | -0.272174814 | 8.358529818 | -2.718627402 | 0.015583034 | 0.309516593 | -2.913505149 |
| LOC606724 | 0.613216134 | 8.393652205 | 2.717920958 | 0.01560534 | 0.309516593 | -2.914686468 |
| RNF180 | 0.438114262 | 10.17392604 | 2.717605849 | 0.0156153 | 0.309516593 | -2.915213369 |
| PNPLA3 | 0.847157042 | 1.870801573 | 2.717500763 | 0.015618622 | 0.309516593 | -2.915389082 |
| lnc-KCNT1-1 | -0.259446542 | 3.627890397 | -2.717344671 | 0.015623559 | 0.309516593 | -2.915650079 |
| COG6 | -0.427309796 | 7.796317082 | -2.71711023 | 0.015630977 | 0.309516593 | -2.916042073 |
| TJP3 | 0.926570601 | 2.711156335 | 2.71709889 | 0.015631336 | 0.309516593 | -2.916061034 |
| BIN3 | 0.286660994 | 8.667473836 | 2.717042139 | 0.015633132 | 0.309516593 | -2.916155923 |
| TCFL5 | -0.490502924 | 6.523905143 | -2.716990471 | 0.015634768 | 0.309516593 | -2.916242312 |
| LIN52 | -0.412600032 | 4.83189423 | -2.716981313 | 0.015635057 | 0.309516593 | -2.916257623 |
| lnc-HIST1H2AI-2 | -0.597410059 | 4.249743523 | -2.716657306 | 0.015645317 | 0.309516593 | -2.916799356 |
| KDM4A | -0.212237704 | 7.503895543 | -2.716224974 | 0.015659017 | 0.309516593 | -2.917522177 |
| ZNF607 | -0.439812745 | 6.199074222 | -2.716070016 | 0.01566393 | 0.309516593 | -2.917781246 |
| CHMP2A | 0.32816253 | 11.4224621 | 2.715959376 | 0.015667439 | 0.309516593 | -2.917966219 |
| LOC280665 | 0.958970175 | 2.893658974 | 2.715165241 | 0.015692648 | 0.309520284 | -2.919293833 |
| LOC101929911 | 0.423832434 | 3.947692919 | 2.715140014 | 0.015693449 | 0.309520284 | -2.919336006 |
| MESP1 | -0.879715164 | 2.989939588 | -2.715111259 | 0.015694363 | 0.309520284 | -2.919384076 |
| DPP3 | -0.480100867 | 5.822683057 | -2.714703221 | 0.015707332 | 0.309582074 | -2.920066182 |
| lnc-RHOV-1 | -0.354937296 | 3.651554142 | -2.714451935 | 0.015715324 | 0.309582074 | -2.920486236 |
| PRRT3 | -0.25293487 | 6.662086818 | -2.713766419 | 0.015737146 | 0.309797751 | -2.921632105 |
| LIN7A | 0.860864588 | 6.901226956 | 2.713379146 | 0.015749487 | 0.309797751 | -2.922279416 |
| IMPDH2 | -0.422103138 | 8.29937233 | -2.712431679 | 0.01577972 | 0.309797751 | -2.923862965 |
| LOC100507006 | 0.963069759 | 10.63479175 | 2.712431538 | 0.015779724 | 0.309797751 | -2.923863202 |
| PRY2 | 0.767065399 | 2.140633715 | 2.712369056 | 0.01578172 | 0.309797751 | -2.923967626 |
| SLC24A3 | 0.947608305 | 6.301659806 | 2.712359886 | 0.015782013 | 0.309797751 | -2.923982951 |
| lnc-CHRAC1-1 | 0.807346447 | 1.287449152 | 2.712150154 | 0.015788713 | 0.309797751 | -2.924333465 |
| LINC01588 | -0.394900072 | 7.228780523 | -2.711171429 | 0.015820019 | 0.31023087 | -2.925969063 |
| RAB33A | -0.372300922 | 8.138962449 | -2.710779655 | 0.015832567 | 0.31023087 | -2.926623734 |
| FUT8 | -0.616840343 | 6.842892716 | -2.710623075 | 0.015837585 | 0.31023087 | -2.92688538 |
| KLHL3 | -0.61283176 | 6.478207589 | -2.708980351 | 0.015890321 | 0.310740694 | -2.929630132 |
| PPP1R11 | 0.406062774 | 5.323461054 | 2.708825185 | 0.015895311 | 0.310740694 | -2.92988937 |
| BUB1 | -1.261270237 | 3.941540361 | -2.708724213 | 0.015898559 | 0.310740694 | -2.930058063 |
| RRAGD | 0.553108521 | 5.940378738 | 2.708575773 | 0.015903335 | 0.310740694 | -2.930306057 |
| RPGRIP1 | 0.610149752 | 6.730573781 | 2.707856384 | 0.015926501 | 0.310740694 | -2.931507868 |
| ACAN | 0.785128961 | 3.085005315 | 2.707762789 | 0.015929517 | 0.310740694 | -2.931664222 |
| TMEM209 | -0.367262187 | 7.961886228 | -2.707558113 | 0.015936115 | 0.310740694 | -2.932006136 |
| ZNF470 | -0.574398667 | 3.95552843 | -2.707160332 | 0.015948946 | 0.310740694 | -2.932670613 |
| ERI3 | -0.333874366 | 5.415889709 | -2.707081014 | 0.015951506 | 0.310740694 | -2.932803107 |
| XLOC_l2_006651 | -0.765644328 | 1.645609272 | -2.707032073 | 0.015953085 | 0.310740694 | -2.932884859 |
| SEC24C | -0.20746664 | 11.63400143 | -2.706619021 | 0.015966423 | 0.310826157 | -2.933574812 |
| CFAP97 | -0.59348041 | 8.560711732 | -2.706123055 | 0.015982452 | 0.310879119 | -2.934403225 |
| SSR4 | -0.393755167 | 11.2948593 | -2.705980985 | 0.015987046 | 0.310879119 | -2.934640517 |
| SORT1 | 0.700912059 | 8.220731589 | 2.705670843 | 0.01599708 | 0.31090016 | -2.93515852 |
| LOC257152 | 0.483677703 | 8.64061693 | 2.704357175 | 0.016039648 | 0.310945974 | -2.937352454 |
| ITK | -0.640903472 | 8.151361051 | -2.703486755 | 0.016067914 | 0.310945974 | -2.938805973 |
| ZNF439 | -0.707489659 | 5.343829538 | -2.703276923 | 0.016074735 | 0.310945974 | -2.939156354 |
| AKR1C3 | -0.922808128 | 7.404570892 | -2.703269354 | 0.016074981 | 0.310945974 | -2.939168993 |
| LINC01530 | -0.75604926 | 4.017611336 | -2.703064285 | 0.01608165 | 0.310945974 | -2.939511415 |
| TMEM245 | -0.510829613 | 8.557027508 | -2.702898702 | 0.016087037 | 0.310945974 | -2.939787897 |
| CNOT2 | -0.20506224 | 10.03621976 | -2.70264247 | 0.016095376 | 0.310945974 | -2.940215733 |
| CCDC180 | -0.666752346 | 5.606376443 | -2.702545296 | 0.01609854 | 0.310945974 | -2.940377983 |
| TVP23C-CDRT4 | -0.732369345 | 1.393176538 | -2.702534097 | 0.016098905 | 0.310945974 | -2.940396682 |
| TMEM91 | 0.753876533 | 8.034683286 | 2.702488345 | 0.016100395 | 0.310945974 | -2.940473073 |
| LOC100507547 | -0.485704521 | 4.443780918 | -2.702478875 | 0.016100703 | 0.310945974 | -2.940488884 |
| TLR5 | 0.963074172 | 7.221086496 | 2.702289347 | 0.016106876 | 0.310945974 | -2.940805331 |
| PDCD7 | -0.380885662 | 9.887069291 | -2.701835792 | 0.016121658 | 0.311058436 | -2.941562587 |
| NCF2 | 0.481231115 | 11.42136722 | 2.700733765 | 0.016157629 | 0.311131654 | -2.943402396 |
| RAF1 | 0.442556016 | 9.037607743 | 2.700301887 | 0.016171747 | 0.311131654 | -2.944123353 |
| MRPL2 | -0.40500397 | 5.384691779 | -2.699992593 | 0.016181865 | 0.311131654 | -2.944639655 |
| lnc-SPACA3-1 | 1.308744865 | 2.294313042 | 2.699705954 | 0.016191248 | 0.311131654 | -2.945118125 |
| NPY4R | 0.625018849 | 3.315644675 | 2.699567593 | 0.016195778 | 0.311131654 | -2.945349078 |
| SLC23A3 | 0.497585265 | 3.792508124 | 2.699396654 | 0.016201378 | 0.311131654 | -2.945634407 |
| RFXAP | -0.737477773 | 5.068680636 | -2.699003934 | 0.016214249 | 0.311131654 | -2.946289909 |
| TKTL1 | -0.482430831 | 7.928584049 | -2.698885925 | 0.016218118 | 0.311131654 | -2.946486878 |
| GPC3 | 0.817482702 | 6.549048536 | 2.698852675 | 0.016219209 | 0.311131654 | -2.946542375 |
| ZNF79 | -0.350208258 | 5.320798983 | -2.698739856 | 0.016222909 | 0.311131654 | -2.946730678 |
| SMCHD1 | 0.493419506 | 11.01649125 | 2.698706686 | 0.016223997 | 0.311131654 | -2.94678604 |
| KCNJ15 | 1.032843955 | 8.720626802 | 2.697901542 | 0.016250431 | 0.311466597 | -2.948129817 |
| lnc-ZNF526-1 | -0.628060034 | 2.770248649 | -2.696565786 | 0.016294377 | 0.311799897 | -2.950358947 |
| C19orf68 | -0.269592576 | 8.585805133 | -2.696561244 | 0.016294527 | 0.311799897 | -2.950366525 |
| HYAL3 | 0.414672676 | 5.183544668 | 2.696509197 | 0.016296242 | 0.311799897 | -2.950453377 |
| ACBD6 | -0.212177237 | 8.629037801 | -2.696258866 | 0.016304491 | 0.311799897 | -2.950871098 |
| CACNA1E | 0.918803816 | 4.83096964 | 2.696009595 | 0.01631271 | 0.311799897 | -2.95128704 |
| CCT7 | -0.45080701 | 10.12512193 | -2.695660603 | 0.016324223 | 0.31184833 | -2.951869362 |
| MBOAT1 | 0.380973025 | 8.513474444 | 2.694649767 | 0.016357614 | 0.312314431 | -2.953555914 |
| PTCSC3 | -0.507302284 | 4.850845859 | -2.694308205 | 0.016368912 | 0.312358421 | -2.954125764 |
| DDHD2 | -0.370664291 | 5.902234042 | -2.692984479 | 0.016412768 | 0.313023311 | -2.956334034 |
| lnc-EBPL-1 | 0.407539895 | 0.849242865 | 2.691589576 | 0.016459105 | 0.313334281 | -2.95866073 |
| RN7SL1 | 0.404546729 | 15.18936832 | 2.691558002 | 0.016460156 | 0.313334281 | -2.958713392 |
| TRIM25 | 0.63666523 | 12.22772664 | 2.691421821 | 0.016464686 | 0.313334281 | -2.958940524 |
| ZBTB40 | -0.415459464 | 8.892526982 | -2.691407542 | 0.016465162 | 0.313334281 | -2.958964339 |
| TM7SF3 | -0.28640084 | 8.622398587 | -2.690989313 | 0.016479084 | 0.313427488 | -2.95966187 |
| DCAF5 | -0.283320136 | 7.254175477 | -2.690117778 | 0.016508133 | 0.313808141 | -2.961115339 |
| FXYD6 | 0.466667227 | 4.957543134 | 2.689787908 | 0.016519141 | 0.313845611 | -2.961665435 |
| PRRC1 | -0.348107264 | 7.458499628 | -2.689507011 | 0.01652852 | 0.313852113 | -2.962133848 |
| LOC101929066 | 0.539476868 | 1.963202089 | 2.68888794 | 0.016549209 | 0.314071449 | -2.96316614 |
| ZNF816-ZNF321P | -1.312645925 | 2.7370241 | -2.688620406 | 0.016558158 | 0.314071449 | -2.963612229 |
| EPS8L1 | -0.485673632 | 5.861429326 | -2.688304346 | 0.016568736 | 0.314100538 | -2.964139217 |
| NOG | -1.207103154 | 7.063841782 | -2.687901127 | 0.01658224 | 0.314185042 | -2.964811505 |
| OR5K1 | -0.505142635 | 1.028770538 | -2.686974676 | 0.016613307 | 0.314185614 | -2.966356077 |
| PRF1 | -0.751283567 | 8.540676221 | -2.686875746 | 0.016616628 | 0.314185614 | -2.966521005 |
| LRRC42 | 0.648859698 | 2.77291851 | 2.686412388 | 0.01663219 | 0.314185614 | -2.96729345 |
| lnc-DOPEY1-1 | 0.382975444 | 0.787595827 | 2.686028029 | 0.01664511 | 0.314185614 | -2.967934173 |
| lnc-VSIG2-1 | -0.754470528 | 2.812825316 | -2.685299535 | 0.016669624 | 0.314185614 | -2.969148497 |
| XLOC_l2_014011 | 1.118489064 | 1.712661357 | 2.685042417 | 0.016678284 | 0.314185614 | -2.969577065 |
| lnc-ZBTB25-1 | -0.872960109 | 4.35870196 | -2.68469697 | 0.016689927 | 0.314185614 | -2.970152843 |
| RHBDD3 | -0.170576603 | 7.481199323 | -2.68467202 | 0.016690768 | 0.314185614 | -2.970194427 |
| ATAD2 | -0.530523538 | 6.6907304 | -2.684598903 | 0.016693233 | 0.314185614 | -2.970316294 |
| SMARCA4 | -0.364544152 | 10.48052847 | -2.684562485 | 0.016694461 | 0.314185614 | -2.970376992 |
| LOC101929422 | -0.601272167 | 2.905637896 | -2.684274311 | 0.016704182 | 0.314185614 | -2.970857288 |
| PIN1 | -0.240890354 | 10.06280425 | -2.684015146 | 0.016712929 | 0.314185614 | -2.971289224 |
| AGO4 | 0.54479729 | 7.855170283 | 2.683838684 | 0.016718887 | 0.314185614 | -2.971583316 |
| LMO2 | 0.513909088 | 10.5601437 | 2.683663225 | 0.016724813 | 0.314185614 | -2.971875732 |
| BCDIN3D | -0.374603612 | 7.5710153 | -2.683612261 | 0.016726535 | 0.314185614 | -2.971960667 |
| LOC102723766 | 0.77599727 | 3.996180696 | 2.683259713 | 0.01673845 | 0.314185614 | -2.972548196 |
| ANO2 | 0.847931643 | 1.938270377 | 2.683143376 | 0.016742383 | 0.314185614 | -2.972742068 |
| SLC4A4 | -1.183972246 | 3.75102056 | -2.682810859 | 0.016753631 | 0.314185614 | -2.973296191 |
| POMT1 | -0.295669734 | 8.763845229 | -2.682795419 | 0.016754154 | 0.314185614 | -2.97332192 |
| FBXW4 | -0.221743099 | 8.212330287 | -2.681017014 | 0.016814437 | 0.315094866 | -2.976285215 |
| ZNF582-AS1 | -0.550193358 | 5.769394768 | -2.680830074 | 0.016820786 | 0.315094866 | -2.976596675 |
| BRD2 | -0.311806165 | 12.16565836 | -2.679829273 | 0.016854814 | 0.315431872 | -2.978264009 |
| EMX2 | -0.456234006 | 1.019484285 | -2.679616292 | 0.016862064 | 0.315431872 | -2.978618813 |
| LOC100128697 | 0.9287218 | 5.795562489 | 2.679500034 | 0.016866023 | 0.315431872 | -2.978812484 |
| FGR | 0.383214511 | 14.22582548 | 2.678428131 | 0.016902566 | 0.315945172 | -2.980598026 |
| SLC16A7 | -0.336047308 | 5.007346369 | -2.678007127 | 0.01691694 | 0.316043748 | -2.981299268 |
| CHD6 | -0.449784928 | 7.603720502 | -2.677446352 | 0.016936104 | 0.316157255 | -2.982233271 |
| lnc-GRID2IP-1 | -0.90874638 | 1.740593386 | -2.677296682 | 0.016941222 | 0.316157255 | -2.982482546 |
| lnc-ZNF484-1 | -0.562138578 | 3.997908967 | -2.676098762 | 0.016982242 | 0.31675256 | -2.984477544 |
| lnc-TBK1-1 | 0.620109259 | 6.945908551 | 2.675741485 | 0.016994495 | 0.316810949 | -2.985072499 |
| VPS37D | -0.356530494 | 4.460816693 | -2.67496305 | 0.01702122 | 0.317077621 | -2.986368715 |
| lnc-C1QTNF1-1 | 0.62146095 | 3.535770064 | 2.674666685 | 0.017031405 | 0.317077621 | -2.98686218 |
| KANK1 | -1.289704222 | 5.510657017 | -2.674272236 | 0.017044971 | 0.317077621 | -2.987518941 |
| RPL28 | -0.35823076 | 14.76981267 | -2.674262132 | 0.017045319 | 0.317077621 | -2.987535763 |
| CA14 | 0.72851531 | 2.367339276 | 2.673747878 | 0.01706302 | 0.317167274 | -2.988391958 |
| ARHGAP5-AS1 | -0.904417574 | 2.37133926 | -2.673591614 | 0.017068403 | 0.317167274 | -2.988652117 |
| ANKH | -0.624331586 | 5.76109282 | -2.672712463 | 0.017098716 | 0.317499039 | -2.990115709 |
| NSMF | -0.43321324 | 5.92689485 | -2.672491258 | 0.017106351 | 0.317499039 | -2.990483944 |
| MRVI1 | 1.162945493 | 9.665628703 | 2.672278925 | 0.017113683 | 0.317499039 | -2.990837404 |
| RILPL2 | 0.227205969 | 11.58622987 | 2.671717891 | 0.01713307 | 0.317689018 | -2.991771292 |
| lnc-PSMA2-2 | 0.394646946 | 3.159452922 | 2.671159587 | 0.017152384 | 0.317762093 | -2.992700581 |
| PIGU | -0.662548762 | 4.345627618 | -2.670466022 | 0.017176407 | 0.317762093 | -2.993854936 |
| YARS2 | -0.429043408 | 6.546714045 | -2.670123174 | 0.017188294 | 0.317762093 | -2.994425533 |
| CDHR2 | 0.506983929 | 3.981847076 | 2.67006233 | 0.017190404 | 0.317762093 | -2.994526793 |
| PTPLAD1 | -0.481897629 | 8.963827723 | -2.670022247 | 0.017191795 | 0.317762093 | -2.994593502 |
| DNM1P46 | -0.580218885 | 5.335162843 | -2.670018981 | 0.017191908 | 0.317762093 | -2.994598936 |
| ZNHIT1 | -0.227710636 | 11.15888606 | -2.669719999 | 0.017202283 | 0.31777736 | -2.995096507 |
| GORASP2 | -0.386945894 | 8.775141834 | -2.668759477 | 0.017235654 | 0.31777736 | -2.996694919 |
| lnc-GPX1-1 | 0.485231635 | 6.25843168 | 2.667681303 | 0.017273187 | 0.31777736 | -2.998488929 |
| ILVBL | -0.254960783 | 7.185954676 | -2.667395594 | 0.017283146 | 0.31777736 | -2.998964296 |
| lnc-RPS21-2 | 0.959689709 | 3.316252698 | 2.666994502 | 0.017297136 | 0.31777736 | -2.999631614 |
| CEBPE | 0.655879623 | 8.349565203 | 2.666729312 | 0.017306392 | 0.31777736 | -3.000072811 |
| ZXDB | -0.44329897 | 7.650291689 | -2.666504887 | 0.017314229 | 0.31777736 | -3.000446176 |
| PIH1D2 | 1.042438013 | 2.205709776 | 2.666413162 | 0.017317433 | 0.31777736 | -3.000598772 |
| LOC100996681 | 0.517248613 | 3.438264274 | 2.666226628 | 0.017323951 | 0.31777736 | -3.00090909 |
| ZNF518B | -0.27632463 | 7.306204445 | -2.666035594 | 0.017330628 | 0.31777736 | -3.001226889 |
| C1QBP | -0.587601232 | 10.85539123 | -2.66586424 | 0.017336619 | 0.31777736 | -3.001511945 |
| FKBP14 | -0.497771074 | 4.767698502 | -2.665447436 | 0.017351201 | 0.31777736 | -3.002205293 |
| ZNF831 | -0.548267401 | 8.977140332 | -2.66535562 | 0.017354415 | 0.31777736 | -3.002358024 |
| RALA | -0.450772052 | 7.118339808 | -2.66535291 | 0.01735451 | 0.31777736 | -3.002362531 |
| CACTIN | -0.215412768 | 8.548311533 | -2.665034912 | 0.017365645 | 0.31777736 | -3.002891492 |
| NIPAL3 | -0.374162675 | 8.76910735 | -2.664887996 | 0.017370792 | 0.31777736 | -3.003135867 |
| lnc-AP000790.1-2 | 1.013330325 | 1.504641031 | 2.664734404 | 0.017376174 | 0.31777736 | -3.003391345 |
| LOC101928227 | 1.311455176 | 3.677487773 | 2.664397088 | 0.017388 | 0.31777736 | -3.003952402 |
| ITPA | -0.371560705 | 9.257503094 | -2.66399681 | 0.017402044 | 0.31777736 | -3.004618159 |
| ABLIM1 | -0.654441362 | 11.20885987 | -2.66332966 | 0.017425475 | 0.31777736 | -3.005727724 |
| HSD17B11 | 0.359613507 | 9.907477628 | 2.663169829 | 0.017431093 | 0.31777736 | -3.005993535 |
| CTD-2151A2.1 | -0.684189179 | 1.997476049 | -2.663090721 | 0.017433874 | 0.31777736 | -3.006125095 |
| ARL11 | 0.74951553 | 6.658829684 | 2.662152505 | 0.017466892 | 0.31777736 | -3.007685314 |
| XLOC_l2_007270 | -0.902470512 | 2.09518185 | -2.662090202 | 0.017469086 | 0.31777736 | -3.007788915 |
| POM121L1P | 0.834077025 | 1.76382006 | 2.661495407 | 0.017490052 | 0.31777736 | -3.008777951 |
| LOC101927851 | 0.690886711 | 7.895418629 | 2.6606759 | 0.017518978 | 0.31777736 | -3.010140542 |
| ANGEL1 | -0.376148201 | 4.743678723 | -2.660642507 | 0.017520158 | 0.31777736 | -3.010196061 |
| ZCCHC14 | -0.486921678 | 7.135808599 | -2.66053747 | 0.017523869 | 0.31777736 | -3.010370697 |
| lnc-AK5-1 | -1.004548101 | 4.098429209 | -2.660413246 | 0.017528259 | 0.31777736 | -3.01057723 |
| SNORA1 | 0.543724225 | 6.148029668 | 2.660260128 | 0.017533671 | 0.31777736 | -3.010831798 |
| FANCF | -0.384458606 | 8.316547762 | -2.659936063 | 0.017545132 | 0.31777736 | -3.011370564 |
| NARFL | -0.240596404 | 6.50395609 | -2.658820742 | 0.01758463 | 0.31777736 | -3.013224668 |
| UBASH3A | -0.528837757 | 7.227935577 | -2.658765895 | 0.017586575 | 0.31777736 | -3.01331584 |
| LOC102723862 | 0.690656283 | 2.657823082 | 2.658233338 | 0.017605467 | 0.31777736 | -3.01420108 |
| LINC00877 | 0.864078176 | 7.402977823 | 2.658139105 | 0.017608812 | 0.31777736 | -3.014357713 |
| lnc-CLCN6-1 | 0.412872134 | 6.889154121 | 2.657417519 | 0.017634446 | 0.31777736 | -3.015557072 |
| TLE4 | 0.44712198 | 9.116272841 | 2.657384021 | 0.017635637 | 0.31777736 | -3.015612747 |
| POT1-AS1 | -0.687663993 | 2.233410525 | -2.657181439 | 0.01764284 | 0.31777736 | -3.015949444 |
| C4orf47 | -0.451594722 | 1.055058579 | -2.656507178 | 0.017666836 | 0.31777736 | -3.017070033 |
| XLOC_l2_005175 | 0.583825965 | 4.337628484 | 2.656390994 | 0.017670974 | 0.31777736 | -3.017263118 |
| NFKBIZ | 0.48657561 | 6.871365963 | 2.656383552 | 0.017671239 | 0.31777736 | -3.017275485 |
| CDR2 | -0.682062611 | 5.39819001 | -2.656162457 | 0.017679116 | 0.31777736 | -3.017642911 |
| FAM114A2 | -0.440147512 | 5.124962094 | -2.656161725 | 0.017679142 | 0.31777736 | -3.017644129 |
| ZNF671 | -0.366515242 | 8.572282315 | -2.656035999 | 0.017683623 | 0.31777736 | -3.017853063 |
| MESDC2 | -0.337003865 | 5.602452439 | -2.65595149 | 0.017686636 | 0.31777736 | -3.017993499 |
| KAT6B | -0.396923421 | 5.487405144 | -2.655898358 | 0.01768853 | 0.31777736 | -3.018081794 |
| LOC101930017 | 0.963635383 | 1.490243291 | 2.655705372 | 0.017695412 | 0.31777736 | -3.018402493 |
| JUN | -0.824420633 | 4.7026945 | -2.655307006 | 0.017709626 | 0.31777736 | -3.019064463 |
| C16orf95 | -0.31171824 | 5.889692704 | -2.655121022 | 0.017716266 | 0.31777736 | -3.019373506 |
| LOC100505501 | -0.717009986 | 5.070404799 | -2.655058858 | 0.017718486 | 0.31777736 | -3.019476801 |
| COLGALT2 | -1.344186908 | 3.720554822 | -2.65445131 | 0.017740196 | 0.31777736 | -3.020486293 |
| lnc-MPZL1-1 | 0.957155942 | 1.695305693 | 2.654441727 | 0.017740538 | 0.31777736 | -3.020502216 |
| EVC2 | -0.543214687 | 1.190317924 | -2.654247926 | 0.017747469 | 0.31777736 | -3.020824218 |
| FBXL22 | -0.871025136 | 3.478583717 | -2.653865328 | 0.017761159 | 0.31777736 | -3.021459889 |
| DKFZp667F0711 | 1.114831919 | 5.516781036 | 2.653443853 | 0.017776252 | 0.31777736 | -3.022160124 |
| PLXDC1 | -0.991577838 | 7.306778589 | -2.653166312 | 0.017786197 | 0.31777736 | -3.02262121 |
| AARS | -0.671755579 | 4.688631952 | -2.653066794 | 0.017789765 | 0.31777736 | -3.022786539 |
| lnc-MYH13-3 | 0.426048281 | 3.526699544 | 2.652894672 | 0.017795936 | 0.31777736 | -3.023072479 |
| lnc-DBT-1 | -0.65379392 | 2.06481908 | -2.652735609 | 0.017801642 | 0.31777736 | -3.023336722 |
| GRIN3A | 1.148367545 | 3.146419551 | 2.65198609 | 0.017828549 | 0.31777736 | -3.024581794 |
| LOC100132272 | 1.095953177 | 2.305675891 | 2.651886615 | 0.017832123 | 0.31777736 | -3.02474703 |
| MED1 | -0.289968334 | 4.963433032 | -2.651763564 | 0.017836545 | 0.31777736 | -3.024951426 |
| GMPS | -0.437492429 | 8.568925222 | -2.650647372 | 0.017876705 | 0.31777736 | -3.026805371 |
| TRGV7 | -0.651331517 | 7.415022995 | -2.64972593 | 0.017909924 | 0.31777736 | -3.02833568 |
| CEACAM7 | 1.172780818 | 2.389944432 | 2.649385853 | 0.0179222 | 0.31777736 | -3.028900434 |
| lnc-RNLS-1 | 0.970885501 | 6.908483215 | 2.649324194 | 0.017924426 | 0.31777736 | -3.029002826 |
| SLC12A8 | -0.40316817 | 1.044076599 | -2.649110458 | 0.017932146 | 0.31777736 | -3.029357757 |
| PIAS1 | 0.403587683 | 7.656871813 | 2.649099066 | 0.017932558 | 0.31777736 | -3.029376675 |
| TRRAP | -0.308780811 | 6.907640973 | -2.648987463 | 0.01793659 | 0.31777736 | -3.029561999 |
| lnc-USP47-2 | -0.829345135 | 2.417380441 | -2.648643043 | 0.01794904 | 0.31777736 | -3.030133919 |
| CIB3 | 0.694393244 | 3.894731616 | 2.64830835 | 0.017961146 | 0.31777736 | -3.030689667 |
| ZNF382 | -0.702138903 | 3.355844902 | -2.647489451 | 0.017990799 | 0.31777736 | -3.032049341 |
| SBNO2 | 0.676924034 | 10.9588506 | 2.647155079 | 0.018002921 | 0.31777736 | -3.032604487 |
| ZNF285 | -1.056780958 | 1.897008008 | -2.647021805 | 0.018007754 | 0.31777736 | -3.03282575 |
| B3GAT3 | -0.257239404 | 8.441521633 | -2.647011377 | 0.018008132 | 0.31777736 | -3.032843063 |
| UHMK1 | -0.194819958 | 8.262135368 | -2.64693686 | 0.018010836 | 0.31777736 | -3.032966778 |
| SLC29A4 | 1.182910169 | 1.765669699 | 2.64675244 | 0.018017527 | 0.31777736 | -3.033272947 |
| lnc-IER5L-1 | 1.054972757 | 2.133393151 | 2.646650428 | 0.01802123 | 0.31777736 | -3.033442303 |
| EGLN1 | 0.650292037 | 9.11698293 | 2.646435984 | 0.018029016 | 0.31777736 | -3.033798307 |
| VPS13A | -0.388610857 | 8.075037309 | -2.646263692 | 0.018035273 | 0.31777736 | -3.034084327 |
| FOSB | -0.752312607 | 3.848320616 | -2.645855876 | 0.018050093 | 0.31777736 | -3.034761318 |
| TOX | -0.67380208 | 5.130176133 | -2.645753484 | 0.018053816 | 0.31777736 | -3.034931289 |
| ATRNL1 | 0.748372106 | 3.056052412 | 2.645702223 | 0.01805568 | 0.31777736 | -3.035016381 |
| DSE | 0.31744529 | 9.181108756 | 2.645229003 | 0.018072897 | 0.31777736 | -3.035801894 |
| LAMC1 | -0.677978582 | 3.705893863 | -2.644940211 | 0.018083412 | 0.31777736 | -3.03628125 |
| CPT1B | 0.674095058 | 6.895432608 | 2.644751124 | 0.018090299 | 0.31777736 | -3.036595101 |
| ITFG2 | -0.351470806 | 7.550041349 | -2.644281314 | 0.018107424 | 0.31777736 | -3.037374874 |
| TCEANC | -0.227862081 | 7.228507682 | -2.643735883 | 0.018127324 | 0.31777736 | -3.03828011 |
| LOC102724156 | 1.10461382 | 6.480676041 | 2.643322597 | 0.018142416 | 0.31777736 | -3.038965994 |
| lnc-CCNB2-1 | 0.597598688 | 1.714888384 | 2.642996257 | 0.018154343 | 0.31777736 | -3.03950756 |
| lnc-SIK1-1 | 0.303780339 | 1.163620728 | 2.642825989 | 0.018160568 | 0.31777736 | -3.039790117 |
| TOMM34 | -0.315496154 | 8.254802833 | -2.642760711 | 0.018162955 | 0.31777736 | -3.039898442 |
| TMEM19 | -0.499851124 | 7.170979975 | -2.642245491 | 0.018181808 | 0.31777736 | -3.040753399 |
| lnc-KIAA1324L-1 | -0.535482199 | 1.184481337 | -2.642078604 | 0.018187919 | 0.31777736 | -3.041030322 |
| ZNF570 | -0.538158792 | 3.471283373 | -2.642074092 | 0.018188084 | 0.31777736 | -3.041037808 |
| SMARCC1 | -0.24399749 | 8.236726395 | -2.641733731 | 0.018200553 | 0.31777736 | -3.041602567 |
| lnc-ZNF8-3 | -0.610877379 | 3.739852559 | -2.641637869 | 0.018204066 | 0.31777736 | -3.041761626 |
| CMTM6 | 0.394239967 | 10.37005821 | 2.641399738 | 0.018212796 | 0.31777736 | -3.04215674 |
| SPATA5 | -0.814779561 | 2.875677056 | -2.640926907 | 0.018230143 | 0.31777736 | -3.042941241 |
| GPR18 | -0.460624219 | 8.209590653 | -2.640878918 | 0.018231904 | 0.31777736 | -3.043020859 |
| HIST1H2BG | 0.55560417 | 5.945141021 | 2.64082493 | 0.018233886 | 0.31777736 | -3.043110431 |
| LINC00630 | -0.773688602 | 2.849901148 | -2.640818409 | 0.018234125 | 0.31777736 | -3.043121251 |
| ZFP69 | -0.555213736 | 4.53349503 | -2.640498876 | 0.018245859 | 0.31777736 | -3.043651378 |
| RAB13 | 0.513850596 | 3.363437881 | 2.640363731 | 0.018250825 | 0.31777736 | -3.043875587 |
| ARL1 | -0.379341387 | 8.359280804 | -2.640228499 | 0.018255794 | 0.31777736 | -3.044099938 |
| PFKFB4 | 0.69715665 | 8.720494391 | 2.640061659 | 0.018261927 | 0.31777736 | -3.044376721 |
| ZMAT4 | -1.317828898 | 4.213266527 | -2.639830943 | 0.018270411 | 0.31777736 | -3.044759466 |
| MEGF8 | -0.420839503 | 7.052697413 | -2.639819332 | 0.018270838 | 0.31777736 | -3.044778728 |
| LOC101929147 | -0.537859327 | 3.518837983 | -2.639399308 | 0.018286294 | 0.31777736 | -3.045475496 |
| MEI1 | -0.526026251 | 10.39606337 | -2.639307671 | 0.018289667 | 0.31777736 | -3.045627507 |
| LINC01233 | 0.414712444 | 4.416108665 | 2.639302512 | 0.018289857 | 0.31777736 | -3.045636065 |
| lnc-SMNDC1-1 | 0.961177756 | 1.560088058 | 2.639171934 | 0.018294666 | 0.31777736 | -3.045852669 |
| lnc-UAP1L1-1 | 0.318329417 | 6.302475608 | 2.639090448 | 0.018297667 | 0.31777736 | -3.045987838 |
| PITX1 | 0.935355196 | 1.72119975 | 2.63888892 | 0.018305091 | 0.31777736 | -3.046322125 |
| RNF113A | -0.333743516 | 10.38036872 | -2.638862439 | 0.018306067 | 0.31777736 | -3.046366051 |
| MRPL10 | -0.312888421 | 9.268858454 | -2.638635799 | 0.018314421 | 0.31777736 | -3.046741984 |
| CDC42EP4 | 0.521425801 | 4.270753908 | 2.638600048 | 0.018315739 | 0.31777736 | -3.046801284 |
| RAB40B | -0.661066469 | 4.34886199 | -2.638541021 | 0.018317915 | 0.31777736 | -3.046899191 |
| PLEKHG4 | -0.479622816 | 6.028687708 | -2.638506974 | 0.018319171 | 0.31777736 | -3.046955665 |
| LOC100507191 | 0.851925092 | 4.486752264 | 2.638425931 | 0.01832216 | 0.31777736 | -3.047090089 |
| RABL2A | -0.395554629 | 9.829997999 | -2.638403853 | 0.018322974 | 0.31777736 | -3.047126709 |
| LOC101929115 | -0.465968158 | 4.251397265 | -2.638318394 | 0.018326126 | 0.31777736 | -3.047268456 |
| SDR42E1 | -0.812193571 | 3.531139979 | -2.638261825 | 0.018328213 | 0.31777736 | -3.047362283 |
| IPO5 | -0.406542842 | 5.993163364 | -2.638053341 | 0.018335906 | 0.31777736 | -3.047708079 |
| COQ3 | -0.583015056 | 3.555798796 | -2.638037886 | 0.018336477 | 0.31777736 | -3.047733712 |
| KLHL36 | -0.287029774 | 6.392291237 | -2.637458503 | 0.018357873 | 0.317989491 | -3.048694643 |
| IFITM2 | 0.640029232 | 15.03565963 | 2.637047347 | 0.018373072 | 0.318087776 | -3.049376526 |
| QPCT | 0.839435058 | 9.67243319 | 2.636809614 | 0.018381865 | 0.318087776 | -3.049770781 |
| OIP5 | -1.098628333 | 4.244742487 | -2.63655259 | 0.018391376 | 0.318093874 | -3.050197016 |
| KCNQ5-IT1 | -0.570084698 | 6.677792398 | -2.635649172 | 0.018424846 | 0.318151695 | -3.051695102 |
| ATG3 | 0.333003725 | 8.736881785 | 2.635422637 | 0.018433248 | 0.318151695 | -3.05207073 |
| PRDX1 | -0.497685324 | 11.51274063 | -2.635182201 | 0.018442169 | 0.318151695 | -3.052469396 |
| LINC01499 | 0.551812528 | 1.445433287 | 2.635180125 | 0.018442246 | 0.318151695 | -3.052472838 |
| GBF1 | -0.430383912 | 8.440869778 | -2.635078772 | 0.018446008 | 0.318151695 | -3.052640888 |
| LOC643802 | 0.688783875 | 8.112909599 | 2.634769379 | 0.018457496 | 0.318151695 | -3.05315387 |
| TMEM161A | -0.343454703 | 9.181580381 | -2.634733088 | 0.018458844 | 0.318151695 | -3.053214042 |
| HNRNPKP3 | 0.384117343 | 8.320057844 | 2.634369731 | 0.018472346 | 0.318217861 | -3.053816473 |
| PELI1 | 0.524915144 | 10.80504129 | 2.633811798 | 0.018493097 | 0.318217861 | -3.054741457 |
| AKAP12 | -0.93847594 | 4.419154668 | -2.633809326 | 0.018493189 | 0.318217861 | -3.054745555 |
| lnc-STK35-2 | 1.017762621 | 2.775799136 | 2.633586818 | 0.018501471 | 0.318217861 | -3.05511443 |
| AVEN | -0.410652056 | 6.968728977 | -2.633398131 | 0.018508496 | 0.318217861 | -3.055427229 |
| ARRB2 | 0.42021191 | 12.01809622 | 2.63240258 | 0.018545608 | 0.318381651 | -3.057077515 |
| POLA1 | -0.415068893 | 6.50484634 | -2.632252218 | 0.01855122 | 0.318381651 | -3.057326747 |
| HSPA1A | 0.55834615 | 11.79981602 | 2.631786967 | 0.018568593 | 0.318381651 | -3.0580979 |
| SAMSN1 | 0.485771709 | 7.048236692 | 2.631331656 | 0.01858561 | 0.318381651 | -3.058852539 |
| PDZRN3 | -0.833317106 | 1.992989415 | -2.631261156 | 0.018588246 | 0.318381651 | -3.058969384 |
| UMPS | -0.405035828 | 8.285875638 | -2.631054145 | 0.018595989 | 0.318381651 | -3.059312471 |
| SMAD7 | -0.506124282 | 6.659029209 | -2.631011865 | 0.01859757 | 0.318381651 | -3.059382543 |
| lnc-BAMBI-1 | -0.377602952 | 1.014501247 | -2.631008807 | 0.018597685 | 0.318381651 | -3.059387611 |
| lnc-SUSD2-1 | -0.370962021 | 3.436534319 | -2.630917727 | 0.018601093 | 0.318381651 | -3.059538559 |
| PUM1 | -0.227093457 | 10.48220411 | -2.630548751 | 0.018614905 | 0.318381651 | -3.060150049 |
| LOC101928670 | 0.933363652 | 7.393016255 | 2.630298566 | 0.018624276 | 0.318381651 | -3.060564658 |
| FAM210A | -0.960342093 | 4.659111125 | -2.630033047 | 0.018634227 | 0.318381651 | -3.061004664 |
| SLC27A5 | -0.565499765 | 7.120758913 | -2.629779019 | 0.018643751 | 0.318381651 | -3.061425616 |
| LINC00598 | 0.805618463 | 2.1264764 | 2.629709309 | 0.018646366 | 0.318381651 | -3.061541132 |
| TMEM173 | -0.349477993 | 10.78693057 | -2.629356677 | 0.018659597 | 0.318451004 | -3.062125459 |
| TNFRSF10C | 0.75400678 | 12.81693935 | 2.62838329 | 0.018696166 | 0.318848656 | -3.063738283 |
| FPR2 | 0.837377241 | 9.985379603 | 2.62824788 | 0.018701259 | 0.318848656 | -3.063962633 |
| lnc-MYOC-2 | -1.029126108 | 1.654238254 | -2.627463627 | 0.01873078 | 0.319195286 | -3.065261931 |
| DIEXF | -0.522673669 | 5.091761687 | -2.627084143 | 0.018745081 | 0.319282327 | -3.065890595 |
| CDK5RAP1 | -0.367083647 | 9.433237426 | -2.626527801 | 0.018766066 | 0.319424505 | -3.066812198 |
| BCKDHB | -0.527931243 | 5.987688657 | -2.625993811 | 0.018786229 | 0.319424505 | -3.067696721 |
| ENPP4 | -0.662460159 | 8.185800602 | -2.625852499 | 0.018791568 | 0.319424505 | -3.067930787 |
| KCNRG | 0.450257006 | 4.943073278 | 2.625818423 | 0.018792856 | 0.319424505 | -3.067987229 |
| UNG | -0.470012808 | 9.080926942 | -2.62531193 | 0.018812006 | 0.319424505 | -3.068826139 |
| lnc-SNX33-1 | -0.777538618 | 3.386349876 | -2.62506455 | 0.018821366 | 0.319424505 | -3.06923586 |
| RBM3 | -0.473975399 | 9.623378413 | -2.624966268 | 0.018825086 | 0.319424505 | -3.069398635 |
| STARD4-AS1 | -0.529722248 | 1.270365089 | -2.62491552 | 0.018827007 | 0.319424505 | -3.069482684 |
| PARD6B | -0.904526375 | 3.803313813 | -2.624465143 | 0.018844065 | 0.319459212 | -3.070228576 |
| SLC9A1 | 0.381546781 | 4.732024259 | 2.624375813 | 0.01884745 | 0.319459212 | -3.070376514 |
| APOA1BP | -0.385423837 | 8.120701311 | -2.623782851 | 0.018869934 | 0.319684293 | -3.071358482 |
| LOC100507520 | 0.404999198 | 5.923668467 | 2.622661706 | 0.018912516 | 0.320106548 | -3.07321496 |
| LINC00595 | 0.411075246 | 4.691620518 | 2.622022099 | 0.01893685 | 0.320106548 | -3.074273965 |
| PDHB | -0.411683255 | 9.186243782 | -2.621956258 | 0.018939357 | 0.320106548 | -3.074382975 |
| ACKR3 | -0.624449109 | 6.759130871 | -2.621591247 | 0.018953259 | 0.320106548 | -3.074987291 |
| EBP | -0.407883568 | 9.120844304 | -2.621393643 | 0.01896079 | 0.320106548 | -3.075314435 |
| FAM50B | -0.65741754 | 6.293398635 | -2.621314912 | 0.018963791 | 0.320106548 | -3.075444776 |
| IMPDH1 | 0.529835513 | 13.09640143 | 2.620474299 | 0.018995862 | 0.320106548 | -3.076836364 |
| NFASC | 0.885573203 | 3.909622455 | 2.619888707 | 0.019018234 | 0.320106548 | -3.077805702 |
| TRAF1 | -0.448241099 | 7.531170337 | -2.619804941 | 0.019021437 | 0.320106548 | -3.077944356 |
| FCAR | 1.134160764 | 7.32930921 | 2.619700907 | 0.019025415 | 0.320106548 | -3.078116556 |
| TMEM253 | 1.179744114 | 1.915660325 | 2.619652274 | 0.019027274 | 0.320106548 | -3.078197053 |
| FCGR1A | 1.436070649 | 5.118661786 | 2.619581895 | 0.019029966 | 0.320106548 | -3.078313545 |
| SLC25A37 | 0.579861599 | 12.56765563 | 2.619499189 | 0.01903313 | 0.320106548 | -3.07845044 |
| RDH13 | -0.493253884 | 4.624839133 | -2.619453397 | 0.019034882 | 0.320106548 | -3.078526233 |
| MIPEP | -0.641197037 | 5.45640043 | -2.619328157 | 0.019039674 | 0.320106548 | -3.078733526 |
| RNF4 | -0.267425989 | 9.78976729 | -2.618905313 | 0.019055861 | 0.320106548 | -3.079433381 |
| LOC101928525 | 0.82292524 | 1.422113226 | 2.618176048 | 0.019083811 | 0.320106548 | -3.080640318 |
| LOC101930457 | 0.197393539 | 0.931737059 | 2.618015658 | 0.019089963 | 0.320106548 | -3.08090575 |
| CCNB1 | -0.859189751 | 5.342396651 | -2.617905849 | 0.019094177 | 0.320106548 | -3.081087473 |
| LOC100506585 | 0.617489901 | 6.824655434 | 2.617627097 | 0.019104876 | 0.320106548 | -3.081548768 |
| C3orf22 | 0.537267183 | 2.993616175 | 2.617505314 | 0.019109552 | 0.320106548 | -3.081750299 |
| lnc-AEBP1-1 | 0.56288521 | 6.909589965 | 2.61738106 | 0.019114324 | 0.320106548 | -3.081955914 |
| SFN | 0.564567293 | 4.147634584 | 2.61690584 | 0.019132587 | 0.320106548 | -3.082742281 |
| LRWD1 | 0.621598288 | 9.693571585 | 2.61690573 | 0.019132591 | 0.320106548 | -3.082742462 |
| CEP76 | -0.628688325 | 4.000011104 | -2.61688028 | 0.01913357 | 0.320106548 | -3.082784574 |
| UNC119 | 0.307316853 | 7.28098027 | 2.616295944 | 0.019156049 | 0.320106548 | -3.08375144 |
| H1FX | -0.324573812 | 7.274277077 | -2.615915563 | 0.019170696 | 0.320106548 | -3.084380799 |
| lnc-orphan.1-1 | -0.701746548 | 1.969005982 | -2.615865757 | 0.019172615 | 0.320106548 | -3.084463204 |
| TTC7B | 0.680126613 | 6.600678363 | 2.61554745 | 0.019184881 | 0.320106548 | -3.084989834 |
| LOC646014 | 0.487609039 | 5.237674439 | 2.615334184 | 0.019193104 | 0.320106548 | -3.085342667 |
| ITM2B | 0.431715135 | 13.78077056 | 2.615149858 | 0.019200213 | 0.320106548 | -3.085647613 |
| ZNF345 | -0.433584796 | 7.020840651 | -2.615125779 | 0.019201142 | 0.320106548 | -3.085687449 |
| PRPF19 | -0.453463015 | 11.56814194 | -2.614947961 | 0.019208003 | 0.320106548 | -3.08598162 |
| LOC101928408 | 0.966261934 | 3.11234299 | 2.614822562 | 0.019212843 | 0.320106548 | -3.08618907 |
| TMEM190 | 0.783634621 | 1.623889846 | 2.614681345 | 0.019218295 | 0.320106548 | -3.086422686 |
| lnc-OR2W1-1 | -0.382607688 | 0.786591368 | -2.614096795 | 0.019240879 | 0.320106548 | -3.087389664 |
| B9D1 | -0.961871942 | 2.924451441 | -2.613629239 | 0.019258961 | 0.320106548 | -3.08816306 |
| NDFIP2 | -0.536117393 | 3.650136085 | -2.613488662 | 0.0192644 | 0.320106548 | -3.088395584 |
| PRMT7 | -0.317293787 | 9.265860117 | -2.613427955 | 0.01926675 | 0.320106548 | -3.088495997 |
| SEH1L | -0.456716667 | 6.987171176 | -2.613205909 | 0.019275346 | 0.320106548 | -3.088863266 |
| LOC727710 | 0.407552002 | 4.179338858 | 2.61310157 | 0.019279387 | 0.320106548 | -3.089035842 |
| G3BP1 | -0.404892745 | 10.4186066 | -2.612659115 | 0.01929653 | 0.320106548 | -3.089767637 |
| PGD | 0.636972828 | 8.957930367 | 2.612627943 | 0.019297739 | 0.320106548 | -3.089819193 |
| AIP | -0.285883978 | 9.655508726 | -2.61255913 | 0.019300406 | 0.320106548 | -3.089933001 |
| SERPINE2 | -1.060585231 | 3.051156556 | -2.611105541 | 0.019356842 | 0.320649581 | -3.092336865 |
| SYMPK | -0.334692693 | 10.87675809 | -2.61102886 | 0.019359823 | 0.320649581 | -3.092463664 |
| FAM126B | 0.672563196 | 9.408204119 | 2.610881246 | 0.019365564 | 0.320649581 | -3.092707755 |
| GGA2 | -0.346153058 | 8.432644419 | -2.610765191 | 0.019370078 | 0.320649581 | -3.092899658 |
| UHRF1 | -0.67925678 | 3.93844541 | -2.609034322 | 0.01943753 | 0.321176047 | -3.09576144 |
| TRMT2A | -0.373938633 | 5.742757554 | -2.608986834 | 0.019439383 | 0.321176047 | -3.095839946 |
| LOC100507175 | 0.858288471 | 2.286752015 | 2.608797492 | 0.019446776 | 0.321176047 | -3.096152964 |
| FPGT-TNNI3K | -0.527238631 | 2.954991713 | -2.608748452 | 0.019448692 | 0.321176047 | -3.096234036 |
| LOC101929648 | -0.695488131 | 1.682100289 | -2.608313638 | 0.019465681 | 0.321176047 | -3.096952835 |
| FCGR3A | 0.551529244 | 14.94397441 | 2.608297186 | 0.019466324 | 0.321176047 | -3.096980032 |
| lnc-STX17-1 | 0.937305416 | 4.14643046 | 2.608152966 | 0.019471962 | 0.321176047 | -3.097218435 |
| ZNF841 | -0.358248349 | 5.697978437 | -2.608053189 | 0.019475864 | 0.321176047 | -3.09738337 |
| PPP3CC | -0.527109722 | 9.15476261 | -2.607453575 | 0.019499327 | 0.321325543 | -3.098374515 |
| TLR8 | 0.668400439 | 8.039704632 | 2.607190479 | 0.019509631 | 0.321325543 | -3.098809384 |
| SNORD105 | -0.584590086 | 4.598407047 | -2.607112516 | 0.019512686 | 0.321325543 | -3.098938245 |
| GPRASP2 | -0.770568746 | 2.770174312 | -2.606228227 | 0.019547361 | 0.32135088 | -3.100399766 |
| NFAM1 | 0.581480593 | 11.53000988 | 2.606140481 | 0.019550805 | 0.32135088 | -3.10054478 |
| SF3A3 | -0.341462243 | 9.463551494 | -2.605909362 | 0.019559879 | 0.32135088 | -3.100926738 |
| PLD4 | -1.251528946 | 5.181660807 | -2.605782604 | 0.019564857 | 0.32135088 | -3.101136218 |
| ZNF718 | -0.336545328 | 4.833144448 | -2.605569754 | 0.01957322 | 0.32135088 | -3.10148797 |
| POLDIP2 | -0.139235707 | 8.679152102 | -2.605537801 | 0.019574476 | 0.32135088 | -3.101540774 |
| HKR1 | -0.50827894 | 5.73705881 | -2.605251517 | 0.019585729 | 0.32135088 | -3.102013863 |
| GADD45A | 0.708005769 | 6.5940561 | 2.605187489 | 0.019588247 | 0.32135088 | -3.102119668 |
| CEP128 | -0.635339212 | 5.280410274 | -2.604805019 | 0.019603293 | 0.321381434 | -3.102751679 |
| lnc-CILP-1 | -0.517294974 | 4.75939419 | -2.604669757 | 0.019608617 | 0.321381434 | -3.102975185 |
| ST6GALNAC3 | 0.87995493 | 4.917513074 | 2.603958139 | 0.019636648 | 0.321417607 | -3.104151003 |
| AZU1 | 1.484396777 | 5.000368934 | 2.603784828 | 0.019643481 | 0.321417607 | -3.104437353 |
| SLC4A7 | -0.606469528 | 6.047620752 | -2.603481889 | 0.01965543 | 0.321417607 | -3.104937864 |
| lnc-RP11-723O4.6.1-1 | 0.726959669 | 2.973139343 | 2.603306192 | 0.019662363 | 0.321417607 | -3.105228139 |
| LINC00996 | -0.805345106 | 4.556717573 | -2.603239601 | 0.019664991 | 0.321417607 | -3.105338156 |
| lnc-ALPL-1 | 1.19088699 | 4.523934011 | 2.602757208 | 0.019684041 | 0.321417607 | -3.106135099 |
| WBP2 | 0.520415233 | 8.446080042 | 2.602390982 | 0.019698516 | 0.321417607 | -3.106740097 |
| lnc-FAM49B-1 | 0.640417799 | 7.438573539 | 2.602369083 | 0.019699381 | 0.321417607 | -3.106776272 |
| PRTN3 | 0.707070339 | 3.518610054 | 2.602263022 | 0.019703575 | 0.321417607 | -3.106951479 |
| DEFB128 | 0.569040197 | 1.777927046 | 2.602235816 | 0.019704651 | 0.321417607 | -3.106996421 |
| lnc-AC136604.1-1 | 0.486960134 | 2.894981548 | 2.601865045 | 0.01971932 | 0.321417607 | -3.107608889 |
| ZFP1 | -0.61641234 | 4.842890758 | -2.601710208 | 0.019725449 | 0.321417607 | -3.107864653 |
| EIF5A2 | -0.534179524 | 5.628570039 | -2.601289522 | 0.01974211 | 0.321417607 | -3.108559532 |
| MTHFS | 0.540152605 | 10.07121245 | 2.601109543 | 0.019749243 | 0.321417607 | -3.108856808 |
| NACC2 | 0.480977131 | 8.086771804 | 2.601099387 | 0.019749645 | 0.321417607 | -3.108873583 |
| ZNF823 | -0.580613191 | 4.426250429 | -2.600417477 | 0.019776691 | 0.321616173 | -3.109999846 |
| BNC2 | -1.208723436 | 3.122526383 | -2.600324853 | 0.019780367 | 0.321616173 | -3.11015282 |
| SBF1 | -0.251605215 | 11.04705581 | -2.600037169 | 0.01979179 | 0.321651312 | -3.110627936 |
| lnc-ADCY2-1 | 0.848201022 | 1.494579162 | 2.598405537 | 0.019856694 | 0.322480603 | -3.113322311 |
| COL6A2 | -1.166355415 | 3.762847448 | -2.598287737 | 0.019861388 | 0.322480603 | -3.113516818 |
| lnc-PHF20L1-2 | 0.385794486 | 0.830400304 | 2.597563329 | 0.019890276 | 0.322797122 | -3.114712879 |
| DTX4 | -0.749363202 | 1.623845012 | -2.597332961 | 0.019899471 | 0.322797122 | -3.115093214 |
| TFAP2C | 0.636604401 | 1.479831695 | 2.596430362 | 0.019935538 | 0.323088818 | -3.116583302 |
| BCL7A | -0.934660189 | 6.084935749 | -2.595872552 | 0.019957858 | 0.323088818 | -3.117504102 |
| FNTA | -0.354629087 | 10.1040697 | -2.595575521 | 0.019969753 | 0.323088818 | -3.1179944 |
| lnc-PI3-1 | 0.639358533 | 7.366800252 | 2.595176515 | 0.019985743 | 0.323088818 | -3.118652997 |
| ZNF707 | -0.245820824 | 7.220502982 | -2.594911623 | 0.019996365 | 0.323088818 | -3.119090209 |
| RAPGEF1 | -0.421950939 | 6.211453701 | -2.594433492 | 0.020015552 | 0.323088818 | -3.119879343 |
| NDUFA8 | -0.4008936 | 10.53507494 | -2.594361461 | 0.020018444 | 0.323088818 | -3.119998224 |
| HIST2H2BF | 0.809627519 | 6.40133536 | 2.594341957 | 0.020019227 | 0.323088818 | -3.120030413 |
| DDX56 | -0.258848277 | 10.5393524 | -2.594149528 | 0.020026955 | 0.323088818 | -3.120347992 |
| C12orf75 | -0.482982535 | 7.929985103 | -2.594070864 | 0.020030116 | 0.323088818 | -3.120477815 |
| SF3B5 | -0.345952856 | 10.76443183 | -2.59376498 | 0.020042408 | 0.323088818 | -3.120982618 |
| XLOC_l2_008622 | 0.846021495 | 4.567873598 | 2.593723269 | 0.020044085 | 0.323088818 | -3.121051453 |
| lnc-PACRGL-2 | -0.686590771 | 1.071258539 | -2.593410046 | 0.020056681 | 0.323088818 | -3.121568346 |
| NPTN | 0.42481457 | 7.251075223 | 2.593290678 | 0.020061483 | 0.323088818 | -3.121765328 |
| ITPK1-AS1 | 0.641639819 | 5.606850569 | 2.59324978 | 0.020063129 | 0.323088818 | -3.121832817 |
| LOC286071 | -0.674550207 | 3.90279875 | -2.593170982 | 0.020066299 | 0.323088818 | -3.121962847 |
| SRP72 | -0.374595936 | 10.77379587 | -2.592926342 | 0.020076147 | 0.323097586 | -3.122366538 |
| CROCCP2 | -0.641020401 | 9.318555815 | -2.59262223 | 0.020088395 | 0.32314496 | -3.122868349 |
| FBXO5 | -0.636949268 | 5.700272592 | -2.592141085 | 0.020107788 | 0.323304202 | -3.123662243 |
| SDHAF1 | -0.340390951 | 8.931541483 | -2.591879554 | 0.020118337 | 0.323304202 | -3.124093754 |
| lnc-C7orf11-2 | 0.783487188 | 6.817042532 | 2.591684145 | 0.020126222 | 0.323304202 | -3.124416159 |
| SUMO2 | -0.29098092 | 11.12019782 | -2.591303712 | 0.020141582 | 0.323401353 | -3.12504381 |
| MSRB3 | 0.585024158 | 4.354867648 | 2.590832353 | 0.020160628 | 0.323447632 | -3.125821437 |
| FAR2 | 0.756378046 | 5.702542407 | 2.590771448 | 0.02016309 | 0.323447632 | -3.125921913 |
| CCDC71 | -0.303781872 | 6.187580089 | -2.590231013 | 0.020184952 | 0.323587947 | -3.126813439 |
| POLE2 | -0.726687471 | 2.704215689 | -2.590094638 | 0.020190472 | 0.323587947 | -3.1270384 |
| lnc-CHPF2-1 | -0.838248391 | 1.516494241 | -2.589759612 | 0.020204039 | 0.323656029 | -3.127591036 |
| HCN4 | -0.423102279 | 1.079696429 | -2.589480451 | 0.02021535 | 0.323687931 | -3.128051504 |
| SLC26A8 | 1.653943965 | 3.836703071 | 2.589168057 | 0.020228016 | 0.323735438 | -3.128566771 |
| AARS2 | -0.360177582 | 7.80494802 | -2.58863853 | 0.020249502 | 0.323735438 | -3.129440137 |
| ZNF880 | -0.688719729 | 6.292627892 | -2.588606037 | 0.020250821 | 0.323735438 | -3.129493727 |
| lnc-C15orf2-9 | 0.616270335 | 1.493662935 | 2.588488252 | 0.020255603 | 0.323735438 | -3.129687985 |
| MRPS10 | -0.246814601 | 9.439073132 | -2.587605815 | 0.020291468 | 0.324029713 | -3.131143268 |
| PTTG1 | -0.549061984 | 8.399015726 | -2.587576123 | 0.020292676 | 0.324029713 | -3.131192233 |
| ZBTB1 | -0.788084657 | 7.324971766 | -2.587311858 | 0.020303428 | 0.324052422 | -3.131628017 |
| CIB2 | -0.864991339 | 5.686502161 | -2.586580143 | 0.02033323 | 0.324335621 | -3.132834577 |
| FLJ27255 | 1.399096598 | 5.456857719 | 2.586212816 | 0.020348207 | 0.324335621 | -3.13344024 |
| YAF2 | -0.250688191 | 7.327697581 | -2.586091498 | 0.020353155 | 0.324335621 | -3.133640268 |
| OXNAD1 | -0.573352571 | 6.568015569 | -2.585800827 | 0.020365017 | 0.324335621 | -3.134119513 |
| ZNF324B | -0.174831929 | 6.608095887 | -2.585305442 | 0.020385247 | 0.324335621 | -3.134936241 |
| ZNF571 | -0.902903406 | 4.783373004 | -2.585206566 | 0.020389287 | 0.324335621 | -3.13509925 |
| SEC61G | -0.673926275 | 8.648625902 | -2.584947586 | 0.020399873 | 0.324335621 | -3.135526199 |
| DNASE1L1 | 0.1868463 | 7.977592125 | 2.584759118 | 0.02040758 | 0.324335621 | -3.135836895 |
| lnc-CDS1-2 | 1.022359653 | 2.881199769 | 2.584590333 | 0.020414484 | 0.324335621 | -3.136115138 |
| lnc-SIVA1-1 | 0.634729891 | 3.8958257 | 2.584079323 | 0.020435401 | 0.324335621 | -3.136957503 |
| ZDHHC4 | -0.405188325 | 9.55888977 | -2.583644452 | 0.020453218 | 0.324335621 | -3.137674319 |
| C9orf24 | -0.552608343 | 4.040857896 | -2.583446813 | 0.02046132 | 0.324335621 | -3.138000082 |
| RNF169 | 0.244288534 | 7.159611934 | 2.583262385 | 0.020468884 | 0.324335621 | -3.138304064 |
| FXN | -0.416283723 | 6.864857694 | -2.583159384 | 0.020473109 | 0.324335621 | -3.138473833 |
| POLR1B | -0.423472731 | 6.177298619 | -2.583075201 | 0.020476563 | 0.324335621 | -3.138612582 |
| lnc-ABCA5-1 | 0.739271397 | 1.559796891 | 2.582944262 | 0.020481937 | 0.324335621 | -3.138828391 |
| CHSY1 | 0.465766496 | 10.05836665 | 2.582837725 | 0.02048631 | 0.324335621 | -3.139003981 |
| UGDH | -0.607129351 | 5.212050235 | -2.582765609 | 0.02048927 | 0.324335621 | -3.139122837 |
| TMEM121 | -0.755288777 | 6.348148997 | -2.581628071 | 0.020536025 | 0.324879304 | -3.140997513 |
| CXCR1 | 0.815863534 | 10.7284673 | 2.581448398 | 0.020543419 | 0.324879304 | -3.141293592 |
| BAZ1A | 0.404501867 | 9.120030649 | 2.58124774 | 0.02055168 | 0.324879304 | -3.141624246 |
| HBP1 | 0.270451638 | 7.566337011 | 2.579844663 | 0.020609531 | 0.325345395 | -3.143936073 |
| NEDD8 | -0.224102165 | 12.76001614 | -2.579812295 | 0.020610867 | 0.325345395 | -3.143989401 |
| ANKRD2 | 0.869098384 | 2.003819641 | 2.579450781 | 0.020625799 | 0.325345395 | -3.144584996 |
| LOC283075 | 0.693212542 | 3.631751248 | 2.579379581 | 0.020628741 | 0.325345395 | -3.144702295 |
| MACF1 | -0.433452941 | 10.26148435 | -2.579006715 | 0.020644155 | 0.325345395 | -3.145316561 |
| FAM86B2 | -0.374157529 | 6.306006121 | -2.578862055 | 0.020650138 | 0.325345395 | -3.145554868 |
| LPCAT2 | 0.665212152 | 7.551836406 | 2.578748533 | 0.020654835 | 0.325345395 | -3.145741877 |
| PPP1R7 | -0.192979296 | 8.993169999 | -2.578521903 | 0.020664213 | 0.325345395 | -3.146115206 |
| DPF3 | -0.666200021 | 4.486180051 | -2.578324031 | 0.020672405 | 0.325345395 | -3.146441153 |
| ZNF512B | -0.521514821 | 6.480500082 | -2.578147156 | 0.020679731 | 0.325345395 | -3.146732506 |
| LOC101928976 | 0.967138833 | 6.620738719 | 2.578039015 | 0.020684211 | 0.325345395 | -3.146910636 |
| CRB1 | 0.931879051 | 1.191915825 | 2.577522283 | 0.02070563 | 0.325416755 | -3.147761763 |
| GCNT4 | -0.985375484 | 1.714693258 | -2.57732861 | 0.020713663 | 0.325416755 | -3.148080754 |
| ANLN | -0.526173241 | 0.97638672 | -2.577251627 | 0.020716857 | 0.325416755 | -3.148207549 |
| LIPT2 | -0.498160294 | 5.119363783 | -2.576345513 | 0.020754487 | 0.325659205 | -3.149699863 |
| DYRK2 | -0.509937514 | 7.965787265 | -2.576325208 | 0.020755331 | 0.325659205 | -3.149733302 |
| HIST1H2AD | 0.741768753 | 10.30576337 | 2.576202735 | 0.020760423 | 0.325659205 | -3.149934995 |
| ASMTL-AS1 | 0.733872969 | 5.000567356 | 2.575737981 | 0.020779754 | 0.325718487 | -3.150700339 |
| lnc-PAPSS2-2 | 0.675089585 | 6.20431571 | 2.575660971 | 0.020782959 | 0.325718487 | -3.150827152 |
| LOXL3 | 0.318265369 | 7.377945116 | 2.575341119 | 0.020796275 | 0.325780173 | -3.151353847 |
| GSS | -0.332205705 | 8.034482116 | -2.57475027 | 0.020820895 | 0.325963908 | -3.152326732 |
| STRAP | -0.329945647 | 10.21885473 | -2.574609255 | 0.020826775 | 0.325963908 | -3.152558916 |
| EWSR1 | -0.258124148 | 9.792592894 | -2.574208032 | 0.020843514 | 0.326078944 | -3.153219514 |
| PLAUR | 0.6581077 | 6.915624263 | 2.573465824 | 0.020874513 | 0.326190518 | -3.154441445 |
| PTGES2 | -0.470732295 | 4.281792227 | -2.573419893 | 0.020876433 | 0.326190518 | -3.15451706 |
| BCL11B | -0.624330991 | 10.06173741 | -2.5731413 | 0.020888081 | 0.326190518 | -3.154975689 |
| NDUFB4 | -0.352687958 | 11.64109154 | -2.573138092 | 0.020888215 | 0.326190518 | -3.154980971 |
| LOC101928403 | -0.952058496 | 4.085120823 | -2.572501256 | 0.020914865 | 0.326361966 | -3.156029292 |
| lnc-GGPS1-1 | 0.876464839 | 2.018343781 | 2.572266534 | 0.020924695 | 0.326361966 | -3.156415658 |
| lnc-SLTM-2 | -0.380146459 | 4.956836138 | -2.57151826 | 0.020956064 | 0.326361966 | -3.157647285 |
| lnc-ABCA13-1 | 0.489103017 | 4.896297327 | 2.571512572 | 0.020956303 | 0.326361966 | -3.157656647 |
| lnc-ZDHHC9-1 | 1.19194153 | 2.619243426 | 2.571442905 | 0.020959226 | 0.326361966 | -3.157771309 |
| TMSB15B | -0.781959493 | 4.35186919 | -2.571321371 | 0.020964326 | 0.326361966 | -3.157971337 |
| ADAT3 | -0.3004214 | 8.370764468 | -2.570765601 | 0.020987662 | 0.326361966 | -3.158886015 |
| NOM1 | -0.460437115 | 6.317023587 | -2.570542171 | 0.020997051 | 0.326361966 | -3.159253716 |
| TLE2 | -0.612998374 | 5.653677394 | -2.570425334 | 0.021001962 | 0.326361966 | -3.159445991 |
| TGFA | 0.516465467 | 8.630032939 | 2.570267947 | 0.02100858 | 0.326361966 | -3.159704994 |
| LOC728147 | -0.398417316 | 3.845455992 | -2.570081702 | 0.021016413 | 0.326361966 | -3.160011481 |
| LOC101928785 | -0.695903167 | 1.111589825 | -2.569837961 | 0.021026669 | 0.326361966 | -3.160412573 |
| lnc-BRMS1L-1 | 0.808643472 | 2.644960126 | 2.569817771 | 0.021027519 | 0.326361966 | -3.160445797 |
| LOC101929531 | -0.780559805 | 7.614888964 | -2.569536323 | 0.021039367 | 0.326361966 | -3.160908921 |
| LOC729603 | 0.758713056 | 5.921420414 | 2.569517717 | 0.021040151 | 0.326361966 | -3.160939538 |
| TASP1 | -0.646975599 | 5.540616975 | -2.568489841 | 0.02108348 | 0.326479212 | -3.162630778 |
| LINC00162 | 0.89106234 | 2.193467516 | 2.568434898 | 0.021085798 | 0.326479212 | -3.162721173 |
| IFT27 | -0.191964489 | 5.702332914 | -2.56832086 | 0.021090611 | 0.326479212 | -3.162908796 |
| FUBP3 | -0.509905038 | 7.017624251 | -2.567728702 | 0.02111562 | 0.326479212 | -3.163883 |
| PAX9 | 0.832383999 | 2.792519858 | 2.567688398 | 0.021117323 | 0.326479212 | -3.163949304 |
| lnc-TOMM20-1 | 0.829100978 | 4.81576098 | 2.567633752 | 0.021119633 | 0.326479212 | -3.164039202 |
| CD300LB | 0.485575663 | 5.576579831 | 2.567370482 | 0.021130763 | 0.326479212 | -3.164472301 |
| SPI1 | 0.581078686 | 12.02280191 | 2.567174957 | 0.021139032 | 0.326479212 | -3.164793944 |
| CTSL | 0.433252779 | 7.258808134 | 2.56714799 | 0.021140173 | 0.326479212 | -3.164838304 |
| USP7 | -0.379521434 | 5.304308546 | -2.566803087 | 0.021154769 | 0.326479212 | -3.165405658 |
| lnc-AVEN-1 | -0.652730566 | 3.349311658 | -2.566777808 | 0.021155839 | 0.326479212 | -3.165447239 |
| LINC00174 | 0.956444748 | 8.210142591 | 2.566629916 | 0.0211621 | 0.326479212 | -3.165690509 |
| MAP3K11 | 0.308559474 | 11.71762218 | 2.566156859 | 0.021182142 | 0.326479212 | -3.166468615 |
| C16orf58 | -0.389174002 | 7.136761071 | -2.565946561 | 0.021191057 | 0.326479212 | -3.166814507 |
| lnc-MTA3-3 | 0.498325295 | 5.897148299 | 2.565727888 | 0.021200331 | 0.326479212 | -3.167174166 |
| CHM | -0.274827052 | 6.140633824 | -2.565710589 | 0.021201064 | 0.326479212 | -3.167202618 |
| SPRR3 | 0.842332342 | 1.424085256 | 2.565386148 | 0.021214832 | 0.326479212 | -3.167736219 |
| GPN1 | -0.418938744 | 9.196207734 | -2.565336987 | 0.021216919 | 0.326479212 | -3.167817071 |
| lnc-UCHL5-1 | 1.031423294 | 2.099043563 | 2.564738439 | 0.021242342 | 0.326725659 | -3.168801425 |
| PPAP2C | 1.08551706 | 3.477002297 | 2.56433725 | 0.021259399 | 0.32684326 | -3.169461169 |
| FAM49A | 0.418817544 | 9.113654486 | 2.563597648 | 0.021290878 | 0.327000336 | -3.170677337 |
| CHRM3-AS2 | -0.948753754 | 4.304234317 | -2.56339099 | 0.021299682 | 0.327000336 | -3.171017134 |
| ATE1 | -0.383494703 | 4.115417994 | -2.563275337 | 0.02130461 | 0.327000336 | -3.171207294 |
| SNORA75 | -0.786811809 | 3.221597974 | -2.563201747 | 0.021307747 | 0.327000336 | -3.171328292 |
| NR1D1 | -0.976620841 | 4.160886982 | -2.562991898 | 0.021316693 | 0.327000336 | -3.171673321 |
| KIAA1191 | -0.346540444 | 9.536843009 | -2.562496042 | 0.021337848 | 0.327077168 | -3.17248856 |
| SIKE1 | -0.228335708 | 8.459562541 | -2.562433027 | 0.021340537 | 0.327077168 | -3.172592159 |
| FAM157C | 1.132732869 | 6.746879517 | 2.561956275 | 0.021360898 | 0.327181629 | -3.173375936 |
| RDH5 | 0.425629551 | 5.781303511 | 2.561776262 | 0.021368591 | 0.327181629 | -3.173671864 |
| TYMS | -0.983977722 | 6.979947706 | -2.561401542 | 0.021384612 | 0.327181629 | -3.174287856 |
| IL17C | -0.647738565 | 2.722493615 | -2.561115595 | 0.021396846 | 0.327181629 | -3.174757895 |
| LOC102724420 | -0.839954728 | 2.414892995 | -2.560999514 | 0.021401814 | 0.327181629 | -3.174948704 |
| ZNF792 | -0.457724107 | 5.260659243 | -2.560767654 | 0.021411741 | 0.327181629 | -3.175329819 |
| DDX54 | -0.388234725 | 10.15544745 | -2.560423424 | 0.021426487 | 0.327181629 | -3.175895618 |
| MCF2L-AS1 | -0.484658387 | 5.116865808 | -2.560312625 | 0.021431236 | 0.327181629 | -3.17607773 |
| DYSF | 1.125588184 | 7.627563996 | 2.560291536 | 0.02143214 | 0.327181629 | -3.176112391 |
| NEFH | -0.590216387 | 6.426474496 | -2.559642408 | 0.02145998 | 0.327462703 | -3.177179258 |
| LOC101927932 | -0.657298187 | 3.011300202 | -2.559190198 | 0.021479396 | 0.327615022 | -3.177922431 |
| EXOC5 | -0.246628111 | 5.02648034 | -2.558964679 | 0.021489084 | 0.327618919 | -3.178293039 |
| TXNDC5 | -1.09064919 | 9.259414677 | -2.558242987 | 0.021520118 | 0.327632839 | -3.179478965 |
| BTBD11 | -0.624712228 | 5.75828624 | -2.558025461 | 0.02152948 | 0.327632839 | -3.179836395 |
| LOC154449 | 0.423188468 | 5.280250393 | 2.557936268 | 0.02153332 | 0.327632839 | -3.179982952 |
| OR1L3 | 1.028112065 | 1.46592036 | 2.55769486 | 0.021543717 | 0.327632839 | -3.180379608 |
| INSL3 | 0.838749302 | 8.157020847 | 2.557642127 | 0.021545988 | 0.327632839 | -3.180466252 |
| TMEM261 | -0.742272058 | 4.145022512 | -2.557608125 | 0.021547453 | 0.327632839 | -3.18052212 |
| METAP1D | -0.664978392 | 4.101031641 | -2.557376432 | 0.021557437 | 0.327632839 | -3.180902798 |
| NOP14 | -0.380956047 | 9.119517507 | -2.5571514 | 0.021567139 | 0.327632839 | -3.181272521 |
| lnc-RAB2A-1 | 0.493480296 | 6.483756037 | 2.556971425 | 0.021574901 | 0.327632839 | -3.18156821 |
| STIP1 | -0.423428527 | 4.544193879 | -2.556653499 | 0.021588619 | 0.327697872 | -3.182090528 |
| ZNF195 | -0.324569467 | 7.485142927 | -2.556325739 | 0.02160277 | 0.327735927 | -3.182628979 |
| SHKBP1 | 0.559319335 | 7.70136131 | 2.556158377 | 0.021609999 | 0.327735927 | -3.182903918 |
| CLEC7A | 0.59469483 | 5.834778791 | 2.555051493 | 0.02165787 | 0.327780336 | -3.184722132 |
| ZNF786 | -0.433128414 | 7.090726988 | -2.555003944 | 0.021659929 | 0.327780336 | -3.184800232 |
| lnc-MPZ-1 | 0.482407404 | 0.867312724 | 2.55469768 | 0.021673193 | 0.327780336 | -3.185303267 |
| USP20 | -0.230493325 | 8.248864334 | -2.554694396 | 0.021673335 | 0.327780336 | -3.185308661 |
| CCNF | -0.553113549 | 1.291286409 | -2.554616691 | 0.021676702 | 0.327780336 | -3.185436287 |
| RGS20 | -0.418550638 | 1.12116506 | -2.554243996 | 0.021692857 | 0.327780336 | -3.186048402 |
| CD320 | -0.513109184 | 7.671954696 | -2.554094722 | 0.02169933 | 0.327780336 | -3.186293562 |
| RNF26 | -0.275575022 | 8.025478841 | -2.553898909 | 0.021707825 | 0.327780336 | -3.186615146 |
| lnc-ZNF2-1 | -0.476663022 | 3.464266322 | -2.553294261 | 0.021734076 | 0.327780336 | -3.187608116 |
| RICTOR | 0.358569746 | 9.561111445 | 2.553221744 | 0.021737226 | 0.327780336 | -3.187727201 |
| IL12RB2 | -0.746656921 | 2.973254468 | -2.553215579 | 0.021737494 | 0.327780336 | -3.187737325 |
| DSEL | -0.719768779 | 4.529793913 | -2.553092382 | 0.021742847 | 0.327780336 | -3.187939631 |
| RNASET2 | 0.339702204 | 12.35417708 | 2.552986808 | 0.021747435 | 0.327780336 | -3.188112995 |
| lnc-PCDH12-1 | 0.631262731 | 1.736727315 | 2.552638075 | 0.021762598 | 0.327780336 | -3.188685637 |
| lnc-YIF1A-1 | -0.718230172 | 2.539760271 | -2.552521522 | 0.021767668 | 0.327780336 | -3.188877021 |
| CTBS | 0.55250582 | 10.44740422 | 2.552262309 | 0.021778947 | 0.327780336 | -3.189302643 |
| ERAP2 | -1.446976344 | 9.363903415 | -2.55205237 | 0.021788087 | 0.327780336 | -3.189647348 |
| MZB1 | -1.229262748 | 8.745737154 | -2.551940292 | 0.021792968 | 0.327780336 | -3.189831369 |
| CHST2 | -0.547669408 | 7.680294347 | -2.551536137 | 0.021810576 | 0.327780336 | -3.190494927 |
| WDR70 | -0.331353133 | 7.694698507 | -2.551371827 | 0.021817739 | 0.327780336 | -3.190764689 |
| INAFM2 | 0.343365252 | 8.641713126 | 2.551338918 | 0.021819174 | 0.327780336 | -3.190818719 |
| C3 | -0.567889394 | 3.775786372 | -2.551160234 | 0.021826966 | 0.327780336 | -3.191112072 |
| LOC100130587 | 0.724775502 | 3.322427371 | 2.550881207 | 0.02183914 | 0.327780336 | -3.191570148 |
| ERBB4 | -0.566423365 | 1.197894049 | -2.5506618 | 0.021848717 | 0.327780336 | -3.191930336 |
| RAPGEF3 | 0.279313026 | 6.62508177 | 2.550658137 | 0.021848876 | 0.327780336 | -3.191936349 |
| HNRNPD | -0.332859501 | 11.51452822 | -2.550092299 | 0.021873594 | 0.327953667 | -3.192865207 |
| NHP2 | -0.494127046 | 11.28277724 | -2.549961385 | 0.021879316 | 0.327953667 | -3.193080101 |
| lnc-MED10-5 | 0.773386534 | 2.161701519 | 2.549665648 | 0.021892248 | 0.328005948 | -3.193565537 |
| KIF15 | -0.914445374 | 3.16169568 | -2.549079303 | 0.02191791 | 0.328147145 | -3.194527935 |
| CYB5D2 | -0.265912298 | 4.372103744 | -2.549018586 | 0.021920569 | 0.328147145 | -3.19462759 |
| SCRN2 | -0.410816921 | 5.299052542 | -2.54826801 | 0.021953465 | 0.328217815 | -3.195859436 |
| lnc-ANKH-1 | -0.431865034 | 3.610850955 | -2.548220269 | 0.021955559 | 0.328217815 | -3.195937785 |
| LOC101928830 | 0.985107883 | 7.978678646 | 2.548079143 | 0.02196175 | 0.328217815 | -3.196169386 |
| AKR1B1 | -0.427118371 | 8.158995445 | -2.548032211 | 0.021963809 | 0.328217815 | -3.196246405 |
| lnc-MBOAT4-2 | -0.374747633 | 0.872705153 | -2.547833198 | 0.021972543 | 0.328217815 | -3.196572995 |
| TRPV2 | -0.33130095 | 11.13485585 | -2.546840162 | 0.022016174 | 0.328723819 | -3.198202497 |
| CSTF2 | -0.399849364 | 6.526877617 | -2.546631597 | 0.022025348 | 0.328723819 | -3.198544712 |
| LOC100507516 | 1.265607237 | 2.449666387 | 2.546053116 | 0.022050812 | 0.328962506 | -3.199493838 |
| STK39 | -0.474986612 | 6.727017056 | -2.545320058 | 0.022083122 | 0.32930306 | -3.200696482 |
| lnc-FMR1NB-1 | 0.500334087 | 5.023644546 | 2.544967575 | 0.022098674 | 0.329393536 | -3.20127472 |
| RCOR1 | 0.303371175 | 9.418404864 | 2.544149587 | 0.022134804 | 0.329778182 | -3.202616505 |
| DTHD1 | -0.812378982 | 2.167811311 | -2.543953581 | 0.02214347 | 0.329778182 | -3.202938003 |
| SLC8A1 | 0.909480425 | 8.321130242 | 2.543481828 | 0.022164341 | 0.329840904 | -3.203711759 |
| UBXN1 | -0.226021786 | 12.53919776 | -2.543429065 | 0.022166676 | 0.329840904 | -3.203798296 |
| TMEM194B | -0.425532351 | 4.796663385 | -2.542822839 | 0.022193527 | 0.330055367 | -3.204792535 |
| CRYL1 | -0.364903331 | 8.554501459 | -2.542674619 | 0.022200096 | 0.330055367 | -3.205035612 |
| LRBA | -0.43983014 | 7.360521724 | -2.542436368 | 0.02221066 | 0.330071125 | -3.205426326 |
| LOC100134868 | 0.378759849 | 8.899636572 | 2.54212658 | 0.022224403 | 0.330100208 | -3.205934338 |
| HDAC2 | -0.317143959 | 9.142369421 | -2.54196382 | 0.022231626 | 0.330100208 | -3.206201237 |
| BTBD6 | -0.365422346 | 10.41429864 | -2.541729588 | 0.022242026 | 0.33011349 | -3.206585326 |
| KLKB1 | 1.021353443 | 5.001664279 | 2.540858434 | 0.022280745 | 0.330405705 | -3.208013725 |
| lnc-CHP2.1-1 | 0.795618967 | 6.162727204 | 2.540715894 | 0.022287087 | 0.330405705 | -3.208247427 |
| PVRIG | -0.36208566 | 11.29102386 | -2.54037656 | 0.02230219 | 0.330405705 | -3.208803767 |
| GPR149 | -0.676646305 | 1.598478145 | -2.540152999 | 0.022312146 | 0.330405705 | -3.209170284 |
| ANAPC2 | -0.413345036 | 3.492694066 | -2.539336852 | 0.022348528 | 0.330405705 | -3.210508224 |
| LHFPL5 | 0.933242318 | 5.806744809 | 2.539142446 | 0.022357202 | 0.330405705 | -3.2108269 |
| lnc-SRRM4-1 | -0.522680122 | 1.240118299 | -2.539096708 | 0.022359243 | 0.330405705 | -3.210901873 |
| PTCH1 | -0.684181147 | 5.156927615 | -2.539054796 | 0.022361114 | 0.330405705 | -3.210970576 |
| FZD5 | 0.523140148 | 2.257227352 | 2.538950334 | 0.022365777 | 0.330405705 | -3.211141808 |
| PTK2 | -0.563605868 | 3.357082435 | -2.538913441 | 0.022367424 | 0.330405705 | -3.211202281 |
| ZW10 | -0.414586085 | 5.465111832 | -2.538490383 | 0.022386321 | 0.330405705 | -3.211895724 |
| ARHGAP24 | 0.884286604 | 5.917582496 | 2.538280465 | 0.022395702 | 0.330405705 | -3.212239789 |
| VPS37C | 0.40876187 | 9.149789329 | 2.53811883 | 0.022402929 | 0.330405705 | -3.212504711 |
| RRP36 | -0.207226093 | 10.09898122 | -2.538008695 | 0.022407854 | 0.330405705 | -3.21268522 |
| ARG1 | 1.685685938 | 6.733187198 | 2.537940985 | 0.022410883 | 0.330405705 | -3.212796194 |
| TBC1D32 | -0.683185805 | 2.163279772 | -2.537872829 | 0.022413931 | 0.330405705 | -3.212907898 |
| GEMIN5 | -0.469100199 | 6.553153619 | -2.537334465 | 0.022438028 | 0.330620588 | -3.213790215 |
| lnc-OLFML3-1 | 0.524255531 | 1.177555873 | 2.536594571 | 0.022471186 | 0.330754394 | -3.215002717 |
| ATG14 | -0.594547789 | 2.369188886 | -2.536419144 | 0.022479054 | 0.330754394 | -3.215290182 |
| CCDC102B | -0.478757746 | 4.652074605 | -2.536354948 | 0.022481934 | 0.330754394 | -3.215395374 |
| ZNF534 | -0.436483604 | 1.02247159 | -2.536208298 | 0.022488515 | 0.330754394 | -3.215635675 |
| LOC101928389 | 0.754226933 | 2.102405531 | 2.535945095 | 0.02250033 | 0.330754394 | -3.21606695 |
| POT1 | -0.845021326 | 6.668130744 | -2.535857783 | 0.022504251 | 0.330754394 | -3.216210013 |
| lnc-PPP4R1-10 | 0.315458375 | 1.040943985 | 2.535425113 | 0.022523689 | 0.330821377 | -3.21691893 |
| SDCBP | 0.52447351 | 10.80642914 | 2.535332343 | 0.022527859 | 0.330821377 | -3.217070925 |
| LOC101927943 | -0.782987971 | 2.058828214 | -2.534577574 | 0.022561813 | 0.331066695 | -3.218307481 |
| IBA57 | -0.353582379 | 6.203439379 | -2.534452837 | 0.022567429 | 0.331066695 | -3.218511828 |
| LOC100129455 | 1.261994392 | 2.163426381 | 2.534325533 | 0.022573163 | 0.331066695 | -3.21872038 |
| NCL | -0.508747509 | 11.06229316 | -2.533897285 | 0.022592459 | 0.331209829 | -3.219421911 |
| JMJD4 | -0.30716569 | 8.980791644 | -2.533157837 | 0.022625814 | 0.331546169 | -3.220633143 |
| BFAR | -0.283645433 | 8.445301457 | -2.532965583 | 0.022634494 | 0.331546169 | -3.22094804 |
| ZNF142 | -0.206755295 | 7.033761052 | -2.532607982 | 0.022650647 | 0.331642908 | -3.22153374 |
| RFC1 | -0.414831297 | 6.935700661 | -2.53205947 | 0.022675446 | 0.331684456 | -3.222432075 |
| lnc-CBWD5-1 | 0.898995359 | 3.28739421 | 2.531896972 | 0.022682798 | 0.331684456 | -3.222698197 |
| GDPD3 | 0.572033296 | 3.88154144 | 2.531752382 | 0.022689341 | 0.331684456 | -3.222934986 |
| lnc-KIAA1755-6 | -0.719587556 | 2.456647153 | -2.531580937 | 0.022697102 | 0.331684456 | -3.223215748 |
| ZNF691 | -0.269531411 | 5.982189169 | -2.53131552 | 0.022709122 | 0.331684456 | -3.22365039 |
| IL1B | 0.70753161 | 10.66024341 | 2.531061795 | 0.022720618 | 0.331684456 | -3.22406587 |
| ARRDC1-AS1 | -0.332874862 | 4.278021907 | -2.530875499 | 0.022729063 | 0.331684456 | -3.224370924 |
| CTAG2 | -0.865978998 | 2.339825713 | -2.530603286 | 0.022741407 | 0.331684456 | -3.224816653 |
| ZNF154 | -0.721011933 | 3.862612261 | -2.530069055 | 0.022765652 | 0.331684456 | -3.225691371 |
| CARD16 | 0.510109603 | 9.049629651 | 2.529928127 | 0.022772052 | 0.331684456 | -3.225922108 |
| COPS7B | -0.56206669 | 3.99909562 | -2.529548744 | 0.022789289 | 0.331684456 | -3.226543238 |
| lnc-HTR1A-1 | 0.48352488 | 1.525593747 | 2.529188271 | 0.022805679 | 0.331684456 | -3.227133381 |
| MACROD2 | 0.726737762 | 7.824964904 | 2.528966063 | 0.022815787 | 0.331684456 | -3.227497151 |
| FAM120C | -0.374754784 | 7.288488507 | -2.528926209 | 0.022817601 | 0.331684456 | -3.227562393 |
| lnc-IL6-3 | 0.785340473 | 3.400582318 | 2.528770242 | 0.022824699 | 0.331684456 | -3.227817716 |
| FLJ27354 | -0.423072747 | 5.012773557 | -2.52861763 | 0.022831647 | 0.331684456 | -3.228067538 |
| LOC101930532 | 0.607718925 | 4.993094525 | 2.528394694 | 0.0228418 | 0.331684456 | -3.228432474 |
| SAA3P | 0.537312609 | 3.698834789 | 2.52823777 | 0.022848949 | 0.331684456 | -3.228689344 |
| HLA-B | 0.372134989 | 13.90223737 | 2.527968333 | 0.022861229 | 0.331684456 | -3.229130374 |
| PDIA5 | -0.541273204 | 6.678163343 | -2.52782076 | 0.022867957 | 0.331684456 | -3.229371925 |
| ZNF354C | -0.587026962 | 1.309411509 | -2.527753491 | 0.022871025 | 0.331684456 | -3.22948203 |
| FAM86FP | -0.307209967 | 4.646624025 | -2.527637935 | 0.022876296 | 0.331684456 | -3.22967117 |
| BBX | -0.21442609 | 10.28227512 | -2.527603279 | 0.022877877 | 0.331684456 | -3.229727893 |
| CPPED1 | 0.51949095 | 10.09543524 | 2.527495901 | 0.022882776 | 0.331684456 | -3.229903644 |
| RARA | 0.650896265 | 10.27204539 | 2.527288434 | 0.022892244 | 0.331684456 | -3.230243207 |
| ZHX3 | -0.395858898 | 5.557763231 | -2.527016223 | 0.022904673 | 0.331726144 | -3.230688723 |
| lnc-TUBA1A-1 | 0.571883158 | 4.428528768 | 2.526440091 | 0.022931 | 0.331820273 | -3.231631601 |
| CREB3L2 | -0.28428977 | 9.287451997 | -2.526392302 | 0.022933185 | 0.331820273 | -3.231709809 |
| LOC100507053 | -0.718617757 | 2.480481316 | -2.52624688 | 0.022939835 | 0.331820273 | -3.231947788 |
| PDIA3 | -0.395402533 | 9.686010635 | -2.525944742 | 0.022953658 | 0.331832319 | -3.232442216 |
| YTHDF2 | -0.319237788 | 10.02709328 | -2.525651405 | 0.022967086 | 0.331832319 | -3.232922224 |
| lnc-PRR7-2 | 0.484664751 | 1.436290224 | 2.525602363 | 0.022969332 | 0.331832319 | -3.233002473 |
| SLC25A4 | -0.756603576 | 5.311394975 | -2.525281135 | 0.022984047 | 0.331906834 | -3.233528095 |
| DPYD | 0.319246779 | 8.416181872 | 2.525035813 | 0.02299529 | 0.331931184 | -3.233929497 |
| RAD1 | -0.466955065 | 5.289795619 | -2.524544424 | 0.023017827 | 0.332070818 | -3.234733483 |
| AKR7A2 | -0.313065254 | 9.175822559 | -2.524408034 | 0.023024087 | 0.332070818 | -3.234956629 |
| RRM1 | -0.502270998 | 8.082839101 | -2.523441398 | 0.023068494 | 0.332489541 | -3.236538011 |
| ITPK1 | 0.655817722 | 4.719230912 | 2.523068406 | 0.023085651 | 0.332489541 | -3.237148157 |
| lnc-SLC22A12-1 | 0.977049576 | 3.126371229 | 2.522885 | 0.023094092 | 0.332489541 | -3.237448166 |
| TMED10 | -0.442124937 | 11.0934201 | -2.522840046 | 0.023096161 | 0.332489541 | -3.237521699 |
| ATP5D | -0.245527564 | 9.416376753 | -2.522578253 | 0.023108216 | 0.332489541 | -3.237949913 |
| FLJ16171 | -0.735680959 | 2.104204329 | -2.521391031 | 0.023162958 | 0.332489541 | -3.239891668 |
| CALM1 | -0.350933378 | 7.651704021 | -2.521348783 | 0.023164908 | 0.332489541 | -3.239960761 |
| EPM2A | -0.594747585 | 1.439563668 | -2.521033772 | 0.023179455 | 0.332489541 | -3.240475921 |
| TREML2 | 0.759198222 | 7.030323963 | 2.520720973 | 0.023193909 | 0.332489541 | -3.240987442 |
| TMEM187 | -0.276666116 | 6.725748155 | -2.520698813 | 0.023194933 | 0.332489541 | -3.24102368 |
| lnc-MRPS25-1 | 0.532055747 | 4.771111853 | 2.520262916 | 0.02321509 | 0.332489541 | -3.241736467 |
| NOB1 | -0.407520738 | 7.021687782 | -2.520055187 | 0.023224701 | 0.332489541 | -3.242076135 |
| ANKRD13A | 0.221024496 | 10.19384355 | 2.519796216 | 0.023236689 | 0.332489541 | -3.242499579 |
| FAM3D | 0.970933775 | 2.752930065 | 2.519711114 | 0.02324063 | 0.332489541 | -3.242638724 |
| NOL8 | -0.339193581 | 9.491554726 | -2.519553988 | 0.023247907 | 0.332489541 | -3.242895631 |
| GRASPOS | 0.455441367 | 4.110980597 | 2.519402555 | 0.023254923 | 0.332489541 | -3.243143224 |
| lnc-RALGPS1-1 | 0.556445577 | 1.689963856 | 2.519151332 | 0.023266567 | 0.332489541 | -3.243553964 |
| SOS2 | 0.498655795 | 8.888144 | 2.519056954 | 0.023270942 | 0.332489541 | -3.243708264 |
| BIRC5 | -1.017863558 | 6.484134283 | -2.519014995 | 0.023272888 | 0.332489541 | -3.243776864 |
| DCAF16 | -0.417877968 | 8.568242674 | -2.51847906 | 0.023297752 | 0.332489541 | -3.244653032 |
| SIGLEC9 | 0.72573878 | 7.025096119 | 2.517904516 | 0.023324436 | 0.332489541 | -3.245592253 |
| P3H1 | -0.221747816 | 7.374514156 | -2.517809233 | 0.023328864 | 0.332489541 | -3.245748007 |
| ILF3-AS1 | -0.372662011 | 8.37214976 | -2.517517551 | 0.023342424 | 0.332489541 | -3.246224791 |
| CSNK1D | 0.436224916 | 7.817576204 | 2.517415578 | 0.023347166 | 0.332489541 | -3.246391473 |
| INA | 0.570042047 | 4.371873927 | 2.517387443 | 0.023348475 | 0.332489541 | -3.24643746 |
| OCIAD2 | -0.607528551 | 10.04648669 | -2.517079557 | 0.0233628 | 0.332489541 | -3.246940704 |
| SMPDL3B | 0.821625916 | 1.796679369 | 2.516844224 | 0.023373755 | 0.332489541 | -3.247325346 |
| TRIM44 | -0.259277235 | 8.458023125 | -2.516488189 | 0.023390339 | 0.332489541 | -3.247907245 |
| KLRD1 | -0.727567537 | 8.191677639 | -2.516251935 | 0.023401349 | 0.332489541 | -3.248293361 |
| HIST1H2BD | 0.512486501 | 6.956417115 | 2.515756168 | 0.02342447 | 0.332489541 | -3.249103566 |
| PHKA1 | -0.803849293 | 1.77285851 | -2.515677426 | 0.023428144 | 0.332489541 | -3.249232245 |
| ESPL1 | -0.601351209 | 3.985187525 | -2.515533478 | 0.023434862 | 0.332489541 | -3.249467478 |
| LOC101929153 | -0.385017278 | 0.952321975 | -2.515171041 | 0.023451786 | 0.332489541 | -3.250059737 |
| FAM63A | 0.635724064 | 10.57113807 | 2.515160627 | 0.023452273 | 0.332489541 | -3.250076756 |
| lnc-EIF2B5-2 | 0.400554369 | 3.062901632 | 2.514982146 | 0.023460611 | 0.332489541 | -3.2503684 |
| HID1-AS1 | 0.615713733 | 3.603196186 | 2.514940217 | 0.02346257 | 0.332489541 | -3.250436913 |
| lnc-PRDM14-1 | 0.539360793 | 3.623877286 | 2.514606382 | 0.023478176 | 0.332489541 | -3.250982392 |
| TIMM50 | -0.275375431 | 7.893658491 | -2.514580272 | 0.023479396 | 0.332489541 | -3.251025054 |
| CAPS | -0.336206215 | 6.330097878 | -2.514571895 | 0.023479788 | 0.332489541 | -3.251038742 |
| ARAP1 | 0.440308961 | 8.768782583 | 2.514489358 | 0.023483648 | 0.332489541 | -3.251173601 |
| PNKD | -0.359440405 | 9.580847392 | -2.514017513 | 0.023505727 | 0.332489541 | -3.25194453 |
| CREB3L4 | -0.369023057 | 6.671298837 | -2.513525634 | 0.023528764 | 0.332489541 | -3.252748142 |
| lnc-RP11-305N23.1.1-1 | 0.340744927 | 5.239346333 | 2.513198459 | 0.023544098 | 0.332489541 | -3.253282637 |
| CD38 | -1.015997943 | 6.993356915 | -2.512856976 | 0.023560114 | 0.332489541 | -3.253840481 |
| XLOC_l2_014123 | -0.388384044 | 3.966735712 | -2.512855086 | 0.023560203 | 0.332489541 | -3.253843569 |
| DYRK3 | -0.715567558 | 2.330551536 | -2.512854569 | 0.023560227 | 0.332489541 | -3.253844413 |
| WDR78 | -0.724132385 | 1.587396431 | -2.512812754 | 0.023562189 | 0.332489541 | -3.253912721 |
| GNB2 | 0.453424433 | 8.611390432 | 2.512546564 | 0.023574682 | 0.332489541 | -3.254347547 |
| SERINC2 | 0.766628893 | 1.789805539 | 2.511772601 | 0.023611041 | 0.332489541 | -3.255611741 |
| RNF19B | 0.619014273 | 6.84324381 | 2.511750181 | 0.023612095 | 0.332489541 | -3.255648361 |
| MEFV | 0.75929401 | 7.900750983 | 2.511468885 | 0.023625323 | 0.332489541 | -3.256107796 |
| LOC102724791 | 0.57847143 | 4.031226858 | 2.511239835 | 0.0236361 | 0.332489541 | -3.256481886 |
| lnc-LGALSL-2 | 0.566985683 | 0.872112596 | 2.510897313 | 0.023652224 | 0.332489541 | -3.257041281 |
| lnc-RTL1-1 | 0.435193778 | 0.879903411 | 2.510653595 | 0.023663704 | 0.332489541 | -3.257439298 |
| lnc-FAS-2 | 0.91886678 | 2.083005644 | 2.51059944 | 0.023666255 | 0.332489541 | -3.257527736 |
| lnc-TM2D2-2 | 0.492351304 | 4.223370114 | 2.510565538 | 0.023667853 | 0.332489541 | -3.2575831 |
| LCLAT1 | -0.475208831 | 7.428486546 | -2.510550056 | 0.023668582 | 0.332489541 | -3.257608383 |
| SAA2 | -0.809634954 | 1.448579831 | -2.510385001 | 0.023676361 | 0.332489541 | -3.257877923 |
| KLHL11 | -0.319551292 | 4.64838603 | -2.510325925 | 0.023679146 | 0.332489541 | -3.257974395 |
| SEC61A1 | -0.384233143 | 7.405845955 | -2.510309563 | 0.023679917 | 0.332489541 | -3.258001114 |
| ISLR2 | 1.201473986 | 3.945993884 | 2.510285129 | 0.023681069 | 0.332489541 | -3.258041014 |
| CTNNA2 | 0.533492396 | 1.12953658 | 2.509902218 | 0.023699128 | 0.332489541 | -3.25866629 |
| LDOC1L | -0.447021484 | 6.506087552 | -2.50972749 | 0.023707373 | 0.332489541 | -3.258951601 |
| LOC101926894 | 0.910084342 | 7.970364859 | 2.50951058 | 0.023717612 | 0.332489541 | -3.259305782 |
| GTF3A | -0.471476095 | 11.28894212 | -2.509468065 | 0.02371962 | 0.332489541 | -3.2593752 |
| PIK3CD-AS1 | 0.475779062 | 5.473085403 | 2.509450873 | 0.023720432 | 0.332489541 | -3.259403273 |
| XLOC_l2_009639 | -0.62877868 | 4.992287043 | -2.509431073 | 0.023721367 | 0.332489541 | -3.259435602 |
| HS2ST1 | -0.478454956 | 5.550113535 | -2.509270858 | 0.023728933 | 0.332489541 | -3.259697199 |
| lnc-DROSHA-4 | 0.588819615 | 1.086505884 | 2.509263023 | 0.023729304 | 0.332489541 | -3.259709991 |
| LOC399715 | 1.042855009 | 6.945127451 | 2.509147554 | 0.023734758 | 0.332489541 | -3.259898524 |
| GNE | -0.352747581 | 8.43538582 | -2.508988977 | 0.023742252 | 0.332489541 | -3.260157437 |
| WWP1 | -0.37780862 | 8.128847729 | -2.508985533 | 0.023742415 | 0.332489541 | -3.26016306 |
| lnc-TPX2-1 | -0.735052439 | 3.208505761 | -2.507921694 | 0.023792744 | 0.332543742 | -3.261899871 |
| HIST3H3 | 0.300878724 | 13.54467075 | 2.50763744 | 0.023806209 | 0.332543742 | -3.2623639 |
| LINC00693 | 0.536641006 | 1.352709501 | 2.50760553 | 0.023807721 | 0.332543742 | -3.26241599 |
| RPL23AP7 | -0.445828139 | 13.05516415 | -2.507531541 | 0.023811227 | 0.332543742 | -3.262536768 |
| ARHGEF18 | -0.391600309 | 11.47196516 | -2.507224028 | 0.023825805 | 0.332543742 | -3.263038738 |
| MAGT1 | -0.281114402 | 8.931494551 | -2.507216864 | 0.023826145 | 0.332543742 | -3.263050432 |
| lnc-AC090186.1-1 | 0.516670303 | 0.877141923 | 2.507084401 | 0.023832427 | 0.332543742 | -3.263266651 |
| C11orf80 | -0.964405582 | 2.535234558 | -2.506740133 | 0.023848762 | 0.332543742 | -3.263828582 |
| TMEM43 | 0.401460993 | 7.995023505 | 2.506605558 | 0.02385515 | 0.332543742 | -3.264048234 |
| LOC102725199 | 0.604352157 | 6.93593975 | 2.506488877 | 0.02386069 | 0.332543742 | -3.264238678 |
| DROSHA | -0.407889561 | 9.197641588 | -2.506482 | 0.023861017 | 0.332543742 | -3.264249902 |
| OLFML1 | 0.816787754 | 1.931191663 | 2.506440295 | 0.023862997 | 0.332543742 | -3.264317971 |
| IRAK3 | 0.910322716 | 6.856399956 | 2.50627683 | 0.023870762 | 0.332543742 | -3.264584766 |
| TRIB3 | -0.378260156 | 8.986993286 | -2.505783283 | 0.023894218 | 0.332638834 | -3.265390262 |
| IKZF2 | -0.704575177 | 8.287179336 | -2.505718104 | 0.023897318 | 0.332638834 | -3.265496634 |
| FAM169A | -0.516932399 | 7.215923974 | -2.505528818 | 0.023906321 | 0.332638834 | -3.265805542 |
| CDYL | -0.391019057 | 5.977458163 | -2.505206572 | 0.023921656 | 0.332718903 | -3.266331417 |
| ZC4H2 | -0.347356326 | 6.227676575 | -2.504219482 | 0.023968686 | 0.333230579 | -3.267942112 |
| EIF2AK3 | -0.797776657 | 4.790190793 | -2.503869782 | 0.023985369 | 0.333230579 | -3.268512688 |
| TMEM8B | -0.497499102 | 6.645384071 | -2.50363223 | 0.023996708 | 0.333230579 | -3.268900266 |
| C17orf100 | -0.729418822 | 3.148043506 | -2.503055195 | 0.024024273 | 0.333230579 | -3.269841675 |
| MCEMP1 | 1.197609405 | 8.489069617 | 2.502881461 | 0.024032578 | 0.333230579 | -3.2701251 |
| NABP1 | 0.490941471 | 9.889442003 | 2.502793998 | 0.02403676 | 0.333230579 | -3.270267782 |
| LINC01550 | -0.599631435 | 6.859651027 | -2.502530301 | 0.024049373 | 0.333230579 | -3.270697954 |
| lnc-SF3B5-1 | 0.264046687 | 1.008635895 | 2.502388874 | 0.024056141 | 0.333230579 | -3.270928657 |
| KRTAP10-5 | 0.409024728 | 3.728252065 | 2.502353912 | 0.024057814 | 0.333230579 | -3.270985689 |
| lnc-TBC1D29-1 | 0.441076631 | 5.156576342 | 2.502322516 | 0.024059316 | 0.333230579 | -3.271036903 |
| MAPRE3 | 0.673593102 | 1.468422082 | 2.502224928 | 0.024063988 | 0.333230579 | -3.271196091 |
| CCND2 | -0.465059298 | 8.416389188 | -2.501786388 | 0.02408499 | 0.333294021 | -3.27191142 |
| lnc-TSFM-1 | 0.861655959 | 1.180177167 | 2.501538395 | 0.024096874 | 0.333294021 | -3.272315917 |
| DEPDC7 | -0.932108832 | 2.340382811 | -2.501528274 | 0.024097359 | 0.333294021 | -3.272332425 |
| lnc-SYCE1L-2 | -0.358510769 | 0.805337334 | -2.501172603 | 0.024114414 | 0.333298642 | -3.272912531 |
| TARSL2 | -0.563927298 | 4.292907152 | -2.500990148 | 0.024123167 | 0.333298642 | -3.273210106 |
| MRPS24 | -0.493621429 | 11.17933984 | -2.500297984 | 0.024156401 | 0.333298642 | -3.274338927 |
| NDUFA10 | -0.328321874 | 10.00711123 | -2.500009922 | 0.024170245 | 0.333298642 | -3.274808683 |
| PIGP | -0.542351172 | 7.090161707 | -2.499943539 | 0.024173436 | 0.333298642 | -3.274916935 |
| lnc-GPR132-1 | 1.2663185 | 1.735714554 | 2.499612816 | 0.024189342 | 0.333298642 | -3.275456234 |
| ANKRD40 | -0.390235144 | 7.938980964 | -2.499601445 | 0.024189889 | 0.333298642 | -3.275474775 |
| FICD | -0.183767109 | 7.031662186 | -2.499527161 | 0.024193463 | 0.333298642 | -3.275595903 |
| TMEM127 | 0.272236606 | 10.66459533 | 2.499457377 | 0.024196821 | 0.333298642 | -3.275709693 |
| LOC101930595 | 0.737663621 | 5.039755373 | 2.499227212 | 0.0242079 | 0.333298642 | -3.276084993 |
| PPP2R5B | 0.480650489 | 7.615329404 | 2.499023345 | 0.024217716 | 0.333298642 | -3.276417402 |
| FRY | 0.428098502 | 9.421991224 | 2.498837393 | 0.024226674 | 0.333298642 | -3.276720591 |
| LOC345051 | 0.933448202 | 3.096586595 | 2.498759922 | 0.024230406 | 0.333298642 | -3.276846903 |
| NUDCD1 | -0.695012272 | 2.802768664 | -2.49872583 | 0.024232049 | 0.333298642 | -3.276902488 |
| ZNF500 | -0.318353814 | 7.947140537 | -2.498455247 | 0.024245092 | 0.333346017 | -3.277343646 |
| PAXBP1 | -0.294585862 | 5.924319839 | -2.498014856 | 0.024266334 | 0.333405088 | -3.278061625 |
| NSUN4 | -0.174025181 | 7.773550106 | -2.497945125 | 0.024269699 | 0.333405088 | -3.278175304 |
| WDR82 | -0.293940309 | 10.30209999 | -2.497755492 | 0.024278852 | 0.333405088 | -3.27848445 |
| ATP2A1-AS1 | -0.888646973 | 4.388105353 | -2.497372422 | 0.024297353 | 0.333405088 | -3.27910892 |
| CHST11 | 0.435283462 | 7.106118066 | 2.497349127 | 0.024298478 | 0.333405088 | -3.279146893 |
| PLB1 | 0.690397961 | 7.581312079 | 2.497114411 | 0.024309821 | 0.333405088 | -3.279529502 |
| lnc-ATP2B2-1 | -0.584975149 | 1.220986075 | -2.496974438 | 0.024316588 | 0.333405088 | -3.279757667 |
| SLAMF7 | -0.681442475 | 10.66282897 | -2.496326113 | 0.024347953 | 0.333618048 | -3.280814413 |
| lnc-RGMA-3 | 1.032352438 | 1.79913675 | 2.496116591 | 0.024358098 | 0.333618048 | -3.281155906 |
| C20orf27 | -0.359793224 | 11.40204594 | -2.49604371 | 0.024361627 | 0.333618048 | -3.28127469 |
| MUC22 | 0.757111071 | 1.439004318 | 2.495754367 | 0.024375645 | 0.333618048 | -3.28174626 |
| lnc-RP11-701P16.2.1-2 | 0.393086106 | 1.274507866 | 2.49549026 | 0.024388447 | 0.333618048 | -3.282176685 |
| KRI1 | -0.248435104 | 9.471973271 | -2.495463272 | 0.024389756 | 0.333618048 | -3.282220667 |
| lnc-HNRNPA2B1-3 | -0.492724278 | 1.085187496 | -2.494999086 | 0.024412273 | 0.333670986 | -3.282977125 |
| PPARA | -0.47771808 | 4.999553185 | -2.49498738 | 0.024412841 | 0.333670986 | -3.282996201 |
| VGLL3 | -0.283549292 | 6.287809804 | -2.494643464 | 0.024429537 | 0.333767834 | -3.28355663 |
| SAMD10 | -0.349435252 | 7.011408164 | -2.494389708 | 0.024441863 | 0.333804923 | -3.283970122 |
| SRRM1 | -0.337646697 | 9.186417285 | -2.493978765 | 0.024461837 | 0.333850412 | -3.284639717 |
| lnc-MKRN3-3 | 0.597046156 | 1.412574776 | 2.49345602 | 0.024487268 | 0.333850412 | -3.285491429 |
| CHEK1 | -1.055399855 | 2.285519368 | -2.493105141 | 0.024504352 | 0.333850412 | -3.286063083 |
| CENPH | -0.721660882 | 4.026092933 | -2.492830012 | 0.024517755 | 0.333850412 | -3.286511306 |
| PSMD6-AS2 | 0.666093925 | 7.770703742 | 2.492783074 | 0.024520043 | 0.333850412 | -3.286587773 |
| GPR50 | 0.587033951 | 1.257520208 | 2.492658631 | 0.024526108 | 0.333850412 | -3.2867905 |
| NFKB2 | 0.313000089 | 5.717766926 | 2.492429823 | 0.024537264 | 0.333850412 | -3.287163238 |
| ARPC5L | -0.387798723 | 9.331285954 | -2.492193924 | 0.02454877 | 0.333850412 | -3.287547515 |
| LOC100288181 | -0.336375504 | 4.220718278 | -2.492099388 | 0.024553383 | 0.333850412 | -3.28770151 |
| ATP7A | 0.348161877 | 8.049368605 | 2.491907111 | 0.024562767 | 0.333850412 | -3.288014713 |
| ISY1-RAB43 | 0.56673184 | 4.543490269 | 2.491802869 | 0.024567856 | 0.333850412 | -3.288184511 |
| EXD2 | -0.741517416 | 1.712205468 | -2.491575962 | 0.024578937 | 0.333850412 | -3.288554108 |
| C11orf84 | -0.592715279 | 3.225242554 | -2.491530587 | 0.024581154 | 0.333850412 | -3.288628016 |
| LOC441268 | 0.64318149 | 10.09559698 | 2.491415495 | 0.024586777 | 0.333850412 | -3.288815477 |
| DNAJB1 | -0.261038187 | 8.077623016 | -2.491308747 | 0.024591993 | 0.333850412 | -3.288989347 |
| KLF13 | -0.268328695 | 12.2842071 | -2.491165424 | 0.024598998 | 0.333850412 | -3.289222783 |
| EHMT1-IT1 | -0.531938117 | 1.202755802 | -2.490725275 | 0.024620523 | 0.33390673 | -3.289939645 |
| SLC7A5 | -0.529395552 | 5.663955656 | -2.490536561 | 0.024629757 | 0.33390673 | -3.290246987 |
| ALDH3B2 | 0.895692973 | 2.509191002 | 2.490331062 | 0.024639817 | 0.33390673 | -3.290581655 |
| MSL1 | 0.532579113 | 10.9064365 | 2.490294534 | 0.024641605 | 0.33390673 | -3.290641143 |
| MRAP | -0.331376758 | 0.861028045 | -2.489749353 | 0.024668313 | 0.333977484 | -3.291528954 |
| CASP1 | 0.443722421 | 10.82080257 | 2.489660758 | 0.024672656 | 0.333977484 | -3.291673222 |
| MELK | -0.974480123 | 1.882381965 | -2.48959916 | 0.024675676 | 0.333977484 | -3.291773527 |
| TET2 | 0.701587494 | 8.790611089 | 2.488980903 | 0.024706006 | 0.334257725 | -3.292780238 |
| ARHGAP26-IT1 | 0.760626943 | 5.642500637 | 2.488104509 | 0.02474906 | 0.334539098 | -3.294207128 |
| FBXW11 | -0.232218805 | 9.058781516 | -2.48769277 | 0.024769312 | 0.334539098 | -3.294877434 |
| PAK1 | 0.417160509 | 9.992668849 | 2.487599917 | 0.024773881 | 0.334539098 | -3.295028593 |
| NKIRAS1 | -0.880277176 | 2.472081014 | -2.487493665 | 0.024779111 | 0.334539098 | -3.295201562 |
| lnc-STX8-2 | -0.546451173 | 3.82366855 | -2.487292725 | 0.024789003 | 0.334539098 | -3.295528667 |
| ISOC2 | -0.544797758 | 5.855296485 | -2.487284223 | 0.024789422 | 0.334539098 | -3.295542507 |
| CMSS1 | -0.567934952 | 8.006953359 | -2.487065117 | 0.024800214 | 0.334539098 | -3.295899173 |
| BCL6 | 0.829140131 | 11.69657579 | 2.486713958 | 0.024817519 | 0.334539098 | -3.296470777 |
| TCF12 | -0.341184414 | 6.645193123 | -2.486666046 | 0.024819881 | 0.334539098 | -3.296548763 |
| LOC100506124 | -0.611479107 | 5.622658313 | -2.486600177 | 0.024823129 | 0.334539098 | -3.296655979 |
| PRPF4 | -0.237203495 | 7.901352903 | -2.486254311 | 0.024840188 | 0.334639148 | -3.29721893 |
| IVD | -0.258662427 | 8.120893513 | -2.485499138 | 0.024877474 | 0.334898903 | -3.298447996 |
| LAMP5 | -0.908699368 | 6.264481238 | -2.485473208 | 0.024878755 | 0.334898903 | -3.298490197 |
| FLJ46836 | 0.64506398 | 1.464607612 | 2.485042409 | 0.024900051 | 0.334931145 | -3.299191274 |
| DOK3 | 0.792261824 | 11.01524347 | 2.484865655 | 0.024908793 | 0.334931145 | -3.299478909 |
| AFF1 | 0.651079387 | 6.525753551 | 2.484839598 | 0.024910082 | 0.334931145 | -3.299521313 |
| TNFSF13B | 0.859197355 | 10.32707324 | 2.484453733 | 0.024929178 | 0.334999567 | -3.300149212 |
| GBA | 0.411436272 | 7.292248763 | 2.484347005 | 0.024934462 | 0.334999567 | -3.300322879 |
| SERPINB1 | 0.377775299 | 11.31582949 | 2.48401341 | 0.024950986 | 0.33509194 | -3.300865685 |
| lnc-BNIP3-2 | 0.3857544 | 5.16310625 | 2.483337476 | 0.024984499 | 0.335250172 | -3.30196545 |
| SEC14L1 | 0.535299664 | 11.7420878 | 2.483312151 | 0.024985756 | 0.335250172 | -3.302006652 |
| OSGIN2 | 0.299009467 | 5.242117196 | 2.483191801 | 0.024991727 | 0.335250172 | -3.302202453 |
| LOC643072 | 0.66760511 | 8.770025907 | 2.482032846 | 0.025049304 | 0.335892791 | -3.304087826 |
| PNPLA1 | 0.766679574 | 1.508951723 | 2.481327378 | 0.025084413 | 0.336233759 | -3.30523532 |
| CEBPA | 0.376947206 | 9.063878491 | 2.480769189 | 0.025112226 | 0.336476699 | -3.306143174 |
| ZNF426 | -0.314787067 | 6.596952138 | -2.48053759 | 0.025123774 | 0.336501612 | -3.306519833 |
| SLITRK4 | 0.416284153 | 6.925387891 | 2.479430086 | 0.025179068 | 0.336537024 | -3.308320838 |
| STK26 | -0.434286789 | 9.77102724 | -2.479371756 | 0.025181984 | 0.336537024 | -3.308415686 |
| GPR56 | -0.758199119 | 12.12703748 | -2.479353862 | 0.025182878 | 0.336537024 | -3.308444782 |
| GIMAP1 | -0.399926417 | 10.73357744 | -2.479324303 | 0.025184356 | 0.336537024 | -3.308492846 |
| XLOC_l2_002160 | -0.410186754 | 1.188296557 | -2.479186244 | 0.025191258 | 0.336537024 | -3.308717332 |
| ZNF800 | -0.282285262 | 5.985323075 | -2.478857566 | 0.025207698 | 0.336537024 | -3.309251752 |
| SLC25A12 | -0.358587474 | 7.807190417 | -2.47874265 | 0.025213448 | 0.336537024 | -3.309438595 |
| AKR1C8P | -0.835293558 | 2.844112399 | -2.478741194 | 0.025213521 | 0.336537024 | -3.309440962 |
| CBS | 1.227010187 | 6.803477526 | 2.478389004 | 0.025231152 | 0.336537024 | -3.310013573 |
| HELZ | -0.284320957 | 9.497162061 | -2.4783133 | 0.025234943 | 0.336537024 | -3.310136652 |
| lnc-IL1R2-1 | 1.581016079 | 4.55840762 | 2.478221328 | 0.02523955 | 0.336537024 | -3.310286181 |
| ACOT9 | 0.439709033 | 10.08904795 | 2.478100825 | 0.025245587 | 0.336537024 | -3.310482089 |
| FCF1 | -0.225206964 | 6.183215003 | -2.477898508 | 0.025255726 | 0.336537024 | -3.310811002 |
| TERF1 | -0.227466278 | 9.031432834 | -2.477771761 | 0.02526208 | 0.336537024 | -3.311017053 |
| ARPC4-TTLL3 | 0.370541133 | 8.789090866 | 2.476946047 | 0.025303509 | 0.33690927 | -3.312359318 |
| SPIN4 | -0.670698726 | 5.209971628 | -2.476783356 | 0.02531168 | 0.33690927 | -3.312623767 |
| FAM129A | 0.658996945 | 12.43939503 | 2.476146611 | 0.025343682 | 0.33690927 | -3.313658716 |
| LINC01176 | 0.521881421 | 5.098073661 | 2.476091513 | 0.025346453 | 0.33690927 | -3.313748266 |
| lnc-KIAA1147-3 | 1.115935696 | 2.139380742 | 2.476034235 | 0.025349334 | 0.33690927 | -3.31384136 |
| lnc-PRDM11-2 | 0.816048522 | 1.83803121 | 2.475881996 | 0.025356993 | 0.33690927 | -3.314088786 |
| LOC102725284 | -0.882945609 | 9.544603223 | -2.475473806 | 0.025377539 | 0.33690927 | -3.314752175 |
| ECE1 | 0.723423204 | 6.679232915 | 2.475416141 | 0.025380443 | 0.33690927 | -3.314845888 |
| SLC26A1 | -0.47369483 | 2.893645237 | -2.475131961 | 0.025394757 | 0.33690927 | -3.31530771 |
| KIF21A | -0.93003449 | 2.318306612 | -2.474824852 | 0.025410236 | 0.33690927 | -3.315806771 |
| ZNF341-AS1 | 0.372177032 | 4.621218443 | 2.474647749 | 0.025419166 | 0.33690927 | -3.31609456 |
| lnc-RBPMS-1 | 0.964231197 | 6.761488473 | 2.474581616 | 0.025422501 | 0.33690927 | -3.316202021 |
| TMEM5 | -0.30048315 | 6.80032732 | -2.47452941 | 0.025425135 | 0.33690927 | -3.316286853 |
| lnc-AFG3L2-1 | 0.341965243 | 5.172566061 | 2.474515556 | 0.025425834 | 0.33690927 | -3.316309365 |
| NTN5 | -0.667144746 | 4.014782827 | -2.474149472 | 0.025444307 | 0.337005606 | -3.316904209 |
| HYPK | -0.274343769 | 9.690921988 | -2.47398697 | 0.025452511 | 0.337005606 | -3.317168244 |
| SMPD4 | -0.383006271 | 10.65661621 | -2.473488449 | 0.025477695 | 0.337091182 | -3.317978215 |
| LOC101927070 | -0.546082232 | 5.526073504 | -2.47339584 | 0.025482377 | 0.337091182 | -3.318128674 |
| TAS2R60 | 0.890776726 | 2.20810093 | 2.473227868 | 0.025490869 | 0.337091182 | -3.318401569 |
| ZBTB41 | -0.721710294 | 3.96415598 | -2.473090853 | 0.025497798 | 0.337091182 | -3.318624165 |
| LOC101927372 | -0.439632332 | 7.287113081 | -2.472460254 | 0.025529713 | 0.337325292 | -3.319648586 |
| NHS | 0.711412096 | 4.906303507 | 2.4723572 | 0.025534932 | 0.337325292 | -3.319815992 |
| S100A8 | 0.539785645 | 15.07910185 | 2.472100844 | 0.02554792 | 0.337368537 | -3.320232415 |
| LEPROTL1 | -0.47364722 | 10.5368816 | -2.469827658 | 0.025663359 | 0.338716702 | -3.3239243 |
| ATF1 | -0.452435875 | 8.290651443 | -2.469706655 | 0.025669518 | 0.338716702 | -3.324120788 |
| lnc-SLC35F5-8 | 0.682918022 | 1.785810695 | 2.469066754 | 0.025702111 | 0.338881064 | -3.325159817 |
| OTULIN | -0.410440685 | 5.019535434 | -2.469012764 | 0.025704863 | 0.338881064 | -3.325247479 |
| HIST1H3E | 0.463748691 | 11.24009331 | 2.468600364 | 0.025725891 | 0.338881064 | -3.325917051 |
| RCN2 | -0.413089255 | 8.921868188 | -2.468546662 | 0.02572863 | 0.338881064 | -3.326004238 |
| lnc-ATF7IP2-1 | 0.473032799 | 3.928792914 | 2.468504877 | 0.025730762 | 0.338881064 | -3.326072078 |
| NACA | -0.452279404 | 12.74751195 | -2.468016686 | 0.02575568 | 0.33908066 | -3.326864642 |
| TGS1 | -0.335527914 | 8.680974519 | -2.46755959 | 0.025779032 | 0.339133302 | -3.327606674 |
| PEX11A | -0.877926395 | 3.585303831 | -2.467496639 | 0.02578225 | 0.339133302 | -3.327708862 |
| lnc-PARP16-1 | 0.570538027 | 1.134784816 | 2.467102246 | 0.025802417 | 0.339133302 | -3.32834906 |
| DIP2A | -0.519185758 | 4.553932951 | -2.466787045 | 0.025818546 | 0.339133302 | -3.328860684 |
| HTR4 | 0.54382212 | 1.162737028 | 2.466545139 | 0.02583093 | 0.339133302 | -3.329253321 |
| POM121L10P | 0.805989909 | 1.411895761 | 2.466289381 | 0.02584403 | 0.339133302 | -3.329668428 |
| lnc-ACCN1-1 | 1.189031042 | 2.093463537 | 2.466106973 | 0.025853377 | 0.339133302 | -3.329964473 |
| LOC101927270 | 1.000332591 | 9.636835961 | 2.466011618 | 0.025858264 | 0.339133302 | -3.330119231 |
| ENTPD1 | 0.781294262 | 8.941641518 | 2.465978191 | 0.025859978 | 0.339133302 | -3.330173482 |
| RNF11 | 0.290905205 | 10.41994073 | 2.465878592 | 0.025865084 | 0.339133302 | -3.330335123 |
| ZNF529 | -0.521706741 | 7.016453554 | -2.465075629 | 0.025906284 | 0.339133302 | -3.331638189 |
| BCL7C | -0.361806201 | 6.85524577 | -2.464832796 | 0.025918757 | 0.339133302 | -3.332032234 |
| lnc-AKTIP-2 | 0.9407704 | 3.002265874 | 2.464553351 | 0.025933116 | 0.339133302 | -3.332485672 |
| ABCF1 | -0.314125175 | 8.290773792 | -2.464527346 | 0.025934453 | 0.339133302 | -3.332527867 |
| ANKEF1 | -0.638898929 | 4.252289706 | -2.464474521 | 0.025937168 | 0.339133302 | -3.332613582 |
| NLK | -0.207942773 | 8.361734172 | -2.464344265 | 0.025943865 | 0.339133302 | -3.332824932 |
| HSPD1 | -0.499345795 | 10.30985636 | -2.464322173 | 0.025945001 | 0.339133302 | -3.332860777 |
| ARL5A | -0.329126018 | 7.007692724 | -2.464235935 | 0.025949436 | 0.339133302 | -3.333000701 |
| CDC42EP3 | 0.737457971 | 3.916220824 | 2.464209964 | 0.025950772 | 0.339133302 | -3.333042841 |
| CCDC120 | -0.3055322 | 5.845686156 | -2.463973232 | 0.025962951 | 0.339133302 | -3.333426938 |
| NME8 | 0.740214762 | 8.39625025 | 2.463844969 | 0.025969551 | 0.339133302 | -3.333635038 |
| LEO1 | -0.422621311 | 6.385924449 | -2.46332453 | 0.02599635 | 0.339133302 | -3.334479388 |
| lnc-CLEC3A-4 | 0.255638428 | 5.031473811 | 2.463046739 | 0.026010666 | 0.339133302 | -3.334930043 |
| LDHB | -0.582810492 | 12.46021491 | -2.462812455 | 0.026022745 | 0.339133302 | -3.335310105 |
| LRP12 | -0.521477719 | 4.226244353 | -2.462712009 | 0.026027925 | 0.339133302 | -3.335473047 |
| lnc-NRIP2-2 | -0.480772283 | 3.653529916 | -2.462624367 | 0.026032446 | 0.339133302 | -3.335615216 |
| CDK4 | -0.432561076 | 8.161819799 | -2.462255422 | 0.026051485 | 0.339133302 | -3.336213685 |
| DIP2A-IT1 | -0.592842997 | 3.544962424 | -2.461835544 | 0.026073169 | 0.339133302 | -3.336894736 |
| ABCB7 | -0.263568799 | 7.936876139 | -2.461497047 | 0.026090662 | 0.339133302 | -3.337443753 |
| POLR3H | -0.314664776 | 8.786326251 | -2.461079085 | 0.026112278 | 0.339133302 | -3.338121619 |
| PARP2 | -0.437223803 | 6.79106771 | -2.461027242 | 0.02611496 | 0.339133302 | -3.338205697 |
| LOC101929128 | 0.917299278 | 7.493602677 | 2.460831044 | 0.026125114 | 0.339133302 | -3.338523883 |
| C3orf17 | -0.445521188 | 6.379249094 | -2.460648088 | 0.026134585 | 0.339133302 | -3.338820584 |
| NUP133 | -0.335273013 | 7.906501169 | -2.46063809 | 0.026135103 | 0.339133302 | -3.338836799 |
| ZBED3 | -0.40405443 | 8.130206438 | -2.460367016 | 0.026149143 | 0.339133302 | -3.339276387 |
| lnc-FCRL3-1 | -0.951023965 | 4.626116356 | -2.460313581 | 0.026151911 | 0.339133302 | -3.339363037 |
| DOLPP1 | -0.410387698 | 6.401015342 | -2.459892374 | 0.026173743 | 0.339133302 | -3.34004605 |
| XLOC_l2_001559 | 0.974434271 | 9.522819978 | 2.459623605 | 0.026187683 | 0.339133302 | -3.340481852 |
| ZBED4 | -0.364459915 | 5.480936566 | -2.45955703 | 0.026191137 | 0.339133302 | -3.340589799 |
| GTF2H4 | -0.677155575 | 2.753723739 | -2.459541549 | 0.02619194 | 0.339133302 | -3.340614901 |
| C10orf131 | 0.677847432 | 1.052467717 | 2.459511038 | 0.026193523 | 0.339133302 | -3.340664373 |
| TXN2 | -0.250850046 | 8.624292695 | -2.459469991 | 0.026195653 | 0.339133302 | -3.340730926 |
| lnc-CHSY1-4 | 0.876610625 | 1.696056263 | 2.459403529 | 0.026199102 | 0.339133302 | -3.340838688 |
| CD3D | -0.638019798 | 12.17027282 | -2.459341237 | 0.026202336 | 0.339133302 | -3.340939687 |
| CCT8 | -0.448245424 | 9.786276701 | -2.459131699 | 0.026213214 | 0.339133302 | -3.341279424 |
| MCAT | 0.451190662 | 7.881947273 | 2.458651429 | 0.026238164 | 0.339133302 | -3.342058073 |
| lnc-AGMAT-3 | -0.448111571 | 3.092244968 | -2.458549309 | 0.026243472 | 0.339133302 | -3.34222363 |
| CDK2AP2 | -0.351219714 | 12.05716601 | -2.458468482 | 0.026247674 | 0.339133302 | -3.342354665 |
| ATP5G2 | -0.322211517 | 12.51823402 | -2.458383709 | 0.026252082 | 0.339133302 | -3.342492097 |
| KCTD10 | -0.353270647 | 6.042611787 | -2.458148651 | 0.026264308 | 0.339133302 | -3.342873154 |
| RNASEH2A | -0.340502332 | 7.855479126 | -2.458113005 | 0.026266162 | 0.339133302 | -3.34293094 |
| lnc-TIGIT-1 | -0.438046016 | 7.044948145 | -2.458088228 | 0.026267451 | 0.339133302 | -3.342971106 |
| SLC35G1 | -0.615686057 | 1.036275363 | -2.457604276 | 0.026292642 | 0.339332389 | -3.343755606 |
| ECRP | 0.97355654 | 5.315969818 | 2.457090394 | 0.026319416 | 0.339551751 | -3.344588562 |
| ZNF236 | -0.438540514 | 5.579740185 | -2.456605637 | 0.026344696 | 0.339658691 | -3.345374251 |
| LAT | -0.30902874 | 11.60794481 | -2.456162182 | 0.026367842 | 0.339658691 | -3.346092949 |
| lnc-NUCB1-1 | 0.435424308 | 5.560656513 | 2.456109558 | 0.02637059 | 0.339658691 | -3.346178233 |
| KRTAP5-7 | 0.632872562 | 3.857764159 | 2.455818859 | 0.026385776 | 0.339658691 | -3.346649335 |
| KCNG4 | 0.79443368 | 1.810060286 | 2.455793512 | 0.0263871 | 0.339658691 | -3.34669041 |
| RRP1B | -0.453733299 | 7.383787326 | -2.455586304 | 0.026397929 | 0.339658691 | -3.347026195 |
| ZNF561 | -0.293089963 | 8.006426981 | -2.45543613 | 0.026405781 | 0.339658691 | -3.347269547 |
| MYO1F | 0.538109196 | 13.16310804 | 2.45543299 | 0.026405945 | 0.339658691 | -3.347274636 |
| LOC100132368 | 0.866485936 | 2.343119007 | 2.454897488 | 0.02643396 | 0.339727948 | -3.348142359 |
| AMPD2 | 0.455173105 | 8.371538072 | 2.45477893 | 0.026440166 | 0.339727948 | -3.34833446 |
| LTBP4 | -0.351302907 | 9.569128795 | -2.454406109 | 0.026459691 | 0.339727948 | -3.348938524 |
| PYGO2 | -0.272928334 | 8.54614917 | -2.454186107 | 0.026471219 | 0.339727948 | -3.349294969 |
| lnc-FAM182B-1 | 0.518025512 | 4.640419363 | 2.454156857 | 0.026472752 | 0.339727948 | -3.349342358 |
| CRELD1 | -0.245194529 | 7.748423841 | -2.454028121 | 0.026479501 | 0.339727948 | -3.349550929 |
| MCM5 | -0.329429341 | 8.119382237 | -2.454022361 | 0.026479803 | 0.339727948 | -3.349560261 |
| UHRF1BP1L | 0.407518049 | 6.526034454 | 2.453402204 | 0.026512335 | 0.339853107 | -3.350564943 |
| PRDM12 | -0.436910026 | 0.88664674 | -2.45339263 | 0.026512838 | 0.339853107 | -3.350580454 |
| LOC100130093 | -0.573600724 | 4.713396309 | -2.453276862 | 0.026518915 | 0.339853107 | -3.350767993 |
| HNRNPUL2 | -0.338801806 | 5.55093136 | -2.452895203 | 0.02653896 | 0.339984534 | -3.35138624 |
| CD81-AS1 | -0.715680798 | 1.291346877 | -2.452660686 | 0.026551284 | 0.340016992 | -3.351766117 |
| PCDHB9 | -0.625649673 | 1.175730264 | -2.452361415 | 0.026567018 | 0.340070379 | -3.352250864 |
| SEC31A | -0.239532994 | 8.72449056 | -2.45216306 | 0.026577452 | 0.340070379 | -3.352572141 |
| lnc-ATP2B3-1 | -0.57518132 | 5.455380486 | -2.45202288 | 0.026584828 | 0.340070379 | -3.352799184 |
| LOC100133331 | 0.929848742 | 9.100686507 | 2.45176034 | 0.026598648 | 0.340121882 | -3.353224398 |
| LOC100652758 | -0.510257697 | 3.698019411 | -2.450542864 | 0.026662822 | 0.340662134 | -3.355196022 |
| TOP2B | -0.398573939 | 10.22560176 | -2.449756868 | 0.026704331 | 0.340662134 | -3.356468705 |
| LOC100506476 | -0.473582677 | 3.579882668 | -2.449444617 | 0.026720838 | 0.340662134 | -3.35697426 |
| CSF2RA | 0.608339486 | 6.796787833 | 2.449143344 | 0.026736774 | 0.340662134 | -3.357462019 |
| HNRNPR | -0.35534099 | 8.676219572 | -2.448762303 | 0.026756942 | 0.340662134 | -3.35807889 |
| lnc-TTC7B-4 | 0.898265183 | 2.582567704 | 2.448646892 | 0.026763053 | 0.340662134 | -3.358265723 |
| lnc-IL1R1-1 | 1.340781737 | 2.931338899 | 2.448614414 | 0.026764773 | 0.340662134 | -3.3583183 |
| LRP8 | -0.537249831 | 6.015520343 | -2.448607946 | 0.026765116 | 0.340662134 | -3.35832877 |
| DCHS1 | -0.94598141 | 4.212458305 | -2.448290487 | 0.026781934 | 0.340662134 | -3.35884267 |
| LOC101929563 | 0.433840212 | 3.197473614 | 2.448189836 | 0.026787269 | 0.340662134 | -3.359005598 |
| lnc-IL12RB2-1 | -0.570188468 | 3.259702341 | -2.447953563 | 0.026799795 | 0.340662134 | -3.359388052 |
| HCFC1 | -0.404158773 | 3.61061691 | -2.447663393 | 0.026815187 | 0.340662134 | -3.359857733 |
| LMAN1L | 0.654042225 | 4.526773492 | 2.447545346 | 0.026821451 | 0.340662134 | -3.360048802 |
| RNF141 | 0.45709647 | 9.005068828 | 2.447536242 | 0.026821934 | 0.340662134 | -3.360063538 |
| TSTD2 | -0.472290446 | 5.57981042 | -2.447299002 | 0.026834527 | 0.340662134 | -3.360447521 |
| BEST1 | 0.709419996 | 6.364468812 | 2.447263923 | 0.026836389 | 0.340662134 | -3.360504296 |
| DMRTC1 | -0.693750316 | 4.825066014 | -2.44696819 | 0.026852096 | 0.340662134 | -3.360982932 |
| IGSF3 | 0.519533101 | 3.641340069 | 2.446877984 | 0.026856889 | 0.340662134 | -3.361128923 |
| VAMP3 | 0.260624277 | 8.576330583 | 2.44673066 | 0.026864718 | 0.340662134 | -3.361367352 |
| lnc-LAT2-1 | 0.265336535 | 4.666348436 | 2.446683667 | 0.026867216 | 0.340662134 | -3.361443403 |
| KRR1 | -0.413003535 | 4.55731441 | -2.446672042 | 0.026867834 | 0.340662134 | -3.361462217 |
| LINC01101 | 0.358923668 | 4.875879858 | 2.446524314 | 0.026875688 | 0.340662134 | -3.361701293 |
| KMT2A | -0.307184187 | 10.81959077 | -2.44651159 | 0.026876364 | 0.340662134 | -3.361721884 |
| lnc-CCDC152-1 | 0.268082974 | 1.164698366 | 2.446326246 | 0.026886221 | 0.340662134 | -3.362021827 |
| SYTL2 | -0.616095936 | 7.485287265 | -2.445938433 | 0.026906857 | 0.340662134 | -3.3626494 |
| TMEM67 | -0.322070742 | 0.888758242 | -2.445908396 | 0.026908456 | 0.340662134 | -3.362698006 |
| HIST1H2BM | 0.569727781 | 7.676736608 | 2.445783996 | 0.026915078 | 0.340662134 | -3.362899305 |
| lnc-ANO1-1 | 0.835830286 | 7.125546924 | 2.445775222 | 0.026915546 | 0.340662134 | -3.362913503 |
| lnc-USP35-1 | -0.422223208 | 11.78004097 | -2.44472421 | 0.026971562 | 0.341139859 | -3.364614064 |
| RAB23 | -1.541767284 | 1.943378474 | -2.444523094 | 0.026982294 | 0.341139859 | -3.364939444 |
| POLH | -0.43819344 | 6.122564119 | -2.444381061 | 0.026989875 | 0.341139859 | -3.365169229 |
| THEMIS | -0.444736897 | 8.083616379 | -2.444267326 | 0.026995948 | 0.341139859 | -3.36535323 |
| TAF1B | -0.454263091 | 7.062290926 | -2.444081165 | 0.02700589 | 0.341139859 | -3.365654394 |
| PUF60 | -0.209597595 | 9.356840952 | -2.443962555 | 0.027012226 | 0.341139859 | -3.365846272 |
| RASAL1 | 0.894842801 | 4.397359276 | 2.4430909 | 0.027058834 | 0.341604253 | -3.367256268 |
| GSTM1 | -0.940899088 | 3.618533161 | -2.442867038 | 0.027070816 | 0.34163134 | -3.36761836 |
| DDX18 | -0.429198244 | 8.869780091 | -2.442435531 | 0.027093927 | 0.341765443 | -3.368316278 |
| lnc-CDYL2-3 | 0.996434991 | 1.468599308 | 2.442218343 | 0.027105566 | 0.341765443 | -3.36866754 |
| AP1B1 | -0.290795045 | 12.33544294 | -2.441997937 | 0.027117383 | 0.341765443 | -3.369023995 |
| lnc-FBXO15-4 | 0.688010547 | 4.390614991 | 2.44176427 | 0.027129916 | 0.341765443 | -3.369401884 |
| LOC101928823 | -0.858547873 | 2.147386374 | -2.441659097 | 0.027135559 | 0.341765443 | -3.369571965 |
| SIGIRR | -0.289517772 | 10.47046737 | -2.441567268 | 0.027140486 | 0.341765443 | -3.369720467 |
| BCL2A1 | 0.768003555 | 10.91887674 | 2.4409549 | 0.027173369 | 0.342055497 | -3.370710702 |
| lnc-METTL13-1 | -0.639867548 | 1.977246594 | -2.440279443 | 0.027209684 | 0.342082322 | -3.371802852 |
| KRTAP4-3 | 0.522315784 | 2.999844818 | 2.439313864 | 0.027261676 | 0.342082322 | -3.37336391 |
| IFITM4P | 0.703698984 | 12.93938832 | 2.439200378 | 0.027267793 | 0.342082322 | -3.373547368 |
| ZNF10 | -0.589163075 | 5.007640211 | -2.43908774 | 0.027273865 | 0.342082322 | -3.373729453 |
| LOC101927507 | 0.628615181 | 3.250499593 | 2.438954283 | 0.027281062 | 0.342082322 | -3.37394519 |
| MDFIC | -0.529199315 | 7.445790012 | -2.438741234 | 0.027292554 | 0.342082322 | -3.374289578 |
| ZNF606 | -0.56910086 | 3.73650952 | -2.438716876 | 0.027293868 | 0.342082322 | -3.374328951 |
| lnc-H2AFJ-2 | 1.200013741 | 1.540988236 | 2.438589291 | 0.027300753 | 0.342082322 | -3.374535183 |
| TRIM31-AS1 | 0.504669537 | 4.664931325 | 2.438569356 | 0.027301829 | 0.342082322 | -3.374567406 |
| lnc-HEATR1-1 | -1.13873874 | 4.792543586 | -2.438441611 | 0.027308724 | 0.342082322 | -3.374773892 |
| lnc-MFSD4-2 | 1.136668632 | 2.301223719 | 2.438057808 | 0.02732945 | 0.342082322 | -3.375394246 |
| lnc-OR52B2-1 | 0.795792064 | 1.272093499 | 2.438023332 | 0.027331312 | 0.342082322 | -3.375449968 |
| PP7080 | -0.625781145 | 6.523778491 | -2.437734767 | 0.027346906 | 0.342082322 | -3.375916358 |
| lnc-AC099552.4.1-1 | 0.608542201 | 4.147009102 | 2.437474464 | 0.02736098 | 0.342082322 | -3.376337053 |
| lnc-LRRC36-1 | 0.652843783 | 4.881871857 | 2.437415577 | 0.027364165 | 0.342082322 | -3.376432223 |
| SLC6A5 | 0.741060063 | 2.994648354 | 2.437094244 | 0.02738155 | 0.342082322 | -3.376951525 |
| SRPRB | -0.422568477 | 8.137540699 | -2.436832836 | 0.0273957 | 0.342082322 | -3.377373967 |
| lnc-ANGPT2-2 | -0.961087336 | 2.126146546 | -2.43676345 | 0.027399458 | 0.342082322 | -3.377486092 |
| TMEM177 | -0.570342671 | 6.433968517 | -2.436641144 | 0.027406082 | 0.342082322 | -3.377683734 |
| lnc-MRPL14-1 | -0.824639599 | 2.860656403 | -2.436505349 | 0.027413438 | 0.342082322 | -3.377903168 |
| lnc-C5orf17-5 | 0.464249608 | 0.892424008 | 2.436329051 | 0.027422991 | 0.342082322 | -3.378188045 |
| NVL | -0.404470699 | 9.245719077 | -2.436293283 | 0.027424929 | 0.342082322 | -3.378245841 |
| RPL7L1 | -0.267129719 | 7.079267662 | -2.436290158 | 0.027425099 | 0.342082322 | -3.378250891 |
| PLEKHH2 | 0.865938577 | 1.696103679 | 2.436264113 | 0.027426511 | 0.342082322 | -3.378292976 |
| SLC19A1 | 0.606689056 | 8.428065885 | 2.436227004 | 0.027428522 | 0.342082322 | -3.378352938 |
| RPE | -0.302779661 | 5.885078795 | -2.436100583 | 0.027435376 | 0.342082322 | -3.378557211 |
| lnc-RBPJ-1 | 0.71900077 | 1.400024158 | 2.435784926 | 0.027452496 | 0.342082322 | -3.379067241 |
| BNIP3 | -0.446991131 | 8.189825578 | -2.43562126 | 0.027461376 | 0.342082322 | -3.379331678 |
| LRP5L | -0.306248157 | 5.171748594 | -2.435577688 | 0.027463741 | 0.342082322 | -3.379402078 |
| CS | -0.310022577 | 11.2956208 | -2.435410447 | 0.027472819 | 0.342082322 | -3.379672281 |
| PHRF1 | -0.322288129 | 11.65199589 | -2.435262664 | 0.027480843 | 0.342082322 | -3.379911043 |
| lnc-VCL-1 | -0.79693129 | 1.843007019 | -2.43447144 | 0.027523842 | 0.342494817 | -3.381189272 |
| PFN2 | -0.709563677 | 1.553988552 | -2.434008094 | 0.027549052 | 0.342507788 | -3.38193774 |
| CHD3 | -0.309978529 | 10.34811124 | -2.433877525 | 0.02755616 | 0.342507788 | -3.382148646 |
| lnc-C1orf124-2 | 0.421529391 | 4.647174505 | 2.433737002 | 0.027563812 | 0.342507788 | -3.382375626 |
| DLG1 | -0.243909542 | 8.305193603 | -2.433727447 | 0.027564332 | 0.342507788 | -3.38239106 |
| IL1RN | 0.885163601 | 12.01350088 | 2.433491102 | 0.027577207 | 0.342545205 | -3.382772803 |
| DIAPH1 | -0.261471612 | 12.59131025 | -2.43262267 | 0.027624562 | 0.343001253 | -3.384175379 |
| TM4SF19 | -1.191992108 | 2.616837028 | -2.432455724 | 0.027633674 | 0.343001253 | -3.384444986 |
| RFC5 | -0.349654904 | 7.631886706 | -2.432092288 | 0.027653521 | 0.343052329 | -3.38503189 |
| OSM | 0.916963924 | 6.950731388 | 2.432018644 | 0.027657545 | 0.343052329 | -3.385150811 |
| lnc-CDKAL1-2 | 0.827043816 | 2.495819219 | 2.431761567 | 0.027671593 | 0.343104048 | -3.385565934 |
| PRMT1 | -0.372530022 | 6.750126968 | -2.431293451 | 0.027697193 | 0.343298894 | -3.386321799 |
| NR2F1-AS1 | 0.571304286 | 1.18510462 | 2.431024073 | 0.027711934 | 0.343357325 | -3.386756736 |
| MARK4 | 0.295100813 | 4.10287776 | 2.430846054 | 0.02772168 | 0.343357325 | -3.387044156 |
| DBR1 | -0.238756602 | 6.085233064 | -2.430481164 | 0.027741666 | 0.343397081 | -3.387633264 |
| HDAC1 | -0.278824157 | 10.46338972 | -2.430282101 | 0.027752576 | 0.343397081 | -3.387954634 |
| PDE6B | -0.57073292 | 3.782800093 | -2.430246036 | 0.027754552 | 0.343397081 | -3.388012856 |
| UBE2D2 | -0.35998489 | 8.547157678 | -2.429473627 | 0.027796924 | 0.343798851 | -3.389259743 |
| BOLA3 | -0.497896566 | 9.154754147 | -2.42902123 | 0.027821769 | 0.343963172 | -3.389989972 |
| GPATCH4 | -0.37620996 | 7.999053185 | -2.428871128 | 0.027830018 | 0.343963172 | -3.390232246 |
| PRR22 | -0.614042474 | 3.262350691 | -2.428395329 | 0.027856178 | 0.344127422 | -3.391000176 |
| ZNF16 | -0.609478752 | 2.60866872 | -2.428227657 | 0.027865403 | 0.344127422 | -3.391270782 |
| lnc-ADAM11-1 | 0.696477199 | 1.880071106 | 2.428089003 | 0.027873033 | 0.344127422 | -3.39149455 |
| NAP1L4 | -0.168182782 | 6.062318304 | -2.427628128 | 0.02789841 | 0.344305958 | -3.392238307 |
| DERL1 | -0.330774688 | 6.491596984 | -2.427279404 | 0.027917626 | 0.344305958 | -3.392801039 |
| lnc-C11orf96-1 | -0.312646596 | 3.813836706 | -2.427123006 | 0.027926248 | 0.344305958 | -3.393053407 |
| LOC102723390 | -0.509805639 | 4.745200137 | -2.427106668 | 0.027927149 | 0.344305958 | -3.393079771 |
| HHIP | 0.923071818 | 1.627399224 | 2.426885649 | 0.027939338 | 0.344334003 | -3.393436402 |
| MAK | 0.999374018 | 6.500874737 | 2.426567489 | 0.027956894 | 0.344428141 | -3.393949757 |
| lnc-HES1-2 | 0.550167458 | 3.566951917 | 2.426200992 | 0.02797713 | 0.34455522 | -3.394541074 |
| LRRC61 | -0.511468404 | 4.379521615 | -2.425869425 | 0.027995449 | 0.344658611 | -3.395076006 |
| LOC102724679 | 0.744515229 | 5.293607749 | 2.425612484 | 0.028009653 | 0.344680092 | -3.395490519 |
| SLC2A3 | 0.763390947 | 6.343057919 | 2.425478849 | 0.028017043 | 0.344680092 | -3.395706104 |
| YTHDF1 | -0.216684913 | 10.68267557 | -2.424725786 | 0.028058722 | 0.345070618 | -3.396920878 |
| LOC284240 | 0.650730949 | 1.510234696 | 2.42397201 | 0.0281005 | 0.345425667 | -3.398136662 |
| lnc-OCM-1 | 0.52170046 | 4.60455568 | 2.423751887 | 0.028112711 | 0.345425667 | -3.398491678 |
| SKA3 | -1.225579048 | 2.082181129 | -2.423462634 | 0.028128765 | 0.345425667 | -3.398958167 |
| lnc-CAP2-1 | 1.197298653 | 2.28216677 | 2.423344409 | 0.028135329 | 0.345425667 | -3.399148828 |
| GABPB2 | -0.383049132 | 6.497543954 | -2.423308515 | 0.028137323 | 0.345425667 | -3.399206714 |
| ANKRD16 | -0.381077332 | 6.605067899 | -2.423092176 | 0.028149339 | 0.34542876 | -3.39955559 |
| MTVR2 | 0.789148851 | 5.333755923 | 2.42294589 | 0.028157467 | 0.34542876 | -3.39979149 |
| lnc-CORIN-2 | -0.451601856 | 1.016828484 | -2.42215808 | 0.028201278 | 0.345844057 | -3.401061818 |
| XPO6 | 0.775545855 | 8.541992451 | 2.421525117 | 0.028236524 | 0.346050966 | -3.402082348 |
| lnc-CTSZ-1 | 0.868399943 | 2.441471308 | 2.42149723 | 0.028238078 | 0.346050966 | -3.402127307 |
| HAUS4 | 0.623307218 | 6.273557849 | 2.421234793 | 0.028252704 | 0.346055031 | -3.402550405 |
| CX3CR1 | -0.598049958 | 12.73836621 | -2.420526451 | 0.028292219 | 0.346055031 | -3.403692299 |
| AP2B1 | -0.365242282 | 6.630635295 | -2.420505389 | 0.028293395 | 0.346055031 | -3.403726251 |
| GCFC2 | -0.45651353 | 6.515934398 | -2.420391397 | 0.028299759 | 0.346055031 | -3.403910001 |
| CXorf23 | 0.35272443 | 5.457936811 | 2.420339382 | 0.028302663 | 0.346055031 | -3.403993845 |
| OR2A42 | -0.628744581 | 1.63187839 | -2.420120846 | 0.028314869 | 0.346055031 | -3.404346105 |
| WDHD1 | -0.638721519 | 4.554005612 | -2.419846689 | 0.028330189 | 0.346055031 | -3.404788003 |
| C21orf33 | -0.328659345 | 9.840880294 | -2.419779432 | 0.028333948 | 0.346055031 | -3.404896408 |
| lnc-PITPNC1-1 | -0.334804527 | 6.052247268 | -2.419487922 | 0.028350248 | 0.346055031 | -3.405366251 |
| EDC4 | -0.235382759 | 10.30945746 | -2.419290987 | 0.028361264 | 0.346055031 | -3.40568365 |
| ST6GALNAC6 | -0.356864919 | 7.129259112 | -2.41920748 | 0.028365937 | 0.346055031 | -3.405818235 |
| IGF1 | -1.477155884 | 3.178497418 | -2.419146341 | 0.028369358 | 0.346055031 | -3.405916769 |
| MRPL55 | -0.251391628 | 9.638120955 | -2.419126349 | 0.028370477 | 0.346055031 | -3.405948989 |
| PATZ1 | -0.426685481 | 3.813265015 | -2.418849972 | 0.028385949 | 0.346055031 | -3.406394397 |
| FGFBP1 | -0.940882921 | 1.358016563 | -2.418815643 | 0.028387872 | 0.346055031 | -3.40644972 |
| GAPT | 0.353546034 | 6.728009251 | 2.418363004 | 0.02841323 | 0.346242627 | -3.407179146 |
| DDX28 | -0.35168263 | 9.354965186 | -2.418052342 | 0.028430647 | 0.346333347 | -3.407679746 |
| SHMT1 | -0.584444974 | 4.173530491 | -2.417669408 | 0.02845213 | 0.346357801 | -3.408296774 |
| ERLEC1 | -0.418179277 | 8.69316232 | -2.417513634 | 0.028460873 | 0.346357801 | -3.408547763 |
| SNAP23 | 0.308870318 | 5.891475996 | 2.417313471 | 0.028472112 | 0.346357801 | -3.408870268 |
| FBXO25 | -0.389735616 | 9.241311153 | -2.417305734 | 0.028472546 | 0.346357801 | -3.408882732 |
| SMAD3 | -0.417167876 | 8.844155493 | -2.417100985 | 0.028484047 | 0.346376378 | -3.409212614 |
| C5AR1 | 0.75907282 | 9.99195309 | 2.416817842 | 0.028499958 | 0.346448557 | -3.409668782 |
| lnc-MUC5B-1 | 0.91987225 | 3.323605515 | 2.416439634 | 0.028521224 | 0.346585761 | -3.410278078 |
| CABLES1 | -0.746818544 | 2.664724067 | -2.416150705 | 0.02853748 | 0.346662011 | -3.410743522 |
| GPR171 | -0.511947679 | 10.03183516 | -2.415368262 | 0.028581548 | 0.346968628 | -3.412003873 |
| lnc-DPP4-1 | 0.601179577 | 4.084196322 | 2.415209659 | 0.028590488 | 0.346968628 | -3.41225933 |
| ARL14EP | -0.384170357 | 8.181005157 | -2.415044032 | 0.028599827 | 0.346968628 | -3.412526094 |
| CYP4F22 | -0.869701154 | 2.056604972 | -2.414949343 | 0.028605168 | 0.346968628 | -3.4126786 |
| CEBPB | 0.589783099 | 12.35542315 | 2.414647362 | 0.028622206 | 0.346968628 | -3.413164955 |
| OCLM | 1.068586431 | 1.730197101 | 2.414476379 | 0.028631857 | 0.346968628 | -3.413440321 |
| BCKDHA | -0.19438554 | 8.110855462 | -2.414453257 | 0.028633163 | 0.346968628 | -3.413477558 |
| UBE2T | -0.662043937 | 6.087467723 | -2.414285332 | 0.028642645 | 0.346968628 | -3.413747993 |
| CSF2RB | 0.842210221 | 6.666994989 | 2.413933541 | 0.028662519 | 0.347088313 | -3.414314507 |
| TBX21 | -0.695828734 | 10.45701384 | -2.41372089 | 0.028674539 | 0.34709531 | -3.414656941 |
| RASAL3 | -0.334425319 | 12.12106093 | -2.413569744 | 0.028683085 | 0.34709531 | -3.414900325 |
| lnc-GTDC1-5 | -0.763114687 | 1.742764001 | -2.413391508 | 0.028693166 | 0.347096362 | -3.415187324 |
| BAZ2B | 0.388012443 | 7.376923542 | 2.412919815 | 0.028719861 | 0.347298323 | -3.415946816 |
| TYSND1 | -0.502992087 | 5.360943224 | -2.412571617 | 0.028739582 | 0.347306467 | -3.416507425 |
| DAZAP1 | -0.293100595 | 8.925222917 | -2.412501624 | 0.028743548 | 0.347306467 | -3.416620114 |
| LINC01281 | -1.076987777 | 2.488037273 | -2.412240883 | 0.028758326 | 0.347306467 | -3.417039891 |
| KIAA0513 | 0.356639502 | 10.81098932 | 2.412201916 | 0.028760535 | 0.347306467 | -3.417102625 |
| ZNF197 | -0.457362522 | 4.554451176 | -2.411666 | 0.028790935 | 0.347385947 | -3.417965359 |
| ZBED5-AS1 | -0.236610077 | 7.708994524 | -2.411597575 | 0.028794818 | 0.347385947 | -3.418075506 |
| SHPRH | -0.37271047 | 7.505387544 | -2.411556936 | 0.028797125 | 0.347385947 | -3.418140923 |
| lnc-TP53I13-2 | -0.852970208 | 2.055300455 | -2.411203105 | 0.028817216 | 0.347507609 | -3.418710483 |
| CENPU | -0.599125207 | 4.72191006 | -2.411019039 | 0.028827673 | 0.347513045 | -3.41900676 |
| LINC00265 | 0.693436619 | 9.536559431 | 2.409662609 | 0.028904843 | 0.348271877 | -3.421189838 |
| IGJ | -1.276143926 | 9.959698467 | -2.409215136 | 0.028930344 | 0.348271877 | -3.421909913 |
| ITGA6 | -0.508214892 | 7.408421052 | -2.408892116 | 0.028948765 | 0.348271877 | -3.422429688 |
| MADCAM1 | 1.04704985 | 3.926471707 | 2.40880925 | 0.028953493 | 0.348271877 | -3.422563023 |
| CXCL16 | 0.627237304 | 7.507979393 | 2.408637149 | 0.028963314 | 0.348271877 | -3.422839937 |
| COL17A1 | 0.805835101 | 1.555219401 | 2.408456316 | 0.028973636 | 0.348271877 | -3.423130894 |
| MGC24103 | -1.033921371 | 5.046081163 | -2.408400941 | 0.028976798 | 0.348271877 | -3.423219988 |
| FAM155A-IT1 | 0.492907581 | 6.822324214 | 2.408379718 | 0.028978009 | 0.348271877 | -3.423254135 |
| lnc-C1orf21-3 | 0.744034195 | 1.101930088 | 2.408101731 | 0.028993887 | 0.348271877 | -3.423701389 |
| LOC101928000 | -0.681703326 | 6.786310362 | -2.408067228 | 0.028995858 | 0.348271877 | -3.423756899 |
| SERPINA1 | 0.66098568 | 12.6387403 | 2.407869011 | 0.029007185 | 0.348271877 | -3.424075796 |
| FAM90A7P | 0.750418162 | 0.983712617 | 2.407803015 | 0.029010957 | 0.348271877 | -3.424181971 |
| UBA2 | -0.501650162 | 7.95108656 | -2.406784512 | 0.029069232 | 0.348850871 | -3.425820398 |
| FBXO46 | -0.217055357 | 8.605161741 | -2.406320811 | 0.0290958 | 0.348972579 | -3.426566249 |
| UBA5 | -0.506307142 | 6.828338294 | -2.406012333 | 0.029113487 | 0.348972579 | -3.427062397 |
| RAPGEFL1 | 0.673883374 | 2.182998627 | 2.405897937 | 0.029120048 | 0.348972579 | -3.427246383 |
| ID3 | -0.444231841 | 6.909940172 | -2.405591252 | 0.029137646 | 0.348972579 | -3.427739615 |
| ASAH1 | 0.308722644 | 12.21838604 | 2.405568806 | 0.029138935 | 0.348972579 | -3.427775713 |
| RAP1GDS1 | -0.350934216 | 7.29217523 | -2.405556124 | 0.029139663 | 0.348972579 | -3.427796109 |
| LOC101929174 | -0.893908025 | 2.64552364 | -2.405363207 | 0.029150738 | 0.348984881 | -3.428106358 |
| COX15 | -0.267931821 | 4.617690281 | -2.404707188 | 0.029188431 | 0.349162144 | -3.429161294 |
| LOC90784 | -0.276011308 | 5.997245655 | -2.404218482 | 0.029216541 | 0.349162144 | -3.429947106 |
| LRFN3 | -0.625944229 | 7.416614235 | -2.40420945 | 0.029217061 | 0.349162144 | -3.429961629 |
| CYP17A1-AS1 | 0.51604725 | 6.271312558 | 2.404050173 | 0.029226228 | 0.349162144 | -3.430217724 |
| lnc-ITGA2-1 | -0.636907002 | 1.265704814 | -2.404014562 | 0.029228278 | 0.349162144 | -3.430274981 |
| ZNF491 | -0.398858586 | 0.978433539 | -2.403699963 | 0.029246393 | 0.349162144 | -3.430780791 |
| LINC01337 | -0.478150857 | 1.010772986 | -2.403605027 | 0.029251862 | 0.349162144 | -3.430933422 |
| TRMT112 | -0.462783228 | 11.85171091 | -2.4036047 | 0.029251881 | 0.349162144 | -3.430933948 |
| lnc-STRA8-1 | 0.656554097 | 2.187639562 | 2.403532725 | 0.029256028 | 0.349162144 | -3.431049664 |
| COX8A | -0.250347684 | 12.42588929 | -2.403279022 | 0.029270649 | 0.349216639 | -3.431457536 |
| F2R | -0.639886227 | 5.938888741 | -2.403081777 | 0.029282021 | 0.349232349 | -3.431774631 |
| SERBP1 | -0.349710193 | 9.54210489 | -2.402842793 | 0.029295806 | 0.349276587 | -3.432158812 |
| DSTN | -0.424419207 | 10.19498742 | -2.402668817 | 0.029305844 | 0.349276587 | -3.432438481 |
| COL24A1 | -0.734728502 | 3.204885362 | -2.402055081 | 0.029341284 | 0.349312123 | -3.433425006 |
| YES1 | -0.67334728 | 4.601986136 | -2.401939227 | 0.029347978 | 0.349312123 | -3.43361122 |
| NEIL2 | -0.74460847 | 3.014078816 | -2.40189638 | 0.029350454 | 0.349312123 | -3.433680089 |
| DDX20 | -0.362558551 | 7.817197047 | -2.401876848 | 0.029351583 | 0.349312123 | -3.433711482 |
| lnc-RPP38-2 | 0.822990294 | 6.30944941 | 2.401666605 | 0.029363737 | 0.349312123 | -3.434049398 |
| RAB3IP | 0.316692221 | 8.075802681 | 2.401285674 | 0.029385769 | 0.349312123 | -3.434661624 |
| FLJ42351 | -0.324882574 | 6.628872523 | -2.401227418 | 0.02938914 | 0.349312123 | -3.43475525 |
| FAIM3 | -0.569417836 | 7.825164759 | -2.401026079 | 0.029400793 | 0.349312123 | -3.435078819 |
| TBX5-AS1 | 0.693560538 | 1.433886804 | 2.4009742 | 0.029403796 | 0.349312123 | -3.435162192 |
| lnc-C3orf23-2 | -0.414033166 | 0.949226415 | -2.400877323 | 0.029409405 | 0.349312123 | -3.435317879 |
| LACE1 | -0.626217143 | 3.9896336 | -2.400381125 | 0.02943815 | 0.349534002 | -3.436115253 |
| ABCE1 | -0.484306542 | 7.049171109 | -2.399679823 | 0.029478822 | 0.349693832 | -3.437242118 |
| LOC102724953 | -0.512776187 | 1.473030012 | -2.399678565 | 0.029478895 | 0.349693832 | -3.437244139 |
| C12orf57 | -0.621294478 | 11.30921363 | -2.399628199 | 0.029481818 | 0.349693832 | -3.437325063 |
| SPAG5 | -0.615421254 | 5.816362247 | -2.399389334 | 0.029495684 | 0.349738859 | -3.437708842 |
| MAML3 | 0.642774861 | 5.891516955 | 2.39915376 | 0.029509366 | 0.349753505 | -3.438087322 |
| POLD1 | -0.232464419 | 7.663311243 | -2.39873201 | 0.029533874 | 0.349753505 | -3.438764879 |
| CLTCL1 | 0.41609199 | 3.870141782 | 2.3987156 | 0.029534829 | 0.349753505 | -3.438791242 |
| SPRY2 | -0.808447372 | 3.657652721 | -2.398674773 | 0.029537202 | 0.349753505 | -3.438856829 |
| C4orf33 | 0.393507927 | 7.636349493 | 2.398496024 | 0.029547597 | 0.349757337 | -3.43914398 |
| LINC01153 | 0.938445385 | 4.26352416 | 2.398113977 | 0.029569824 | 0.349811883 | -3.439757691 |
| FBXO41 | -0.364688035 | 8.635623952 | -2.398001978 | 0.029576344 | 0.349811883 | -3.439937597 |
| LOC730257 | -0.350633019 | 9.491070981 | -2.397897582 | 0.029582422 | 0.349811883 | -3.440105287 |
| lnc-NTHL1-1 | 1.075861699 | 1.697267064 | 2.39763866 | 0.029597501 | 0.349827483 | -3.440521178 |
| SPATC1L | -0.64038282 | 4.135606462 | -2.397529057 | 0.029603886 | 0.349827483 | -3.44069722 |
| OGFRL1 | 0.409769592 | 8.782162427 | 2.39722598 | 0.02962155 | 0.34987009 | -3.441184004 |
| MAN2A1 | -0.394535823 | 5.814648886 | -2.39712153 | 0.02962764 | 0.34987009 | -3.44135176 |
| UXT | -0.322355745 | 10.64543969 | -2.396558513 | 0.029660486 | 0.350091425 | -3.442255965 |
| MRPS27 | -0.43088932 | 7.675247409 | -2.396010715 | 0.029692478 | 0.350091425 | -3.443135651 |
| ELAVL4 | 0.634583287 | 1.226614298 | 2.395900611 | 0.029698912 | 0.350091425 | -3.443312454 |
| RAI1 | -0.395847141 | 6.302811591 | -2.395464354 | 0.029724418 | 0.350091425 | -3.444012953 |
| ECEL1P2 | 0.656014194 | 6.39769402 | 2.395115895 | 0.029744805 | 0.350091425 | -3.44457244 |
| ISOC1 | -0.531470709 | 7.32079981 | -2.394995495 | 0.029751853 | 0.350091425 | -3.444765747 |
| CHIAP2 | 0.681412695 | 1.385352002 | 2.394953945 | 0.029754285 | 0.350091425 | -3.444832457 |
| LOC101927588 | -0.475186159 | 1.377428793 | -2.394944044 | 0.029754865 | 0.350091425 | -3.444848354 |
| SDCCAG3 | -0.321536015 | 8.141229722 | -2.394797714 | 0.029763433 | 0.350091425 | -3.445083286 |
| C7orf34 | 1.099744911 | 4.614327644 | 2.394635346 | 0.029772943 | 0.350091425 | -3.445343961 |
| SNRPN | -0.320809651 | 6.829690055 | -2.394340115 | 0.029790242 | 0.350091425 | -3.445817923 |
| TMEM198B | -0.230339715 | 6.538979161 | -2.394305317 | 0.029792282 | 0.350091425 | -3.445873786 |
| SMAD1-AS2 | -0.367422365 | 0.821034896 | -2.394261006 | 0.029794879 | 0.350091425 | -3.445944921 |
| RNF24 | 0.712680207 | 10.44043627 | 2.393910957 | 0.029815406 | 0.350091425 | -3.446506853 |
| lnc-SPAG1-3 | -0.894334007 | 2.751092429 | -2.39391076 | 0.029815417 | 0.350091425 | -3.446507169 |
| ALKBH7 | -0.289773009 | 9.211557172 | -2.393842087 | 0.029819446 | 0.350091425 | -3.446617405 |
| CYSTM1 | 0.892549319 | 9.849255695 | 2.393601359 | 0.029833571 | 0.350091425 | -3.447003822 |
| lnc-CHTF8-2 | 0.402091024 | 1.000516808 | 2.393585382 | 0.029834509 | 0.350091425 | -3.447029468 |
| AASDHPPT | -0.34168287 | 8.317013259 | -2.393527437 | 0.02983791 | 0.350091425 | -3.447122479 |
| OSBP | -0.279689605 | 6.861182823 | -2.39319525 | 0.029857415 | 0.350201972 | -3.447655675 |
| ZFP3 | -0.270911676 | 6.662620488 | -2.392744522 | 0.029883901 | 0.350281635 | -3.448379096 |
| lnc-PGPEP1L-1 | -0.54453032 | 1.502943955 | -2.392728636 | 0.029884834 | 0.350281635 | -3.448404592 |
| TMEM203 | -0.292226668 | 8.278686703 | -2.392386276 | 0.029904967 | 0.350281635 | -3.448954047 |
| LOC102724851 | -0.686205916 | 5.169872255 | -2.392369656 | 0.029905945 | 0.350281635 | -3.448980719 |
| DKC1 | -0.334705411 | 8.62207661 | -2.391995091 | 0.029927987 | 0.350281635 | -3.449581822 |
| KLRF1 | -0.903805886 | 8.574823741 | -2.391882312 | 0.029934626 | 0.350281635 | -3.449762803 |
| OR2B2 | 1.013901328 | 1.776958118 | 2.391879224 | 0.029934808 | 0.350281635 | -3.449767759 |
| MRGBP | -0.411919228 | 6.143076737 | -2.391619701 | 0.029950093 | 0.350324388 | -3.450184214 |
| ENDOV | 0.484914195 | 4.760298769 | 2.391474691 | 0.029958636 | 0.350324388 | -3.450416902 |
| CNTLN | 0.538489646 | 3.647982335 | 2.390924936 | 0.029991046 | 0.35038194 | -3.451299012 |
| TRAF5 | -0.488508863 | 9.230043349 | -2.390469207 | 0.030017939 | 0.35038194 | -3.452030192 |
| lnc-AMDHD1-2 | 0.858674994 | 2.83554431 | 2.390411376 | 0.030021353 | 0.35038194 | -3.452122975 |
| RHOF | -0.362922308 | 5.60576621 | -2.390234156 | 0.030031818 | 0.35038194 | -3.452407293 |
| WDR12 | -0.587426953 | 6.144256418 | -2.390215258 | 0.030032934 | 0.35038194 | -3.45243761 |
| GCSH | -0.336648022 | 7.519344614 | -2.390011973 | 0.030044943 | 0.35038194 | -3.452763736 |
| FTL | 0.396720976 | 12.71152258 | 2.389764367 | 0.030059576 | 0.35038194 | -3.453160947 |
| TRAF3IP1 | -0.455875831 | 5.973265146 | -2.38964573 | 0.030066589 | 0.35038194 | -3.453351261 |
| LOC100288884 | 0.921031214 | 3.877443161 | 2.389539234 | 0.030072886 | 0.35038194 | -3.453522095 |
| FTSJ1 | -0.398773382 | 9.369319957 | -2.389369026 | 0.030082953 | 0.35038194 | -3.453795125 |
| lnc-RP11-582J16.5.1-4 | -0.727944227 | 2.913600431 | -2.389355279 | 0.030083767 | 0.35038194 | -3.453817176 |
| TBC1D8 | 0.410112431 | 6.004375465 | 2.389340809 | 0.030084623 | 0.35038194 | -3.453840388 |
| ZNF444 | -0.251938935 | 9.630983283 | -2.388874593 | 0.030112215 | 0.350419734 | -3.454588202 |
| USP12 | -0.364107813 | 3.432854707 | -2.388793572 | 0.030117013 | 0.350419734 | -3.454718156 |
| TULP4 | -0.479527375 | 6.903918773 | -2.388549615 | 0.030131462 | 0.350419734 | -3.455109437 |
| lnc-C17orf62-3 | -0.765952771 | 3.647668594 | -2.388518884 | 0.030133283 | 0.350419734 | -3.455158726 |
| MEF2BNB | 0.593673967 | 7.536057994 | 2.388369318 | 0.030142146 | 0.350419734 | -3.455398606 |
| PTPRA | -0.262473431 | 9.309509029 | -2.388263688 | 0.030148407 | 0.350419734 | -3.455568016 |
| HNRNPA0 | -0.434564665 | 6.377874336 | -2.387850698 | 0.030172897 | 0.350587053 | -3.456230343 |
| DEPDC1 | -0.57334917 | 4.518592846 | -2.387268932 | 0.030207427 | 0.350782824 | -3.457163268 |
| OLFM4 | 2.496978592 | 5.43042902 | 2.387226509 | 0.030209946 | 0.350782824 | -3.457231295 |
| PLXNA1 | -0.429731604 | 7.402894894 | -2.386791949 | 0.030235765 | 0.350907568 | -3.457928097 |
| lnc-C1orf106-1 | 0.948520658 | 3.056239113 | 2.386553027 | 0.03024997 | 0.350907568 | -3.45831118 |
| MDH2 | -0.293478564 | 9.802941174 | -2.38647833 | 0.030254412 | 0.350907568 | -3.458430944 |
| lnc-KIF13B-2 | -0.769469266 | 1.591677048 | -2.386365803 | 0.030261105 | 0.350907568 | -3.45861136 |
| KIRREL3-AS2 | -0.54584106 | 1.373030067 | -2.386029532 | 0.030281114 | 0.350920708 | -3.459150489 |
| XKR5 | 0.733994887 | 3.780128181 | 2.38585626 | 0.030291429 | 0.350920708 | -3.459428276 |
| APRT | -0.354499678 | 11.76689088 | -2.385837431 | 0.030292551 | 0.350920708 | -3.459458463 |
| SNORA49 | 0.323367604 | 4.942374817 | 2.385496597 | 0.030312852 | 0.351038791 | -3.460004859 |
| GTF2H3 | -0.404748985 | 4.248434747 | -2.385049143 | 0.030339523 | 0.351096436 | -3.460722136 |
| MAPKAP1 | 0.258774494 | 5.487321946 | 2.384860626 | 0.030350766 | 0.351096436 | -3.461024316 |
| SDHA | -0.313093584 | 11.34961204 | -2.384736123 | 0.030358194 | 0.351096436 | -3.46122388 |
| LILRB3 | 0.556208916 | 14.21445663 | 2.384629118 | 0.030364579 | 0.351096436 | -3.461395395 |
| TP73 | 0.71892559 | 2.312926156 | 2.384565496 | 0.030368376 | 0.351096436 | -3.461497371 |
| ZC3H8 | -0.581809816 | 0.926216615 | -2.38391279 | 0.030407356 | 0.351353211 | -3.462543492 |
| INPP5A | 0.348154453 | 6.981739606 | 2.383854834 | 0.030410819 | 0.351353211 | -3.462636377 |
| lnc-CST9LP1-2 | -0.567027438 | 1.141906918 | -2.383645834 | 0.030423312 | 0.351380655 | -3.462971323 |
| TXK | -0.584463006 | 7.776289355 | -2.383411624 | 0.030437318 | 0.351402832 | -3.46334666 |
| LINC01000 | 0.536761511 | 10.27337948 | 2.383259449 | 0.030446421 | 0.351402832 | -3.463590521 |
| DLGAP5 | -1.066628618 | 3.375426812 | -2.383106273 | 0.030455587 | 0.351402832 | -3.463835982 |
| USP54 | -0.339906425 | 5.494781215 | -2.382897738 | 0.030468069 | 0.351430102 | -3.464170143 |
| STK32B | -0.583053882 | 1.118268634 | -2.382647638 | 0.030483046 | 0.351486116 | -3.464570895 |
| VWA5B1 | 0.479305304 | 2.934291114 | 2.382322346 | 0.030502536 | 0.351594114 | -3.465092107 |
| ATAD3B | -0.212328176 | 9.480580894 | -2.38170358 | 0.030539642 | 0.351814183 | -3.466083472 |
| CAND1 | -0.284142608 | 6.999025339 | -2.381666145 | 0.030541888 | 0.351814183 | -3.466143446 |
| RBL1 | -0.510145855 | 4.557061423 | -2.381269807 | 0.030565679 | 0.351971498 | -3.466778389 |
| MRPL45 | -0.452773038 | 9.183260718 | -2.380991359 | 0.030582404 | 0.351991832 | -3.467224445 |
| XLOC_l2_014217 | -0.844769864 | 2.287821147 | -2.380638743 | 0.030603597 | 0.351991832 | -3.467789284 |
| SNORA66 | -0.741706347 | 2.70495016 | -2.380379184 | 0.030619205 | 0.351991832 | -3.468205038 |
| SPIN1 | -0.462403433 | 6.387363123 | -2.38036019 | 0.030620348 | 0.351991832 | -3.468235462 |
| JUNB | 0.666839296 | 6.233958614 | 2.380333433 | 0.030621957 | 0.351991832 | -3.46827832 |
| RSPO3 | 0.892270114 | 1.978750475 | 2.380142336 | 0.030633455 | 0.351991832 | -3.468584401 |
| lnc-SHISA9-4 | -0.871167141 | 2.349530034 | -2.379984289 | 0.030642967 | 0.351991832 | -3.468837537 |
| ZNF121 | -0.929505828 | 2.909145428 | -2.379727821 | 0.030658408 | 0.351991832 | -3.469248296 |
| lnc-UTS2R-1 | 0.72560623 | 2.811938526 | 2.379586217 | 0.030666937 | 0.351991832 | -3.469475083 |
| BTN2A1 | 0.429073338 | 8.316059939 | 2.379555355 | 0.030668796 | 0.351991832 | -3.469524509 |
| SLC25A3 | -0.319140476 | 12.73755583 | -2.37913219 | 0.030694298 | 0.352067648 | -3.470202194 |
| PABPC1L | 0.370102951 | 6.471158909 | 2.379109328 | 0.030695676 | 0.352067648 | -3.470238805 |
| UTP3 | -0.461626534 | 9.464696495 | -2.378683785 | 0.030721343 | 0.352245704 | -3.47092025 |
| lnc-C6orf221-2 | -0.74562857 | 3.10013985 | -2.378438409 | 0.030736152 | 0.352299192 | -3.471313162 |
| CHAF1A | -0.246967395 | 7.94063441 | -2.377996142 | 0.030762861 | 0.352488996 | -3.472021306 |
| MTMR6 | 0.277485256 | 6.552495599 | 2.377795361 | 0.030774993 | 0.352511712 | -3.472342775 |
| lnc-PABPN1L-1 | 0.575924528 | 5.693494407 | 2.377433154 | 0.030796892 | 0.352646239 | -3.472922674 |
| LINC00526 | -0.717238317 | 3.776731738 | -2.377101732 | 0.030816942 | 0.352759519 | -3.473453255 |
| LOC101928858 | 0.540642479 | 2.031304655 | 2.376470455 | 0.030855166 | 0.352968823 | -3.474463802 |
| ACSS1 | -0.35315565 | 9.098635808 | -2.376464072 | 0.030855553 | 0.352968823 | -3.474474019 |
| lnc-PIGF-3 | -0.504383358 | 1.242025821 | -2.375957675 | 0.030886248 | 0.353095308 | -3.475284582 |
| FOXA3 | 0.67262832 | 2.931567402 | 2.375821815 | 0.030894488 | 0.353095308 | -3.475502034 |
| NET1 | -0.667480491 | 5.62704005 | -2.375778591 | 0.03089711 | 0.353095308 | -3.475571215 |
| lnc-MYOM2-2 | -1.991918312 | 3.252572972 | -2.375470524 | 0.030915804 | 0.353108163 | -3.476064274 |
| TMEM258 | -0.381741974 | 11.15964693 | -2.375424967 | 0.03091857 | 0.353108163 | -3.476137186 |
| lnc-SLC4A1AP-1 | 1.592899203 | 6.693104094 | 2.375077967 | 0.030939641 | 0.353232648 | -3.476692521 |
| L3MBTL1 | -0.417164649 | 6.287076272 | -2.374675996 | 0.030964066 | 0.353295887 | -3.477335794 |
| HSF4 | 0.525638415 | 3.652601598 | 2.374652003 | 0.030965525 | 0.353295887 | -3.477374188 |
| COG8 | -0.238157072 | 7.73842146 | -2.374403361 | 0.030980644 | 0.353299122 | -3.477772065 |
| CDKN2A-AS1 | 0.569528044 | 3.433902261 | 2.374120557 | 0.030997848 | 0.353299122 | -3.478224591 |
| TUBGCP4 | -0.663193599 | 4.142804968 | -2.374112288 | 0.030998351 | 0.353299122 | -3.478237821 |
| RASA3 | -0.440529971 | 11.66171781 | -2.373978397 | 0.031006499 | 0.353299122 | -3.478452057 |
| TAB1 | -0.619163127 | 5.077154093 | -2.373521338 | 0.031034331 | 0.353390605 | -3.47918335 |
| EBF4 | -0.554110777 | 4.004772368 | -2.373387844 | 0.031042464 | 0.353390605 | -3.479396931 |
| ACY1 | -0.301722398 | 6.294462017 | -2.373085076 | 0.031060918 | 0.353390605 | -3.479881317 |
| FAM111A | 0.192157508 | 6.588833074 | 2.37302086 | 0.031064833 | 0.353390605 | -3.479984051 |
| CIT | -0.7068088 | 3.937746632 | -2.372756495 | 0.031080956 | 0.353390605 | -3.480406974 |
| GNPNAT1 | -0.551399516 | 6.53262847 | -2.372512426 | 0.031095849 | 0.353390605 | -3.480797411 |
| HOPX | -0.743604825 | 9.496656079 | -2.372357515 | 0.031105305 | 0.353390605 | -3.481045216 |
| UBE2D1 | 0.732919915 | 9.204692303 | 2.372355327 | 0.031105438 | 0.353390605 | -3.481048716 |
| LINC00313 | 0.505250674 | 2.261047045 | 2.372270639 | 0.031110609 | 0.353390605 | -3.481184184 |
| ELOVL5 | 0.24255373 | 8.600834259 | 2.37202703 | 0.031125487 | 0.353390605 | -3.481573855 |
| ECI2 | -0.353028767 | 8.891525449 | -2.372011145 | 0.031126457 | 0.353390605 | -3.481599264 |
| TTC34 | -0.608495012 | 4.766068419 | -2.371329676 | 0.031168113 | 0.353677779 | -3.482689239 |
| SNORA62 | -0.483230102 | 4.658894629 | -2.371211688 | 0.031175331 | 0.353677779 | -3.482877942 |
| DAPK2 | 0.785715807 | 7.526776578 | 2.371097749 | 0.031182302 | 0.353677779 | -3.483060165 |
| C10orf2 | -0.621156358 | 3.874243535 | -2.370352768 | 0.03122792 | 0.354079554 | -3.484251538 |
| LINC-PINT | 0.456307121 | 8.854113122 | 2.369986039 | 0.0312504 | 0.35408737 | -3.484837956 |
| FUT1 | 0.553496682 | 1.62109588 | 2.369212115 | 0.031297889 | 0.35408737 | -3.486075384 |
| TARDBP | -0.35332458 | 11.20803286 | -2.36908365 | 0.031305778 | 0.35408737 | -3.48628077 |
| SETD6 | -0.417672857 | 6.812227317 | -2.369019119 | 0.031309742 | 0.35408737 | -3.48638394 |
| GDPGP1 | -0.23269049 | 5.065681689 | -2.36891223 | 0.031316308 | 0.35408737 | -3.486554827 |
| CCDC85B | -0.301575647 | 8.163429807 | -2.368843848 | 0.03132051 | 0.35408737 | -3.486664149 |
| HSD17B10 | -0.368440098 | 10.09715466 | -2.368805574 | 0.031322862 | 0.35408737 | -3.486725338 |
| IFRD1 | 0.55661027 | 7.153227958 | 2.368673859 | 0.031330957 | 0.35408737 | -3.486935907 |
| ADAMTS3 | -0.347418853 | 1.07460554 | -2.368625744 | 0.031333915 | 0.35408737 | -3.487012827 |
| OR6M1 | 0.505906507 | 2.598379916 | 2.368548135 | 0.031338686 | 0.35408737 | -3.487136894 |
| LOC102724958 | -0.438148435 | 1.080593208 | -2.368430107 | 0.031345943 | 0.35408737 | -3.487325574 |
| TMEM88 | 1.004850405 | 7.226440928 | 2.368087554 | 0.031367015 | 0.35408737 | -3.48787316 |
| lnc-ITSN1-2 | -0.67893987 | 5.606365252 | -2.367630157 | 0.031395172 | 0.35408737 | -3.488604281 |
| MTSS1 | -0.440390318 | 7.560905556 | -2.367617347 | 0.031395961 | 0.35408737 | -3.488624756 |
| VCP | -0.22876827 | 11.84330316 | -2.367420224 | 0.031408103 | 0.35408737 | -3.488939828 |
| SLC25A32 | -0.44109421 | 8.076308356 | -2.367221624 | 0.031420341 | 0.35408737 | -3.489257249 |
| RBM26-AS1 | -0.549916092 | 4.410746681 | -2.366984984 | 0.031434929 | 0.35408737 | -3.489635456 |
| DMPK | -0.308928283 | 6.885219662 | -2.366708464 | 0.031451983 | 0.35408737 | -3.490077381 |
| TRAPPC2 | -0.222085327 | 7.529636647 | -2.366610848 | 0.031458005 | 0.35408737 | -3.490233383 |
| CCDC115 | -0.365694806 | 9.677553547 | -2.366572364 | 0.03146038 | 0.35408737 | -3.490294885 |
| PMM1 | -0.292951058 | 6.5854199 | -2.366528413 | 0.031463092 | 0.35408737 | -3.490365122 |
| MLST8 | -0.223431201 | 6.973411507 | -2.366398858 | 0.031471088 | 0.35408737 | -3.490572161 |
| CSTF2T | -0.28360471 | 7.045584106 | -2.366290082 | 0.031477803 | 0.35408737 | -3.490745988 |
| LY6G6C | 0.874908349 | 3.003193402 | 2.36612227 | 0.031488165 | 0.35408737 | -3.491014151 |
| lnc-OR51B4-3 | 0.864085378 | 2.385913851 | 2.365934045 | 0.031499791 | 0.35408737 | -3.491314925 |
| E2F7 | -0.934853186 | 3.478256312 | -2.365847382 | 0.031505145 | 0.35408737 | -3.491453406 |
| TSR2 | -0.292766625 | 8.699020683 | -2.365722809 | 0.031512843 | 0.35408737 | -3.491652459 |
| QRICH1 | -0.283182094 | 8.389362565 | -2.365661412 | 0.031516638 | 0.35408737 | -3.491750563 |
| lnc-POLR1E-1 | -0.727001857 | 1.737183348 | -2.365537836 | 0.031524277 | 0.35408737 | -3.491948018 |
| COL15A1 | -0.47360122 | 0.980276777 | -2.365086195 | 0.031552211 | 0.354135722 | -3.492669631 |
| PSMD1 | -0.244066449 | 10.18652931 | -2.365035579 | 0.031555343 | 0.354135722 | -3.492750501 |
| MPV17 | -0.255439991 | 9.94300951 | -2.364814718 | 0.031569013 | 0.354135722 | -3.49310336 |
| RNFT2 | -0.471332283 | 4.122614858 | -2.364644223 | 0.031579569 | 0.354135722 | -3.493375743 |
| lnc-SALL3-1 | 0.961763021 | 1.619146587 | 2.364347673 | 0.031597939 | 0.354135722 | -3.493849496 |
| PYGM | 0.501685958 | 4.494594171 | 2.364331267 | 0.031598955 | 0.354135722 | -3.493875704 |
| LOC100506974 | -0.644265414 | 5.49633988 | -2.364315053 | 0.03159996 | 0.354135722 | -3.493901605 |
| lnc-GBE1-6 | -0.695290986 | 1.461090853 | -2.364069957 | 0.03161515 | 0.354191666 | -3.494293136 |
| IL32 | -0.572978318 | 10.40428762 | -2.363706766 | 0.031637673 | 0.354315654 | -3.494873291 |
| LOC374443 | -0.396836797 | 7.741470579 | -2.363060501 | 0.031677786 | 0.354315654 | -3.495905535 |
| PGA3 | -0.87116334 | 3.459545667 | -2.363013997 | 0.031680675 | 0.354315654 | -3.495979809 |
| NECAP2 | -0.287323422 | 10.63708777 | -2.362857558 | 0.031690393 | 0.354315654 | -3.496229661 |
| GPR124 | -0.586350091 | 4.609087159 | -2.362806638 | 0.031693557 | 0.354315654 | -3.496310987 |
| lnc-C10orf131-1 | 0.935101535 | 2.735525423 | 2.362804869 | 0.031693667 | 0.354315654 | -3.496313811 |
| lnc-TFDP2-2 | -0.608273368 | 1.131385119 | -2.362741068 | 0.031697631 | 0.354315654 | -3.496415706 |
| RECQL4 | -0.512460931 | 6.363829635 | -2.36165877 | 0.031764957 | 0.35479127 | -3.49814406 |
| PLA2G16 | -0.680811667 | 9.390950212 | -2.361631601 | 0.031766649 | 0.35479127 | -3.498187443 |
| NDRG2 | -0.443515254 | 8.758165501 | -2.361564494 | 0.031770828 | 0.35479127 | -3.498294598 |
| FGFBP2 | -0.886603792 | 7.688927646 | -2.361288929 | 0.031787994 | 0.354868864 | -3.498734597 |
| IPP | -0.491562739 | 6.275253805 | -2.360932576 | 0.031810206 | 0.355002717 | -3.499303564 |
| SFMBT2 | -0.498983527 | 4.074847325 | -2.360096057 | 0.031862405 | 0.355279781 | -3.500639048 |
| CTSG | 1.733010388 | 5.994036113 | 2.360011486 | 0.031867686 | 0.355279781 | -3.500774053 |
| lnc-MAN2C1-4 | 0.485169289 | 3.766187817 | 2.359756505 | 0.031883616 | 0.355279781 | -3.501181082 |
| LOC100288069 | 0.760020189 | 9.788103218 | 2.359728614 | 0.031885359 | 0.355279781 | -3.501225604 |
| ATG2A | 0.413803438 | 10.82409417 | 2.359677528 | 0.031888551 | 0.355279781 | -3.501307149 |
| SSSCA1 | -0.325233522 | 7.965258477 | -2.359428481 | 0.031904119 | 0.355279781 | -3.501704683 |
| HEG1 | -0.544819443 | 7.665468559 | -2.359388154 | 0.031906641 | 0.355279781 | -3.501769052 |
| CBFB | -0.321952734 | 8.466749969 | -2.359025955 | 0.031929297 | 0.355344321 | -3.502347168 |
| CREBRF | 0.368844615 | 10.64293321 | 2.358554956 | 0.031958781 | 0.355344321 | -3.50309889 |
| LARS2 | -0.394537362 | 6.019874196 | -2.358548499 | 0.031959185 | 0.355344321 | -3.503109193 |
| MAP4K1 | -0.35035807 | 7.707235128 | -2.358510297 | 0.031961578 | 0.355344321 | -3.503170162 |
| lnc-BBOX1-1 | -0.335054565 | 0.944849664 | -2.358377848 | 0.031969874 | 0.355344321 | -3.50338154 |
| SNAP47 | -0.272443625 | 8.945487533 | -2.358263063 | 0.031977066 | 0.355344321 | -3.503564724 |
| ALKBH8 | -0.448894717 | 6.069105735 | -2.358127065 | 0.031985588 | 0.355344321 | -3.503781757 |
| lnc-PRDM9-2 | 0.384889629 | 1.066265204 | 2.357903915 | 0.031999577 | 0.355344321 | -3.504137862 |
| ANKRD30BL | 0.731520576 | 2.729574424 | 2.357646216 | 0.032015739 | 0.355344321 | -3.504549083 |
| lnc-PRDM13-4 | 0.816793392 | 2.325538294 | 2.357591842 | 0.03201915 | 0.355344321 | -3.504635847 |
| SLC7A11 | -0.973132902 | 3.196539829 | -2.357356784 | 0.0320339 | 0.355344321 | -3.505010919 |
| DLG3 | -0.415244997 | 6.29924555 | -2.357335812 | 0.032035217 | 0.355344321 | -3.505044383 |
| COG2 | -0.340351902 | 8.250849288 | -2.35716026 | 0.032046237 | 0.35535307 | -3.505324494 |
| H2AFB3 | 0.505658151 | 3.421079817 | 2.356808731 | 0.032068316 | 0.355370223 | -3.505885368 |
| DOCK10 | -0.536694181 | 9.789622012 | -2.356786201 | 0.032069731 | 0.355370223 | -3.505921316 |
| ABCA3 | -0.671741433 | 4.285449752 | -2.356646961 | 0.032078481 | 0.355370223 | -3.506143466 |
| MTAP | -0.425639188 | 6.810657569 | -2.356135881 | 0.032110616 | 0.355612784 | -3.506958827 |
| ABCA7 | 0.52960385 | 9.050343943 | 2.355710503 | 0.032137385 | 0.355795789 | -3.507637409 |
| MIIP | 0.210973515 | 11.2330048 | 2.355292004 | 0.032163742 | 0.355797792 | -3.508304968 |
| PATL1 | 0.418673739 | 6.88747051 | 2.355077505 | 0.032177259 | 0.355797792 | -3.508647103 |
| CIAO1 | -0.185141468 | 8.849584819 | -2.354808958 | 0.03219419 | 0.355797792 | -3.509075431 |
| MORC4 | -0.585551023 | 5.201435343 | -2.354687514 | 0.032201849 | 0.355797792 | -3.509269124 |
| TPD52L2 | 0.467897411 | 5.660623775 | 2.354445437 | 0.032217121 | 0.355797792 | -3.509655207 |
| DLGAP1-AS3 | -0.845481477 | 3.271256698 | -2.354299828 | 0.03222631 | 0.355797792 | -3.509887427 |
| IGFL1 | -0.586344475 | 1.330854898 | -2.354293117 | 0.032226734 | 0.355797792 | -3.50989813 |
| PHACTR4 | -0.419745114 | 6.778160262 | -2.353323355 | 0.032288 | 0.355797792 | -3.511444582 |
| lnc-KIAA0226L-4 | -0.800762718 | 3.763183222 | -2.353271663 | 0.032291269 | 0.355797792 | -3.511527008 |
| DNAJC21 | -0.265133387 | 6.679641 | -2.353089526 | 0.032302789 | 0.355797792 | -3.511817426 |
| NADK2 | -0.341449742 | 4.896832529 | -2.353057494 | 0.032304815 | 0.355797792 | -3.511868499 |
| GLT1D1 | 0.707523794 | 10.60147442 | 2.352878674 | 0.03231613 | 0.355797792 | -3.512153617 |
| ATAD3A | -0.239685601 | 9.263414962 | -2.352642122 | 0.032331104 | 0.355797792 | -3.512530773 |
| TMED3 | -0.220556763 | 7.96749126 | -2.352544078 | 0.032337312 | 0.355797792 | -3.51268709 |
| LCE3B | -0.520204144 | 1.533769762 | -2.352527583 | 0.032338356 | 0.355797792 | -3.512713387 |
| CHD1L | -0.370455303 | 7.225958243 | -2.352518906 | 0.032338906 | 0.355797792 | -3.512727222 |
| MSRA | 0.281789404 | 8.575718399 | 2.352508662 | 0.032339554 | 0.355797792 | -3.512743554 |
| ATP2B1 | 0.251494592 | 6.768850337 | 2.35246157 | 0.032342537 | 0.355797792 | -3.512818634 |
| SLC39A14 | -0.56188839 | 5.665722656 | -2.352450791 | 0.032343219 | 0.355797792 | -3.512835818 |
| FAM102A | -0.659359289 | 11.49927622 | -2.352348954 | 0.03234967 | 0.355797792 | -3.512998176 |
| lnc-TMEM18-11 | 1.189931579 | 5.37294248 | 2.352208023 | 0.032358598 | 0.355797792 | -3.513222856 |
| DCP1A | -0.196819026 | 10.44991142 | -2.351993088 | 0.03237222 | 0.355797792 | -3.513565507 |
| PLSCR1 | 0.913538199 | 8.918589754 | 2.351977725 | 0.032373194 | 0.355797792 | -3.513589999 |
| LINC00482 | 0.492719188 | 7.171589812 | 2.351704318 | 0.03239053 | 0.355875701 | -3.514025848 |
| ASCC1 | -0.394752323 | 5.308478853 | -2.351063399 | 0.032431202 | 0.356086904 | -3.515047482 |
| URM1 | -0.215749177 | 10.17957267 | -2.350745941 | 0.032451365 | 0.356086904 | -3.515553474 |
| CITED2 | -0.269640284 | 8.778053196 | -2.350585752 | 0.032461544 | 0.356086904 | -3.515808787 |
| GATA3 | -0.410894246 | 7.405467287 | -2.350575774 | 0.032462178 | 0.356086904 | -3.515824689 |
| ZCCHC3 | -0.31847772 | 7.094372037 | -2.350120307 | 0.032491137 | 0.356086904 | -3.516550582 |
| DMXL2 | 0.43745631 | 4.748426815 | 2.350117298 | 0.032491329 | 0.356086904 | -3.516555377 |
| LINC01094 | 0.799809385 | 5.078975153 | 2.349963981 | 0.032501082 | 0.356086904 | -3.516799711 |
| SLC52A2 | -0.304381451 | 10.24897096 | -2.349951278 | 0.03250189 | 0.356086904 | -3.516819956 |
| IGSF9B | 0.284183859 | 6.20716692 | 2.349934381 | 0.032502965 | 0.356086904 | -3.516846883 |
| lnc-NME3-1 | 0.390504325 | 3.86572988 | 2.349694701 | 0.032518219 | 0.356086904 | -3.517228834 |
| STAT3 | 0.562559177 | 7.202110112 | 2.349626903 | 0.032522536 | 0.356086904 | -3.517336874 |
| ZNF696 | -0.268405812 | 6.251560201 | -2.349395456 | 0.032537274 | 0.356135997 | -3.517705686 |
| RPP25L | -0.215644787 | 10.73122613 | -2.348612551 | 0.032587175 | 0.356460689 | -3.518953141 |
| lnc-AL020996.1-1 | -0.531816973 | 4.112430671 | -2.348607986 | 0.032587466 | 0.356460689 | -3.518960415 |
| SLFN12L | -0.393777846 | 3.347991257 | -2.348390676 | 0.03260133 | 0.356500057 | -3.519306641 |
| OXLD1 | -0.406311014 | 10.69613538 | -2.347937812 | 0.032630239 | 0.356703872 | -3.520028116 |
| FSTL4 | -0.483369335 | 1.21706372 | -2.34721644 | 0.032676339 | 0.356881901 | -3.521177247 |
| FFAR3 | 1.229879578 | 5.620984448 | 2.347214348 | 0.032676473 | 0.356881901 | -3.52118058 |
| MANSC1 | 0.987552424 | 5.658957203 | 2.34708993 | 0.03268443 | 0.356881901 | -3.521378761 |
| ANGPT2 | -0.800669746 | 1.477699099 | -2.346970443 | 0.032692074 | 0.356881901 | -3.521569084 |
| LYRM1 | 0.299349389 | 6.772036807 | 2.346677221 | 0.032710838 | 0.356881901 | -3.52203612 |
| lnc-SDCCAG8-1 | -0.691029227 | 1.716807852 | -2.346561728 | 0.032718232 | 0.356881901 | -3.522220068 |
| GEMIN4 | -0.403109793 | 6.557512581 | -2.346461835 | 0.032724628 | 0.356881901 | -3.522379166 |
| C19orf54 | 0.525081197 | 3.118371933 | 2.34607921 | 0.032749139 | 0.356881901 | -3.522988544 |
| EIF4B | -0.292272319 | 10.58733689 | -2.345936857 | 0.032758262 | 0.356881901 | -3.52321525 |
| XLOC_l2_007456 | 1.054150017 | 6.939776135 | 2.345808339 | 0.032766501 | 0.356881901 | -3.523419915 |
| HNRNPA1 | -0.535319356 | 12.93822304 | -2.345778679 | 0.032768403 | 0.356881901 | -3.523467149 |
| TMEM54 | 0.850107511 | 1.648107913 | 2.345689349 | 0.032774131 | 0.356881901 | -3.523609405 |
| MAF | -0.450481272 | 8.090410052 | -2.345596102 | 0.032780111 | 0.356881901 | -3.523757897 |
| EDNRA | 0.45632663 | 1.155188387 | 2.345319557 | 0.032797853 | 0.356963159 | -3.524198267 |
| B4GALT5 | 1.050124104 | 9.620516421 | 2.344897679 | 0.032824936 | 0.357146002 | -3.524870026 |
| ZNF639 | -0.451297888 | 5.394351676 | -2.344018988 | 0.032881413 | 0.357255039 | -3.526269016 |
| lnc-MTERFD3-1 | -0.757758219 | 3.8547073 | -2.343794668 | 0.032895845 | 0.357255039 | -3.526626127 |
| CD8A | -0.672867583 | 11.45142504 | -2.34354716 | 0.032911777 | 0.357255039 | -3.52702014 |
| ZNF805 | -0.392976451 | 5.894064852 | -2.343546657 | 0.032911809 | 0.357255039 | -3.52702094 |
| LINC00173 | 0.686225137 | 8.888127273 | 2.343080784 | 0.032941816 | 0.357255039 | -3.527762524 |
| TRIM28 | -0.307726379 | 8.45012042 | -2.342751916 | 0.032963013 | 0.357255039 | -3.528285985 |
| MSH6 | -0.370769475 | 9.134300739 | -2.342704756 | 0.032966054 | 0.357255039 | -3.528361048 |
| LOC101929610 | 0.51177889 | 5.01880257 | 2.342613185 | 0.032971959 | 0.357255039 | -3.528506795 |
| FAM83D | -0.710261241 | 2.124134349 | -2.3426045 | 0.032972519 | 0.357255039 | -3.528520618 |
| GZMB | -0.759388433 | 12.56222668 | -2.342598297 | 0.032972919 | 0.357255039 | -3.528530492 |
| SAMD14 | 1.234306336 | 1.54612928 | 2.342539204 | 0.032976731 | 0.357255039 | -3.528624545 |
| lnc-TTC4-1 | 0.413101653 | 4.228262799 | 2.342501407 | 0.032979169 | 0.357255039 | -3.528684703 |
| VPS13A-AS1 | 0.889512946 | 5.03040268 | 2.342240731 | 0.032995988 | 0.357255039 | -3.529099583 |
| ZNF667 | 0.332683047 | 7.104929831 | 2.342231761 | 0.032996567 | 0.357255039 | -3.529113859 |
| CNKSR1 | -0.707254206 | 4.661463545 | -2.342114289 | 0.033004149 | 0.357255039 | -3.529300817 |
| NARF | 0.481151436 | 9.848604387 | 2.342104997 | 0.033004749 | 0.357255039 | -3.529315605 |
| CYP2D6 | 0.376474273 | 4.799534394 | 2.342026273 | 0.033009831 | 0.357255039 | -3.529440892 |
| CACHD1 | -0.592625059 | 1.076046876 | -2.34171354 | 0.033030027 | 0.357362256 | -3.529938581 |
| PRDM5 | 0.715528331 | 5.048687786 | 2.341242318 | 0.033060481 | 0.357580347 | -3.530688442 |
| TMEM186 | -0.33521474 | 6.46193303 | -2.341075054 | 0.033071297 | 0.357585971 | -3.530954597 |
| LPPR2 | 0.655347488 | 6.63782666 | 2.34072929 | 0.033093666 | 0.357640082 | -3.53150476 |
| GTPBP6 | -0.295364459 | 10.74256054 | -2.340679372 | 0.033096897 | 0.357640082 | -3.531584184 |
| PCDHB19P | 0.501965137 | 1.139805324 | 2.340279577 | 0.033122782 | 0.357682866 | -3.532220274 |
| IRF8 | -0.526829607 | 9.650138441 | -2.339865642 | 0.033149602 | 0.357682866 | -3.532878814 |
| lnc-SMARCC2-4 | -0.417287445 | 1.163405643 | -2.339861902 | 0.033149845 | 0.357682866 | -3.532884764 |
| CASP4 | 0.324951944 | 12.74291551 | 2.339725543 | 0.033158684 | 0.357682866 | -3.53310169 |
| XLOC_l2_001953 | -0.497943558 | 4.479668062 | -2.339657401 | 0.033163103 | 0.357682866 | -3.533210093 |
| CCT4 | -0.53267727 | 12.04731624 | -2.339641195 | 0.033164154 | 0.357682866 | -3.533235874 |
| ANO10 | 0.339550395 | 8.96059606 | 2.339144701 | 0.033196364 | 0.357682866 | -3.534025665 |
| INTS2 | -0.459910601 | 3.80147455 | -2.338930412 | 0.033210275 | 0.357682866 | -3.534366522 |
| CYP4F2 | 1.037257196 | 4.101227836 | 2.338782171 | 0.033219901 | 0.357682866 | -3.534602313 |
| BIN1 | -0.434806845 | 7.624255258 | -2.3387342 | 0.033223017 | 0.357682866 | -3.534678613 |
| lnc-OXNAD1-1 | 0.366039248 | 5.538918904 | 2.33868111 | 0.033226466 | 0.357682866 | -3.534763057 |
| GULP1 | 0.342210039 | 0.856855741 | 2.338677916 | 0.033226673 | 0.357682866 | -3.534768136 |
| RHOBTB3 | -0.998271591 | 2.769882938 | -2.338337334 | 0.033248804 | 0.357682866 | -3.535309831 |
| HS6ST1 | -0.273708101 | 7.764670514 | -2.33820984 | 0.033257093 | 0.357682866 | -3.535512601 |
| NLRC5 | 0.494734932 | 7.358338227 | 2.338117799 | 0.033263077 | 0.357682866 | -3.535658982 |
| lnc-C1orf124-1 | 0.530807447 | 0.996491945 | 2.338078399 | 0.033265639 | 0.357682866 | -3.535721644 |
| TIMM13 | -0.413465929 | 4.496353821 | -2.337800878 | 0.033283692 | 0.357766207 | -3.536162996 |
| LOC389247 | -0.669204183 | 4.284974334 | -2.337481298 | 0.033304491 | 0.357879018 | -3.536671211 |
| ARF3 | 0.268927201 | 10.44529338 | 2.337200232 | 0.033322794 | 0.35796494 | -3.537118155 |
| NR2C1 | -0.404623649 | 4.740922959 | -2.33692821 | 0.033340518 | 0.358044581 | -3.537550695 |
| LHFPL4 | 0.512045223 | 3.434975307 | 2.336559352 | 0.033364564 | 0.35810503 | -3.538137184 |
| lnc-NDNL2-1 | 0.545440686 | 1.027170521 | 2.33638662 | 0.03337583 | 0.35810503 | -3.538411815 |
| EXOG | -0.380386832 | 6.722490288 | -2.336273753 | 0.033383194 | 0.35810503 | -3.538591263 |
| EXOC6 | 0.395188181 | 9.494494218 | 2.336082465 | 0.033395677 | 0.35810503 | -3.538895383 |
| DNAJC11 | -0.27222194 | 7.026842139 | -2.335971861 | 0.033402897 | 0.35810503 | -3.539071221 |
| CYP39A1 | -0.390651573 | 1.203008295 | -2.335790032 | 0.03341477 | 0.35810503 | -3.539360288 |
| SAP30L | 0.156469232 | 8.302879785 | 2.335606997 | 0.033426725 | 0.35810503 | -3.539651262 |
| RAD51 | -0.768816972 | 4.811292073 | -2.335577752 | 0.033428635 | 0.35810503 | -3.539697752 |
| GOLGA3 | -0.352208336 | 9.115557815 | -2.335379503 | 0.03344159 | 0.358133336 | -3.5400129 |
| LOC101927344 | -0.514753325 | 0.999135683 | -2.334903983 | 0.033472681 | 0.358286628 | -3.540768772 |
| lnc-RP11-1220K2.2.1-1 | 1.14026693 | 5.010684119 | 2.334822187 | 0.033478032 | 0.358286628 | -3.540898785 |
| PTGS1 | 0.622527042 | 6.311174953 | 2.334687372 | 0.033486853 | 0.358286628 | -3.541113068 |
| C15orf39 | 0.379366483 | 9.864269402 | 2.334350319 | 0.033508916 | 0.358349873 | -3.541648779 |
| ZFPM1 | -0.356927199 | 6.897436135 | -2.334281836 | 0.0335134 | 0.358349873 | -3.541757621 |
| POLR2E | -0.224619769 | 10.63990124 | -2.33356811 | 0.033560171 | 0.358669579 | -3.542891895 |
| RNF144A | -0.369560146 | 4.872047268 | -2.33351041 | 0.033563954 | 0.358669579 | -3.542983587 |
| ZNF569 | -0.657595407 | 6.457322401 | -2.333253401 | 0.033580813 | 0.358727189 | -3.543391995 |
| OR4X2 | 0.722551721 | 2.632935307 | 2.333113342 | 0.033590003 | 0.358727189 | -3.543614553 |
| PABPC4 | -0.377712658 | 10.03171261 | -2.332527935 | 0.033628443 | 0.358822973 | -3.544544718 |
| TEP1 | 0.297994097 | 8.537717041 | 2.332448946 | 0.033633633 | 0.358822973 | -3.544670218 |
| POM121 | -0.241547523 | 9.010044833 | -2.332433185 | 0.033634668 | 0.358822973 | -3.54469526 |
| XLOC_l2_010601 | -1.26651003 | 1.582497172 | -2.332249427 | 0.033646745 | 0.358822973 | -3.544987213 |
| RNF43 | -0.62293841 | 5.163870474 | -2.332190308 | 0.033650631 | 0.358822973 | -3.545081139 |
| lnc-C1orf201-3 | -0.581198835 | 6.799613474 | -2.331462676 | 0.033698497 | 0.35922308 | -3.54623709 |
| GAS8-AS1 | 0.867606433 | 1.727922105 | 2.33114557 | 0.033719377 | 0.359277784 | -3.546740813 |
| TSEN34 | 0.488028547 | 12.4957305 | 2.331070552 | 0.033724318 | 0.359277784 | -3.546859975 |
| PYCR1 | -0.731999621 | 5.424199301 | -2.330555816 | 0.033758242 | 0.359528902 | -3.547677564 |
| NIT1 | 0.287689463 | 6.66663335 | 2.330234248 | 0.033779451 | 0.359601656 | -3.548188293 |
| lnc-SOX2-1 | -0.641902781 | 1.638259618 | -2.329945984 | 0.033798475 | 0.359601656 | -3.548646104 |
| lnc-TCOF1-1 | -0.450969679 | 0.885243837 | -2.329893624 | 0.033801931 | 0.359601656 | -3.548729257 |
| DPH5 | -0.481748762 | 6.937763203 | -2.329773364 | 0.033809871 | 0.359601656 | -3.548920242 |
| PRPH | 1.266303346 | 2.157087894 | 2.329602253 | 0.033821171 | 0.359601656 | -3.549191975 |
| C1orf216 | -0.922295503 | 2.459256964 | -2.329365092 | 0.03383684 | 0.359601656 | -3.549568586 |
| lnc-NUMB-2 | 0.66105902 | 6.827377127 | 2.329150687 | 0.033851011 | 0.359601656 | -3.549909047 |
| HCRTR1 | 0.452731537 | 5.39567606 | 2.329049541 | 0.033857698 | 0.359601656 | -3.550069655 |
| lnc-GBP5-3 | 1.040428551 | 1.897782296 | 2.329007977 | 0.033860446 | 0.359601656 | -3.550135654 |
| lnc-RNF113B-2 | -0.411342045 | 0.874903052 | -2.328802742 | 0.033874019 | 0.359601656 | -3.550461532 |
| HTR2A | -1.047543717 | 1.228772689 | -2.328727909 | 0.03387897 | 0.359601656 | -3.550580353 |
| lnc-TRAPPC4-1 | -0.745027929 | 2.065797589 | -2.328398637 | 0.03390076 | 0.359723007 | -3.55110315 |
| XLOC_l2_001669 | -0.458945878 | 3.419079691 | -2.328196134 | 0.033914168 | 0.359755361 | -3.551424657 |
| HUWE1 | -0.211974407 | 7.872225526 | -2.327601165 | 0.03395359 | 0.359933488 | -3.552369202 |
| ZNF678 | -0.507786885 | 6.2658725 | -2.327489226 | 0.033961011 | 0.359933488 | -3.5525469 |
| LGALS12 | 0.816584478 | 7.208778752 | 2.327473543 | 0.033962051 | 0.359933488 | -3.552571796 |
| MUTYH | -0.268744201 | 8.344294928 | -2.32706243 | 0.033989322 | 0.36011262 | -3.553224388 |
| ZDHHC19 | 0.686653439 | 1.258419068 | 2.326875986 | 0.034001697 | 0.360133862 | -3.553520329 |
| BRWD3 | 0.458631831 | 5.267836125 | 2.326533426 | 0.034024444 | 0.360166964 | -3.554064047 |
| LOC646652 | 0.872095489 | 5.032351313 | 2.32651658 | 0.034025563 | 0.360166964 | -3.554090784 |
| MVP | 0.373085466 | 13.2837105 | 2.325918416 | 0.034065319 | 0.360362834 | -3.555040122 |
| NAT8L | 0.27381827 | 8.072794241 | 2.325835813 | 0.034070812 | 0.360362834 | -3.555171211 |
| CYB5B | -0.435894875 | 5.268231739 | -2.325769911 | 0.034075196 | 0.360362834 | -3.555275795 |
| FLJ44838 | -0.482516354 | 1.371828686 | -2.325205686 | 0.034112746 | 0.360550947 | -3.556171152 |
| UGT1A6 | 0.923619158 | 3.160590967 | 2.324995861 | 0.03412672 | 0.360550947 | -3.556504097 |
| POGLUT1 | -0.435843464 | 7.165909634 | -2.324923893 | 0.034131514 | 0.360550947 | -3.55661829 |
| EDF1 | -0.301636388 | 11.56668547 | -2.324755937 | 0.034142705 | 0.360550947 | -3.556884787 |
| C17orf107 | -0.716346359 | 3.683492202 | -2.32472314 | 0.034144891 | 0.360550947 | -3.556936824 |
| MOSPD2 | 0.550464604 | 8.568634803 | 2.324567091 | 0.034155292 | 0.360551157 | -3.557184419 |
| TMTC3 | -0.879224181 | 3.588990177 | -2.324411169 | 0.034165688 | 0.360551308 | -3.557431805 |
| DGAT2 | 0.908767285 | 7.49890318 | 2.323710167 | 0.034212463 | 0.360935254 | -3.55854393 |
| lnc-HEPH-1 | 0.786256453 | 9.271911749 | 2.323530329 | 0.034224473 | 0.360952307 | -3.558829218 |
| SNHG22 | -0.556714679 | 4.934435642 | -2.323170749 | 0.034248498 | 0.361096029 | -3.559399612 |
| ZFP82 | -0.57499183 | 4.617971141 | -2.322923495 | 0.034265027 | 0.361154762 | -3.559791805 |
| ZNRF1 | -0.612533752 | 3.78292997 | -2.322776362 | 0.034274866 | 0.361154762 | -3.560025179 |
| lnc-SLCO2B1-2 | -0.31312116 | 3.998974512 | -2.322177244 | 0.034314959 | 0.361333345 | -3.560975401 |
| lnc-ABCC5-1 | 0.802186778 | 3.652536526 | 2.322124402 | 0.034318497 | 0.361333345 | -3.561059206 |
| ANKRD13C | -0.374850568 | 4.902092365 | -2.321995623 | 0.034327122 | 0.361333345 | -3.56126344 |
| MRPS5 | -0.353121646 | 11.05682556 | -2.321762797 | 0.034342719 | 0.361333345 | -3.56163267 |
| lnc-RUNDC3A-1 | -0.3817994 | 4.497539123 | -2.321665194 | 0.03434926 | 0.361333345 | -3.561787451 |
| CCNB2 | -1.010378019 | 5.754796712 | -2.321590915 | 0.034354239 | 0.361333345 | -3.561905242 |
| CERS6 | -0.38499791 | 9.124464779 | -2.32121883 | 0.034379187 | 0.361486279 | -3.562495271 |
| lnc-HDAC2-1 | 0.760278 | 1.298503848 | 2.320457806 | 0.034430268 | 0.361913805 | -3.563701931 |
| PURA | -0.413246351 | 9.415497482 | -2.320285962 | 0.034441812 | 0.36192561 | -3.56397438 |
| JAK2 | 0.498722444 | 8.327708225 | 2.320080896 | 0.034455593 | 0.361948602 | -3.564299489 |
| DNAJC9 | -0.347047165 | 8.438285217 | -2.319792461 | 0.034474985 | 0.361948602 | -3.564756752 |
| NTNG2 | 0.847137551 | 9.870066825 | 2.319600872 | 0.034487872 | 0.361948602 | -3.56506047 |
| ZBTB43 | -0.174207571 | 9.06155011 | -2.319500894 | 0.034494598 | 0.361948602 | -3.565218955 |
| CD163L1 | 1.01911045 | 2.310643057 | 2.319309473 | 0.03450748 | 0.361948602 | -3.565522391 |
| CBX6 | -0.353272104 | 10.80493081 | -2.319203299 | 0.034514627 | 0.361948602 | -3.56569069 |
| FEZ1 | -0.904474214 | 5.0664879 | -2.319109071 | 0.034520972 | 0.361948602 | -3.565840051 |
| TTTY14 | 0.373649431 | 8.30499589 | 2.318890909 | 0.034535664 | 0.361948602 | -3.56618585 |
| FN3KRP | -0.394042064 | 8.763184349 | -2.318859258 | 0.034537796 | 0.361948602 | -3.566236018 |
| LOC400684 | -0.743065592 | 2.89377429 | -2.318434425 | 0.034566426 | 0.36204802 | -3.566909359 |
| lnc-ANKRD33B-1 | 0.787036936 | 1.162745837 | 2.318306874 | 0.034575026 | 0.36204802 | -3.567111513 |
| lnc-CCDC107-2 | 0.565008403 | 3.172392307 | 2.318146516 | 0.034585841 | 0.36204802 | -3.567365654 |
| S100A12 | 0.697889055 | 13.13593271 | 2.318099962 | 0.034588982 | 0.36204802 | -3.567439434 |
| BARD1 | -0.418834446 | 6.136323488 | -2.317873313 | 0.034604274 | 0.362069574 | -3.567798621 |
| XLOC_l2_009510 | 1.090272512 | 1.639920734 | 2.317681765 | 0.034617203 | 0.362069574 | -3.56810217 |
| LOC388210 | 0.893176003 | 4.004897782 | 2.317606027 | 0.034622317 | 0.362069574 | -3.568222189 |
| USP39 | -0.264931377 | 10.83864151 | -2.317230221 | 0.034647699 | 0.362171935 | -3.568817696 |
| TMEM106B | -0.532234187 | 7.917462492 | -2.317152352 | 0.034652961 | 0.362171935 | -3.568941082 |
| XLOC_l2_014289 | -0.760260586 | 2.475643523 | -2.316912632 | 0.034669164 | 0.362219358 | -3.569320919 |
| TTC27 | -0.466396917 | 8.053667872 | -2.316578204 | 0.03469178 | 0.362219358 | -3.569850794 |
| TMEM56-RWDD3 | -0.537899679 | 3.694565537 | -2.316476834 | 0.034698638 | 0.362219358 | -3.5700114 |
| PHF20L1 | 0.49562265 | 9.444426083 | 2.316119792 | 0.034722803 | 0.362219358 | -3.570577059 |
| lnc-GLYATL2-1 | 0.388053474 | 1.235715737 | 2.315879689 | 0.034739062 | 0.362219358 | -3.570957433 |
| ANO7 | 0.952831667 | 3.661934984 | 2.315854922 | 0.034740739 | 0.362219358 | -3.570996668 |
| TMBIM4 | 0.2069768 | 12.00331416 | 2.315093352 | 0.034792361 | 0.362219358 | -3.57220304 |
| ABCA6 | -0.422435845 | 2.567770586 | -2.31503184 | 0.034796534 | 0.362219358 | -3.572300472 |
| CCDC34 | -0.692564697 | 5.161790802 | -2.314776935 | 0.03481383 | 0.362219358 | -3.572704216 |
| HJURP | -1.008637863 | 4.745535703 | -2.31468731 | 0.034819913 | 0.362219358 | -3.572846169 |
| SLC7A1 | -0.708257009 | 5.732670136 | -2.314686717 | 0.034819953 | 0.362219358 | -3.572847107 |
| lnc-STX16-1 | 0.677716802 | 3.228485893 | 2.314607415 | 0.034825337 | 0.362219358 | -3.572972708 |
| BICD1 | -0.909921652 | 1.685794797 | -2.314546134 | 0.034829498 | 0.362219358 | -3.573069765 |
| IFNLR1 | -0.614498424 | 6.677822319 | -2.314514994 | 0.034831612 | 0.362219358 | -3.573119084 |
| XLOC_l2_015762 | -0.41232929 | 10.08730585 | -2.3145023 | 0.034832474 | 0.362219358 | -3.57313919 |
| SMIM17 | -0.568039765 | 2.156822014 | -2.314381417 | 0.034840683 | 0.362219358 | -3.57333064 |
| NME3 | -0.289050816 | 10.44442013 | -2.314365766 | 0.034841746 | 0.362219358 | -3.573355427 |
| lnc-ZNF726-1 | -0.347475623 | 3.833950789 | -2.314314461 | 0.034845231 | 0.362219358 | -3.573436681 |
| ALG11 | -0.226742928 | 8.86642955 | -2.313834429 | 0.034877853 | 0.36242266 | -3.574196891 |
| TRG-AS1 | -0.587101635 | 8.215236735 | -2.313701017 | 0.034886924 | 0.36242266 | -3.574408158 |
| HLA-DOA | -0.542834554 | 10.3654419 | -2.313566171 | 0.034896095 | 0.36242266 | -3.574621693 |
| UAP1 | -0.609350248 | 4.968737736 | -2.313196435 | 0.034921253 | 0.362523557 | -3.575207156 |
| LOC100505824 | 0.445728247 | 3.173197821 | 2.312995282 | 0.034934947 | 0.362523557 | -3.57552566 |
| SRGN | 0.462926348 | 13.19865122 | 2.312880272 | 0.034942779 | 0.362523557 | -3.57570776 |
| DNAJC14 | -0.172688464 | 7.924006201 | -2.312699118 | 0.034955119 | 0.362523557 | -3.575994581 |
| lnc-C10orf10-1 | 0.482218762 | 1.212594664 | 2.312656803 | 0.034958002 | 0.362523557 | -3.576061577 |
| TNPO2 | -0.229328288 | 9.498619024 | -2.312060663 | 0.03499864 | 0.362711066 | -3.577005372 |
| lnc-THOC5-1 | 0.372611992 | 3.920040264 | 2.311941418 | 0.035006775 | 0.362711066 | -3.577194144 |
| lnc-FAM168A-1 | 0.513305508 | 4.712476279 | 2.311932045 | 0.035007414 | 0.362711066 | -3.577208983 |
| SCARNA11 | 0.666455189 | 1.683856246 | 2.311645154 | 0.035026992 | 0.362805676 | -3.577663136 |
| TNFRSF1A | 0.514299946 | 9.953304981 | 2.311329766 | 0.035048526 | 0.362834632 | -3.578162372 |
| NLGN1 | -0.376429047 | 1.064153409 | -2.311298202 | 0.035050682 | 0.362834632 | -3.578212334 |
| RPS28 | -0.386844154 | 15.62469654 | -2.311021071 | 0.035069616 | 0.362922453 | -3.578650987 |
| CARD14 | 0.411888262 | 6.19763038 | 2.310741547 | 0.035088722 | 0.36298271 | -3.579093405 |
| LOC102546298 | -0.866952713 | 2.111322362 | -2.310481957 | 0.035106475 | 0.36298271 | -3.579504253 |
| GSTM5 | 0.411715731 | 6.280610952 | 2.310477312 | 0.035106793 | 0.36298271 | -3.579511604 |
| lnc-HIVEP1-2 | -0.482331635 | 2.867108638 | -2.310172404 | 0.035127656 | 0.363027947 | -3.579994151 |
| C9orf142 | -0.32555499 | 9.005688917 | -2.310107859 | 0.035132074 | 0.363027947 | -3.580096295 |
| SPC25 | -1.41184284 | 3.150827867 | -2.309802787 | 0.035152962 | 0.363135747 | -3.58057907 |
| TMEM217 | 0.340759588 | 4.998484728 | 2.309268509 | 0.035189573 | 0.363137896 | -3.581424497 |
| lnc-FGD4-1 | 0.803611526 | 2.481323264 | 2.309234173 | 0.035191927 | 0.363137896 | -3.581478826 |
| lnc-CLEC18B-2 | -0.332736917 | 7.483473235 | -2.309228524 | 0.035192314 | 0.363137896 | -3.581487764 |
| HSD17B12 | -0.164910752 | 8.068830265 | -2.30908141 | 0.035202401 | 0.363137896 | -3.581720537 |
| PTH1R | -0.940652789 | 1.429689714 | -2.309001583 | 0.035207876 | 0.363137896 | -3.581846843 |
| GNLY | -0.646235311 | 11.95315942 | -2.308884521 | 0.035215906 | 0.363137896 | -3.582032058 |
| RFX7 | -0.413936136 | 7.603804163 | -2.308659257 | 0.035231364 | 0.363138962 | -3.58238846 |
| AFG3L2 | -0.408773867 | 5.210830539 | -2.308495501 | 0.035242604 | 0.363138962 | -3.582647539 |
| CRHR2 | 1.394512294 | 3.416638829 | 2.308192874 | 0.035263386 | 0.363138962 | -3.583126305 |
| HECW1 | 0.587557641 | 1.084778827 | 2.307765068 | 0.035292784 | 0.363138962 | -3.583803063 |
| SNX13 | 0.283486512 | 8.038469171 | 2.30769996 | 0.03529726 | 0.363138962 | -3.583906054 |
| CD44 | -0.307550818 | 12.73908499 | -2.307551793 | 0.035307449 | 0.363138962 | -3.584140429 |
| lnc-SLC46A2-2 | 0.542216802 | 1.198734283 | 2.307397443 | 0.035318065 | 0.363138962 | -3.584384577 |
| RNF149 | 0.480319221 | 12.80809306 | 2.307097784 | 0.035338684 | 0.363138962 | -3.584858554 |
| TLR2 | 0.58535052 | 12.51495629 | 2.306780146 | 0.035360553 | 0.363138962 | -3.58536094 |
| GSTA4 | -0.922483013 | 3.491263279 | -2.306713735 | 0.035365127 | 0.363138962 | -3.585465974 |
| CEP120 | -0.375991328 | 6.824093782 | -2.306569142 | 0.035375088 | 0.363138962 | -3.585694654 |
| OR2A9P | -0.563273275 | 5.164382178 | -2.306291127 | 0.035394246 | 0.363138962 | -3.586134331 |
| lnc-OSTF1-1 | 0.686479776 | 4.312649102 | 2.306219786 | 0.035399164 | 0.363138962 | -3.586247151 |
| NBEAL2 | 0.68373696 | 9.182618344 | 2.306124403 | 0.03540574 | 0.363138962 | -3.586397992 |
| DHRS12 | 0.509601919 | 8.593525513 | 2.306084754 | 0.035408474 | 0.363138962 | -3.586460692 |
| PRPS1L1 | -0.472625288 | 4.339892066 | -2.306082856 | 0.035408605 | 0.363138962 | -3.586463693 |
| VWA9 | -0.396959484 | 6.378549298 | -2.305990545 | 0.035414971 | 0.363138962 | -3.586609671 |
| KIAA0930 | 0.273583105 | 6.859354297 | 2.305958805 | 0.03541716 | 0.363138962 | -3.586659864 |
| FCHO2 | 0.597297664 | 7.56579819 | 2.305676911 | 0.035436608 | 0.363138962 | -3.587105625 |
| AP1M1 | -0.166219178 | 8.734799139 | -2.305507136 | 0.035448325 | 0.363138962 | -3.587374081 |
| GAS2 | -0.679879133 | 1.160514036 | -2.30528159 | 0.035463898 | 0.363138962 | -3.587730713 |
| GABRR3 | 0.94759556 | 1.711170979 | 2.305230317 | 0.035467439 | 0.363138962 | -3.587811782 |
| ZCRB1 | -0.349883058 | 6.989790594 | -2.304868375 | 0.035492444 | 0.363138962 | -3.588384044 |
| FAM214B | 0.47650627 | 7.687228507 | 2.304544834 | 0.03551481 | 0.363138962 | -3.58889556 |
| NPIPB3 | -0.564861923 | 1.18652354 | -2.304543968 | 0.03551487 | 0.363138962 | -3.588896929 |
| HNRNPDL | -0.412299323 | 9.353538192 | -2.304532871 | 0.035515637 | 0.363138962 | -3.588914472 |
| CNNM3 | -0.253579367 | 7.291835966 | -2.304515375 | 0.035516847 | 0.363138962 | -3.588942133 |
| OSBPL6 | 1.141504526 | 3.025990594 | 2.304346931 | 0.035528497 | 0.363138962 | -3.589208428 |
| POLR1E | -0.579801622 | 4.812938919 | -2.304290304 | 0.035532415 | 0.363138962 | -3.589297949 |
| LRIG2 | -0.390359409 | 6.706625516 | -2.30418632 | 0.035539609 | 0.363138962 | -3.589462332 |
| LLGL2 | -0.494778446 | 10.00566072 | -2.304060608 | 0.035548309 | 0.363138962 | -3.589661062 |
| SLC16A10 | -1.039510009 | 3.065557058 | -2.304027453 | 0.035550604 | 0.363138962 | -3.589713473 |
| LOC102467146 | 0.660427051 | 7.422819452 | 2.303805345 | 0.03556598 | 0.363187084 | -3.590064573 |
| SMO | -0.488953678 | 2.898136759 | -2.30355567 | 0.035583272 | 0.363187084 | -3.590459233 |
| SCARNA22 | 0.355442866 | 8.73313383 | 2.303506378 | 0.035586687 | 0.363187084 | -3.590537147 |
| SIGLEC11 | 0.406785117 | 5.623846082 | 2.30296909 | 0.035623929 | 0.363297503 | -3.591386367 |
| NFATC1 | -0.277773341 | 8.723160987 | -2.302932237 | 0.035626485 | 0.363297503 | -3.591444613 |
| C1QTNF3 | -0.63576087 | 3.631078049 | -2.302765692 | 0.035638037 | 0.363297503 | -3.591707831 |
| FAM8A1 | 0.420500307 | 5.838353339 | 2.302746786 | 0.035639349 | 0.363297503 | -3.591737711 |
| METTL22 | 0.232374107 | 7.394234375 | 2.302478145 | 0.035657991 | 0.363380877 | -3.592162267 |
| lnc-VSTM2B-1 | 0.778214074 | 4.757578034 | 2.302057871 | 0.035687174 | 0.36357159 | -3.592826422 |
| lnc-CAPN13-1 | -0.271385595 | 0.769205764 | -2.301755975 | 0.035708151 | 0.363678614 | -3.593303474 |
| lnc-OIT3-2 | 0.433145684 | 5.577154691 | 2.301368721 | 0.035735075 | 0.363844226 | -3.593915369 |
| SPATA31A3 | 0.300059124 | 1.14879619 | 2.30122081 | 0.035745364 | 0.363844226 | -3.594149071 |
| lnc-SNRPD1-2 | 0.698971707 | 1.783357239 | 2.300664329 | 0.035784098 | 0.363906414 | -3.59502826 |
| lnc-SPINK9-1 | 0.533859364 | 4.235223555 | 2.300529998 | 0.035793454 | 0.363906414 | -3.595240477 |
| YIPF3 | 0.326838442 | 10.29916268 | 2.300411015 | 0.035801744 | 0.363906414 | -3.595428443 |
| CD59 | 0.624520953 | 4.046103768 | 2.300354324 | 0.035805694 | 0.363906414 | -3.595518 |
| PPT2 | -0.227578193 | 6.229762421 | -2.300162312 | 0.035819075 | 0.363906414 | -3.595821323 |
| LINC00665 | -0.317259261 | 6.324712659 | -2.300007768 | 0.035829849 | 0.363906414 | -3.596065451 |
| lnc-KLHL28-2 | 0.592819431 | 1.488945127 | 2.299945934 | 0.035834161 | 0.363906414 | -3.596163126 |
| LOC730081 | -0.430426299 | 1.166081715 | -2.299929612 | 0.035835299 | 0.363906414 | -3.596188908 |
| LGALS9 | 0.505874659 | 7.873002771 | 2.299736267 | 0.035848784 | 0.363916025 | -3.596494314 |
| HCRT | 1.400100142 | 3.955747611 | 2.299437817 | 0.035869609 | 0.363916025 | -3.596965721 |
| FAM184A | -1.217664533 | 3.759319077 | -2.299354376 | 0.035875434 | 0.363916025 | -3.597097512 |
| KIAA1377 | -0.792429137 | 1.7043221 | -2.299264781 | 0.035881689 | 0.363916025 | -3.597239022 |
| JADE2 | -0.302429082 | 6.826684054 | -2.298812748 | 0.035913262 | 0.363916025 | -3.597952945 |
| ZNF548 | -0.570193045 | 4.215648813 | -2.298796879 | 0.035914371 | 0.363916025 | -3.597978007 |
| ADAMTS4 | -0.896827957 | 3.815410785 | -2.298399886 | 0.035942122 | 0.363916025 | -3.598604951 |
| GLDC | -1.910235826 | 3.537336434 | -2.298303911 | 0.035948834 | 0.363916025 | -3.598756512 |
| XLOC_l2_004817 | -0.402980515 | 5.514365958 | -2.298303059 | 0.035948894 | 0.363916025 | -3.598757857 |
| ALDH6A1 | -0.321186371 | 6.365510533 | -2.298178866 | 0.035957581 | 0.363916025 | -3.598953974 |
| KANTR | -0.187490506 | 7.824244576 | -2.298102258 | 0.035962941 | 0.363916025 | -3.599074945 |
| DCAF7 | -0.268859152 | 8.666217762 | -2.29809892 | 0.035963174 | 0.363916025 | -3.599080216 |
| DDX27 | -0.315392415 | 9.618181858 | -2.297966149 | 0.035972465 | 0.363916025 | -3.59928987 |
| KBTBD8 | -0.689181215 | 3.456701718 | -2.297032054 | 0.036037893 | 0.36429968 | -3.600764727 |
| FAM107B | -0.354399191 | 10.64760449 | -2.296799104 | 0.036054227 | 0.36429968 | -3.601132495 |
| HMCES | -0.237440998 | 5.817375338 | -2.296706227 | 0.036060741 | 0.36429968 | -3.601279121 |
| HDAC3 | -0.214995473 | 10.27067579 | -2.296396169 | 0.036082496 | 0.36429968 | -3.60176859 |
| EIF1AX | -0.515663656 | 9.954042634 | -2.296302739 | 0.036089054 | 0.36429968 | -3.601916077 |
| lnc-CORO6-1 | 0.628492519 | 9.041802648 | 2.296110109 | 0.036102579 | 0.36429968 | -3.60222015 |
| TMEM248 | -0.27002197 | 9.774914503 | -2.296039454 | 0.03610754 | 0.36429968 | -3.602331681 |
| LINC01586 | 0.835933615 | 1.520117874 | 2.295929501 | 0.036115263 | 0.36429968 | -3.602505239 |
| SETD3 | -0.2339387 | 5.283588565 | -2.295902839 | 0.036117136 | 0.36429968 | -3.602547323 |
| IL27 | 0.903846331 | 5.147163507 | 2.295876988 | 0.036118952 | 0.36429968 | -3.602588128 |
| TLE3 | 0.702516872 | 7.139420955 | 2.295773359 | 0.036126233 | 0.36429968 | -3.602751699 |
| LINC00958 | 0.701444288 | 4.640102954 | 2.295540915 | 0.036142569 | 0.36429968 | -3.603118584 |
| COBLL1 | -0.657930622 | 7.314427986 | -2.29548141 | 0.036146752 | 0.36429968 | -3.603212503 |
| TRPV1 | -0.359182288 | 5.74315384 | -2.294643318 | 0.036205716 | 0.364514923 | -3.604535187 |
| XLOC_l2_002204 | -0.687876825 | 2.221072588 | -2.294628815 | 0.036206738 | 0.364514923 | -3.604558074 |
| FKBP1A | 0.268037678 | 8.819223519 | 2.294620333 | 0.036207335 | 0.364514923 | -3.604571459 |
| NEK2 | -0.772281948 | 1.46449293 | -2.294581181 | 0.036210092 | 0.364514923 | -3.604633244 |
| TMEM50A | 0.281305006 | 8.641459881 | 2.294249037 | 0.036233487 | 0.364588022 | -3.605157373 |
| BCAT2 | -0.373779095 | 6.641302438 | -2.294149244 | 0.036240519 | 0.364588022 | -3.605314843 |
| ACAA2 | -0.376340937 | 7.756054726 | -2.29394824 | 0.036254687 | 0.364588022 | -3.60563201 |
| ARHGAP19 | 0.413032082 | 5.998094388 | 2.293882184 | 0.036259344 | 0.364588022 | -3.605736238 |
| lnc-E2F3-1 | 0.524648418 | 1.146435671 | 2.293444203 | 0.036290237 | 0.364666113 | -3.606427286 |
| HNRNPAB | -0.294932102 | 6.104924846 | -2.293399487 | 0.036293393 | 0.364666113 | -3.606497835 |
| RBP7 | 0.458221421 | 8.878868419 | 2.293122677 | 0.036312932 | 0.364666113 | -3.606934554 |
| SNORD9 | 0.715752237 | 1.384031898 | 2.293040817 | 0.036318712 | 0.364666113 | -3.607063699 |
| lnc-ASCL4-1 | 1.388787866 | 2.556611512 | 2.292884302 | 0.036329766 | 0.364666113 | -3.607310617 |
| SNORD19B | 0.481201014 | 1.431516768 | 2.292158573 | 0.036381061 | 0.364666113 | -3.608455432 |
| OLA1 | -0.39587473 | 8.982579664 | -2.291966575 | 0.036394643 | 0.364666113 | -3.608758277 |
| lnc-N4BP2-1 | -0.622741584 | 6.314541206 | -2.29176644 | 0.036408806 | 0.364666113 | -3.609073946 |
| LOC101927204 | 0.222159776 | 5.159400214 | 2.29145089 | 0.036431147 | 0.364666113 | -3.609571633 |
| TNNC2 | 0.734408806 | 5.86789126 | 2.291409051 | 0.03643411 | 0.364666113 | -3.609637621 |
| lnc-AL590822.1-1 | 0.45707117 | 2.296865836 | 2.291403482 | 0.036434504 | 0.364666113 | -3.609646403 |
| ZNF77 | -0.550457257 | 5.871077131 | -2.291351864 | 0.03643816 | 0.364666113 | -3.609727812 |
| SIK2 | -0.48047409 | 3.349125861 | -2.291198844 | 0.036449001 | 0.364666113 | -3.609969141 |
| OS9 | 0.261928751 | 10.28591939 | 2.290006211 | 0.036533593 | 0.364666113 | -3.611849816 |
| lnc-ZPBP-1 | -0.649819722 | 1.586489238 | -2.289848938 | 0.036544762 | 0.364666113 | -3.612097791 |
| NXT1 | -0.67487764 | 8.898290827 | -2.28974574 | 0.036552092 | 0.364666113 | -3.6122605 |
| SRGAP3-AS3 | 0.754388987 | 1.515581216 | 2.289639701 | 0.036559626 | 0.364666113 | -3.612427687 |
| ZNF252P | -0.330508601 | 4.035460453 | -2.289616518 | 0.036561273 | 0.364666113 | -3.612464238 |
| OGG1 | -0.287169405 | 8.736788238 | -2.289382109 | 0.036577933 | 0.364666113 | -3.612833804 |
| SNX5 | -0.460504096 | 10.00252818 | -2.289256188 | 0.036586885 | 0.364666113 | -3.613032323 |
| LOC100034248 | -0.582030919 | 2.877361741 | -2.289185608 | 0.036591904 | 0.364666113 | -3.613143593 |
| LOC101929726 | -0.727945073 | 1.828877061 | -2.28916722 | 0.036593211 | 0.364666113 | -3.613172582 |
| TSC22D4 | 0.368730664 | 8.312013748 | 2.289054075 | 0.036601259 | 0.364666113 | -3.613350952 |
| PTP4A2 | -0.265914671 | 9.35129393 | -2.289017969 | 0.036603827 | 0.364666113 | -3.613407871 |
| lnc-PHACTR4-1 | -0.33702053 | 3.762637975 | -2.288891222 | 0.036612844 | 0.364666113 | -3.613607679 |
| RNF217-AS1 | -0.417382863 | 1.09586831 | -2.288685391 | 0.036627491 | 0.364666113 | -3.613932147 |
| COX11 | -0.464367752 | 8.369863046 | -2.288374203 | 0.036649647 | 0.364666113 | -3.614422676 |
| ZNF264 | -0.334409073 | 9.176056629 | -2.288361783 | 0.036650531 | 0.364666113 | -3.614442251 |
| ELP4 | -0.367667743 | 7.06587289 | -2.288290613 | 0.0366556 | 0.364666113 | -3.614554434 |
| NUCB2 | -0.446418926 | 8.125455552 | -2.288239784 | 0.036659221 | 0.364666113 | -3.614634551 |
| NNT | -0.368480473 | 5.550058338 | -2.287888657 | 0.036684241 | 0.364666113 | -3.615187987 |
| LOC101929331 | 0.696870883 | 6.341147516 | 2.287831907 | 0.036688287 | 0.364666113 | -3.615277431 |
| GLIPR2 | 0.254171358 | 12.73638423 | 2.287680539 | 0.036699079 | 0.364666113 | -3.615515999 |
| RNA18S5 | 0.382638836 | 9.321816889 | 2.287652553 | 0.036701074 | 0.364666113 | -3.615560107 |
| HIST1H2AB | -0.463086145 | 4.151657761 | -2.287375285 | 0.036720851 | 0.364666113 | -3.615997083 |
| CELF4 | 0.38103273 | 1.063679131 | 2.287301485 | 0.036726117 | 0.364666113 | -3.616113389 |
| CYP4A11 | 0.461425131 | 3.480613951 | 2.287052616 | 0.036743879 | 0.364666113 | -3.616505583 |
| BAIAP3 | 0.821474651 | 4.010752752 | 2.287035431 | 0.036745106 | 0.364666113 | -3.616532665 |
| ST3GAL1 | -0.225097857 | 9.154793257 | -2.286847347 | 0.036758536 | 0.364666113 | -3.616829056 |
| XRN2 | 0.300555952 | 6.29338451 | 2.286713569 | 0.036768091 | 0.364666113 | -3.617039863 |
| BPNT1 | -0.384930668 | 6.143725771 | -2.286691355 | 0.036769678 | 0.364666113 | -3.617074867 |
| BCR | -0.291767974 | 8.304975585 | -2.28649128 | 0.036783973 | 0.364666113 | -3.617390132 |
| RPP30 | -0.475813584 | 5.750613848 | -2.286489079 | 0.03678413 | 0.364666113 | -3.6173936 |
| WDR74 | -0.321536735 | 7.059755968 | -2.286339233 | 0.036794839 | 0.364666113 | -3.61762971 |
| lnc-TEFM-2 | 0.484233013 | 5.836668387 | 2.286287601 | 0.03679853 | 0.364666113 | -3.617711066 |
| lnc-CTC-236F12.4.1-4 | 0.497050329 | 1.178954849 | 2.285860012 | 0.03682911 | 0.364666113 | -3.618384768 |
| MFSD7 | 0.502397666 | 7.468633557 | 2.28585402 | 0.036829538 | 0.364666113 | -3.618394209 |
| lnc-KLHL25-4 | 0.62221552 | 1.453501247 | 2.285351767 | 0.036865488 | 0.364666113 | -3.619185482 |
| S100PBP | -0.360837828 | 8.895060631 | -2.285159739 | 0.036879241 | 0.364666113 | -3.619487992 |
| DHFRL1 | -0.425489982 | 5.50296887 | -2.284898336 | 0.036897971 | 0.364666113 | -3.619899776 |
| TCN1 | 0.638148139 | 5.698176691 | 2.284732771 | 0.036909838 | 0.364666113 | -3.620160577 |
| ACTG1 | -0.317860819 | 11.53785465 | -2.284571854 | 0.036921376 | 0.364666113 | -3.620414049 |
| SELP | 0.859535651 | 5.938693966 | 2.284351349 | 0.036937192 | 0.364666113 | -3.620761368 |
| lnc-TNFAIP6-2 | 0.64547957 | 4.885932099 | 2.284218066 | 0.036946755 | 0.364666113 | -3.620971298 |
| LOC101928117 | 0.962519943 | 2.748660862 | 2.284178517 | 0.036949593 | 0.364666113 | -3.621033589 |
| PDCD6IPP2 | -0.266288697 | 8.444203945 | -2.284106812 | 0.036954739 | 0.364666113 | -3.621146525 |
| NCK1-AS1 | -0.519108153 | 4.455429407 | -2.28407366 | 0.036957118 | 0.364666113 | -3.621198741 |
| lnc-VAMP1-1 | 0.369947447 | 5.349685952 | 2.284046369 | 0.036959077 | 0.364666113 | -3.621241724 |
| DHX35 | -0.393503213 | 7.556483116 | -2.283699138 | 0.036984009 | 0.364666113 | -3.621788592 |
| RAD54B | -0.924851671 | 4.430143892 | -2.283642648 | 0.036988067 | 0.364666113 | -3.621877556 |
| ZNF19 | -0.361925192 | 4.108621841 | -2.283520411 | 0.036996848 | 0.364666113 | -3.622070062 |
| lnc-THEMIS-1 | -0.515964417 | 1.186791936 | -2.283261873 | 0.037015428 | 0.364666113 | -3.622477207 |
| UMODL1-AS1 | 1.080298003 | 1.923305575 | 2.283248807 | 0.037016367 | 0.364666113 | -3.622497782 |
| LRRTM3 | 0.496429732 | 0.940972046 | 2.283156159 | 0.037023027 | 0.364666113 | -3.62264368 |
| lnc-ROBO2-1 | 1.250836064 | 1.592443083 | 2.282734655 | 0.037053343 | 0.364666113 | -3.623307408 |
| SLC39A6 | -0.515884591 | 4.103021388 | -2.282550338 | 0.037066607 | 0.364666113 | -3.62359763 |
| TPM1 | 0.418260816 | 5.639503668 | 2.28234809 | 0.037081167 | 0.364666113 | -3.623916073 |
| METTL15 | -0.40149615 | 6.378131022 | -2.282282589 | 0.037085883 | 0.364666113 | -3.624019204 |
| lnc-RMI1-6 | -0.5762223 | 1.275951984 | -2.282261207 | 0.037087423 | 0.364666113 | -3.624052868 |
| KIAA1328 | -0.545757925 | 3.663234925 | -2.282204316 | 0.03709152 | 0.364666113 | -3.624142441 |
| VNN1 | 0.991619269 | 7.320609335 | 2.282174301 | 0.037093682 | 0.364666113 | -3.624189699 |
| EIF3I | -0.340431928 | 9.684293711 | -2.282140541 | 0.037096113 | 0.364666113 | -3.624242852 |
| lnc-RARRES2-2 | -0.401369844 | 1.001643289 | -2.282039997 | 0.037103356 | 0.364666113 | -3.62440115 |
| ASIP | -0.749298316 | 2.273017895 | -2.282022154 | 0.037104641 | 0.364666113 | -3.624429241 |
| SGPP1 | -0.430453386 | 5.133502807 | -2.281956207 | 0.037109393 | 0.364666113 | -3.624533068 |
| CDK19 | 0.330825752 | 10.23500708 | 2.281935051 | 0.037110917 | 0.364666113 | -3.624566375 |
| DGKZ | -0.277147603 | 11.81964088 | -2.281921683 | 0.03711188 | 0.364666113 | -3.624587421 |
| lnc-NKAIN3-1 | -0.956309929 | 1.540643372 | -2.28188387 | 0.037114605 | 0.364666113 | -3.624646952 |
| LINC01299 | 0.555813169 | 2.618456661 | 2.281844155 | 0.037117467 | 0.364666113 | -3.624709477 |
| B4GALT1-AS1 | 0.953345605 | 2.104620775 | 2.281701971 | 0.037127715 | 0.364666113 | -3.624933321 |
| ZNF275 | -0.374890681 | 8.780725562 | -2.28162729 | 0.037133099 | 0.364666113 | -3.625050891 |
| TRAF2 | -0.271244418 | 9.694216927 | -2.281570753 | 0.037137175 | 0.364666113 | -3.625139895 |
| CEP41 | -0.56709507 | 5.096506096 | -2.281527662 | 0.037140282 | 0.364666113 | -3.625207731 |
| lnc-FOXK2-1 | 0.876961825 | 1.786811257 | 2.281405204 | 0.037149113 | 0.364666113 | -3.625400508 |
| TAS2R14 | -0.356244455 | 5.051195123 | -2.28123512 | 0.037161382 | 0.364683474 | -3.625668253 |
| RYK | -0.468449984 | 8.850821556 | -2.280919823 | 0.037184137 | 0.364803691 | -3.626164568 |
| lnc-STARD4-2 | -0.325210546 | 0.926843671 | -2.280652237 | 0.037203458 | 0.364814836 | -3.626585757 |
| NEXN-AS1 | 0.417984638 | 2.349664872 | 2.280613144 | 0.037206281 | 0.364814836 | -3.626647288 |
| FDPS | -0.22337037 | 9.996739481 | -2.280153283 | 0.03723951 | 0.364916054 | -3.627371069 |
| SNHG15 | -0.369462647 | 8.403230643 | -2.280091529 | 0.037243974 | 0.364916054 | -3.627468259 |
| OTUD5 | 0.203270249 | 10.4178183 | 2.280034109 | 0.037248126 | 0.364916054 | -3.627558627 |
| TRIB1 | 0.54806629 | 10.09280512 | 2.27984425 | 0.037261856 | 0.364947618 | -3.627857424 |
| CDC25B | -0.541195804 | 8.706774816 | -2.279573967 | 0.03728141 | 0.365002972 | -3.628282772 |
| RGS18 | 0.429454251 | 11.10690597 | 2.27935371 | 0.037297352 | 0.365002972 | -3.628629377 |
| ANKRD23 | -0.282687924 | 5.05316803 | -2.278883386 | 0.037331415 | 0.365002972 | -3.629369451 |
| HSD17B8 | -0.456816756 | 6.459406394 | -2.278740667 | 0.037341757 | 0.365002972 | -3.629594012 |
| lnc-SPI1-1 | 0.492786407 | 4.687784084 | 2.278316528 | 0.037372508 | 0.365002972 | -3.630261336 |
| VNN3 | 0.846253089 | 4.840346341 | 2.278312932 | 0.037372769 | 0.365002972 | -3.630266995 |
| RHEBL1 | -0.37106513 | 7.501507466 | -2.278269728 | 0.037375902 | 0.365002972 | -3.630334968 |
| AP3D1 | 0.252413133 | 7.962424943 | 2.277962344 | 0.037398205 | 0.365002972 | -3.630818556 |
| STEAP4 | 0.793430236 | 7.588540222 | 2.277645341 | 0.037421218 | 0.365002972 | -3.63131725 |
| C16orf62 | -0.301311391 | 6.592508269 | -2.277625673 | 0.037422647 | 0.365002972 | -3.631348189 |
| CRISPLD2 | 0.911252234 | 4.933419934 | 2.277594558 | 0.037424906 | 0.365002972 | -3.631397137 |
| lnc-C5orf38-4 | 0.458757799 | 3.095240496 | 2.277583488 | 0.03742571 | 0.365002972 | -3.63141455 |
| lnc-MEX3B-2 | -0.682855785 | 1.210908446 | -2.277566681 | 0.037426931 | 0.365002972 | -3.63144099 |
| C2orf88 | 0.899266088 | 6.580689227 | 2.277552475 | 0.037427963 | 0.365002972 | -3.631463336 |
| IRF4 | -0.797832602 | 5.872751283 | -2.27735593 | 0.03744224 | 0.365002972 | -3.631772511 |
| DRGX | 1.243809008 | 1.654439706 | 2.277072127 | 0.037462866 | 0.365002972 | -3.632218926 |
| MGAM | 1.107791518 | 7.019041318 | 2.277049613 | 0.037464503 | 0.365002972 | -3.632254339 |
| lnc-MFSD9-4 | 0.673582005 | 3.541203115 | 2.276926315 | 0.037473467 | 0.365002972 | -3.632448275 |
| FAM53C | 0.628507279 | 8.104229764 | 2.276759982 | 0.037485563 | 0.365002972 | -3.632709895 |
| SNORA58 | -0.366443161 | 6.078001119 | -2.276756279 | 0.037485833 | 0.365002972 | -3.632715719 |
| TAF1A-AS1 | -0.807494349 | 3.510802568 | -2.276723567 | 0.037488212 | 0.365002972 | -3.63276717 |
| SKA2 | -0.473661987 | 4.339779722 | -2.27651399 | 0.037503459 | 0.365049088 | -3.633096791 |
| JDP2 | 0.426887468 | 9.65602303 | 2.275764317 | 0.037558048 | 0.365450964 | -3.63427577 |
| NFATC2 | -0.451251164 | 4.986854306 | -2.275278698 | 0.037593449 | 0.365450964 | -3.635039393 |
| SIMC1 | -0.439215539 | 6.380823419 | -2.274826883 | 0.037626414 | 0.365450964 | -3.635749797 |
| RCL1 | -0.426414534 | 7.545544369 | -2.274626833 | 0.037641018 | 0.365450964 | -3.636064322 |
| HOXA6 | -0.66874811 | 2.256317705 | -2.274599788 | 0.037642993 | 0.365450964 | -3.636106843 |
| FRY-AS1 | 0.72122604 | 4.095246889 | 2.274580137 | 0.037644428 | 0.365450964 | -3.636137738 |
| CCL4L2 | -0.67569343 | 10.09076374 | -2.274234568 | 0.03766967 | 0.365450964 | -3.636681022 |
| LOC100128239 | -0.424753101 | 2.504170006 | -2.274107647 | 0.037678945 | 0.365450964 | -3.63688055 |
| RAB20 | 0.892100346 | 3.667685618 | 2.274005674 | 0.037686399 | 0.365450964 | -3.637040856 |
| TUBB | -0.306785716 | 8.497609031 | -2.273955826 | 0.037690043 | 0.365450964 | -3.637119217 |
| C20orf195 | -0.663894944 | 4.271040365 | -2.273944648 | 0.03769086 | 0.365450964 | -3.637136789 |
| LSMEM1 | 0.831539747 | 7.327812614 | 2.273938831 | 0.037691285 | 0.365450964 | -3.637145934 |
| TMX2 | -0.310954328 | 8.372500739 | -2.273827784 | 0.037699404 | 0.365450964 | -3.637320498 |
| NLRP3 | 0.657160838 | 9.304211489 | 2.273698705 | 0.037708844 | 0.365450964 | -3.637523404 |
| LILRA4 | 0.6055878 | 8.318192003 | 2.273655907 | 0.037711974 | 0.365450964 | -3.637590679 |
| WAS | 0.411806448 | 8.587794087 | 2.273615649 | 0.037714919 | 0.365450964 | -3.637653962 |
| SNRPF | -0.505137184 | 10.1076001 | -2.273427942 | 0.037728652 | 0.365450964 | -3.637949014 |
| MTHFD2L | -0.74049683 | 1.892645601 | -2.273141076 | 0.037749649 | 0.365450964 | -3.638399912 |
| AR | -1.040717304 | 2.663791476 | -2.27314092 | 0.037749661 | 0.365450964 | -3.638400158 |
| AMIGO1 | -0.462922823 | 7.185110479 | -2.272953361 | 0.037763395 | 0.365450964 | -3.638694951 |
| STX7 | 0.285991724 | 7.318840101 | 2.272741673 | 0.037778902 | 0.365450964 | -3.639027658 |
| ORC6 | -0.753205488 | 1.827145361 | -2.272683321 | 0.037783177 | 0.365450964 | -3.639119365 |
| LUC7L2 | -0.33666877 | 7.686534922 | -2.272634334 | 0.037786767 | 0.365450964 | -3.639196354 |
| GPR115 | 0.311078283 | 0.913656562 | 2.271945449 | 0.03783728 | 0.365721803 | -3.640278945 |
| CAD | -0.429123547 | 6.360161226 | -2.271280669 | 0.037886086 | 0.365721803 | -3.641323521 |
| lnc-WFIKKN2-1 | -0.666507478 | 1.448186229 | -2.271091685 | 0.037899972 | 0.365721803 | -3.641620449 |
| NR1I3 | -0.615178707 | 1.856058688 | -2.271003408 | 0.037906459 | 0.365721803 | -3.641759145 |
| NOLC1 | -0.408458369 | 7.838719964 | -2.270869423 | 0.037916308 | 0.365721803 | -3.641969651 |
| PPIL1 | -0.462284407 | 8.154615535 | -2.270719351 | 0.037927342 | 0.365721803 | -3.642205424 |
| ADAMTS9-AS2 | 0.812350757 | 1.47198237 | 2.270564993 | 0.037938695 | 0.365721803 | -3.642447922 |
| LOC101928043 | -0.658789017 | 1.425575539 | -2.270437783 | 0.037948053 | 0.365721803 | -3.642647766 |
| XLOC_l2_001760 | 0.611275548 | 6.73176167 | 2.270107615 | 0.037972352 | 0.365721803 | -3.643166432 |
| PDXDC1 | -0.368306112 | 8.168052911 | -2.27004901 | 0.037976666 | 0.365721803 | -3.643258491 |
| PTPDC1 | -0.540563283 | 5.104120016 | -2.270016075 | 0.037979091 | 0.365721803 | -3.643310226 |
| lnc-RP11-124K4.2.1-3 | 0.895371551 | 3.114977244 | 2.269931366 | 0.037985329 | 0.365721803 | -3.643443289 |
| PNPLA4 | -0.738168864 | 3.204460938 | -2.269739042 | 0.037999494 | 0.365721803 | -3.643745388 |
| NPM3 | -0.524141274 | 7.126844 | -2.26969408 | 0.038002806 | 0.365721803 | -3.643816012 |
| LRRC20 | -0.437293448 | 5.632420968 | -2.269626183 | 0.038007809 | 0.365721803 | -3.643922659 |
| CTPS2 | -0.196059375 | 6.669371777 | -2.269547447 | 0.03801361 | 0.365721803 | -3.644046331 |
| SCLY | -0.22079994 | 6.993046872 | -2.269465638 | 0.03801964 | 0.365721803 | -3.644174827 |
| CNOT6L | -0.364230533 | 9.305041314 | -2.269391772 | 0.038025084 | 0.365721803 | -3.644290845 |
| C12orf56 | 0.378156648 | 1.06792487 | 2.269187787 | 0.038040123 | 0.365721803 | -3.644611226 |
| NT5C3B | -0.896023687 | 3.468492229 | -2.269042945 | 0.038050805 | 0.365721803 | -3.64483871 |
| lnc-IL18-1 | 0.470165225 | 4.730048773 | 2.269029687 | 0.038051783 | 0.365721803 | -3.644859533 |
| LOC102724507 | 0.583775518 | 1.174201337 | 2.269021888 | 0.038052358 | 0.365721803 | -3.644871781 |
| PEMT | -0.43519912 | 6.264831186 | -2.268875159 | 0.038063183 | 0.365721803 | -3.645102221 |
| RAB29 | -0.274000215 | 7.443452069 | -2.268816631 | 0.038067501 | 0.365721803 | -3.645194138 |
| TGFBR3 | -0.652799086 | 5.719738809 | -2.268275014 | 0.038107486 | 0.365900488 | -3.646044691 |
| THAP7 | -0.357337693 | 5.178992151 | -2.268204311 | 0.038112709 | 0.365900488 | -3.646155717 |
| MIF-AS1 | -0.54281481 | 6.131914634 | -2.268136657 | 0.038117707 | 0.365900488 | -3.646261951 |
| lnc-CGN-1 | 0.536209627 | 2.972115569 | 2.267803298 | 0.038142343 | 0.365994654 | -3.646785399 |
| TIMM21 | -0.465416441 | 7.169480738 | -2.267600061 | 0.03815737 | 0.365994654 | -3.647104511 |
| SNORA38 | 0.423839097 | 3.612530905 | 2.267576242 | 0.038159132 | 0.365994654 | -3.647141909 |
| XLOC_l2_015033 | 0.752189711 | 7.452551477 | 2.267305602 | 0.038179152 | 0.366085572 | -3.64756683 |
| lnc-METAP1-2 | 0.630981009 | 3.776597963 | 2.266714241 | 0.038222931 | 0.366144107 | -3.648495229 |
| EOGT | -0.479542608 | 1.273265994 | -2.266566332 | 0.038233888 | 0.366144107 | -3.64872742 |
| METTL23 | -0.347121601 | 10.26930514 | -2.266545974 | 0.038235397 | 0.366144107 | -3.648759378 |
| SHISA3 | -0.829989082 | 1.438901511 | -2.266536229 | 0.038236119 | 0.366144107 | -3.648774675 |
| ENOSF1 | -0.502575274 | 7.500563408 | -2.266310112 | 0.038252876 | 0.366144107 | -3.649129624 |
| CDH15 | -0.325477095 | 0.880985175 | -2.266196741 | 0.038261281 | 0.366144107 | -3.649307584 |
| AMIGO2 | -0.3941651 | 7.21405677 | -2.266126034 | 0.038266523 | 0.366144107 | -3.64941857 |
| ZDHHC21 | -0.274123701 | 7.559589115 | -2.265814883 | 0.038289601 | 0.366144107 | -3.64990696 |
| lnc-FPGT-1 | -0.331950167 | 0.956993943 | -2.265368522 | 0.038322731 | 0.366144107 | -3.650607528 |
| NUP88 | -0.426778058 | 7.842560673 | -2.265072016 | 0.038344753 | 0.366144107 | -3.651072864 |
| PSMB4 | -0.264232019 | 12.12842061 | -2.264721504 | 0.038370801 | 0.366144107 | -3.651622922 |
| lnc-C4orf27-1 | 0.534802255 | 3.683841162 | 2.264702085 | 0.038372245 | 0.366144107 | -3.651653395 |
| ZMAT2 | 0.229190723 | 10.43031424 | 2.264535552 | 0.038384627 | 0.366144107 | -3.651914722 |
| IL13RA1 | 0.445470324 | 11.64026343 | 2.264086137 | 0.03841806 | 0.366144107 | -3.652619909 |
| RMDN1 | -0.337891985 | 8.039241947 | -2.264026299 | 0.038422514 | 0.366144107 | -3.652713798 |
| LOC344887 | 0.551302599 | 1.247120608 | 2.263725158 | 0.038444934 | 0.366144107 | -3.653186286 |
| KRTAP12-1 | 0.906172383 | 1.830846563 | 2.263426584 | 0.038467176 | 0.366144107 | -3.65365472 |
| SPPL2B | 0.235269655 | 4.261270104 | 2.263149808 | 0.038487804 | 0.366144107 | -3.654088931 |
| ARMC3 | 0.744769934 | 1.560162328 | 2.263131016 | 0.038489205 | 0.366144107 | -3.654118411 |
| LINC00342 | -0.513660146 | 8.365300197 | -2.263006448 | 0.038498493 | 0.366144107 | -3.654313827 |
| UCP2 | -0.423223222 | 9.690745914 | -2.262844691 | 0.038510557 | 0.366144107 | -3.654567575 |
| PSMD7 | -0.237607173 | 10.32834978 | -2.262836952 | 0.038511134 | 0.366144107 | -3.654579716 |
| SPAG16 | -0.510713435 | 4.80003173 | -2.262833616 | 0.038511383 | 0.366144107 | -3.65458495 |
| RWDD2A | -0.735446081 | 4.881189674 | -2.262707106 | 0.038520821 | 0.366144107 | -3.654783401 |
| UBTD1 | 0.632195857 | 8.008978806 | 2.26251142 | 0.038535424 | 0.366144107 | -3.655090357 |
| NAMA | 0.677806155 | 1.179149389 | 2.262458876 | 0.038539345 | 0.366144107 | -3.655172775 |
| RETSAT | -0.425417658 | 3.62733286 | -2.262444308 | 0.038540433 | 0.366144107 | -3.655195627 |
| GORAB | -0.504913511 | 4.983264851 | -2.262422325 | 0.038542074 | 0.366144107 | -3.655230108 |
| TTLL1 | -0.318828605 | 6.587651404 | -2.262378654 | 0.038545334 | 0.366144107 | -3.655298607 |
| lnc-GTDC1-4 | 0.621138232 | 3.186899535 | 2.262283883 | 0.03855241 | 0.366144107 | -3.655447258 |
| LOC727993 | 0.252314138 | 4.597209437 | 2.262211092 | 0.038557845 | 0.366144107 | -3.655561432 |
| CANX | -0.191564059 | 7.282292914 | -2.262117906 | 0.038564804 | 0.366144107 | -3.655707591 |
| THEM4 | -0.362364361 | 7.758184175 | -2.262062705 | 0.038568928 | 0.366144107 | -3.655794172 |
| SNORD33 | -0.39245059 | 6.717105888 | -2.261915903 | 0.038579895 | 0.366144107 | -3.65602442 |
| lnc-KLHL25-9 | 0.646695797 | 3.05718841 | 2.261567702 | 0.038605919 | 0.366144107 | -3.656570521 |
| TXNDC11 | -0.372367476 | 7.980109466 | -2.260880108 | 0.038657358 | 0.366144107 | -3.657648802 |
| CHCHD6 | -0.74754484 | 3.163444686 | -2.260838573 | 0.038660467 | 0.366144107 | -3.657713933 |
| TCEAL6 | -0.265116883 | 7.405615149 | -2.260823274 | 0.038661613 | 0.366144107 | -3.657737921 |
| MYO1E | -0.557002201 | 7.12277335 | -2.260593211 | 0.03867884 | 0.366144107 | -3.65809867 |
| ZNF793 | -0.586053578 | 5.804438573 | -2.260578053 | 0.038679975 | 0.366144107 | -3.658122438 |
| lnc-C22orf34-1 | -0.449994714 | 1.427457052 | -2.260498057 | 0.038685967 | 0.366144107 | -3.65824787 |
| lnc-MPP7-1 | -0.534908363 | 4.164623922 | -2.260165056 | 0.038710919 | 0.366144107 | -3.658769991 |
| lnc-HSPH1-3 | 0.395564245 | 1.081771587 | 2.259988931 | 0.038724122 | 0.366144107 | -3.659046127 |
| CLDND2 | -0.859808782 | 3.004972086 | -2.259865885 | 0.038733349 | 0.366144107 | -3.65923904 |
| PAICS | -0.46311711 | 7.049661501 | -2.259691635 | 0.038746418 | 0.366144107 | -3.659512221 |
| CTNNBL1 | -0.25799765 | 10.05271339 | -2.259629445 | 0.038751084 | 0.366144107 | -3.659609719 |
| SERTAD3 | 0.292507912 | 5.441035505 | 2.259627137 | 0.038751257 | 0.366144107 | -3.659613336 |
| TNNI1 | 0.899600564 | 2.338635761 | 2.259606251 | 0.038752824 | 0.366144107 | -3.65964608 |
| XLOC_l2_013116 | -0.417888659 | 1.042308005 | -2.25956343 | 0.038756037 | 0.366144107 | -3.65971321 |
| MIR133A1HG | 0.826701722 | 2.111967331 | 2.259495116 | 0.038761163 | 0.366144107 | -3.659820304 |
| JUND | 0.35068845 | 6.629203649 | 2.259471353 | 0.038762947 | 0.366144107 | -3.659857557 |
| MAPK13 | 0.29649921 | 6.572504778 | 2.259428 | 0.0387662 | 0.366144107 | -3.659925521 |
| SENP5 | -0.229512266 | 9.457089276 | -2.259262934 | 0.038778591 | 0.366144107 | -3.660184283 |
| LOC101927055 | -0.385540511 | 1.020216463 | -2.259077965 | 0.03879248 | 0.366144107 | -3.660474234 |
| LOC100130768 | 0.381344063 | 1.188526742 | 2.259046506 | 0.038794843 | 0.366144107 | -3.660523548 |
| UBOX5 | -0.213561099 | 6.569202359 | -2.258805222 | 0.038812968 | 0.366144107 | -3.66090176 |
| TFB2M | -0.527840132 | 6.777380723 | -2.258760342 | 0.03881634 | 0.366144107 | -3.660972108 |
| AP5B1 | 0.698817611 | 6.323639196 | 2.258496033 | 0.038836206 | 0.366144107 | -3.661386388 |
| MROH6 | 0.4915008 | 5.407380942 | 2.258384249 | 0.038844611 | 0.366144107 | -3.661561593 |
| CBLL1 | -0.241612976 | 7.04192304 | -2.258315338 | 0.038849793 | 0.366144107 | -3.661669599 |
| HERC2P4 | -0.528795279 | 4.96123323 | -2.258109805 | 0.038865253 | 0.366144107 | -3.661991728 |
| PSMB8 | 0.341994754 | 9.051003713 | 2.258008471 | 0.038872877 | 0.366144107 | -3.662150542 |
| lnc-TLE3-6 | 0.466515275 | 3.652477901 | 2.257896129 | 0.038881331 | 0.366144107 | -3.662326605 |
| lnc-C4orf42-3 | 0.467160218 | 4.130967 | 2.257663762 | 0.038898823 | 0.366144107 | -3.66269076 |
| XLOC_l2_010225 | -0.28422439 | 0.926841594 | -2.257108703 | 0.038940636 | 0.366144107 | -3.663560555 |
| TREX2 | 0.421200427 | 3.953081455 | 2.256974771 | 0.038950731 | 0.366144107 | -3.663770418 |
| SP110 | 0.426652552 | 10.27901382 | 2.256685017 | 0.038972581 | 0.366144107 | -3.664224423 |
| RHOG | 0.501629327 | 9.21503665 | 2.256633042 | 0.038976501 | 0.366144107 | -3.664305858 |
| SPATS1 | -0.25974035 | 0.790543634 | -2.25652206 | 0.038984874 | 0.366144107 | -3.664479744 |
| TPST1 | 1.212767898 | 6.504627933 | 2.256205391 | 0.039008773 | 0.366144107 | -3.664975876 |
| lnc-OSCAR-1 | 0.855266176 | 2.624460121 | 2.256035382 | 0.039021609 | 0.366144107 | -3.66524222 |
| SLC25A5 | -0.385412238 | 11.20260676 | -2.256017462 | 0.039022962 | 0.366144107 | -3.665270294 |
| NACA2 | -0.40088614 | 11.89318111 | -2.255686639 | 0.039047952 | 0.366144107 | -3.665788551 |
| S100A16 | 0.728851858 | 1.713959547 | 2.25558396 | 0.039055711 | 0.366144107 | -3.665949398 |
| lnc-AC010536.1-1 | 0.989851305 | 7.374506113 | 2.255518894 | 0.039060629 | 0.366144107 | -3.666051323 |
| SNORA21 | 0.48258927 | 6.761819743 | 2.255513717 | 0.03906102 | 0.366144107 | -3.666059432 |
| KIAA0101 | -0.980245014 | 6.083837867 | -2.255511556 | 0.039061184 | 0.366144107 | -3.666062817 |
| ALG3 | -0.234405936 | 9.035522755 | -2.255471929 | 0.039064179 | 0.366144107 | -3.666124891 |
| lnc-C5orf43-3 | 0.427515907 | 5.670894468 | 2.25537205 | 0.03907173 | 0.366144107 | -3.666281345 |
| ZBTB38 | -0.424476292 | 4.278810103 | -2.255341308 | 0.039074054 | 0.366144107 | -3.666329501 |
| BTN2A3P | 0.417644991 | 6.568209978 | 2.255103339 | 0.039092051 | 0.366144107 | -3.66670225 |
| VNN2 | 0.522116462 | 13.44253877 | 2.255067619 | 0.039094753 | 0.366144107 | -3.666758199 |
| THBS1 | 0.659940535 | 6.007725901 | 2.255039255 | 0.039096898 | 0.366144107 | -3.666802626 |
| DYRK4 | -0.350927761 | 6.608567893 | -2.254919124 | 0.039105987 | 0.366144107 | -3.666990788 |
| MRPS26 | -0.286519349 | 8.566054848 | -2.254834761 | 0.039112371 | 0.366144107 | -3.667122923 |
| CHST15 | 0.669974083 | 10.67883219 | 2.254769545 | 0.039117307 | 0.366144107 | -3.667225067 |
| ZNF449 | -0.508987346 | 4.683143653 | -2.254728214 | 0.039120435 | 0.366144107 | -3.667289802 |
| RNF220 | -0.304843791 | 10.90892133 | -2.25469777 | 0.03912274 | 0.366144107 | -3.667337484 |
| LILRA5 | 0.824990929 | 7.287919323 | 2.254578102 | 0.039131799 | 0.366144107 | -3.667524907 |
| MYBL1 | -0.568844399 | 8.962422551 | -2.254536022 | 0.039134985 | 0.366144107 | -3.667590812 |
| HADH | -0.374507192 | 8.476231366 | -2.254094476 | 0.039168432 | 0.366144107 | -3.668282318 |
| XPO4 | -0.516730858 | 5.510361364 | -2.254050238 | 0.039171785 | 0.366144107 | -3.668351597 |
| lnc-DDX19A-1 | 0.259514435 | 5.242409138 | 2.254038793 | 0.039172652 | 0.366144107 | -3.668369519 |
| PCNA | -0.426459852 | 8.097831143 | -2.253991191 | 0.03917626 | 0.366144107 | -3.668444064 |
| MAN2A2 | 0.535442724 | 8.600789455 | 2.253742751 | 0.039195094 | 0.366221582 | -3.668833113 |
| LOC100499489 | -0.367746768 | 6.632523256 | -2.253209543 | 0.039235545 | 0.36650094 | -3.669668034 |
| MALT1 | -0.36353563 | 3.993708646 | -2.252562958 | 0.03928465 | 0.366785194 | -3.670680371 |
| lnc-HAPLN1-2 | -0.607809245 | 1.568394458 | -2.252530743 | 0.039287098 | 0.366785194 | -3.670730805 |
| lnc-TNFRSF13B-4 | 0.895591908 | 2.15894478 | 2.25237514 | 0.039298925 | 0.366797006 | -3.670974407 |
| lnc-C11orf36-2 | 0.78246898 | 4.053797416 | 2.252146681 | 0.039316295 | 0.366860536 | -3.671332054 |
| DNAJB4 | -0.689565134 | 3.347322129 | -2.251969976 | 0.039329735 | 0.36688737 | -3.67160867 |
| LOC400541 | 0.711218879 | 1.698775718 | 2.251398737 | 0.039373211 | 0.366966406 | -3.672502831 |
| lnc-HDDC3-2 | 0.623876049 | 2.324455103 | 2.251377696 | 0.039374814 | 0.366966406 | -3.672535764 |
| PAFAH2 | -0.401927607 | 5.842497677 | -2.251314063 | 0.03937966 | 0.366966406 | -3.672635363 |
| CDCA2 | -1.051807248 | 3.71612349 | -2.251303396 | 0.039380472 | 0.366966406 | -3.672652059 |
| ZNF766 | -0.290491622 | 9.035471327 | -2.250858116 | 0.039414399 | 0.367041065 | -3.673348974 |
| GSDMA | 0.710870907 | 1.481926429 | 2.250798627 | 0.039418934 | 0.367041065 | -3.673442076 |
| IL7R | -0.505131081 | 13.38418235 | -2.250782159 | 0.039420189 | 0.367041065 | -3.673467849 |
| PPP2R2B | -0.637560536 | 8.002657864 | -2.250501534 | 0.039441588 | 0.367141876 | -3.673907021 |
| LMLN-AS1 | -0.944442378 | 1.506544903 | -2.250228078 | 0.03946245 | 0.367237643 | -3.674334951 |
| ATXN7L3B | -0.342608522 | 7.617592555 | -2.249869124 | 0.03948985 | 0.367321729 | -3.674896644 |
| RHOBTB2 | -0.320269423 | 5.715145479 | -2.249576419 | 0.039512207 | 0.367321729 | -3.675354638 |
| PIK3IP1 | -0.533251791 | 6.655043959 | -2.249388105 | 0.039526597 | 0.367321729 | -3.675649281 |
| EIF3C | -0.410353158 | 13.35242475 | -2.249275854 | 0.039535176 | 0.367321729 | -3.675824906 |
| LOC100996535 | 0.781653277 | 2.946255031 | 2.249269524 | 0.03953566 | 0.367321729 | -3.67583481 |
| ERBB2 | -0.632006459 | 6.303402267 | -2.249075386 | 0.039550503 | 0.367321729 | -3.676138545 |
| B3GAT1 | -1.06786576 | 7.105414733 | -2.249061964 | 0.039551529 | 0.367321729 | -3.676159544 |
| ZNF268 | -0.438579307 | 8.465487359 | -2.249002232 | 0.039556097 | 0.367321729 | -3.676252994 |
| lnc-MAGEA8-2 | 0.626016298 | 3.325396584 | 2.248527365 | 0.03959243 | 0.367455739 | -3.67699588 |
| LPXN | -0.263456365 | 8.486102853 | -2.24846619 | 0.039597113 | 0.367455739 | -3.677091578 |
| YWHAH | 0.242622032 | 8.212516534 | 2.248398832 | 0.03960227 | 0.367455739 | -3.677196947 |
| REN | 1.319788495 | 2.678997447 | 2.248157955 | 0.039620716 | 0.367528702 | -3.677573741 |
| DNAJB11 | -0.374533741 | 8.910607033 | -2.247742621 | 0.03965254 | 0.367587199 | -3.678223388 |
| LOC256880 | -0.789646844 | 2.518716379 | -2.247636695 | 0.03966066 | 0.367587199 | -3.678389064 |
| LAIR1 | -0.352940906 | 10.60326376 | -2.247623404 | 0.039661679 | 0.367587199 | -3.678409852 |
| ASUN | -0.420205236 | 7.062899211 | -2.247523254 | 0.039669359 | 0.367587199 | -3.678566492 |
| UNC5A | 0.776040324 | 1.891523485 | 2.247312234 | 0.039685543 | 0.367639081 | -3.678896525 |
| lnc-EIF4E1B-1 | 0.832117301 | 3.847521047 | 2.247142779 | 0.039698544 | 0.367661453 | -3.679161543 |
| lnc-FAIM3-2 | -0.898392579 | 3.62794742 | -2.246882048 | 0.039718556 | 0.367748724 | -3.679569292 |
| PCSK4 | -0.38324088 | 4.878664207 | -2.246455345 | 0.039751328 | 0.367950091 | -3.680236556 |
| LINGO3 | -0.625160962 | 3.972920736 | -2.246235876 | 0.039768193 | 0.367950091 | -3.680579732 |
| TIGD3 | 0.621937709 | 5.599123042 | 2.245904567 | 0.039793665 | 0.367950091 | -3.681097762 |
| SKP1 | -0.278549661 | 11.7026407 | -2.245824349 | 0.039799835 | 0.367950091 | -3.681223184 |
| lnc-C6orf192-1 | -0.810954801 | 1.970677139 | -2.245818135 | 0.039800313 | 0.367950091 | -3.6812329 |
| LPA | -0.559830423 | 5.891000863 | -2.245771859 | 0.039803873 | 0.367950091 | -3.681305251 |
| IGSF22 | -0.753250097 | 3.543473243 | -2.245439137 | 0.039829475 | 0.367959749 | -3.681825443 |
| OXA1L | -0.28375745 | 8.635812551 | -2.245376122 | 0.039834326 | 0.367959749 | -3.681923959 |
| SPECC1L | -0.256669804 | 8.785828185 | -2.245345252 | 0.039836702 | 0.367959749 | -3.681972219 |
| C19orf24 | -0.222966801 | 10.63954714 | -2.244986009 | 0.039864367 | 0.368117379 | -3.682533825 |
| lnc-ATP6V1G3-2 | 0.573308608 | 3.897832424 | 2.244761084 | 0.039881697 | 0.368172218 | -3.682885432 |
| ZFP36L2 | -0.255681452 | 11.55710863 | -2.244633801 | 0.039891508 | 0.368172218 | -3.683084395 |
| KIAA1107 | -0.674030113 | 1.61589531 | -2.244479876 | 0.039903374 | 0.368183896 | -3.683324997 |
| NT5C | -0.348097411 | 10.95956864 | -2.243971057 | 0.039942624 | 0.368377145 | -3.68412029 |
| ZNF71 | -0.487241085 | 5.055685717 | -2.243872044 | 0.039950266 | 0.368377145 | -3.684275039 |
| LOC101929129 | 0.625106495 | 4.024358397 | 2.243636878 | 0.039968422 | 0.368377145 | -3.684642574 |
| MXD3 | 0.485072172 | 9.666338406 | 2.243616693 | 0.039969981 | 0.368377145 | -3.684674119 |
| lnc-AL137145.1-1 | 1.127874013 | 1.925027505 | 2.243396461 | 0.039986992 | 0.368377145 | -3.685018296 |
| SLC20A1 | -0.327085378 | 10.90575694 | -2.243383932 | 0.03998796 | 0.368377145 | -3.685037876 |
| ZNF615 | -0.302167926 | 6.349377022 | -2.242875016 | 0.040027295 | 0.368624461 | -3.685833148 |
| SOCS3 | 0.963993746 | 6.658968409 | 2.242680578 | 0.040042333 | 0.368624461 | -3.686136971 |
| DOLK | -0.337397117 | 6.240948113 | -2.242235417 | 0.040076782 | 0.368624461 | -3.686832521 |
| lnc-RP11-1105G2.3.1-3 | 0.772848385 | 4.236121336 | 2.242221622 | 0.04007785 | 0.368624461 | -3.686854074 |
| FAM83G | -0.440615371 | 7.711717833 | -2.242189833 | 0.040080311 | 0.368624461 | -3.686903742 |
| lnc-LALBA-1 | 0.88870199 | 2.325915277 | 2.242181802 | 0.040080933 | 0.368624461 | -3.686916289 |
| CHMP3 | 0.344260785 | 8.13135516 | 2.24207627 | 0.040089104 | 0.368624461 | -3.687081169 |
| SPON2 | -0.874489039 | 12.91226895 | -2.241745469 | 0.040114729 | 0.368688057 | -3.68759798 |
| NCR3LG1 | -0.410383722 | 4.26870581 | -2.241701068 | 0.040118169 | 0.368688057 | -3.687667346 |
| WDR43 | -0.540300287 | 6.194459335 | -2.241575922 | 0.040127868 | 0.368688057 | -3.687862851 |
| CCT6A | -0.388386627 | 9.673675175 | -2.241037973 | 0.040169584 | 0.368766402 | -3.68870319 |
| ICE2 | -0.586564386 | 4.152981008 | -2.240899062 | 0.040180362 | 0.368766402 | -3.68892017 |
| RAB11FIP3 | -0.33211492 | 7.357611083 | -2.240726498 | 0.040193756 | 0.368766402 | -3.689189708 |
| RAD54L2 | -0.239165335 | 8.336362778 | -2.240582348 | 0.040204947 | 0.368766402 | -3.689414859 |
| TTC37 | -0.340659485 | 8.25198474 | -2.240368044 | 0.04022159 | 0.368766402 | -3.689749571 |
| ALPK3 | 0.778724184 | 3.653936641 | 2.240310301 | 0.040226076 | 0.368766402 | -3.689839756 |
| ZNF273 | 0.430069425 | 6.673912413 | 2.240264472 | 0.040229636 | 0.368766402 | -3.689911331 |
| MET | -0.984123184 | 1.573489769 | -2.240235065 | 0.040231921 | 0.368766402 | -3.689957259 |
| ARPC1A | 0.142296978 | 8.509371422 | 2.239880529 | 0.040259476 | 0.368766402 | -3.690510948 |
| GMNN | -0.431473745 | 6.588839116 | -2.239815049 | 0.040264567 | 0.368766402 | -3.690613206 |
| GNB1L | -0.192615221 | 7.851593591 | -2.2397002 | 0.040273498 | 0.368766402 | -3.690792559 |
| TK1 | -0.803096504 | 8.696278402 | -2.23953926 | 0.040286016 | 0.368766402 | -3.691043882 |
| XLOC_l2_015830 | 0.499936315 | 2.043076349 | 2.239528796 | 0.04028683 | 0.368766402 | -3.691060223 |
| lnc-AC008394.1-1 | -0.676669454 | 1.09616572 | -2.239513645 | 0.040288009 | 0.368766402 | -3.691083881 |
| MCPH1 | -0.411518043 | 5.67599434 | -2.239309667 | 0.040303881 | 0.368766402 | -3.691402399 |
| AKT3 | -0.427687839 | 6.879029259 | -2.239267276 | 0.04030718 | 0.368766402 | -3.691468592 |
| ZBTB49 | -0.430668512 | 4.052410956 | -2.238599381 | 0.040359194 | 0.368766402 | -3.692511431 |
| STAC2 | 0.637958981 | 2.287326771 | 2.238489323 | 0.040367771 | 0.368766402 | -3.692683261 |
| NDUFB11 | -0.313805832 | 10.8426858 | -2.23842108 | 0.04037309 | 0.368766402 | -3.692789805 |
| CXCR2P1 | 0.80855443 | 7.802437578 | 2.238413807 | 0.040373657 | 0.368766402 | -3.692801159 |
| IGSF8 | -0.306939963 | 5.77087786 | -2.238356969 | 0.040378088 | 0.368766402 | -3.692889895 |
| USP40 | -0.600484758 | 3.852402829 | -2.238344766 | 0.040379039 | 0.368766402 | -3.692908947 |
| TLR3 | -0.673151074 | 3.747249903 | -2.23797324 | 0.040408013 | 0.368766402 | -3.693488951 |
| NDUFA3 | -0.241406244 | 11.47673104 | -2.237948093 | 0.040409975 | 0.368766402 | -3.693528208 |
| PATE3 | 0.460511992 | 4.617830334 | 2.237864242 | 0.040416517 | 0.368766402 | -3.693659104 |
| UBTD2 | -0.328785964 | 5.113100986 | -2.237863036 | 0.040416611 | 0.368766402 | -3.693660988 |
| ALG2 | -0.376229098 | 6.772298681 | -2.237577333 | 0.04043891 | 0.368766402 | -3.694106971 |
| lnc-CACNA1B-1 | 0.625753153 | 4.579705739 | 2.237511239 | 0.04044407 | 0.368766402 | -3.694210141 |
| PPBP | 0.920583233 | 10.81859902 | 2.23750804 | 0.04044432 | 0.368766402 | -3.694215136 |
| BFSP1 | -0.785086904 | 6.216876032 | -2.237247721 | 0.04046465 | 0.368787065 | -3.694621467 |
| RETN | 1.593415915 | 9.393586874 | 2.237113455 | 0.040475139 | 0.368787065 | -3.694831034 |
| PICALM | 0.313875791 | 12.45668539 | 2.237071182 | 0.040478442 | 0.368787065 | -3.694897015 |
| UNK | -0.242552925 | 8.338955515 | -2.236581606 | 0.040516714 | 0.369038938 | -3.695661111 |
| CHAMP1 | -0.330266373 | 6.848406474 | -2.236404161 | 0.040530594 | 0.369068567 | -3.695938036 |
| XLOC_l2_012082 | -0.545015182 | 3.956274317 | -2.236225374 | 0.040544583 | 0.369099177 | -3.696217045 |
| lnc-GPR137B-1 | 0.85308127 | 2.091023807 | 2.235324213 | 0.040615163 | 0.369628653 | -3.697623221 |
| LOC100506411 | -0.860382477 | 3.843831854 | -2.23510744 | 0.040632158 | 0.369628653 | -3.697961436 |
| DENND1C | -0.213192413 | 9.627954524 | -2.235020355 | 0.040638987 | 0.369628653 | -3.698097303 |
| THOP1 | -0.228785511 | 9.127647368 | -2.234834864 | 0.040653537 | 0.369628653 | -3.698386696 |
| PRO2852 | 0.479645112 | 8.37486358 | 2.234803997 | 0.040655959 | 0.369628653 | -3.698434851 |
| lnc-PDE4D-1 | -0.894685701 | 2.059870431 | -2.234575985 | 0.040673852 | 0.369652426 | -3.698790566 |
| NID2 | 0.533777767 | 3.001421801 | 2.234393437 | 0.040688183 | 0.369652426 | -3.699075342 |
| LOC729083 | 0.565603342 | 6.140943165 | 2.234363865 | 0.040690505 | 0.369652426 | -3.699121473 |
| lnc-RP11-1070N10.3.1-2 | 0.987446672 | 2.13403615 | 2.233396811 | 0.040766504 | 0.370175104 | -3.700629897 |
| EME1 | -0.682843624 | 3.136431398 | -2.233360536 | 0.040769357 | 0.370175104 | -3.700686474 |
| LOC102723979 | 0.548675984 | 1.753957249 | 2.233219065 | 0.040780487 | 0.370179381 | -3.700907115 |
| S1PR2 | -0.452025917 | 7.151479267 | -2.233008833 | 0.040797032 | 0.370214651 | -3.701234989 |
| lnc-ARID1B-2 | 0.725587638 | 1.575594112 | 2.232898815 | 0.040805692 | 0.370214651 | -3.701406565 |
| SVIL | 0.467035046 | 7.278996557 | 2.23261156 | 0.040828313 | 0.37026252 | -3.701854528 |
| lnc-ZNF841-1 | -0.406417534 | 5.724674773 | -2.232561059 | 0.040832291 | 0.37026252 | -3.70193328 |
| lnc-C2orf27B-3 | -0.438771995 | 7.269329406 | -2.232300579 | 0.040852815 | 0.370351933 | -3.702339463 |
| MYO5C | -0.683964051 | 2.585418033 | -2.231821655 | 0.040890576 | 0.370432725 | -3.703086224 |
| APCDD1L-AS1 | 0.762789654 | 2.153177547 | 2.231724414 | 0.040898247 | 0.370432725 | -3.703237838 |
| ZNF530 | -0.508129668 | 4.379379009 | -2.231335032 | 0.040928977 | 0.370432725 | -3.703844917 |
| PIK3CD | 0.353128167 | 9.932711789 | 2.231252613 | 0.040935484 | 0.370432725 | -3.703973408 |
| PHF6 | -0.313556156 | 5.918586006 | -2.231163377 | 0.040942531 | 0.370432725 | -3.704112525 |
| lnc-ZNF345-1 | -0.809094623 | 3.163871413 | -2.231093661 | 0.040948037 | 0.370432725 | -3.704221209 |
| MTPAP | -0.188221292 | 6.355164041 | -2.230887164 | 0.04096435 | 0.370432725 | -3.704543121 |
| ADH5 | -0.515648731 | 6.007734023 | -2.230756622 | 0.040974665 | 0.370432725 | -3.704746618 |
| SFT2D1 | 0.218654514 | 9.110954685 | 2.230569315 | 0.040989471 | 0.370432725 | -3.705038593 |
| SECTM1 | 0.59206403 | 11.51298782 | 2.230542983 | 0.040991553 | 0.370432725 | -3.705079639 |
| NAA16 | -0.395396877 | 7.454428256 | -2.230432818 | 0.041000263 | 0.370432725 | -3.705251359 |
| DZIP1L | 0.899597909 | 1.756999229 | 2.230295515 | 0.041011122 | 0.370432725 | -3.705465376 |
| HORMAD1 | 0.684288782 | 3.888399292 | 2.230265058 | 0.041013531 | 0.370432725 | -3.70551285 |
| JMJD1C | 0.380618625 | 7.160597944 | 2.230188038 | 0.041019624 | 0.370432725 | -3.705632899 |
| WEE1 | -0.529504093 | 4.885003453 | -2.230060421 | 0.041029721 | 0.370432725 | -3.705831808 |
| lnc-STK39-1 | -0.825426546 | 1.424094597 | -2.230026767 | 0.041032384 | 0.370432725 | -3.705884262 |
| lnc-PCDH18-3 | 0.432670874 | 1.231888408 | 2.229824022 | 0.041048431 | 0.37048129 | -3.706200257 |
| CCDC50 | -0.384550506 | 6.358149479 | -2.229144549 | 0.041102253 | 0.370817336 | -3.707259176 |
| LOC728975 | 0.747780634 | 6.365368979 | 2.229024544 | 0.041111765 | 0.370817336 | -3.707446182 |
| PARP15 | -0.323699908 | 5.87158573 | -2.228821753 | 0.041127845 | 0.370817336 | -3.707762185 |
| MTF2 | -0.191717497 | 8.400015691 | -2.228784063 | 0.041130834 | 0.370817336 | -3.707820915 |
| QKI | 0.533654562 | 7.829882866 | 2.228587999 | 0.041146387 | 0.370817336 | -3.70812642 |
| LIX1L | -0.325409134 | 6.719311272 | -2.228545899 | 0.041149727 | 0.370817336 | -3.708192019 |
| LINC01197 | 0.526754995 | 4.214859094 | 2.228166372 | 0.041179851 | 0.370830369 | -3.708783356 |
| TPGS1 | -0.493916066 | 4.344244478 | -2.228155126 | 0.041180743 | 0.370830369 | -3.708800877 |
| XLOC_l2_013415 | 0.702509074 | 3.02864686 | 2.228096499 | 0.041185399 | 0.370830369 | -3.708892219 |
| RAC1 | 0.201428706 | 8.854089961 | 2.227989664 | 0.041193883 | 0.370830369 | -3.709058667 |
| OR1L6 | 0.362649905 | 4.990014415 | 2.227649426 | 0.041220914 | 0.370892785 | -3.709588732 |
| lnc-EXOSC6-3 | -0.92124389 | 3.507755088 | -2.22763356 | 0.041222175 | 0.370892785 | -3.709613448 |
| PSMC4 | -0.236782166 | 8.096487184 | -2.22733225 | 0.041246128 | 0.371012182 | -3.710082833 |
| RFC4 | -0.466626777 | 7.037852535 | -2.226732779 | 0.041293822 | 0.371345016 | -3.711016615 |
| UVRAG | -0.310487283 | 6.377224917 | -2.226580782 | 0.041305924 | 0.371355255 | -3.711253359 |
| XRCC4 | 0.323365714 | 6.494883305 | 2.22629674 | 0.041328546 | 0.371355255 | -3.71169575 |
| lnc-TMEM242-2 | 0.672694721 | 1.580888336 | 2.226290673 | 0.041329029 | 0.371355255 | -3.7117052 |
| LOC102724792 | -0.746310865 | 3.008839373 | -2.226181452 | 0.041337731 | 0.371355255 | -3.711875304 |
| LINC01424 | -0.440040389 | 1.138117959 | -2.225867439 | 0.04136276 | 0.371484004 | -3.712364335 |
| CPNE6 | 1.064393869 | 2.47821805 | 2.225692356 | 0.041376721 | 0.371513316 | -3.712636988 |
| ZRANB2-AS1 | -0.666840769 | 2.480652498 | -2.225415681 | 0.041398791 | 0.371615412 | -3.71306783 |
| MAGEH1 | -0.541252429 | 8.233921609 | -2.224748913 | 0.041452026 | 0.37185744 | -3.714106029 |
| TIMM9 | -0.412472936 | 9.703268214 | -2.224704307 | 0.041455589 | 0.37185744 | -3.714175478 |
| XLOC_l2_007315 | -0.732644477 | 1.45341584 | -2.224675693 | 0.041457875 | 0.37185744 | -3.714220029 |
| lnc-COL6A3-5 | -0.555491292 | 3.336317316 | -2.224380594 | 0.041481459 | 0.371960636 | -3.714679465 |
| lnc-UBE2B-1 | 0.799868238 | 3.927452346 | 2.224246623 | 0.04149217 | 0.371960636 | -3.714888034 |
| MAB21L3 | 0.621030875 | 3.061658468 | 2.224031006 | 0.041509414 | 0.371960636 | -3.715223699 |
| ESYT1 | -0.408108246 | 8.090789227 | -2.223798755 | 0.041527995 | 0.371960636 | -3.715585243 |
| lnc-ARMC10-1 | 0.705787974 | 1.427659509 | 2.223742448 | 0.041532501 | 0.371960636 | -3.715672894 |
| PTPRM | -1.206172982 | 3.504078581 | -2.223728211 | 0.041533641 | 0.371960636 | -3.715695055 |
| TAF1A | -0.385330694 | 5.207725627 | -2.223414126 | 0.041558785 | 0.371969259 | -3.716183956 |
| GNG5 | 0.258007739 | 9.995565771 | 2.223256396 | 0.041571418 | 0.371969259 | -3.716429465 |
| SMIM12 | -0.247034886 | 7.366791884 | -2.223068547 | 0.041586467 | 0.371969259 | -3.716721845 |
| TP53RK | -0.358908786 | 7.650695831 | -2.223063341 | 0.041586884 | 0.371969259 | -3.716729948 |
| LOC101927221 | 0.545649307 | 8.306049283 | 2.223047477 | 0.041588155 | 0.371969259 | -3.716754638 |
| LOC440910 | 0.721592471 | 1.457032084 | 2.222844546 | 0.041604419 | 0.37201892 | -3.717070479 |
| LOC101928324 | 0.467482976 | 4.582366452 | 2.222004042 | 0.041671845 | 0.372125863 | -3.718378493 |
| KIF3C | 0.524274761 | 4.985666286 | 2.221777659 | 0.041690023 | 0.372125863 | -3.718730757 |
| CASC5 | -0.802744939 | 4.054467673 | -2.221678025 | 0.041698025 | 0.372125863 | -3.718885789 |
| SPRED2 | -0.329784194 | 0.781612121 | -2.221645634 | 0.041700627 | 0.372125863 | -3.718936189 |
| RAB11FIP1 | 0.407073715 | 10.64491553 | 2.221502581 | 0.041712121 | 0.372125863 | -3.719158773 |
| KATNAL2 | -0.610968454 | 2.44030373 | -2.221459947 | 0.041715546 | 0.372125863 | -3.71922511 |
| UBE2D3 | 0.13805809 | 11.09727808 | 2.221239901 | 0.041733233 | 0.372125863 | -3.719567476 |
| TOMM20L | 0.405039947 | 8.579761481 | 2.221219504 | 0.041734872 | 0.372125863 | -3.71959921 |
| lnc-SSTR4-4 | 1.073294627 | 2.669614203 | 2.221104337 | 0.041744132 | 0.372125863 | -3.71977839 |
| SPATA31C2 | -0.406692351 | 0.867623549 | -2.221048003 | 0.041748662 | 0.372125863 | -3.719866035 |
| KRTAP20-3 | 0.691333773 | 1.18597224 | 2.221013497 | 0.041751437 | 0.372125863 | -3.719919719 |
| OR6C74 | -0.386886791 | 0.889923096 | -2.220990288 | 0.041753304 | 0.372125863 | -3.719955827 |
| FCHSD1 | 0.34937636 | 4.501209121 | 2.220871335 | 0.041762872 | 0.372125863 | -3.720140888 |
| KRTAP10-1 | 0.276137826 | 6.548327417 | 2.220765811 | 0.041771361 | 0.372125863 | -3.720305054 |
| CATIP-AS1 | 0.804437482 | 5.927070261 | 2.220650354 | 0.041780652 | 0.372125863 | -3.72048467 |
| EIF2S1 | -0.405179768 | 6.5963448 | -2.220450104 | 0.04179677 | 0.372125863 | -3.720796184 |
| GTPBP4 | -0.424987441 | 8.774345493 | -2.220428225 | 0.041798531 | 0.372125863 | -3.72083022 |
| ANXA2R | -0.385220085 | 10.47425766 | -2.219770252 | 0.041851534 | 0.372141 | -3.721853692 |
| MAPK14 | 0.699824967 | 7.227602561 | 2.219768516 | 0.041851674 | 0.372141 | -3.721856392 |
| lnc-VKORC1L1-1 | 0.492222719 | 7.258802789 | 2.219370819 | 0.041883741 | 0.372141 | -3.722474941 |
| C2orf40 | -0.686181463 | 5.461974075 | -2.219351506 | 0.041885299 | 0.372141 | -3.722504977 |
| QPCTL | -0.547999203 | 3.433197929 | -2.219344811 | 0.041885839 | 0.372141 | -3.722515389 |
| PSMD11 | -0.297233669 | 7.902140053 | -2.219204388 | 0.041897168 | 0.372141 | -3.722733781 |
| BID | 0.230888356 | 11.26075164 | 2.219038025 | 0.041910592 | 0.372141 | -3.722992505 |
| GPR135 | -0.260143753 | 5.09788714 | -2.219037061 | 0.04191067 | 0.372141 | -3.722994005 |
| HIST1H2BI | 0.504083665 | 7.332421779 | 2.218998543 | 0.041913779 | 0.372141 | -3.723053905 |
| RPL22 | -0.5028334 | 11.89596229 | -2.218973157 | 0.041915828 | 0.372141 | -3.723093384 |
| CUL4A | -0.364466884 | 8.867768091 | -2.218672121 | 0.041940134 | 0.372141 | -3.723561522 |
| lnc-DLGAP1-1 | -0.541576911 | 0.955354065 | -2.218635445 | 0.041943096 | 0.372141 | -3.723618554 |
| TIMM8A | -0.387353301 | 5.881808585 | -2.218265841 | 0.041972957 | 0.372141 | -3.724193277 |
| SAP25 | 0.4275984 | 12.58063086 | 2.218130578 | 0.04198389 | 0.372141 | -3.724403597 |
| CLIC3 | -0.776325231 | 9.321039034 | -2.218041168 | 0.041991119 | 0.372141 | -3.724542616 |
| lnc-C1orf124-3 | -0.321927143 | 0.894254732 | -2.217739798 | 0.042015492 | 0.372141 | -3.725011184 |
| lnc-AAAS-1 | -0.642841616 | 2.492983384 | -2.217733364 | 0.042016012 | 0.372141 | -3.725021188 |
| RBM19 | -0.368955589 | 7.446953479 | -2.217454706 | 0.042038561 | 0.372141 | -3.725454417 |
| XLOC_l2_000945 | 0.665189399 | 1.025034133 | 2.217415546 | 0.04204173 | 0.372141 | -3.725515298 |
| DANCR | -0.43952145 | 8.259419911 | -2.217296241 | 0.042051388 | 0.372141 | -3.725700772 |
| SLC35F3 | -1.072852268 | 4.645525693 | -2.217080098 | 0.042068891 | 0.372141 | -3.726036781 |
| C9orf66 | 0.679563936 | 2.888509737 | 2.21661407 | 0.042106651 | 0.372141 | -3.726761205 |
| EZH2 | -0.41482724 | 6.190347405 | -2.216566907 | 0.042110474 | 0.372141 | -3.726834514 |
| EXT1 | 0.519448736 | 4.103781572 | 2.216525463 | 0.042113834 | 0.372141 | -3.726898933 |
| ZBTB45 | -0.151321074 | 9.214232919 | -2.216387921 | 0.042124986 | 0.372141 | -3.727112721 |
| lnc-SLC7A7-1 | -0.702990669 | 1.781632982 | -2.216337369 | 0.042129085 | 0.372141 | -3.727191293 |
| TMED6 | 0.635142811 | 3.816249298 | 2.216274641 | 0.042134173 | 0.372141 | -3.727288791 |
| TP53TG3C | 0.5667236 | 3.661175159 | 2.216262863 | 0.042135128 | 0.372141 | -3.727307098 |
| HEXA | -0.178001428 | 7.805745991 | -2.216093347 | 0.04214888 | 0.372141 | -3.727570569 |
| lnc-AUH-2 | -0.884377541 | 3.291783601 | -2.216087541 | 0.042149351 | 0.372141 | -3.727579591 |
| SLC15A3 | 0.378716155 | 9.522017365 | 2.215986019 | 0.042157589 | 0.372141 | -3.727737378 |
| lnc-ACRC-1 | -0.83276771 | 6.128767973 | -2.215948427 | 0.042160639 | 0.372141 | -3.727795802 |
| USP47 | -0.276080202 | 7.768531237 | -2.215849904 | 0.042168636 | 0.372141 | -3.727948924 |
| KRTAP4-1 | 0.791000547 | 2.569018517 | 2.215833034 | 0.042170005 | 0.372141 | -3.727975142 |
| DCAF4L1 | 0.779599703 | 1.267238507 | 2.215768232 | 0.042175266 | 0.372141 | -3.728075853 |
| MATK | -0.499968965 | 7.816475565 | -2.215422004 | 0.042203382 | 0.372294501 | -3.728613913 |
| lnc-ELTD1-6 | 0.487416357 | 10.06021526 | 2.215105401 | 0.042229107 | 0.372348703 | -3.729105902 |
| TPRG1-AS1 | 0.585208006 | 5.790280403 | 2.214889211 | 0.042246682 | 0.372348703 | -3.729441834 |
| LOC101927440 | 1.096786432 | 2.241229627 | 2.214841897 | 0.04225053 | 0.372348703 | -3.729515352 |
| KIAA1147 | -0.482287448 | 8.119350706 | -2.214771929 | 0.04225622 | 0.372348703 | -3.72962407 |
| CRTC3 | -0.410711842 | 6.649223218 | -2.214596683 | 0.042270474 | 0.372348703 | -3.729896363 |
| C9orf84 | 0.590925484 | 1.264264546 | 2.214555142 | 0.042273854 | 0.372348703 | -3.729960908 |
| SIRT4 | -0.547389932 | 3.93835466 | -2.213792096 | 0.042335976 | 0.372723728 | -3.731146387 |
| MECR | -0.416613141 | 5.081701749 | -2.213626338 | 0.042349482 | 0.372723728 | -3.731403886 |
| TP73-AS1 | -0.402049428 | 6.125958882 | -2.213409253 | 0.042367177 | 0.372723728 | -3.731741107 |
| WNT2B | 0.521306544 | 0.873164171 | 2.213391801 | 0.0423686 | 0.372723728 | -3.731768216 |
| FAM58A | -0.324840407 | 9.613650631 | -2.213245798 | 0.042380504 | 0.372723728 | -3.731995009 |
| G0S2 | 0.894037578 | 5.258572063 | 2.213241885 | 0.042380824 | 0.372723728 | -3.732001086 |
| TNFAIP2 | 0.367121644 | 13.46628452 | 2.212967336 | 0.042403218 | 0.372740432 | -3.732427536 |
| SRSF8 | -0.463839866 | 8.362287721 | -2.212897588 | 0.042408909 | 0.372740432 | -3.73253587 |
| LOC388780 | -1.450643019 | 2.552625359 | -2.212708275 | 0.04242436 | 0.372740432 | -3.732829907 |
| lnc-AL445989.1-4 | -0.425906857 | 0.982761353 | -2.212692435 | 0.042425653 | 0.372740432 | -3.732854509 |
| E2F5 | -0.779561125 | 4.403289054 | -2.212134788 | 0.042471196 | 0.373027426 | -3.733720564 |
| lnc-PROK1-1 | 0.590434412 | 4.114510145 | 2.212029498 | 0.0424798 | 0.373027426 | -3.733884074 |
| FA2H | -1.135324175 | 3.632208348 | -2.211576809 | 0.042516812 | 0.373169783 | -3.734587037 |
| MROH8 | -0.501812981 | 4.174221182 | -2.211244541 | 0.042543997 | 0.373169783 | -3.735102961 |
| ZNF32 | -0.448372595 | 4.802798223 | -2.21101632 | 0.042562679 | 0.373169783 | -3.735457306 |
| CALHM1 | 0.44165473 | 0.946797452 | 2.210687785 | 0.042589586 | 0.373169783 | -3.735967376 |
| SLC16A1 | -0.596906097 | 3.373677795 | -2.21063501 | 0.042593909 | 0.373169783 | -3.736049308 |
| SPTSSB | -0.709612612 | 2.201278389 | -2.210508344 | 0.042604288 | 0.373169783 | -3.736245953 |
| RAET1K | -0.704754267 | 1.463997902 | -2.210508227 | 0.042604298 | 0.373169783 | -3.736246135 |
| SSR4P1 | -0.560411748 | 3.976819922 | -2.210342949 | 0.042617844 | 0.373169783 | -3.736502715 |
| lnc-MAP1LC3B2-2 | 0.80801502 | 3.80643198 | 2.210273707 | 0.04262352 | 0.373169783 | -3.736610205 |
| THAP2 | -0.49289545 | 3.626272732 | -2.210224124 | 0.042627586 | 0.373169783 | -3.736687176 |
| FOCAD | -0.270147617 | 8.422620668 | -2.210199866 | 0.042629575 | 0.373169783 | -3.736724833 |
| lnc-RAI1-1 | 0.644340409 | 4.42241776 | 2.21007562 | 0.042639763 | 0.373169783 | -3.736917704 |
| BUB3 | -0.367740565 | 10.13404684 | -2.209741363 | 0.042667185 | 0.373169783 | -3.737436556 |
| DPH2 | -0.370908301 | 6.89092282 | -2.209725244 | 0.042668507 | 0.373169783 | -3.737461575 |
| PCDH9-AS2 | 0.484689456 | 1.391746966 | 2.209622822 | 0.042676913 | 0.373169783 | -3.737620553 |
| lnc-TNN-1 | 0.486623732 | 4.507395142 | 2.209616573 | 0.042677426 | 0.373169783 | -3.737630251 |
| ME3 | -0.73197351 | 5.555569367 | -2.20960136 | 0.042678675 | 0.373169783 | -3.737653864 |
| LCN2 | 1.291770797 | 6.31973906 | 2.209341079 | 0.042700044 | 0.373184937 | -3.738057851 |
| POLR3G | -0.634184096 | 3.279789314 | -2.209318498 | 0.042701899 | 0.373184937 | -3.738092897 |
| MAMDC4 | 0.362300239 | 4.888016656 | 2.208928054 | 0.042733974 | 0.373282814 | -3.738698869 |
| MED19 | -0.236695315 | 8.331750874 | -2.208752328 | 0.042748418 | 0.373282814 | -3.73897158 |
| CLYBL-AS1 | -0.389765091 | 1.013024773 | -2.208726433 | 0.042750547 | 0.373282814 | -3.739011765 |
| lnc-SPATA17-2 | -0.627288 | 1.858873938 | -2.208658999 | 0.042756091 | 0.373282814 | -3.739116414 |
| IRF1 | 0.404045644 | 9.654770209 | 2.208242553 | 0.042790344 | 0.373417563 | -3.73976265 |
| ASPM | -0.828906933 | 4.918357972 | -2.208113823 | 0.042800937 | 0.373417563 | -3.739962401 |
| TBC1D13 | -0.173807194 | 10.15334643 | -2.208079267 | 0.042803781 | 0.373417563 | -3.74001602 |
| NLRP12 | 0.669732343 | 9.681283099 | 2.207835213 | 0.042823872 | 0.373483307 | -3.740394701 |
| SOX9-AS1 | 1.058284419 | 1.527004628 | 2.207698353 | 0.042835143 | 0.373483307 | -3.740607048 |
| LOC728673 | -0.665103054 | 2.606293051 | -2.207595937 | 0.042843579 | 0.373483307 | -3.74076595 |
| HIST1H2AI | -0.612994212 | 3.845969799 | -2.207208183 | 0.042875532 | 0.373503975 | -3.741367533 |
| S100A11 | 0.52776282 | 11.79080096 | 2.20707276 | 0.042886697 | 0.373503975 | -3.741577623 |
| ELAVL1 | -0.303497795 | 4.939101868 | -2.206979129 | 0.042894417 | 0.373503975 | -3.741722877 |
| lnc-GAPDH-3 | -0.513397278 | 2.905152017 | -2.206942952 | 0.042897401 | 0.373503975 | -3.741778998 |
| lnc-CD83-2 | 1.291766888 | 6.581538299 | 2.206914804 | 0.042899722 | 0.373503975 | -3.741822664 |
| MGAT5 | -0.326835627 | 10.51957525 | -2.206400511 | 0.042942159 | 0.373697883 | -3.742620441 |
| LARP4 | -0.404581302 | 8.341929913 | -2.206384094 | 0.042943514 | 0.373697883 | -3.742645907 |
| HNRNPA1L2 | -0.45288806 | 11.00655696 | -2.206056436 | 0.042970572 | 0.37383967 | -3.743154128 |
| GZMA | -0.727322444 | 12.44155667 | -2.205651762 | 0.043004011 | 0.374036893 | -3.743781759 |
| lnc-EIF3M-2 | -0.467192033 | 1.016810833 | -2.205188901 | 0.043042289 | 0.374106931 | -3.744499572 |
| C9orf89 | 0.220493893 | 11.2137372 | 2.205172446 | 0.04304365 | 0.374106931 | -3.74452509 |
| TRAM1 | -0.413075896 | 10.03593382 | -2.205163628 | 0.043044379 | 0.374106931 | -3.744538763 |
| ZNF547 | -0.50466938 | 3.145535065 | -2.204490623 | 0.043100094 | 0.374132983 | -3.745582343 |
| LILRA6 | 0.594240317 | 10.5880236 | 2.204409044 | 0.043106852 | 0.374132983 | -3.745708831 |
| LINC01220 | 0.530341935 | 4.177599417 | 2.204336579 | 0.043112856 | 0.374132983 | -3.745821186 |
| ELMSAN1 | -0.212282173 | 8.138670805 | -2.204056433 | 0.043136074 | 0.374132983 | -3.746255532 |
| KCNA3 | -0.513281154 | 7.982228048 | -2.203976063 | 0.043142737 | 0.374132983 | -3.746380134 |
| ZNF394 | -0.109820364 | 9.236133328 | -2.20385413 | 0.043152847 | 0.374132983 | -3.746569171 |
| SEC13 | -0.303394241 | 11.67933581 | -2.203744071 | 0.043161975 | 0.374132983 | -3.746739795 |
| RPS15 | -0.338888605 | 14.50321466 | -2.203722657 | 0.043163751 | 0.374132983 | -3.746772993 |
| SMCR5 | -0.713344518 | 1.244593171 | -2.203690227 | 0.043166441 | 0.374132983 | -3.746823268 |
| CDIPT | 0.287260695 | 6.459698413 | 2.203651577 | 0.043169648 | 0.374132983 | -3.746883185 |
| HCG4 | -0.895856952 | 2.471357124 | -2.203502 | 0.043182058 | 0.374132983 | -3.747115062 |
| MAPKAPK5 | -0.410842685 | 6.547853619 | -2.203449833 | 0.043186387 | 0.374132983 | -3.747195932 |
| ZNF778 | -0.33109873 | 5.330923339 | -2.203437375 | 0.043187421 | 0.374132983 | -3.747215244 |
| ZGRF1 | -0.457532009 | 5.253104488 | -2.203275184 | 0.043200883 | 0.374156277 | -3.747466665 |
| DEDD2 | 0.347752482 | 9.112348695 | 2.203056431 | 0.043219046 | 0.374183886 | -3.747805752 |
| NDUFA12 | -0.425103838 | 9.613761861 | -2.20268074 | 0.043250256 | 0.374183886 | -3.748388071 |
| NR3C2 | -0.577643251 | 5.188018731 | -2.202610103 | 0.043256127 | 0.374183886 | -3.748497553 |
| GNL1 | -0.361690574 | 5.781671261 | -2.202440067 | 0.043270261 | 0.374183886 | -3.74876109 |
| B3GALT5-AS1 | 0.303509838 | 7.476573832 | 2.202402166 | 0.043273412 | 0.374183886 | -3.748819831 |
| DNAJC30 | -0.36893636 | 7.603613362 | -2.202094994 | 0.043298959 | 0.374183886 | -3.749295884 |
| lnc-DNAJB11-3 | 0.575838771 | 4.104860766 | 2.202079099 | 0.043300281 | 0.374183886 | -3.749320517 |
| SNORA27 | 0.455047731 | 5.52196801 | 2.201846314 | 0.043319651 | 0.374183886 | -3.749681266 |
| PRPF38A | -0.250633351 | 8.159506636 | -2.201724281 | 0.043329808 | 0.374183886 | -3.749870373 |
| MPZL1 | 0.657641281 | 9.37259683 | 2.201714528 | 0.04333062 | 0.374183886 | -3.749885487 |
| S1PR3 | 0.535793899 | 7.45285561 | 2.201565006 | 0.043343069 | 0.374183886 | -3.750117187 |
| EEF1G | -0.444833823 | 13.73406654 | -2.201551922 | 0.043344158 | 0.374183886 | -3.750137461 |
| ITPR3 | -0.43369016 | 7.345302127 | -2.201419794 | 0.043355162 | 0.374183886 | -3.750342199 |
| XLOC_l2_000423 | 0.931897292 | 1.682254752 | 2.201199098 | 0.043373547 | 0.374183886 | -3.750684167 |
| GPR123 | 0.278023849 | 1.076286777 | 2.201196895 | 0.04337373 | 0.374183886 | -3.750687581 |
| ZNF442 | -0.601821293 | 3.053161586 | -2.201164182 | 0.043376456 | 0.374183886 | -3.750738267 |
| ABCB8 | -0.391168514 | 6.104682015 | -2.200931278 | 0.043395867 | 0.374258375 | -3.75109913 |
| DDIT4 | -0.632180308 | 8.51402464 | -2.200700668 | 0.043415095 | 0.374272764 | -3.751456423 |
| ST8SIA3 | 0.621066911 | 3.012693073 | 2.200527652 | 0.043429527 | 0.374272764 | -3.75172447 |
| SLC25A46 | -0.351825565 | 6.883505882 | -2.200523585 | 0.043429866 | 0.374272764 | -3.751730772 |
| DNMBP-AS1 | 0.376564179 | 1.070157108 | 2.200283408 | 0.043449906 | 0.374302512 | -3.752102854 |
| CAMK2D | -0.365074477 | 6.291477563 | -2.199970288 | 0.043476046 | 0.374302512 | -3.752587913 |
| EDC3 | -0.234421943 | 6.693409878 | -2.199969968 | 0.043476073 | 0.374302512 | -3.752588408 |
| TRAPPC5 | 0.219193019 | 10.06170803 | 2.199965716 | 0.043476428 | 0.374302512 | -3.752594995 |
| lnc-CREG1-1 | 0.793458502 | 2.555603449 | 2.199562608 | 0.043510101 | 0.374310017 | -3.753219407 |
| KIF13A | 0.656412862 | 3.687103914 | 2.199438124 | 0.043520505 | 0.374310017 | -3.753412221 |
| SRD5A3 | -0.477270831 | 3.61251506 | -2.199400396 | 0.043523659 | 0.374310017 | -3.753470657 |
| HIST1H3I | 0.33656408 | 5.409715016 | 2.19928508 | 0.043533299 | 0.374310017 | -3.753649265 |
| FNIP1 | 0.52846645 | 8.050031564 | 2.199238272 | 0.043537213 | 0.374310017 | -3.753721763 |
| lnc-C15orf2-6 | -0.263641701 | 0.774529777 | -2.199178811 | 0.043542185 | 0.374310017 | -3.753813857 |
| TFB1M | -0.393999167 | 6.687541361 | -2.198931413 | 0.043562877 | 0.374310017 | -3.754197019 |
| LOC101930240 | 0.419325172 | 4.737489258 | 2.198877227 | 0.043567411 | 0.374310017 | -3.754280937 |
| DIS3 | -0.649317186 | 1.951039882 | -2.198794905 | 0.043574299 | 0.374310017 | -3.754408429 |
| MSANTD3-TMEFF1 | -0.506154927 | 1.156097316 | -2.198332412 | 0.043613016 | 0.374507303 | -3.755124648 |
| SFXN2 | -0.87602677 | 3.241868782 | -2.198262968 | 0.043618832 | 0.374507303 | -3.755232183 |
| OR10A5 | 1.010908143 | 2.821644825 | 2.198024467 | 0.043638813 | 0.374586253 | -3.755601495 |
| TRPC6 | 0.317555354 | 8.475512724 | 2.197881401 | 0.043650803 | 0.374596586 | -3.75582302 |
| WDR33 | -0.265817583 | 8.299855984 | -2.197715115 | 0.043664742 | 0.37460014 | -3.75608049 |
| GJA3 | -0.642755404 | 2.008774021 | -2.197619146 | 0.043672789 | 0.37460014 | -3.756229081 |
| NAT6 | -0.310791433 | 6.004100756 | -2.197461711 | 0.043685993 | 0.374620871 | -3.756472834 |
| OR10H4 | 0.827050335 | 1.477324629 | 2.197291854 | 0.043700242 | 0.374622351 | -3.75673581 |
| NDUFS1 | -0.181919523 | 10.29439075 | -2.197202516 | 0.043707739 | 0.374622351 | -3.756874121 |
| ST6GALNAC2 | 0.636165729 | 7.932134456 | 2.196833415 | 0.043738723 | 0.374717533 | -3.757445528 |
| C2orf44 | -0.398849973 | 5.132838702 | -2.196813173 | 0.043740423 | 0.374717533 | -3.757476863 |
| GPR113 | 0.439456478 | 0.904880192 | 2.196547014 | 0.043762779 | 0.374727649 | -3.757888877 |
| CECR5-AS1 | -0.517280552 | 4.497769997 | -2.196493743 | 0.043767255 | 0.374727649 | -3.757971337 |
| FKBP2 | -0.38621045 | 11.48233592 | -2.196413795 | 0.043773973 | 0.374727649 | -3.758095091 |
| IMMT | -0.307402693 | 5.585193464 | -2.196124129 | 0.043798321 | 0.374762476 | -3.758543454 |
| MILR1 | 0.465859756 | 7.06991869 | 2.19589044 | 0.043817974 | 0.374762476 | -3.758905153 |
| lnc-RNF38-2 | 0.518099208 | 1.264565992 | 2.195827459 | 0.043823271 | 0.374762476 | -3.75900263 |
| ASPRV1 | 0.793415971 | 9.103533209 | 2.195555978 | 0.043846115 | 0.374762476 | -3.759422795 |
| NKRF | -0.363525208 | 7.426440774 | -2.195518508 | 0.043849269 | 0.374762476 | -3.759480785 |
| TMEM161B | -0.401378745 | 7.255966391 | -2.195488292 | 0.043851812 | 0.374762476 | -3.759527547 |
| LOC100130920 | 1.730522881 | 7.580970398 | 2.195365189 | 0.043862175 | 0.374762476 | -3.759718059 |
| PPL | 0.555626287 | 1.233669753 | 2.195253641 | 0.043871568 | 0.374762476 | -3.759890686 |
| TBCD | -0.195564181 | 9.814689424 | -2.195002286 | 0.043892739 | 0.374762476 | -3.760279656 |
| EPRS | -0.42186787 | 9.489632102 | -2.194866686 | 0.043904164 | 0.374762476 | -3.760489487 |
| NOP58 | -0.531009871 | 10.7218204 | -2.194817384 | 0.043908319 | 0.374762476 | -3.760565778 |
| PRIM1 | -0.378488386 | 6.405381375 | -2.194760051 | 0.043913151 | 0.374762476 | -3.760654493 |
| TNFRSF17 | -1.483597559 | 6.72040235 | -2.194698713 | 0.043918321 | 0.374762476 | -3.760749406 |
| DSC2 | 0.923369044 | 5.212353078 | 2.193772726 | 0.043996439 | 0.37533685 | -3.762182094 |
| NAV1 | -0.595108148 | 3.273268172 | -2.193608629 | 0.044010296 | 0.375362861 | -3.762435957 |
| lnc-KCTD10-1 | -1.006300059 | 2.260992841 | -2.193395119 | 0.044028331 | 0.37542449 | -3.76276625 |
| lnc-AC079341.1-2 | 0.385711387 | 1.101053669 | 2.192907245 | 0.044069569 | 0.375529716 | -3.763520917 |
| LOC102725166 | -0.357734021 | 5.422186951 | -2.19286939 | 0.04407277 | 0.375529716 | -3.763579471 |
| CADM1 | -0.645120761 | 4.729986876 | -2.192865366 | 0.04407311 | 0.375529716 | -3.763585694 |
| PNP | -0.268563485 | 10.99104922 | -2.192710281 | 0.044086227 | 0.375549345 | -3.763825569 |
| OR52N5 | 1.005789378 | 1.888989935 | 2.192415668 | 0.044111156 | 0.375669553 | -3.764281235 |
| LOC101928101 | -0.621219871 | 3.547907051 | -2.191880578 | 0.044156465 | 0.375888793 | -3.765108763 |
| lnc-C5orf49-1 | 0.974118412 | 4.186124225 | 2.191712397 | 0.044170715 | 0.375888793 | -3.76536884 |
| ZNF311 | -0.438494116 | 1.246669749 | -2.19162283 | 0.044178305 | 0.375888793 | -3.765507344 |
| PYCR2 | -0.264318309 | 10.04425773 | -2.191600575 | 0.044180192 | 0.375888793 | -3.765541758 |
| LOC100131131 | 1.132361412 | 1.563345987 | 2.191283965 | 0.044207034 | 0.37599872 | -3.766031329 |
| lnc-EMX2-1 | 0.803733407 | 1.790123038 | 2.191192822 | 0.044214765 | 0.37599872 | -3.766172257 |
| PMS1 | -0.356651898 | 6.646683427 | -2.190732219 | 0.044253849 | 0.376234362 | -3.766884412 |
| ZNF200 | 0.391198337 | 8.497292478 | 2.190562738 | 0.044268238 | 0.376234362 | -3.767146435 |
| ICAM2 | -0.328101705 | 12.35168029 | -2.190363871 | 0.044285128 | 0.376234362 | -3.767453879 |
| LOC100128437 | 0.546293488 | 1.360894119 | 2.190344878 | 0.044286741 | 0.376234362 | -3.767483241 |
| FCGR1B | 0.975846663 | 10.76785472 | 2.189753306 | 0.044337021 | 0.376234362 | -3.768397716 |
| CD97 | 0.482612766 | 13.28086152 | 2.189730467 | 0.044338963 | 0.376234362 | -3.768433019 |
| ERCC4 | -0.508322615 | 4.767761249 | -2.189437817 | 0.044363857 | 0.376234362 | -3.768885365 |
| MAP3K9 | -0.667860236 | 4.138154729 | -2.18916024 | 0.04438748 | 0.376234362 | -3.769314386 |
| TRIM51HP | -0.635043221 | 2.020276121 | -2.188967734 | 0.04440387 | 0.376234362 | -3.769611907 |
| MIR181A1HG | -0.408595875 | 3.160292338 | -2.18891112 | 0.044408692 | 0.376234362 | -3.769699402 |
| MYO19 | -0.291666437 | 8.320188338 | -2.188896077 | 0.044409973 | 0.376234362 | -3.76972265 |
| TYK2 | 0.3647977 | 5.638664576 | 2.188876381 | 0.04441165 | 0.376234362 | -3.769753091 |
| lnc-SEPT9-1 | 1.525107397 | 2.935187179 | 2.188557462 | 0.04443882 | 0.376234362 | -3.770245948 |
| FABP5 | -0.397361736 | 6.962014874 | -2.188470345 | 0.044446245 | 0.376234362 | -3.770380573 |
| CDKN2AIPNL | -0.329197329 | 5.723120451 | -2.188369013 | 0.044454882 | 0.376234362 | -3.770537163 |
[truncated: 218,310 more chars]
